# Data management strategy for a collaborative research center

**DOI:** 10.1093/gigascience/giad049

**Published:** 2023-07-04

**Authors:** Deepti Mittal, Rebecca Mease, Thomas Kuner, Herta Flor, Rohini Kuner, Jamila Andoh

**Affiliations:** Institute of Pharmacology, Heidelberg University, 69120 Heidelberg, Germany; Institute of Physiology and Pathophysiology, Heidelberg University, 69120 Heidelberg, Germany; Institute for Anatomy and Cell Biology, Heidelberg University, 69120 Mannheim, Germany; Department of Cognitive and Clinical Neuroscience, Central Institute of Mental Health, Medical Faculty Mannheim, Heidelberg University, 68159 Mannheim, Germany; Institute of Pharmacology, Heidelberg University, 69120 Heidelberg, Germany; Department of Psychiatry and Psychotherapy, Central Institute of Mental Health, Medical Faculty Mannheim, Heidelberg University, 68159 Mannheim, Germany

## Abstract

The importance of effective research data management (RDM) strategies to support the generation of Findable, Accessible, Interoperable, and Reusable (FAIR) neuroscience data grows with each advance in data acquisition techniques and research methods. To maximize the impact of diverse research strategies, multidisciplinary, large-scale neuroscience research consortia face a number of unsolved challenges in RDM. While open science principles are largely accepted, it is practically difficult for researchers to prioritize RDM over other pressing demands. The implementation of a coherent, executable RDM plan for consortia spanning animal, human, and clinical studies is becoming increasingly challenging. Here, we present an RDM strategy implemented for the Heidelberg Collaborative Research Consortium. Our consortium combines basic and clinical research in diverse populations (animals and humans) and produces highly heterogeneous and multimodal research data (e.g., neurophysiology, neuroimaging, genetics, behavior). We present a concrete strategy for initiating early-stage RDM and FAIR data generation for large-scale collaborative research consortia, with a focus on sustainable solutions that incentivize incremental RDM while respecting research-specific requirements.

## Introduction

Extensive efforts have recently been made to promote the reproducibility, replicability, and transparency in scientific research. The evolution of open-access publishing [1], open-source data repositories [2], and open-source software applications has transformed the work of researchers in various fields. As research has become more sophisticated, these developments were inevitable, and large-scale multidisciplinary projects have been developed to promote collaborative work.

Research in the field of neuroscience increasingly encompasses a variety of fields, including biophysics, molecular biology, medicine, cognitive neuroscience, psychology, and ethology. Neuroscience datasets are constantly growing as a result of scientific advances in acquisition systems that produce large-scale multimodal datasets [3–7]. Moreover, research institutions are increasingly involved in interdisciplinary collaborative research. Such collaborative developments pose new challenges for research data management (RDM) [8], specifically in terms of data harmonization, use of computational resources, and data sharing [9]. Integration of neuroscientific datasets and data sharing are among the greatest obstacles in large-scale consortia that combine multimodal and multisite studies. These challenges become even more pronounced if they are not dealt with from the beginning and can have a direct impact on research collaborations and the publishing process [10].

The primary goal of this report is to describe our approach to implementing a data management strategy across a collaborative research consortium (Heidelberg-based collaborative research center [CRC] 1158). The consortium comprises independent, multidisciplinary research groups with a common goal-oriented research. Additionally, we aim to provide recommendations and guidelines for best practices. We specifically describe our ongoing RDM efforts, which are divided into 2 sections: (i) RDM planning phase: evaluating common data management challenges, RDM challenges in specific projects, and CRC researchers' data management requirements and (ii) RDM implementation phase: implementing common RDM procedures across consortium projects, resource allocation decisions, and key resources. This report discusses our experience in developing and implementing a data management strategy and offers concrete solutions to promote multidisciplinary collaborative research and open science objectives.

### Consortium-wide RDM planning phase

The RDM planning phase is critical to ensuring effective and efficient RDM. During this phase, we assess common and project-specific data challenges, review the scope and objectives of the consortium and its research projects, collect information on data acquisition, implement data management policies and procedures, and create a comprehensive data management plan. This plan must take into account the type of data being collected, stored, and secured; the analysis methods; and any legal or ethical considerations, including those related to sensitive data. We also review the challenges that have emerged from our consortium's efforts to create coherent RDM planning for diverse datasets, developing common infrastructure, documenting data and metadata, and establishing procedures for sharing, archiving, and handling sensitive data. This phase provides the foundation for successful and sustainable data management and compliance with laws and regulations.

### Common data management challenges across projects

#### Challenges due to the diversity in data types

While developing an RDM strategy for our CRC, a central challenge was the diversity of data types produced by multidisciplinary approaches utilized in different projects. The projects involve balancing data from basic and clinical research as well as data from animals and humans. Diverse signals are collected at various spatial and temporal scales, such as single-cell and network data, genetic (e.g., genome-wide association studies, gene expression profiles, epigenetic modifications), imaging (e.g., magnetic resonance imaging [MRI]; positron emission tomography [PET]), intra- and extracellular electrophysiology, calcium imaging using fluorescence-based microscopy, confocal light sheet microscopy, behavioral data (e.g., task performance), and clinical data (e.g., patient surveys, medication, cognitive assessments, and psychological questionnaires). These diverse techniques and collected data types raise multiple data manageability issues within and between projects, with broad implications for data interoperability and reuse. Some of these manageability issues include inconsistent data formats, limited harmonization of heterogeneous datasets, integration of multimodal datasets, difficulties in achieving and maintaining good data quality (checking for missing data and duplicates), and adhering to privacy and security regulations while enabling access to specific users.

All human research projects in our consortium use a multimodal approach that collects and combines data from 2 or more of the following methods: MRI, including structural (anatomical, diffusion-weighted imaging) and functional (task based, resting state); electroencephalography (EEG), magnetoencephalography (MEG); behavioral; psychometric evaluations; and genetics. The use of multiple data sources allows researchers to identify relationships between different modalities and to gain a more comprehensive and accurate understanding of the neural processes underlying their research topic. To improve reliability and gain further insights, datasets from multiple sources at different time points can be integrated. However, there are significant problems associated with acquiring and integrating multimodal datasets [11].

The cost of acquiring data from multiple sources can be prohibitively expensive, and the process of collecting and combining the data can be time-consuming because it frequently necessitates the use of specialized equipment and software [12]. For instance, combining functional MRI (fMRI) and EEG data [13] into a unified dataset requires knowledge of both data types and use of special software to analyze them. Interpreting the results of multimodal data can be challenging due to the complexity of the data and the potential for bias. Therefore, this can lead to overfit models and to incorrect conclusions. The complexity of the data, as well as the need for specialized software, can cause a bottleneck in data processing and analysis.

Additionally, it can be difficult to accurately integrate data from different sources due to differences in formats, storage locations, scales, and resolutions (spatial and temporal) such as resulting from fMRI, EEG, and behavior. There may be discrepancies between the data collected from different sources, making it difficult to integrate the data into a comprehensive benchmark dataset. The datasets and metadata may not be structured in a consistent way that allows for integration with other datasets or the use of more sophisticated data analysis techniques. Labeling data points is often difficult and time-consuming, making it difficult to develop accurate models. As different complex measurements become routine parts of data collection, this problem will only increase.

Moreover, data are often acquired at multiple time points, either for longitudinal assessments or due to time constraints among participants or to prevent volunteer fatigue [14]. Datasets gathered over several days are typically randomized or pseudo-randomized. This is particularly the case in some human projects where longitudinal studies are conducted [15]. Studies that involve repeated assessments typically span a long period of time (e.g., 10 years). In such studies, it is common that data are collected by different researchers, with different types of software, and new experiments may be added or removed. It is therefore essential to provide sufficient metadata as a set of documents available to download alongside the data themselves. This would support data reuse and enable accurate analysis and interpretation.

Although many advances have been made regarding the organization, annotation, and description of research datasets, there is still much work to be done to ensure that datasets are fully standardized and can be accurately shared and reused [16]. For example, data standards such as the Brain Imaging Data Structure (BIDS) [17] exist for neuroimaging data [18], EEG-BIDS for electroencephalography data [19], and MEG-BIDS for magnetoencephalography data [20], while standards for other data modalities (e.g., sensory testing, ecological momentary assessments [EMAs]) are not yet available. Particularly, integrating behavioral data is challenging, as there is a lack of clear standards and ontology that allows for generalization and thus grouping of different behavioral paradigms (see section about behavior data standardization). Finding data that can potentially be pooled remains challenging, let alone ensuring that datasets are in standardized formats for meta-analyses by third parties. Strategies for applying Findable, Accessible, Interoperable, and Reusable (FAIR) [21] principles are still under development, and standard annotation systems and clear data identifiers are crucially needed.

#### Challenges due to diversity in acquisition, preprocessing and analysis approaches

The availability of robust neuroscience resources such as high-performance computing (HPC) clusters [22–24], modern workflow technologies (e.g., Galaxy [25], Snakemake [26]), cloud-enabled storage and computing infrastructures (e.g., Amazon AWS, Google Cloud [27]), secure databases [28], repositories [29], and analysis platforms is fundamentally changing how research in neuroscience is communicated and linked to existing raw data and findings [30]. Such tools allow researchers to utilize diverse techniques and produce massive amounts of high-dimensional data (large sample size, various models and conditions), providing greater statistical power and the opportunity to perform robust secondary data analysis [31]. However, the data-driven neuroscience approach faces several technical issues that need to be resolved before its full potential can be realized.

While the majority of collaborative research consortia collect diverse multidimensional datasets, one of the primary challenges is that most of these datasets are typically inadequate for modern research methods and infrastructure [32]. Before committing to any tools for processing and analysis, it is important to understand the type, format, size, and complexity of the collected data.

Neuroscience experiments often result in incompatible datasets that cannot be compared and pooled across different research groups due to the use of custom methods for organizing and describing data. Data formats used in each project may also vary, leading to data and metadata being stored in different locations. Even if datasets are imported into a common file format, researchers' choices for data preprocessing and analysis may not be compatible between laboratories or even between different projects in the same laboratory. This is further complicated by the use of various resources, such as custom preprocessing workflows and software, which can vary widely.

Custom preprocessing pipelines and analysis scripts are another significant challenge. These pipelines are tailored to meet the specific needs of the project or research groups and may use a combination of open-source software and proprietary tools (e.g., Python scripts for examining oscillatory frequencies associated with experimental pain, followed by proprietary software for statistical analysis or visualization). As a result, researchers may need to write custom analysis scripts or to convert datasets into compatible formats to use publicly available analysis tools. The use of different preprocessing or analysis software can also result in different file input and output formats, making it challenging to compare and pool datasets. Furthermore, lab-specific workflows and pipelines usually prioritize internal needs over the needs of a broader community, which can limit the reproducibility of the research outcomes. Additionally, lab-customized software or hardware solutions may not perform efficiently on large or complex datasets. Using third-party tools and software in workflows may lead to broken dependencies and issues with reproducibility [33]. This issue is exacerbated by the fact that original analyses were done in different software environments, operating systems (e.g., Linux, Macintosh), and software versions.

One of the most pressing challenges encountered in our consortium projects is the lack of standardized preprocessing and analysis approaches [34]. Deep learning neuroimage analysis tools require significant computing power, memory, and storage, and HPC clusters can provide these resources, dramatically accelerating the analysis process [35, 36]. However, nonexperts may find it challenging to access these resources and perform scientific computing [22]. Especially for experimentalists, there is a fundamental need to provide succinct documentation on how to use these resources efficiently. To address these challenges, applications for image data processing must have application programming interfaces (APIs) and a user-friendly graphical user interface (GUI) that can be utilized without specialized coding knowledge. It is often recommended to use comparative analysis methods and multiple software packages to obtain reliable and reproducible research results. Developing such tools (e.g., bwVisu [37]) requires significant customization and software development costs, which may not be possible for individual research labs. To overcome these obstacles, experimentalists, data managers, and computer scientists must work in a close, strategic partnership.

Reproducibility and variability in published results has been a topic of investigation [38, 39], and research has shown that there is no single “best” way to process and analyze large-scale single or multimodal datasets. For instance, a neuroimaging study presented the results of a survey of fMRI experiments that revealed substantial differences in how individual labs preprocess and analyze their data. Seventy independent laboratories analyzed the same dataset and produced varying results [40]. Another study supported these findings and showed that analytical decisions made by individual researchers can significantly impact the findings from an fMRI data set [41]. Analyzing fMRI data with software packages such as SPM (Statistical Parametric Mapping) [42] or FMRIB Software Library (FSL) [43] can also lead to different outcomes. These findings emphasize the potential implications of the absence of standardized pipelines for handling complex data and how this can impact research outcomes. Efforts are being made to determine sources of variability and to develop homogeneous and standardized computing environments [44].

#### Metadata challenges

Another challenge is managing metadata [45, 46], especially for complex, large, multisite, heterogeneous datasets [47]. In an ideal scenario, all metadata related to acquired datasets would be readily accessible and sufficient for data sharing. In reality, they are not (yet). The associated metadata (such as the origin and type of a sample, experimental conditions, applied measurement techniques, devices used, calibration methods, and units) are frequently missing, incomplete, or only available in fragmented form. Additionally, datasets often lack critical details such as accuracy and variability of data points, as well as the underlying data structure. For instance, currently available datasets may not possess the resolution, annotation, or labeling required for deep learning algorithms to be applied. Even if metadata are available, extracting meaningful insights from the data may require additional tools. Knowledge of dataset quality and accompanying metadata is increasingly crucial for ensuring reproducibility [48, 49].

For most neuroimaging datasets, data annotation is highly essential. For example, when analyzing task-based data, the extent to which events are clearly documented determines an experiment's reproducibility. It is indeed important that metadata be informative about the dataset to be analyzed while following standardized ethical and quality measures. For instance, some projects in the consortium examine pain chronicity by monitoring pain patients over a period of days or years and collect various data (such as MRI and EEG) and metadata (such as pain ratings, response times, or error rate) at multiple time points. Associations between data and metadata are made to establish relationships between, for example, neural alterations and pain variables in patients with chronic pain [50] or changes in pain chronicity and associated neural networks with time [51]. Such studies could not be performed without sufficient and reliable documentation of metadata.

Moreover, a laboratory can generate a large dataset from a single experiment or a single dataset from multiple experiments. The collected metadata can be very complex and stored in multiple files with different formats, which can only be read by the acquisition software or by customized codes written for internal use. In such cases, a consolidated strategy is necessary to unify the data into a single format that can be read by various software applications and analyzed in an efficient and reproducible manner. Furthermore, depending on individual lab practices, raw data and associated metadata may be distributed across different files or separate directories. It requires additional effort to read and extract the metadata from their original raw data files and integrate them into a single file. Interoperability between file formats can be a technical issue if the appropriate software to read, view, and process the files is no longer available. It is also possible that the format is no longer supported by any software, making it impossible to open the file.

#### Data storage and volume

The data volume varies substantially depending on the data modality, ranging from a few megabytes (e.g., questionnaire data) to terabytes (e.g., high-resolution fluorescence imaging). Projects involving large amounts of data generated from high-resolution fluorescence imaging, volume electron microscopy, electrophysiology, or MRI can typically yield terabytes of data. Such data are often stored in dispersed locations and infrastructures in various formats (often proprietary), requiring a significant amount of time and effort to manage, utilize, and curate the data efficiently [52]. Researchers, particularly those working with high-dimensional data, require consistent support for data storage, timely backups, and archival systems. Inefficient data storage processes can lead to data integrity failures, accessibility issues, and increased operational costs.

Commercial cloud storage solutions are available, offering a wide range of general-purpose data backup and restoration services. However, their adoption may be challenging and limited for individual research labs or universities due to differences in data types, volumes, privacy regulations, and budgets [53]. Cloud solutions can be expensive, especially when large amounts of data or complex computing workloads need to be stored. Besides, many cloud providers may lack the necessary APIs, scripts, and tools to facilitate data migration onto analysis platforms and may not have sufficient data protection support for sensitive data (e.g., clinical data) [54].

Handling protected clinical data requires additional layers of security, privacy, and regulatory compliance. Universities may deal with sensitive data, such as research data or intellectual property, subject to strict regulations such as the European Union and the General Data Protection Regulation (GDPR [55]) and the Health Insurance Portability and Accountability Act (HIPAA). Cloud solutions require a reliable and fast Internet connection, and universities may have limited bandwidth that affects research activities. Besides, it can be difficult to migrate to a different provider or back to on-premises infrastructure, resulting in long-term dependency on a single vendor and the associated risks. Despite these challenges, cloud solutions can offer significant benefits, but research groups should evaluate their data protection needs, choose compliant cloud storage and backup services, and take steps to ensure that data are stored securely and in compliance with any relevant regulations or institutional policies. To meet these requirements, cloud providers have developed dedicated platforms and specialized backup and storage solutions designed specifically for health care organizations. However, these services cannot be easily implemented or adopted by individual labs or consortia.

Ensuring access to secure and optimal storage solutions that can be integrated with workflows encompassing data acquisition, intermediate analysis, and archiving is thus a major challenge.

#### Challenges in data documentation

Data documentation presents a number of challenges, including the adoption of digital systems and laboratory inventory management systems for large consortia. Electronic laboratory notebooks are essential for data documentation (such as hypotheses, methods, observations, experimental protocols, notes). Many efforts over the past years have recognized the critical need for institution-wide adoption and implementation of an electronic laboratory notebook (ELN) [56].

For a large-scale neuroscience consortium spanning diverse experimental protocols, it is important to select an ELN that can provide comprehensive support for a wide range of experimental protocols and flexibility to add domain-specific features if required [57]. The initial challenge is to select an appropriate option that fits into the current laboratory standards. In addition, a usable and sustainable ELN needs to be interoperable and incorporated into existing data workflows. There are obvious issues of user resistance, expensive costs involved in the implementation, and secure configuration and maintenance, and the user will be ultimately responsible for managing the digital system.

There are various open-source and proprietary options available for use [58], but it is important to note that for proprietary options, documentation may exist in the form of vendor specifications or may be created and maintained within a global community. However, it is possible that these options may not fulfill domain-specific requirements. In some cases, a key functionality that could support easy documentation is absent, and the available features may not be beneficial to users. Additionally, there may not be an automated end-to-end solution that enables users to document experiments, which can make the process time-consuming and tedious when performed manually.

When choosing an ELN, it is crucial to consider potential legal and data privacy concerns [58]. ELNs are digital resources that allow multiple users to access confidential information. It is therefore essential to select an ELN that is designed in compliance with applicable laws, regulations, and ethical standards in order to ensure legal and data privacy regulations. It is also important to consider security measures such as encryption and user authentication to maintain the confidentiality and security of stored data. Lastly, reviewing the terms of service is critical to understand how data are used, stored, and shared, as well as any restrictions of use. By taking these factors into account, organizations can ensure the security and compliance of their data. Fortunately, resources such as the ELN Matrix created by the Harvard Biomedical Data Management Group and the ELN Finder, which provides information on various software options, can be incredibly useful in this process [59, 60].

#### Data sharing and dissemination challenges

There are significant challenges in organizing datasets in a useful manner to enable sharing with collaborators. Even if a dedicated central data storage infrastructure is available, insufficient quality control measures as well as time constraints have a direct impact on data-sharing practices. Especially in small research groups or individual projects, limited funding and sustainable resources directly impact the level of data sharing and reuse. Another significant issue is motivating researchers to share data publicly. Researchers are often hesitant to openly share data due to concerns of not receiving credit, reducing their own chances of performing secondary studies, mishandling data sensitivity, and facing criticism about data quality. Despite an increasing number of research organizations, academic journals, and large-scale projects supporting extra efforts to build realistic data sharing techniques, such procedures have not yet become standard research practice [61].

Furthermore, while many journals require open data sharing and dataset submission to public repositories prior to manuscript submission, there is limited oversight of data-sharing policies. Additionally, choosing a suitable public repository could be difficult for a number of reasons. Researchers should confirm that the repository complies with the research data regulations of their host institution before contributing datasets to open repositories. Finding a suitable subject-specific repository for a given dataset could be challenging. An alternative is to submit data to a general-purpose repository, but there can be issues regarding data visibility as such repositories might not be well recognized within a particular field of research.

Submitting data to general repositories can pose significant issues, including inadequate support for certain types and formats of data [62, 63]. For example, if the datasets are in a nonstandard format, the repository may not be able to process them correctly or even accept them. Also, general repositories may lack specialized tools or services for converting, organizing, or analyzing highly specific data such as medical records or geospatial data. Without the necessary support, researchers may not be able to make full use of the data or even access them. General repositories may also lack the same level of curation and organization as for specialized repositories, leading to difficulty in evaluating the quality and relevance of the data, thereby reducing reproducibility and hindering the building on previous research. Therefore, it is recommended that researchers submit their data to specialized repositories that are tailored to their field of research. For example, neuroscience-specific repositories such as OpenNeuro are designed to accommodate the unique needs of neuroscience data, are managed by experts in the field, and provide necessary infrastructure to ensure the safe and secure storage of data. Furthermore, they often offer additional services, including data analysis, curation, and visualization tools, allowing researchers to better understand and to use the data.

Even after identifying a suitable repository, bureaucratic procedures and demands for publishing datasets in open data repositories require additional work, including converting files to the required format, compiling consent forms and contracts, removing sensitive information, and preparing documentation. Finally, maintenance funding must be taken into account because many repositories charge a fee based on the data volume. In the latter stages of the data life cycle, these factors can hamper discoverability and reusability.

#### Challenges due to sensitive data

Projects involving human subject data or other sensitive data must adhere to strict data privacy regulations for the storage, use, and sharing of research data [64]. Sensitive data containing potentially identifying information must be anonymized or pseudonymized prior to making the data public to protect participant confidentiality. Maintaining such high ethical standards can be costly and time-consuming, adding further burden to researchers [65]. Long-term preservation and sharing of sensitive data largely depend on informed consent, data reuse agreements and policies, and the type of archiving solution or data repository used. Each step of handling sensitive data must protect privacy and identity protection rights, often through deidentification or anonymization. There are distinct sets of regulations for full anonymization versus deidentification of data [65]. Therefore, it is recommended to retain multiple versions of the data: one suitable for public release and one suitable for further research but available on a highly restricted basis [66]. These considerations can lead to increased data duplication and storage needs.

The sharing of sensitive data between collaborators located in different locations requires additional effort, as data controllers need to ensure that data protection requirements are met in both the original location where the data were collected and the collaborator's location [67]. Furthermore, external collaborations across universities can present logistical challenges in the form of access and security entitlements. These concerns are compounded when the collection of sensitive data is part of the research project, or for collaborations with researchers embedded in clinical settings. Another major obstacle to sharing confidential data with external parties is the cost involved in adopting secure data-sharing platforms, as well as major risk of participants being identified. In this context, researchers require consistent training and education that promote responsible research conduct and to adhere to institutional and discipline-specific data management policies, including risks of data disclosure, confidentiality obligations, privacy principles, and network security.

#### Challenges in behavioral experiments

The increasing number of collaborative studies may be hampered by challenges in standardizing behavioral experiments across laboratories (e.g., continuous animal movement recordings, mouse trajectories). There are no specific community data standards for storing behavioral datasets, which has a direct impact on data sharing. Also, research labs may not have access to modern tools for extracting and analyzing behavior because their implementation may require advanced computational skills.

Another challenge that behavioral data present are reproducibility issues with regards to experimental results because it is often difficult to replicate the exact same conditions in which the experiment was conducted across the laboratories or even within the same laboratory [68]. It is indeed difficult to standardize metadata across behavioral experiments due to various factors that are difficult to control (confounding variables), such as laboratory environment (e.g., time of testing during light or dark phases, housing system for rodent experiments, auditory sounds) and experimenter bias, which can lead to inconsistencies in data collection.

The lack of publicly available behavioral datasets with accurate annotations is a major impediment to benchmarking algorithms used in behavioral analysis [69]. These algorithms can range from simple statistical tests to more complex machine learning models that classify, cluster, or extract features from the behavior data [70, 71]. However, due to the complexity of behavior patterns, consistent labeling of data is challenging, and the time and resources required for collecting and labeling datasets make it expensive for many labs to obtain high-quality datasets for testing and comparing algorithms.

To address these issues, it is essential to develop a centralized database for storing methods and experimental protocols of behavioral assays, parameters (e.g., sex, age, strain of the animal, genotype, marking, testing conditions), data and metadata files generated in the task (such as behavioral responses and compressed video and audio files), and a common framework that supports further analysis and visualization [72].

### RDM challenges for specific projects

Across the consortium, several projects presented specific challenges in RDM, either in data organization and governance, sheer data volume, logistics, or for collaborative data sharing. In these cases, effective data management is integral to project success and may require customized strategies and resources. Below, we list examples representing the consortium extreme cases.

#### Electrophysiology with high-density probes

A few of the animal projects in the consortium make use of technologies such as high-density Neuropixels probes [73]. Neuropixels datasets are often large (∼80 GB/hour) and computationally demanding, which can make it difficult to scale spike-sorting workflows across different labs and datasets. Data storage requirements increase due to the significant amounts of derived data needed for intermediate processing (such as filtering and spike sorting) as well as stimulation and/or behavioral parameters (such as optogenetic stimulation, motion or whisker tracking, and task performance). Analysis and postprocessing may often require computationally intensive algorithms and hardware acceleration to handle data that cannot be loaded into local memory [74].

Differences in recording conditions, spike-sorting algorithms, and data preprocessing can lead to significant outcome variability. The real-time processing requirements for closed-loop experiments only serve to exacerbate these issues. Important parameters initially recorded from raw data (e.g., animal arousal/anesthesia level, impedance measurements) might be excluded or lost in derived datasets used for analysis, which can affect the accuracy of the results. Complex hierarchies of derived data and multimodal datasets (e.g., accelerometer, whisker or pupil tracking) collected with different instruments compound these issues. It can be challenging to validate and reproduce results, because the use of various algorithms and parameters can produce different outcomes. Therefore, it is essential to carefully consider and account for all relevant parameters and sources of variability in the analysis of complex datasets.

#### Large-scale in vivo 2-photon calcium imaging

Some rodent projects within the consortium acquire large amounts of data, collected over months [75]. For example, data acquired using imaging techniques such as fluorescence imaging or 2-photon microscopy calcium imaging (2-photon imaging) generate a large volume of spatiotemporal imaging data (up to 100 GB/hour), which require rigorous preprocessing steps (image segmentation, denoising, motion correction, manipulation and handling of large video files, and neural activity deconvolution) using high-throughput computing [76]. The downstream processing and analysis of the resulting datasets generated over the course of months is often challenging and requires complex workflows [77]. A few open-source software solutions such as CalmAn [78] and EZCalcium [79] have been proposed to deal with these challenges. However, comparative analysis studies have revealed that the neural assemblies (collection of neurons that are activated simultaneously in response to a particular stimulus and form assemblies) recovered from 2-photon imaging datasets can vary significantly depending on the algorithms used. Some algorithms have been found to have high precision and slow runtimes, while others have faster runtimes but lower accuracy [80]. Another issue is that many studies include synthetic or benchmarking datasets, but the production and analysis of these datasets require challenging calculations, raising the computational complexity and costs. This highlights the need for more scalable and fully automated workflows that can be run on HPC clusters [75], in order to ensure reliability and performance [20]. Existing software solutions can be used for analysis and visualization of datasets, but any adopted data and metadata standards must be interoperable with these tools.

#### Challenges associated with human–animal tandem projects

Projects collecting data from both human and animal models pose several challenges, such as the systematic and parallel implementation of experimental designs, techniques, and analysis tools [81, 82]. Data management processes to create harmonized datasets and analysis workflows while establishing clear linkages between human and animal models are difficult, and standards for integrating data are somewhat ad hoc. In collaborative projects involving multiple laboratories working on multiple species, the integration of data and analysis should happen systematically, not only sporadically. Apart from the sheer scale of such collaborations involving multiple research areas and the multimodal RDM issues discussed above, these tandem projects require a secure platform for data transfer between different sites (e.g., laboratories and clinics) with different security permissions and data-handling standards [83].

### Consortium-wide RDM implementation phase

The implementation of an RDM strategy for a large consortium is primarily based on the various types of data generated across research projects, as well as on practical methods for organizing and managing the data. Our priority was to adequately characterize the consortium's needs before committing to any specific resources. One of the primary goals of identifying common requirements was to ensure that the best data management practices could be implemented across the consortium while taking individual lab practices into account.

Direct involvement with researchers during the planning and initial implementation phases was crucial to identifying the most helpful RDM measures. These measures may be as simple as coordinating communication between core IT staff and researchers and facilitating access to institutional or other preexisting resources. We devoted significant time to initially gathering information about publicly available tools and services that would be useful to the diverse projects within the consortium. Given the large number of laboratories from various institutions participating in the consortium and the increasing number of requirement changes over the course of a project, information was gathered in a variety of ways (e.g., virtual individual interviews with project principal investigators (PIs), discussions during online data seminars led by the CRC data manager, and personal meetings with experimentalists and PhD students). Data discussions and regular communication with consortium members have greatly aided our assessment approach.

In addition, we developed an RDM assessment questionnaire in order to tailor the data management solutions to the common needs of the projects. The PIs or project responsible persons were required to respond to a variety of data management questions about the major challenges they faced when managing data in their labs (see Supplementary Information 2).

The RDM assessment questionnaire included questions about the types of experimental models, acquisition methods and techniques, types of analysis tools and software, data modalities, raw and intermediate file formats, workflows for data preprocessing and analysis, procedures for sharing and publishing datasets, and so on. Additionally, we discussed challenges in publishing data and metadata in open data repositories.

The outcome from the RDM assessment questionnaire was then used to implement common RDM solutions for CRC projects, such as identifying and targeting data storage, organizing data, and sharing resources. In terms of research data and technological advancements, the survey response was extremely diverse. The most common challenge reported by the consortium's researchers was the sharing of large-scale datasets with collaborators and to efficiently curate the data from data archives and repositories after the project was finished.

#### Assessment of data management requirements in the Heidelberg Pain Consortium

**Figure 1:**
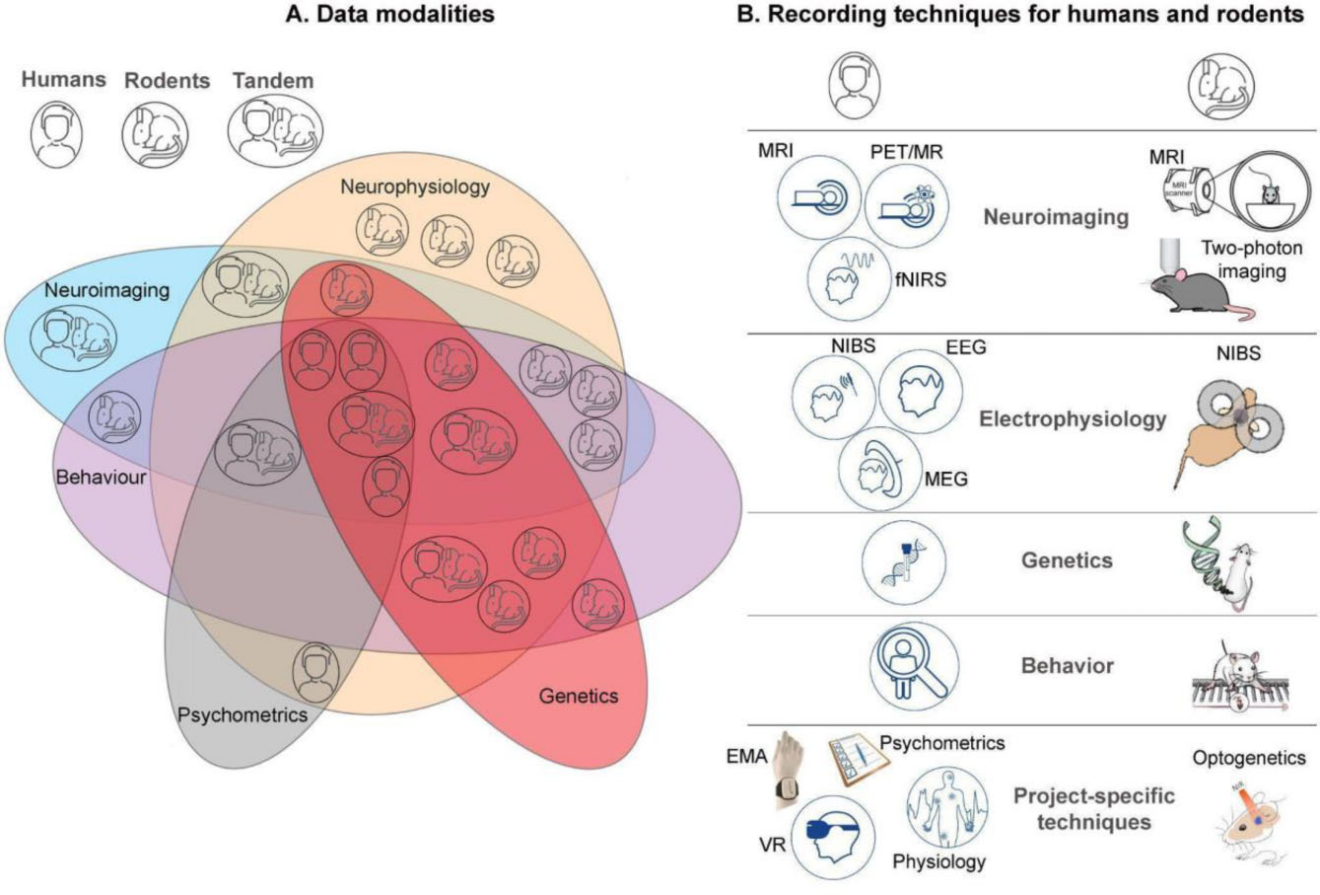
The Heidelberg Pain Consortium investigates humans, rodents, and tandem (human and rodents) using various modalities: neuroimaging, neurophysiology, behavior, psychometrics, and genetics (A). Each data modality can be recorded using different techniques: MRI: magnetic resonance imaging; PET: positron emission tomography; fNIRS: functional near-infrared spectroscopy; NIBS: noninvasive brain stimulation; EEG: electroencephalography; MEG: magnetoencephalography; Genetics; 2-photon imaging; Behavior; EMA: ecological momentary assessment; psychometrics; physiology; VR: virtual reality; optogenetics (B).

To identify common RDM measures, we first examined the commonalities between all projects, such as the type of population studied (rodents, humans, or tandem), followed by the type of data modalities acquired (e.g., neurophysiology, neuroimaging, and behavior). Human projects include data collected from both healthy individuals and patients with various clinical conditions (e.g., chronic back pain, severe depression, diabetic neuropathy) and rodent projects utilize mice as animal models (Fig. 1A).

We further categorized projects into subgroups based on common data modalities that were being acquired. Fig. 1B depicts an overview of the various data types collected across the consortium projects. Neurophysiology data (including electrophysiology and cellular physiology) are the most frequent data category collected across all studies (i.e., 83% for animal and tandem projects and 100% for human projects). Imaging, behavioral, and genetic data are collected in similar proportions in human projects (75%), whereas psychometric data are collected by all human projects. Imaging data are collected in 41.2% of the animal projects and 83% of the tandem projects. Behavioral data (e.g., various pain models) are collected in 58% of the animal projects and 66.67% of the tandem projects.

Our consortium includes research projects that typically collect data from a wide range of methods and techniques, such as electrophysiology, neuroimaging, extracellular and intracellular signals, and 2-photon imaging for rodents; behavior, including stress and fear assessment in humans and rodents; and multiomics datasets (Fig. 1B). Rodent and human projects use comparable methods, such as MRI of both the brain and peripheral nerves; electrophysiology, including EEG and MEG for humans; extra- and intracellular signals and 2-photon imaging for rodents; and brain stimulation methods, including transcranial magnetic stimulation and transcranial electrical stimulation. Additional specific methods in humans are peripheral physiology (e.g., heart rate, blood pressure, sensory profiles), virtual reality, psychometrics, and daily assessments of psychological methods such as EMA. Specific methods for rodents include optogenetics.

We gathered information on optimal storage solutions for projects, and the response was diverse, as some projects acquired large numbers of datasets (e.g., ranging from 5 TB/day to 1 petabyte), whereas other projects acquired relatively smaller datasets (e.g., a few gigabytes per month). For instance, some projects involved the continuous recording of neurophysiology datasets from high-density probes for a few days or a week at a time, which can generate up to 100 TB of data. We documented that 80% of the projects were already utilizing university infrastructure for data storage, whereas many human projects utilized individual lab servers.

We then collected information about file formats utilized for collecting and preprocessing raw data from different acquisition systems and a wide range of methods. Given the complexity and diversity of experiments and the different volume of data collected in the consortium, the acquired file formats are most often highly specific to certain data types (such as time series, e.g., voltage traces, image stacks, stimuli, or behavior) or acquisition or recording devices. Several projects require the development of new tools and software for migration to open data standards, resulting in the need for additional resources and support from the CRC.

Electrophysiology experiments, equipment, and analysis pipelines, in particular, are customized for each project and generate data in a variety of file formats. Data are collected using various techniques and experimental designs, such as patch clamp to tetrodes in freely moving animals and high-density silicon probe recordings. The steps for preprocessing for intracellular, juxtacellular, and extracellular techniques are frequently customized. Any measures to standardize must be compatible with existing lab analysis tools and data-processing methods. Despite the fact that a number of community-developed electrophysiology metadata and data standards are available and evolving, they have not yet been widely adopted.

We also collected information about the most common preprocessing and analysis software (e.g., IgorPro [84], ImageJ [85], MATLAB) utilized across different projects. Our assessment also included information regarding projects using electronic lab notebooks and those using traditional handwritten notebooks. Additionally, we assessed the definition of user permission for data access, protocols for data sharing, short- and long-term storage needs, and implementation costs.

#### RDM communication and exchange

Several neuroscience-specific RDM solutions already exist, including software and infrastructures for streamlining data collection and acquisition protocols, collaborative data analysis and visualization packages, and data sharing and archiving platforms [66, 86–88]. Our initial observation while implementing RDM strategies was that many researchers were not aware of the benefits of existing resources, partly due to uncertainties regarding the bureaucratic procedures, the GDPR, and more often, the technical requirements for easy integration of these resources into existing laboratory practices [89, 90]. Therefore, we put great emphasis on promoting and encouraging the use of preexisting resources that meet the needs of our consortium or that help in a particular use case. An important aspect was to find a balanced approach that encourages an appropriate degree of integration of existing resources with realistic domain specificity. We curated a list of both generic and neuroscience-specific RDM resources, both on the consortium/institutional (internal) and national and international (external) levels. The list can be accessed in the data management section of our CRC website [91].

#### Data infrastructure (platforms for storage, organization, analysis, and sharing of data)

A technical infrastructure was made available to consortium members based on the individual CRC project's needs and demands. We aimed for simple and efficient solutions for secure data transfer between collaborators with controlled access, all while balancing ease of access for research. Each of these services and their underlying technology, properties (e.g., sharing possibilities, availability on HPC, backup, versioning, access), technological foundation, and usage scenario are explained in the next section.

#### Data management plans for CRC projects

We designed 3 data management plan (DMP) templates after defining and categorizing the data management needs for each project depending on its experimental model type: human, animal, and human–animal tandem (see Supplementary Information 3.1, 3.2, and 3.3, respectively). The templates can also be found on Zenodo [92, 93]. DMP templates for animal, human, and tandem projects can differ depending on the scope of the project, the type of data collected, and how the data will be managed, but the major difference is the ethical considerations associated with each experimental model type. Animal projects, for instance, may necessitate additional safety protocols for the storage and management of animal tissue samples, whereas human projects require more stringent regulations and oversight for the ethical processing of human data [94]. Additionally, DMPs for human projects may include the collection of sensitive data (personally identifiable information), which must be securely stored and shared according to regulations. The DMP should adhere to GDPR-compliant guidelines for handling of personal data. It should specify information and authentication measures for reuse and recovery, as well as deidentification procedures for datasets involving human participants before sharing. Additionally, DMP should include details of the publication process for anonymized data. Human–animal tandem projects are more concerned with resource allocation and special protocols for the integration of different data types collected from multiple sources. It is important to ensure accuracy and consistency across all sources.

The DMP states minimum requirements for metadata that must be provided for long-term preservation and secondary analysis of research data. It also contains the information about data migration and access by third parties or future collaborators even after the project ends.

#### Data storage solutions

Projects in the CRC generate massive volumes of data of various types and rely on data interoperability among labs. It is strongly advised that researchers securely store full datasets (e.g., raw, preprocessed, analysis files, codes) associated with published findings and results, as this promotes the consortium's goal of further engaging in open science.

**Figure 2:**
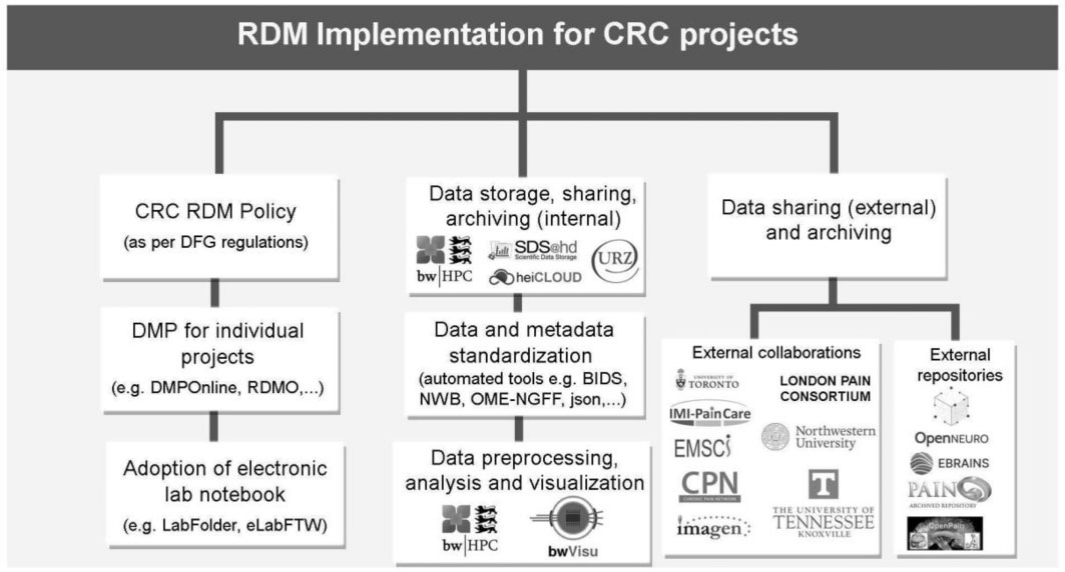
Schematic of the data management implementation for CRC projects. RDM: research data management; DMP: data management plan.

The CRC provides support for various storage solutions for all stages of the data life cycle, and the best option for a research project is determined by the type of data being collected, the size of the data, the security requirements, and the scalability of the platform. Factors such as interoperability with the existing infrastructure of the project group, reliability and accessibility for a particular storage solution, and availability of user training and service support are also taken into consideration. For example, if the research project involves collecting large amounts of data, then a cloud-based storage platform may be the best option. Additionally, cloud-based storage platforms provide access to data from anywhere with an Internet connection, making it easy for researchers to collaborate and share data with colleagues. If the data are sensitive, then a secure, on-premises storage solution may be the best choice because it can be customized to meet the specific security needs of the organization, such as encryption, authentication, and access control. Additionally, if the research project requires scalability (e.g., projects collecting terabytes of data from methods such as optogenetics, electrophysiology, and calcium imaging), then a platform that can easily scale up or down may be the best option. Ultimately, the best data storage platform for a research project will depend on the specific needs of the project, and university-approved data storage services are recommended to guarantee data privacy and confidentiality (see Fig. 2).

Most of the CRC researchers are encouraged to use our internal data storage platform SDS@hd (SDS, Scientific Data Storage, with a capacity of 20 petabytes), a central service for securely storing scientific data [95]. To facilitate easy data sharing between internal collaborators, all datasets collected from rodent experiments are shared across different research teams via SDS@hd, which stores experimental protocols, raw and preprocessed data, session (e.g., session start time), mouse information (e.g., animal weight), task files (e.g., behavioral responses, videos, audio data), metadata parameters (e.g., dimensions, pixel types, and instrumentation settings). Such procedure is intended for data that are frequently accessed (“hot data”). A common storage place (“Speichervorhaben,” meaning data storage projects) is requested for collaborative projects or work groups to ensure that data are easily accessible to all project members with proper authentications (university credentials). The Heidelberg University network connects the consortium's labs and university departments, using a 10-GB capacity network to expedite data transfer among institutes and facilities. Using SDS@hd drastically increases the ease of data access for shared projects and data safety by virtue of automated mirroring for data backup. Datasets collected from confocal or 2-photon microscopes are stored on acquisition computers temporarily (a few days), allowing researchers to quickly access, check, and transfer the data to a secure data storage platform on SDS@hd for further preprocessing and analysis.

Once the datasets are fully copied and backed up, individual users must ensure that the large datasets are timely (usually in a few days or a week depending on data volume) removed from the acquisition computers to free up the acquisition storage system for new data or for another user. The reason to remove data from acquisition computers is that such computers are often not designed for long-term storage and can be vulnerable to hardware failures, data corruption, or security breaches. To mitigate these risks, it is a common practice to copy the data immediately to the data storage platform to ensure long-term data preservation, security, and accessibility.

Apart from its storage and fast data transfer capacity, another potential reason for storing large datasets on SDS@hd is its direct access to other university platforms such as HPC systems (explained in the next section). Other university storage solutions that can be requested via support from CRC data manager include a SERVER BACKUP [96], which is a service for data storage for servers (based on a data protection and recovery software, i.e., IBM Spectrum Protect [ISP] [97]; a CLIENT BACKUP service [98], which can be accessed via on all operating systems via Duplicati software for secure storage of workstations and PCs; and HEIVOL-I [99], a service for creating network drives for university institutes and facilities). Similar services are available at the other participating CRC labs from other institutions.

For human data storage (including sensitive data), researchers use a storage server with restricted access (Dell Isilon server with a large storage and archival capacity). Designated personnel have the authority and responsibility to enable access to internal collaborators. When necessary, access can be given to external collaborators by assigning guest accounts with a data-sharing agreement in place.

#### Data processing, analysis, and visualization

Several projects and laboratories in the consortium use laboratory-based analysis infrastructure, such as local computers, shared analysis workstations (laboratory computers with GPUs and preinstalled acquisition and analysis tools that are shared by several members), and computational servers that are run by individual laboratories or groups.

The CRC highlights the importance of keeping track of every step, from initial data recording to the analysis, and proper documentation of analysis code, pipelines, and scripts. As a constructive starting point, the CRC 1158 data manager has set up a dedicated code space on GitHub [100], where multiple repositories with analysis code and scripts can be hosted and shared for each CRC 1158 project. The CRC 1158’s data management organization repositories are maintained by the data manager, and access is given only to the authorized project members.

In addition to local infrastructure and computing servers, there is university infrastructure available for more demanding data-processing tasks, such as running computationally intensive analyses of heterogeneous and large-scale imaging datasets collected from humans and rodent projects. An application for access to these services can be made by individual laboratories with an initial application and usage support from the data manager. To seamlessly integrate the data analysis with the setup and execution of preclinical experiments, a platform such as the one proposed here was necessary. Some of the CRC projects are utilizing bwForCluster MLS&WISO [101], and a detailed tutorial on access and use is made available [102] This eliminates administrative and technical barriers to performing computationally intensive tasks such as large-scale modeling, simulation, and analysis projects (e.g., Neuropixels systems). The HPC allows job scheduling using SLURM [103] and also sets up reproducible computing environments (e.g., Docker [104], Singularity [105]) to optionally run the modules on the HPC for particularly large data sets that are streamed directly to SDS@hd during acquisition, for example, chronic recordings with dense electrode array or image segmentation for chronic imaging (miniscope) with the perspective of running standardized analysis workflows. Allowing users to access large datasets stored in SDS@hd without downloading them to their local computer aids in the seamless integration of data analysis procedures, saving overall computational time and costs. The data can be accessed from the bwHPC cluster using the same protocols as for local storage, such as NFS, SMB, and FTP. This service also allows users to access data stored in SDS@hd from multiple bwHPC nodes simultaneously, thus increasing overall speed of data access. By utilizing direct data access, scientists are able to take advantage of the increased computing power available in HPC systems and gain access to data stored in a scientific data storage system.

While writing this article, bwForCluster Helix [106], a successor of the current HPC system, was made available to users. The Helix component will enable the use of seamless, cross-system workflows for processing and analysis of large amounts of data.

In our consortium's case, data management efforts had led to an increased user base for the HPC machines. Many factors played into a user's decision to use a particular HPC machine, such as its performance, cost, and availability. Data management efforts had made the HPC machines more attractive to users by providing individual support and training for access and use. The amount of training necessary to promote the use of HPC machines in a research lab depended on the particular needs and existing infrastructure. Generally, training and seminars covered topics such as high-level usage of programming languages, high-performance computing paradigms, and best practices for using HPC machines for neuroscience data processing and analysis. It also included guidance on how to design and optimize applications for HPC systems (e.g., refactoring of tools for direct access or use on HPC and bwVISU). A lab might also need to provide additional training for data management and analysis or for using specific software packages.

Similarly, for processing massive datasets (e.g., neuroimaging), some projects are utilizing the heiCLOUD [107], an infrastructure-as-a-service cloud service that provides virtual machines that may be customized and utilized as needed for the project. A possible scenario for heiCLOUD usage within our consortium is to install complex and computationally expensive software packages and perform concurrent processing of massive neuroimaging datasets. This provides powerful workstations for data analysis and can be especially useful for collaborative research projects that require data sharing between multiple research teams.

Another service that is frequently accessed by CRC members is heiBOX [108], a secure sync-and-share service hosted on heiCLOUD. This service is similar to commercial cloud storage services like Dropbox and Google Drive and allows users to save, synchronize, share, publish, and jointly edit files. heiBOX is based on the Seafile software [109] and allows users to search for text files, PDF files, and Office files in unencrypted libraries using a full-text search. Additionally, Office files can be edited by multiple people, and files and folders can be tagged and commented on. Markdown documents can be used to create private or public wikis. heiBOX also provides backup, synchronization, and storage of small research data and document files, and guest accounts can be requested for data exchange with external collaborators. The most common use of heiBOX in our consortium is to share documents such as CRC meetings notes, data seminar and workshops presentations, individual projects DMPs, manuscripts, and figures.

Another application that some of the CRC projects are utilizing is bwVISU [37], a remote service for scientists (universities in Baden-Württemberg state, Germany), as well as the corresponding software stack to deploy such a service on premises. It has an interactive web front end that supports large-scale data analysis and visualization without much human intervention. Our RDM services also include providing technical assistance with the refactoring of lab-customized preprocessing analysis pipelines (MATLAB and Python scripts) into more organized workflows/GUI that can be run on HPC applications.

#### Metadata documentation and standardization

To standardize datasets generated within the collaborative studies across the consortium, we prioritized data documentation as a key first step. We encouraged consortium-wide adoption of ELNs to help researchers document experimental protocols in well-annotated electronic form at an early stage of the research project [110]. In order to select an appropriate option for our consortium projects and take individual lab requirements into account, we tested multiple ELN options based on factors such as licensing, security, implementation and maintenance costs, ease of access and integration with existing resources, and domain-specific features that may be required. We selected 2 options: elabFTW [111, 112].

The Competence Centre for Research Data (Kompetenzzentrum Forschungsdaten [KFD]), a joint institution run by the Library and Computing Centre at the University of Heidelberg, established a fully encrypted, web-based elabFTW instance [113] with secure cloud-based data storage. Our computing center's local installation of the elabFTW, including secure cloud-based data storage, has proven beneficial to individual researchers in our consortium, serving as a central repository for shared experimental protocols. To facilitate the transfer of experimental protocols from traditional notebooks or digital documents to ELN in a consistent manner, additional time and effort were required from project members. To overcome this potential burden, we created templates for the most common types of experiments performed in a single project (based on design protocols, biological methods). By developing such templates, we were able to guarantee the seamless integration of existing protocols into ELN and ensure easy access to them at all stages of the experiment. Despite the initial investment, most labs found the benefits of ELNs outweighed any additional burden by saving time and effort in the long run.

In our RDM framework, we use ELNs as a platform to document experimental protocols alongside minimal metadata generated automatically during an experiment. Most commonly, we use elabFTW, which accepts JavaScript Object Notation (JSON) files. elabFTW acts as a “notebook,” tracking both primary data (experiment findings, measurements, etc.) and metadata (date, time, author, units, used inventory, etc.). The experimental metadata (e.g., microscope specifications, data acquisition settings) is stored in a standardized manner using a generic metadata file format that is compatible with open file formats, such as JSON or XML.

However, ELNs are not usually designed as full-fledged metadata systems and may lack domain-specific functionalities, such as support for file formats specific to neuroscience. Integrating metadata generated during different stages of a neuroscience research project (such as experimental design, data acquisition and preprocessing, statistical analysis, visualization, and dissemination) into ELN presents a significant challenge. Most ELNs do not offer sufficient support for the diverse data or metadata file formats generated during these intermediate steps, which makes incorporating additional metadata challenging without an automated tool or API, which can be time-consuming.

In addition to the limitations of ELNs, many neuroscience datasets (e.g., electrophysiology experiments such as single-unit recordings or local field potential [LFP] recordings) lack consistent metadata schema even at the most basic level. This means there is no standardized structure or format and terminology for storing metadata such as experimental parameters (e.g., stimulus type, duration, intensity, and location), animal or subject information (e.g., species, age, sex, and weight), recording equipment and settings (e.g., amplifier type, sampling rate, and filtering), data-processing parameters, and analysis methods. To add, search, filter, or use various types of metadata effectively, specialized tools are often necessary. These tools are designed to handle the complexity and diversity of metadata formats and to streamline the process of metadata management and integration with data analysis workflows. Without such tools, it can be challenging and time-consuming to work with large, complex datasets that contain multiple types of metadata.

For instance, when converting complete datasets, such as raw, preprocessed, and analyzed files from an electrophysiology experiment, into a standardized open data format, the resulting basic metadata file is frequently insufficient and lacks important information such as analysis parameters, spike sorting, or filtering parameters.

Therefore, we face the challenge of integrating comprehensive metadata, including experimental, acquisition, and analytical metadata, into a single file with a format compatible with open data standards. To address these challenges of inconsistent metadata schemas, lack of tools for metadata management, and insufficient support for domain-specific file formats in ELNs, we developed custom tools (explained in the next section), which enable to standardize experiments or data modality–specific standardization and to automate the data documentation process.

#### Data standardization in rodent projects

In order to devise practical solutions for harmonizing diverse neurophysiology datasets with diverse file formats, we divided our standardization approach into 3 main steps: (i) comprehensive metadata documentation, (ii) adoption of open data standards, and (iii) standardized preprocessing and analysis workflow HPC environment.

To ensure comprehensive metadata documentation and standardization of neurophysiology datasets collected across rodent projects of the consortium, we collaborated with Catalyst Neuro, a neuroscience software solutions company [114], to design a web-based metadata standardization GUI. The source code and installation guide are available for use [115]. The metadata handling GUI allows standardized documentation of metadata (experimental information, acquisition and analytical parameters, etc.) collected from neurophysiology experiments [116].

The metadata handling GUI generates JSON files based on the initial set of JSON schemas for different experiment types (e.g., extracellular electrophysiology and optical physiology). These schemas incorporate ontologies (structured and controlled vocabularies) to describe the data and associated metadata, including fields such as the type of population studied, type of data collected, date and duration of the experiment, and the equipment used. The GUI uses a centralized data dictionary that contains key metadata fields and possible values sourced from the consortium.

In addition to facilitating routine metadata entry through default field values, the GUI ensures that all necessary information is included and kept in a consistent format. It generates JSON files that incorporate standardized details of the experiment and analysis parameters, as well as raw file information. These files can be saved locally or centrally and imported into ELNs and open data repositories.

While eLabFTW is useful for recording experimental protocols, the GUI provides extra features such as automatic data validation and support for specific metadata formats. It can be easily customized to fit project requirements, facilitating more flexible and standardized metadata management across different platforms and tools. Overall, the GUI improves the efficiency and reliability of workflows within the lab and enables submission of complete datasets for archiving and future use.

Standardizing electrophysiology datasets recorded with Neuropixels probes involves the use of an open-source acquisition system (Neuralynx system [117] and open-source acquisition software such as SpikeGLX [118] and Open Ephys [119]). Several electrophysiology projects within the consortium focus on using the Neurodata Without Borders (NWB) data standard [120]. The NWB 2.0 format is based on the Hierarchical Data Format version 5 (HDF5) and organizes files in a hierarchical structure that contains metadata, data, and processing code. It supports a wide range of data modalities, including electrophysiology (extracellular and intracellular recordings, electrocorticography) and optophysiology (2-photon imaging, fluorescent wide-field images, etc.). NWB 2.0 format contains all the metadata required to specify the neurophysiology experiment parameters, such as voltage having a sampling rate and being connected to electrodes, and the data can be shared between labs in a fully standardized format.

The NWB 2.0 data standard uses the JSON format for metadata files and a JSON schema to define the metadata structure and validate the metadata content. This ensures that the metadata conform to a consistent format and contain all the necessary information for data sharing and reuse. The JSON metadata files in NWB 2.0 are typically associated with HDF5 files that store the actual data, enabling efficient storage, querying, and analysis of large neuroscience datasets.

Our metadata-handling GUI produces JSON files based on JSON schema that follow the same NWB 2.0 standard. This enables easy conversion of datasets into the NWB format and simplifies the process of sharing and using data across the consortium's research groups and projects. Moreover, our GUI's ability to generate JSON files in the same format as NWB 2.0 allows for easy data conversions between different formats, further facilitating the sharing and use of data across different research labs within the consortium.

Another useful strategy was the use of open-source analysis and visualization software packages such as SpikeInterface [121, 122], which supports import and export of data in NWB format. It elegantly solves the problem of importing hardware-specific acquisition formats into a common environment while also providing preprocessing capabilities and streamlined access to a variety of spike-sorting algorithms. The best way to ensure accuracy and reliability in the results from different spike sorters when looking at the Neuropixels probes is to use a consistent, well-defined analysis workflow. Using HPC clusters and remote visualization platforms (e.g., bwVisu), it was possible to overcome the challenge of real-time processing of large electrophysiology datasets from multiple recordings. Running similar preprocessing and analysis workflows in a single HPC environment allows for efficient data processing and ensures that the results are consistent and reproducible.

Additional assessment techniques in animals include EEG, MRI, and PET. The datasets generated by microscopic imaging techniques and by a variety of acquisition devices, such as the repetitive in vivo multiphoton imaging experiments conducted over a 20- to 24-week period in living mice, are challenging to standardize. Researchers collect and view microscopic imaging data from different vendor-specific acquisition software in diverse file formats (e.g., TIFF, multipage TIFF, Nikon ND2, Leica LIF, Leica CZI, or ZVI). It is often difficult to read metadata from these files in other software. While TIFF is the most commonly used file format because it is easily accessible by many current analysis software platforms, it has some limitations such as long latency and delayed data access while working with large batches of files [123].

Our standardization strategy focuses primarily on tools that are interoperable with preexisting services such as local data storage platforms used for storing imaging datasets, bioimaging software applications (ImageJ/Fiji, etc.) [85, 124] utilized for analysis of data, the type of ELNs adopted within the consortium labs, and the use of HPC clusters. The objective is to create a feasible level of automatic interoperability with existing data analysis and visualization tools as well as ELNs. We utilize existing bioimaging application Fiji [125], which supports the import and export of multiple imaging acquisition file formats. Fiji also allows automated extraction and display of metadata from raw files (e.g., .TIFF, .LIFF) using its own Bio-Formats plugin [126], including Bio-Formats Importer and Exporter, Bio-Formats Macro Extensions, Data Browser, and so on. We have not yet decided to use one single data standard across the imaging projects, but evaluating these community proposed formats will enable us to implement solutions that will allow users to link the experimental metadata (design protocols, biological methods, etc.) with microscope specifications, image acquisition settings, and analysis workflows in a more comprehensive metadata file (JSON or OME-XML format) [127]. We are currently setting up a modular pipeline for exporting the data into more open-source and standardized formats such as a next-generation file format OME-NGFF [128] and Microscopy-BIDS format (an extension to BIDS for microscopic imaging data) [129]. Our standardization pipeline will also allow the incorporation of missing metadata values and will allow users to create more fields in order to support the other diverse acquired file formats.

#### Data standardization in human projects

All human research projects acquire multimodal data (neuroimaging, neurophysiological, behavior, psychometrics, etc.). Within the plan of implementing good practices for data management, we followed recent developments in data standards and methodologies to make data interoperable. For this purpose, we created some standard protocols for each data type, along with the associated metadata.

For instance, to standardize MRI data acquisitions, we developed magnetic resonance (MR) acquisition protocols for anatomical (e.g., T1-weighted images) and functional scans (e.g., echo planar imaging [EPI]) in terms of image resolution, type of acquisition, and duration. The use of the same acquisition parameters with consistent terminologies across studies allowed researchers to use similar preprocessing and analysis pipelines, promoting efficiency and reproducibility. Such homogeneity in data acquisition enables also the pooling of data across projects, which is particularly suitable to increase sample size or for comparison purposes. For example, we can directly compare the structure and function of various patient populations acquired in different projects (e.g., to examine commonalities and differences between individuals with chronic back pain and fibromyalgia patients).

Our goals of data integration and homogeneity were facilitated by the recent opening of the Center for Innovative Psychiatric and Psychotherapeutic Research (CIPP) [130], an extensive, modern research infrastructure with access to neuroimaging, pharmacological, and psychotherapeutic techniques. In this center, the researchers share the laboratories and equipment, which allows the collection of homogeneous data types and data formats for behavioral (e.g., motor) or sensory (e.g., quantitative sensory) testing. In addition, we set up a core set of standardized assessments (e.g., motor paradigms, the use of electronic diaries for pain assessments, quantitative sensory testing, stress-induced analgesia) and psychological questionnaires (e.g., hospital anxiety and depression scale (HADS), multidimensional pain inventory (MPI) [131, 132]) to be used across all relevant studies.

We have made significant progress in clinical projects involving human studies by using the BIDS [17] data standard for anonymization, organization, and annotation of neuroimaging and behavioral data [18, 133]. BIDS also includes support for other multimodal data, longitudinal and multisession studies, and physiological metadata collected during MRI experiments.

For example, a typical MR brain acquisition includes anatomical scans (e.g., T1-weighted images) and functional scans (e.g., EPI) and have predefined directory labels in BIDS nomenclature, respectively, “anat” and “func.” In BIDS, metadata fields common across all subjects are specified in a single JSON file in the root directory instead of multiple files repeated for each subject. Organizing the data according to the BIDS standards ensures that the metadata are automatically included in the metadata file, eliminating the need for manual input and saving time and effort in processing large amounts of data. Moreover, adoption of BIDS enabled the development of workflows for automated data extraction, curation, and labeling. For example, automatic extraction of a minimal set of BIDS-compatible metadata can be performed using dcm2niix [134].

Regarding data storage, a secure storage server is used for anonymized data in accordance with accepted ethical and quality standards to maintain data protection and privacy. The original sensitive data are stored separately with restricted access to reduce the risk of disclosure or unauthorized access. The anonymized data to be analyzed are then uploaded to the laboratory server. The server is used as a shared infrastructure where a set of open-source software (e.g., freesurfer [135], FSL [136], fMRIPrep [137], QSIprep [138)] is installed for data preprocessing and analysis. We are using custom scripts for the anonymization of MRI datasets. Currently, these scripts are written in MATLAB, but we envision developing a modular automated tool with an interactive GUI. The custom codes used for anonymization, preprocessing, and analysis are available online via GitHub and released under the BSD license [139]. The resulting datasets in their BIDS format can also be validated using BIDS-Validator (open-source code available at GitHub [140] and the online tool [141]). After anonymization and quality control, the datasets are available for sharing within and outside of the laboratory. The data can also be made publicly available with proper security measures.

#### Behavioral data standardization

To facilitate some level of behavioral data harmonization within CRC, we have adopted a very simple and intuitive approach. Numerous studies from the past have shown that adopting standard operating procedures (SOPs) and standardizing experimental conditions across labs for multisite, large-scale projects led to more accurate and reproducible results [142–146]. All rodent projects adhere to standardized experimental procedures for behavior assays. We strongly encourage each project to share its SOPs, experimental site conditions, hardware, software, acquisition software, and preprocessing and analysis pipelines. To ensure consistency, we have provided SOPs to control variables such as mouse strain, age, and weight range. We have adopted a simple approach for storing metadata for behavior datasets. For projects that combine behavioral data with any other type of experimental data (e.g., electrophysiological recordings or neuroimaging), we prioritize the use of the same metadata file format (e.g., JSON, XML) that is incorporated in the adopted data standard (e.g., NWB, BIDS) for other data types. This helps to define parameters for behavioral paradigms and facilitates the integration of datasets. Although this approach is not fully automated, it does provide an initial level of data documentation, which will help to promote further standardization [69].

Acquisition of standard human behavioral data has been facilitated by a service project aiming at training researchers and homogenizing acquisition protocols. Moreover, the CIPP infrastructure enabled researchers to collect homogeneous data, acquired using similar equipment, resulting in similar formats. For example, quantitative sensory testing experiments have been standardized across projects in terms of measured variables and output saved as .csv files. However, some projects collect additional data that are specific to their patient population and therefore do not have any standards yet (e.g., defining body markers that trigger referred sensations or defining the modality [e.g., sensory, motor] that evokes phantom pain in amputees). Additionally, framerates or resolutions of videos recording tracking data during virtual reality experiments are also project specific and should be documented.

#### Data dissemination in CRC

Our consortium's collaborations with national and international neuroscience initiatives such as EBRAINS [147] and the NFDI bioimaging initiative in Germany, NFDI4BIOIMAGE [148], promote data sharing and encourage all projects to share large datasets (such as electrophysiological datasets from Neuropixels, cellular imaging datasets from preclinical projects) with external collaborators from the international community. Similarly, data harmonization efforts significantly aid in the sharing of large human imaging datasets in open data repositories.

The consortium's data policy encourages the submission of published datasets to repositories and the publication of open-access articles. Unless specifically exempted datasets, all consortium research data must be made available via a suitable data publishing or archiving platform under appropriate authorization and licensing (for example, a Creative Commons or open-source initiative-approved [software] license) to allow for flexible public reuse. Any third-party data gathered by or provided for consortium research activities are equally subject to these standards, unless data use agreements clearly restrict it. We assist consortium members in archiving and publishing data on heiDATA [149], an institutional repository for research data based on the DataVerse Project [150]. This repository supports data documentation as well as administrative, technical, and descriptive metadata; each dataset is given a persistent identification, a citable address, and a DataCite ID [151]. In addition to data publication, the heiDATA repository allows data access via a simple interface. This provides for the permanent publication of data records in the repository while also providing a separate interface for regular data access. Complete datasets are collected in a dataverse established for CRC 1158 projects (research data, code, documentation, and metadata) [152].

The Research Data Competence Center (KFD) also provides specific guidelines and procedures on data repositories, archiving, licensing, and access restrictions in order to provide public access to these datasets. The KFD is currently developing heiARCHIVE [153], a digital long-term archive for research data preservation and archiving, which will be available to the CRC 1158 during the next funding period or near the end of the current funding period [154]. This service will provide researchers with an easy-to-use end-user platform for archiving their research data (at least for 10 years), as well as the option of performing open archival information system (OAIS)-compatible long-term preservation with features such as format recognition, validation, and file conversion of appropriate file formats.

## Discussion

Open science and data sharing are increasingly promoted by funding organizations and research groups. However, individual scientists often find it challenging to prioritize FAIR procedures amid competing research needs. In practice, applying FAIR standards involves enormous constraints on researchers, many of whom are under immense time pressure to deliver outputs and may lack practical or conceptual RDM expertise. Implementing effective RDM strategies has the potential to improve the efficiency and accuracy of research and reduce the amount of time spent on data management by individual researchers [155].

Knowledge transfer, data reuse, and sharing may reduce redundant research [156, 157]. One of the goals of data management is to make study results freely available through open-access publishing. By investing in collaborative projects with long-term goals, it ensures that the data are organized in a way that makes them easily accessible and retrievable for future use. This makes it easier and faster to develop new research projects, as well as to replicate or build on existing studies, which should have a direct impact on public research funding. Effective data management strategies can indeed enable the optimization of public research funding by pooling resources and infrastructure from multiple sources and bringing together experts from universities, research institutes, and other community organizations to work on long-term interdisciplinary projects. Furthermore, by ensuring proper RDM, researchers should be able to reduce animal use. For example, making informed decisions about which animal models to use for their studies should enable them to use the same animals for multiple experiments, instead of having to continuously use new animals for each study. In addition, sharing previously acquired data with adequate metadata or reusing control group data from similar studies can avoid repeating in vivo work [158–161].

We have learned from our own experience of implementing RDM activities across the consortium that many projects do not fully realize the benefits of available infrastructure and resources. This is partly due to uncertainties regarding the organizational and technical requirements and a lack of knowledge of existing resources. Therefore, we place great emphasis on promoting and encouraging the use of available generic tools and infrastructures whenever possible. Our RDM implementation strategy is based on the flexible and easy integration of existing, maximally generic components to support researchers in implementing specific solutions for data collection, processing, analysis, storage, publication, and, when appropriate, the development of sustainable, project-specific infrastructures, such as data and metadata standardization tools.

The central strategy of the consortium involves coordinated cross-species analyses in experimental animal models and in human subjects. To achieve this, we use multiscale imaging; electrophysiological, psychometric, and behavioral readouts; and a range of interventional strategies across both rodent and human populations. Our consortium involves translational research projects, with the goal of translating animal research into human applications or from basic science to treatments and therapies that benefit patients. To ensure the long-term preservation of valuable data sets, we developed a CRC RDM policy (see section about funding information and supplementary Information 1) in accordance with the German Research Foundation (Deutsche Forschungsgemeinschaft [DFG]) guidelines for research data handling [162]. The CRC data policy serves as a recommended guideline for individual projects on how to format their data. The acquisition of heterogeneous data in multiple projects can render the process of data formatting challenging, and the data policy guidelines do not specify a level of granularity for data formatting. However, they do provide general recommendations, such as using standard data formats and tagging data with descriptive metadata, for formatting such diverse datasets. To support individual research groups, we provide resources and funds for the implementation of modern tools and infrastructure that are compatible with community RDM standards. For instance, we recommend using “standard” formats for data of similar modalities (e.g., neuroimaging data [MRI] formatted to NIfTI [Neuroimaging Informatics Technology Initiative], electrophysiology data formatted to NWB) as described in the data standardization sections.

Our data policy places emphasis on the significance of data documentation and sharing, while also promoting the use of open-access repositories to facilitate data sharing. In addition, our RDM services provide general support for research groups, such as assistance with deploying cloud-based data storage solutions (e.g., Amazon S3, Google Cloud Storage, and Microsoft Azure), establishing data governance policies, using automated software to streamline data storage and retrieval, and educating researchers about data privacy and GDPR regulations, among others.

### Data stewards, community engagements, and collaborations

To effectively implement data policy and governance procedures in large consortia, the presence of data managers and stewards is crucial [163]. These roles are ideally suited for individuals with a background in research, computer science, bioinformatics, and strong communication skills. Additionally, candidates should have experience in developing high-throughput analysis pipelines, domain-specific data structures and standards, open-access publishing, modern data science approaches, high-performance computing environments, cloud computing, data security, and databases, among other things, depending on the consortium's needs. The data manager's diverse role involves working closely with core computing and library resources to streamline access to the consortium's common research data infrastructure, which is available at the host institutions of participating labs.

Data managers are responsible for supporting ongoing research, providing guidance on best practices for data handling, and keeping up to date with the latest developments in the RDM field. They act as a vital link between consortium researchers, collaborators, the university's RDM planning group and computing center, and community organizations. By bridging the gap between lab-based scientists and available technical infrastructure and services, they provide direct assistance in daily tasks such as data organization, tool selection, workflow development, and standardization, which benefit individual researchers and research groups. Addressing data management tasks early in the research timeline is essential to making the research process more efficient and ensuring the interoperability and reusability of datasets. Expert guidance on existing infrastructure and resources, such as scientific repositories, databases, and legal and ethical issues, is also necessary to promote an effective data-sharing strategy.

In our consortium, the data manager maintains consistent communication with various research groups and other consortia to establish a community network and links to other scientific communities, such as the National Research Data Infrastructure (NFDI) consortium. This ensures the dissemination of data throughout the community and the development of data management techniques that are specifically designed to facilitate neuroscience research. Our consortium actively engages in numerous international and national RDM initiatives, including NFDI4BIOIMAGE and EBRAINS, which promote the development of high-level infrastructures and services across various scientific disciplines. Our active engagement in diverse task areas of these community-led initiatives, including Neuromorphic Computing, Data Analytics, Workflows, GDPR, The Virtual Brain Cloud, and so on, is instrumental in supporting the development of a sustainable and community-oriented RDM strategy.

To ensure the harmonization of our RDM efforts, our CRC follows the recommendations of the International Neuroinformatics Coordinating Facility [164, 165] and employs community-developed standards that have gained international recognition for neurophysiology and neuroimaging datasets, such as BIDS and NWB (see list [166]). Moreover, we utilize resources like FAIRsharing [167, 168] and the UK Digital Curation Centre [169] to provide a comparative overview of data and metadata standards. In addition, we participate in the Research Data Alliance [170] and European Open Science Cloud [171, 172] initiatives to adopt and develop novel resources for open data exchange across technologies and scientific disciplines.

We also focused on developing RDM strategies that included joint efforts and cooperation between consortium members and other large-scale consortia and collaborative centers within Germany. We acknowledge the common issue of data organization for different projects in collaborative centers. Joint efforts were made for the development of a data organization strategy that works for most of the projects within the consortium working in similar research areas. Our main goal was to engage more directly in several overlooked aspects of managing data in a large collaborative consortium while keeping the global neuroscience community in mind.

We recommended the CRC 1158 project members to utilize logical file and folder templates to support systematic data organization. Consistent folder organization depends on the type of research data acquired for a project as well as on the governance procedures. Our goal is to provide researchers with an easy way to manage their project digital files and datasets on different data infrastructure services, both locally and on subject-specific data repositories such as GIN: a Modern Research Data Management System for Neuroscience. For this purpose, we use folder structure templates for research repositories developed in collaboration with the NFDI Neuroscience (NFDI-Neuro) consortium (currently nonfunded) and 3 neuroscience CRCs (CRC 1158, CRC 1315, and CRC/TRR 135), [173]. The template structure is available on Zenodo [92, 173].

These templates are designed to reflect the typical workflow of a research project. This means that the structure is organized in a way that makes it easy to track the different stages of data acquisition, processing, and analysis. Depending on the specific needs of the CRC projects, we customize the templates based on the type of experiment or data modality as well as the analysis processes that should be integrated with existing data organization systems. The template structure includes separate sections for raw data and analyzed data, as well as for documentation and code related to each stage of the workflow. To illustrate, neuroimaging datasets such as MEG or fMRI that are in the BIDS format can be efficiently organized and stored in the “03_data” directory. The raw data (BIDS raw, e.g., NIfTI and JSON) can be stored in the subfolder “001_defaultexp” (default experimental data), and the analyzed data (BIDS derivatives, e.g., fMRIPrep, SPM, FSL, FreeSurfer, QSIprep) can be stored in the subfolder “999_processed_data.” It is recommended to add workflows and code libraries used for data analysis to the designated analysis directory (i.e., “04_data_analysis”). These folder structure templates facilitate reproducibility and data sharing, and they can be utilized on different storage devices to accommodate various data sets generated during experiments, independent of their format.

### Sharing sensitive data from human projects

The sharing of human data gathered from clinical or nonclinical populations in neuroscience research is essential for advancing science and producing important public health benefits. However, a clear set of regulations and guidelines must be established before sharing human data gathered from clinical or nonclinical populations. Specific rules addressing privacy issues, established processes for data protection, data use and reuse, and the preservation of sensitive data are required. It is essential to make data accessible and understandable to remote (or future) collaborators in order to maximize the potential of existing algorithms and tools and accelerate the creation of new ones. Regulations and guidelines should ensure that the data are used for the purpose for which they were gathered and protect the rights of participants in the research. These guidelines should also cover how data should be collected, stored, shared, and destroyed. They should also specify the types of data that must be kept confidential and the appropriate methods for handling and safeguarding the data. Additionally, regulations should ensure that the data are secure, kept confidential, and not used for marketing or other commercial purposes. Furthermore, regulations should ensure that the data are used responsibly and not used to discriminate against people with disabilities or other vulnerable populations. Ethical rules for the reuse and sharing of data should be based on the principle of informed consent. This includes obtaining consent from the original data collectors or from research participants, as well as obtaining permission from any third parties involved in the data collection. Researchers should also seek to minimize the risk of data misuse or breach of confidentiality.

Additionally, there is a lack of efficient software programs that can adequately segregate and maintain control over sensitive data. Developing effective software that is secure, user-friendly, and cost-effective can be difficult. The maintenance of such software requires a significant investment of resources, and often there is a lack of funding available for such measures. Furthermore, the adoption of such software requires an investment in training and resources, which many organizations may be unwilling to do.

The legal and ethical requirements surrounding the use of sensitive data are often complex and difficult to understand, leading to confusion and ambiguity about the best way to protect them. It is important to provide researchers working with sensitive data with truly “useful” tools that do not require preexisting, in-depth knowledge of legal and ethical requirements or time to delve into the details. Such tools are essential to ensure that sensitive data are protected and securely stored.

The use of such tools can help researchers make informed decisions about how to best use and manage sensitive data, allowing them to work with them in an ethical and responsible manner. Finally, these tools can help to reduce the risk of data breaches and data misuse, which can have serious consequences for the people and organizations whose data are affected. By providing such tools, researchers can focus on their research rather than legal and ethical considerations, saving time and resources.

Several software tools can be used to maintain sensitive patient data in neuroscience research, such as a web-based platform REDCap (Research Electronic Data Capture) [174], an open-source imaging informatics platform XNAT (XNAT Central) [175], and LORIS: Longitudinal Online Research and Imaging System [176]. It is important to note that the security features of these tools may vary and should be evaluated before use. In addition to software tools, secure data storage and access protocols should also be in place to ensure that sensitive patient data are protected.

Currently, we are expanding our collaborative efforts by creating a data infrastructure platform that will establish a GDPR-compliant data registry called the PainReg registry. It is based on the Germany-wide ParaReg registry [177, 178] for human volunteers. To facilitate cross-project data merging, a core clinical dataset will be defined, assigning a unique identifier to each study participant that is shared by all projects. This allows researchers to determine if a volunteer has participated in multiple projects, thereby helping to reduce redundant data acquisition. This can result in cost and time savings, as well as increased data collection accuracy. For example, we found that the same study participant could be tested twice and assigned different IDs, belonging to different projects, resulting in redundant data acquisition and unnecessary increased costs, particularly for genetic analysis.

Furthermore, the data registry will ensure that data privacy regulations are strictly followed by obtaining participants' consent to access data for secondary or follow-up studies. This will also include an identity management feature to limit access to authorized users. The registry will contain a wide range of data, including brain imaging, genetic, cognitive, and physiological. This collaborative work will be coordinated by the consortium's future data infrastructure project, which will be tasked with implementing, testing, optimizing, and standardizing data analysis procedures and models to be utilized in all projects.

### Data integration

For collaborative research, the step of data integration and standardization is crucial for interoperability and data sharing [179], but it can be quite challenging to implement given the wide range of methodologies represented in the consortium. Early standardization of data can have massive benefits for data integration in collaborative projects. This can be achieved by streamlining the use of tools for more replicable and reproducible analysis. The data integration process often depends on the individual projects and their underlying workflow and processes. The degree to which this is possible will depend on the modalities used, the subject population, and the experimental design. Researchers may also integrate the raw data collected from each partner into a core dataset. Integrated datasets can provide a more comprehensive understanding of the research question, as well as allowing the researchers to compare the results of their analyses more directly. Depending on the modalities used, the data may need to be transformed or normalized before integration, and the analysis techniques may need to be adapted to the combined dataset.

For some projects, researchers can even analyze data from their partners and vice versa. In some human projects, fMRI data from one group and EEG data from another group are combined to gain a better understanding of how the 2 modalities interact. This may involve combining datasets or running analyses on the combined dataset to identify common patterns or trends. However, this process requires careful consideration of the data sources, data formats, and analysis techniques used by individual labs, as well as the selected methods for data fusion and data mining. At the most basic level, researchers can compare the data collected from each partner to identify commonalities and differences in the data. This could include comparing the number and types of modalities used, the subject population, the experimental design, and the type of analysis performed. For example, they could investigate how brain structure (e.g., gray matter volume, cortical thickness) relates to behavior. There are also association studies aiming to compare brain activity between 2 groups of participants (e.g., healthy, chronic pain) to explore neural differences in cognition or behavior. They could also examine associations between neural activity in different brain areas and physiological responses of the subject.

Furthermore, researchers can use deep learning algorithms to identify patterns in the data and gain insights that can enhance the understanding of the brain. For instance, deep learning algorithms can detect patterns in EEG data to identify different states of consciousness or seizures. Additionally, artificial intelligence techniques can be employed to combine multiple datasets to gain a better understanding of the complex relationship between brain and behavior.

From our own experiences, we have realized that a systematic effort in developing standardized guidelines for multimodal data acquisition would strongly facilitate the data integration process and promote the adoption of FAIR data standards across all studies. Our CRC is developing a multimodal digital intervention platform that aims to combine data collected by the CRC projects for further analyses. This platform benefits from an increased sample size, which should result in improved prediction accuracy, with the potential to optimize therapies [180], such as the use of invasive or noninvasive neurostimulations. Our current efforts in harmonizing and standardizing datasets (e.g., using BIDS) and preprocessing approaches would also facilitate this development and further improve data analysis.

### Data standardization

We have devised a set of strategies to ensure that the datasets can be thoroughly documented and converted into open data standards with a minimum amount of effort. The consortium's projects combine datasets from electrophysiological recordings, optogenetic manipulations, rodent behavior assays such as sensory testing (von Frey filaments), cold plate test and open field test [181, 182], 2-photon in-vivo, and MRI, resulting in a plethora of disparate file formats and unorganized metadata. These datasets are saved in a variety of file formats, including video files (.avi), the original raw ASCII log files, text-based file formats (.csv), and detailed stimulus material (e.g., .wav and .png files), among others. We are utilizing deep learning–based approaches for behavior data acquisition, analysis, pose estimation, and so on for both human and rodent experimental models [183, 184]. This includes software packages tools such as Noldus EthoVision XT [185] (behavior data acquisition and analysis from rodents), Bonsai [186] (behavioral tracking and closed-loop experiments), ANY-maze (RRID:SCR_014289 [187] (automated video-tracking software), PsychoPy [188] (data acquisition and analysis from humans), SPSS (Statistical Package for the Social Sciences), MATLAB and GraphPad Prism [189] (data analysis and visualization), and DeepLabCut (markerless pose estimation) [190, 191] for measuring rodent behavior [192]. However, there is no single coordinated data standardization strategy for every stage of behavioral data collection to data analysis, making harmonization and thus grouping different behavioral paradigms difficult [69, 193].

Electrophysiology datasets are collected using a variety of proprietary file formats for raw data and intermediate preprocessing/analysis, such as Cambridge Electronic Design Spike2 (.smrx) and Neuralynx (.ncs). However, many of these formats are only read or accessible through proprietary software, imposing additional constraints on adoption of open data standards. Despite this, development of open data standards is often encouraged by the community and supported by interoperable tools for data import and export, validation, and analysis, which can form the core of standard workflows. Nevertheless, attempting to include all raw and intermediate preprocessing file types within a single standard format proved to be impractical within our consortium. Such an approach would be unrealistic given the diverse range of data modalities and analysis applications commonly employed. Instead, it was important to adopt an open data format that included consistent metadata structures, which enabled thorough metadata description. In this regard, the development of local and customized solutions became essential.

Additionally, as we started exploring and assessing available resources for standardizing data and metadata, including open-source tools, data conversion pipelines, and file formats and their specifications, we identified several open-source tools and data formats [194] specific to neurophysiology that could potentially be utilized for our consortium's cases. This includes data formats (e.g., Neuroscience Information Exchange [195–197], NWB [120, 198, 199]), data versioning tools (e.g., DataLad, a US–German collaboration for computational neuroscience project [200, 201], GIN [202]), metadata collection tools (e.g., CEDAR [203]), NIDM [204], open metadata markup language (odML) [47, 205], data representation models (Neo [206]), data analysis tools (e.g., Electrophysiology Analysis Toolkit, Elephant [207]; FieldTrip [208, 209]), simulation interfaces (e.g., PyNN [210]), and so on.

The data formats used are compatible with a wide range of modern software packages and analysis tools, including Brainstorm, Elephant, Spike2, and NeuroExplorer, with the majority of these tools being open source. The CRC has also encouraged the development of new tools and pipelines to reduce the time and effort required by individual labs.

For example, the NWB data conversion tools [211] can be used to build conversion pipelines that convert additional raw file formats (e.g., .rhd from the Intan RHD recording system, .smrx from Spike2) into standard data formats. This approach is advantageous for data handling within the laboratory and the development of standardized acquisition and analysis workflows. Another useful resource is the BIDS-animal-ephys extension [212], which supports other types of neuroscientific data, such as electrophysiological data recorded in animals.

### Data infrastructure

#### Adoption of project-specific DMPs

Funding agencies and research organizations are increasingly requesting DMPs when submitting a grant application. The obligation to submit a DMP depends on the requirements of the funding organizations. DMPs should be created early on, ideally when applying for funding or at the beginning of a research project, and updated as needed. For example, european research council (ERC)-funded projects that participate in the Horizon 2020 Open Research Data pilot are required to submit the first version of their DMP within 6 months after the start of their grant. Open-access publications are encouraged, and grantees should demonstrate FAIR-compliant data management and resource use. However, some research studies involving sensitive data are exempt from these requirements.

The DMP developed for each research project highlights relevant information regarding research data and associated metadata that are required for research result reproducibility. Preliminary versions of DMPs can surely assist participating labs in making informed decisions about their data management resource requirements (financial support or personnel). DMPOnline [213] and RDMO [214] are 2 commercial open-source software solutions for creating custom DMPs [215, 216]. Several DMP templates have already been made available in response to funding agency criteria [217].

Individual project DMPs can be created using these templates, or if a dataset requires particular RDM resources, a dataset-specific DMP can be created. These DMP templates cover questions about how data are handled at each stage of the project, including a general project description, experimental and dataset descriptions, specific data documentation (types of data and experimental models, methods for acquisition and collection, questionnaires, and analysis software), decisions on data and metadata standards and formats, and proposed plans for organization, access, sharing, short- and long-term storage, reuse, and implementation costs. The document, once prepared, explains the management of the research data acquired, reviewed, and processed as part of the CRC 1158 initiatives. The template includes some generic questions regarding best practices for each stage of the data management life cycle that may be answered early in the project, while domain-specific questions can be answered later in the project.

#### Data storage, organization, and sharing

In addition to providing support for access and use of internal university resources for data storage and sharing, the CRC also supports adoption of innovative community-developed solutions. Versioning of data sets, along with software and code, becomes critical for such projects as data files and metadata are updated over time. Even in the case of complete datasets published or submitted in a repository, versioning helps in tracking changes in the data files or metadata that are incorporated after data reuse or reanalysis. For instance, since most CRC projects run for multiple funding periods and involve extended analyses, data versioning becomes even more crucial. Researchers often modify, refine, or add to their datasets during the course of their research. Without proper versioning, it may not be possible to reproduce previous findings, which can negatively impact the credibility of the research outcomes.

This includes, for example, data comparison between various groups of pain patients collected at different funding periods, associations between various types of data modalities (e.g., data collected using electroencephalography for the first funding period and fMRI data during the next funding period), or simply comparisons between various analysis toolboxes (e.g., FSL vs. SPM).

Platforms such as DataLad [200] and GIN [202] may effectively compensate for a lack of local resources. Data hosting and sharing can also ensure data versioning and encourage reproducible management of scientific data. Both DataLad and GIN are based on git and git-annex to provide a decentralized system for the exchange of large datasets. Datalad is an open-source software package for the management of distributed datasets. It facilitates the acquisition, organization, and management of data stored in remote repositories but does not offer storage. Moreover, the GIN service can be deployed locally at all participating labs and can be used as an in-house storage server and web user interface for DataLad datasets. Datasets hosted on either platform can be accessed via git-compatible systems. Other examples of resources for supporting collaborative workflow development and integration of data hosting and processing/analysis computing resources include the Open Science Framework [218] and the Open Science Grid [219].

#### Data analysis and visualization

We are currently refactoring and developing image analysis tools for the automated running of deep learning applications on bwVISU [220]. With such extra computational resources, it is possible to set up automated analysis workflows on HPC that could allow for faster, more accurate diagnoses in near-real time. The goal of this project is to implement a deep learning API for image data processing and to provide a platform for the scientific community to directly compare and integrate data generated across the consortium projects. The development of an open-source and extensible platform to train and share deep learning models will guarantee high standards in many image analysis workflows and additionally reduce the amount of annotated data necessary for training supervised deep learning algorithms. For example, our initial efforts involve integrating the most commonly used deep learning image analysis tools to make the initial GUI more flexible for model training and inferences. Some of the considered tools include StarDist [221], Noise2Void (image denoising) [222], CellPose (cell segmentation) [223], and Elektronn3 [224] (EM data segmentation).

#### Data dissemination

Public neuroscience repositories are rapidly being developed, and a lot of progress has been made in this direction. Several data repository options can be found in online resources [225, 226]. It is worth searching for both general-purpose repositories, such as Zenodo [227], and domain-specific repositories. For example, for repositories on chronic pain, we use OpenPain [228], the Pain and Interoception Imaging Network repository [229, 230], and ENIGMA [231]. In the context of bioimaging data storage and sharing, the EMBL-EBI BioImage Archive [231] is a large-scale, centralized data resource that hosts reference imaging data. The OpenNEURO project [232], which was originally created for the free and open sharing of raw MRI datasets (old datasets available [233]), has since expanded to include datasets from other neuroimaging modalities such as MEG, EEG, and PET and has been renamed the OpenNeuro Project [234, 235]. Certain repositories require datasets to be submitted in a standardized format; for example, OpenNeuro (which accepts anonymized human-derived datasets), OMEGA (Open MEG Archive, exclusively for MEG data), and MNE-BIDS have all adopted the BIDS format (which links BIDS and the MNE-Python analysis tool for MEG and EEG data). In addition to providing basic features such as data hosting and support for metadata files, there are repositories that provide restricted data sharing and anonymization services, which are highly suitable for publishing datasets from clinical projects. The Cancer Imaging Archive (TCIA) the LONI Image Data Archive are 2 examples.

Other neuroscience-focused data repositories with specific purposes include G-node GIN for datasets derived from both human and nonhuman organisms, BrainLife (human neuroimaging) [236], Distributed Archives for Neurophysiology Data Integration [237], and Fenix-backed EBRAINS [238]. The HBP EBRAINS data curation team may assist with data submission and integration, as well as to provide defined embargo durations to allow for progressive disclosure. The European Union–funded Human Brain Project produced EBRAINS, an open European digital research infrastructure that provides one of the most complete platforms for sharing brain research data of various types, as well as spatial and temporal scales.

Aside from these domain-specific repositories, numerous well-known open data repositories, as well as sharing and management platforms, accept data from a wide range of disciplines. Zenodo [239] and Dryad, for example, are an online archive that manages research datasets with metadata and allows long-term data access via persistent identification. Figshare [240, 241], a commercial free data repository with unique features such as custom storage options, version control, visualization, metadata customization, data curation using DOI, and so on, is one example. The EMBL SourceData SmartFigure [242] focuses on the scientific figure as a sharing unit, combining data sharing and visualization. The Harvard Dataverse Network is both a platform for institutions and a data repository implemented on FAIR data principles to publish, share, reference, extract, and analyze research data. Consortia offering support and access to cloud computing, such as OpenScienceGrid, JetstreamCloud, Fenix, and the European Commission–backed European Open Science Cloud, can support analysis if institutional solutions are not available.

In addition to contributing datasets to repositories, sharing other relevant information such as experimental protocols, source code, and research software used for processing or analyzing these datasets is essential for reproducing research findings [243]. This approach can complement existing efforts to standardize complete datasets and to facilitate contributions to repositories. However, when dealing with complex projects that involve sensitive data, data sharing can become more difficult. In such cases, additional measures such as obtaining consent forms, implementing access regulations, and using anonymization strategies may be required to protect data confidentiality.

For the long-term preservation of data, researchers need permanent archiving systems, along with sufficient funds to build such archives for both internal usage and to satisfy the open data needs of journals and funding organizations. Ideally, archival systems should be developed with the user's perspective, especially in scientific settings where researchers with limited expertise in digital preservation collaborate on projects that generate a wide range of data [244].

Researchers often encounter challenges in determining the best practices for archiving and gaining access to archival systems. Researchers require assistance and support to ensure that their research data are appropriately archived and accessible to other researchers. First, researchers need guidance on best practices for archiving their research data, including how to stay informed about the storage, retention, and disposal of all research data. This is especially crucial as good archival practice requires a scheduled review of data in long-term storage. Therefore, researchers need to remain informed about these practices, whether their data are stored in an institutional or external repository. In addition, researchers need support to ensure that their data handling complies with various regulations and guidelines. This includes existing discipline-specific privacy and ethical standards, copyright or licensing arrangements, and publication and legal requirements. Meeting these regulations and guidelines is crucial to ensure that researchers' data are protected and accessible to other researchers. To address these challenges, research institutions and organizations can provide researchers with the necessary training, resources, and infrastructure for archiving their data. This includes developing guidelines and policies on data management and sharing, providing access to data repositories and storage facilities, and offering training and support on data management and archiving best practices.

The period for which data should be preserved for research purposes or archiving should be determined by prevailing standards for the specific type of research domain and should follow the retention policies of any applicable stakeholders (e.g., sponsoring institution, funding agency). For example, in the context of our consortium funded by the DFG, primary research data should be appropriately archived in the researcher's own institution or an appropriate nationwide infrastructure for at least 10 years [162].

## Conclusion

We have presented a data management strategy that we developed and put into practice within the framework of a collaborative research center, encompassing both basic and clinical research on humans and animals. To foster FAIR and open science, this strategy strives to offer practical solutions for multimodal and multidisciplinary research. This strategy is composed of adaptive and incremental phases: planning, implementation, and dissemination. Consistent communication with consortium project members during the planning and implementation phases was crucial to identify the most helpful RDM measures. We spent a considerable amount of time learning about publicly accessible tools, services, and new developments in the RDM field that could be beneficial to our consortium. We believe that this knowledge might be beneficial to other researchers.

In the planning phase, we evaluated common data management practices across projects. We categorized projects based on the typical population studied and the common measurement methods used. We focused on addressing issues such as metadata management, documentation of experimental protocols, preprocessing and analysis pipelines, data storage and data volume, data sharing, data dissemination, data archiving, and sensitive data-related issues that arise when working with highly diverse and heterogeneous data. The complexity was subsequently raised by the major RDM challenges encountered in tandem projects that work with both human and animal populations, such as including data and metadata standardization, the integration of various different data types, and the harmonization of datasets and analysis workflows.

In the implementation phase, we presented some innovative solutions based on preexisting and customized solutions developed for flexible and incremental data management solutions with a focus on research collaborations. We discussed the implementation of project-specific data management plans, structured based on data acquisition, processing, and analysis methods across the CRC 1158 projects. Relatively simple measures, such as offering ELN options for documenting experimental protocols or tutorials on HPC resource access and regular data seminars on basic RDM tools (such as data versioning tools, code and workflow management software), can improve data management practices in noticeable ways. We focused on the development of new tools for metadata organization and management depending upon the requirements of each project and the type of data collected. In animal projects, we assisted with migration from proprietary formats and supported experimental annotation and organization. Moreover, for large datasets, we provided easy access to software and tools on large web-based applications to enable interactive analysis and visualization.

For human projects, we adopted standard protocols to associate various data types with the respective metadata. In the case of MRI, we standardized MR acquisition protocols, data organization, and preprocessing pipelines. Behavioral, sensory testing, and psychological questionnaires were standardized in collaboration with a service project of CRC 1158.

The CRC 1158 emphasizes that active communication and engagement with general and domain-specific RDM community initiatives are required for the development of RDM strategies for any large-scale research consortium. Modern research infrastructure and technological advancements, such as web-based technologies for sharing data and analysis tools, provide opportunities to increase the reproducibility of research outcomes in both basic and translational neuroscience.

Further development of this RDM model with more specialized technical infrastructure is envisioned for the next period of the consortium. A federated data-sharing approach is required for multisite and multispecies projects, which will allow for the integration of data from different computer systems for participating labs that are geographically distributed without moving the data to a centralized location.

### CRC 1158 data management policies and funding information

CRCs (short SFB for German “Sonderforschungsbereich”) are university research projects that are funded by the DFG, generally for a period of up to 12 years [245]. The Heidelberg Pain Consortium [246] is a collaborative research center (CRC 1158) composed of 44 principal investigators in Germany aiming to understand the mechanisms of pain and pain chronicity to identify causal links and possible therapeutic interventions. It involves a multidisciplinary team of scientists and clinicians working on 23 different projects, including 12 animal projects, 6 tandem (human–animal) projects, 4 human projects, and 1 central administrative project. CRC 1158 includes additionally 2 service projects: the first one (tandem project) aims at establishing standard protocols, models, and ethical standards to facilitate homogeneous implementation across all human or rodent projects. The second service project (animal project) aims at developing simplified systems to accelerate the analysis of the translational potential of acquired research insights.

Since 2015, the DFG has supported CRC 1158 [247]. In June 2019, CRC 1158 was successfully renewed and got funding for another 4 years, 2019–2023, under project number 255156212 from DFG. CRC 1158 has many national (e.g., the University of Heidelberg, the Central Institute for Mental Health, European Molecular Laboratory, and German Cancer Research Center) and international collaborations (institutions located in the United States, Canada, England, and France).

The Heidelberg Pain Consortium implemented a development strategy in a central administration project (Z01) [248] to promote RDM as an integral part of the research process in order to maximize the impact of collaborative science. By implementing this strategy, the consortium was able to take a systematic and standards-based approach to documenting, archiving, and sharing its research data with collaborators and the research community, with the goal of significantly accelerating scientific progress. This RDM model is expected to evolve in response to the development of new and specialized (domain-specific) technical infrastructures.

As a CRC host institution, Heidelberg University offers comprehensive recommendations for the administration of research data [249]. CRC's data policy (available in Supplementary Data 1) highlights the use of a variety of RDM services to researchers to ensure that RDM for each project adheres to the DFG guidelines [250].

These services include aid with proper data documentation, the integration and support of open data management solutions, data storage and accessibility, the development of new tools for the adoption of open data and metadata standards, the sharing of various diverse datasets within the consortium and with external collaborators, and the dissemination of research outcomes into national and international data repositories. The policy is applicable to all researchers working in the CRC, including PIs, doctoral and postdoctoral researchers, and student research assistants. It also applies to any research project carried out within the CRC as well as to any data generated or shared (with outside sources).

## Supplementary Material

giad049_GIGA-D-22-00262_Original_Submission

giad049_GIGA-D-22-00262_Revision_1

giad049_GIGA-D-22-00262_Revision_2

giad049_Response_to_Reviewer_Comments_Original_Submission

giad049_Response_to_Reviewer_Comments_Revision_1

giad049_Reviewer_1_Report_Original_Submission Amanda Charbonneau -- 11/15/2022 Reviewed

giad049_Reviewer_1_Report_Revision_1 Amanda Charbonneau -- 3/10/2023 Reviewed

giad049_Reviewer_2_Report_Original_Submission Michael Denker -- 11/18/2022 Reviewed

giad049_Reviewer_2_Report_Revision_1 Michael Denker -- 3/9/2023 Reviewed

giad049_Supplemental_Files

## Data Availability

All supporting data are available via the *GigaScience* repository, GigaDB [251].

## Abbreviations

API: application programming interface; BIDS: Brain Imaging Data Structure; CIPP: Center for Innovative Psychiatric and Psychotherapeutic Research; CRC: collaborative research center; DFG: Deutsche Forschungsgemeinschaft; DMP: data management plan; EEG: electroencephalography; ELN: electronic laboratory notebook; EMA: ecological momentary assessment; EPI: echo planar imaging; FAIR: Findable, Accessible, Interoperable, and Reusable; fMRI: functional magnetic resonance imaging; FSL: FMRIB Software Library; GDPR: General Data Protection Regulation; GUI: graphical user interface; HDF5: Hierarchical Data Format version 5; HIPAA: Health Insurance Portability and Accountability Act; HPC: high-performance computing; JSON: JavaScript Object Notation; KPD: Kompetenzzentrum Forschungsdaten; LFP: local field potential; MEG: magnetoencephalography; MR: magnetic resonance; MRI: magnetic resonance imaging; NFDI: National Research Data Infrastructure; NIfTI: Neuroimaging Informatics Technology Initiative; NMC: Neuromorphic Computing; NWB: Neurodata Without Borders; PET: positron emission tomography; PI: principal investigator; RDM: research data management; SDS: Scientific Data Storage; SOP: standard operating procedure; SPM: Statistical Parametric Mapping.

## Additional Files

**Supplementary Information 1**. Data management strategy for the Collaborative Research Centre (CRC1158) - Heidelberg Pain Consortium.

**Supplementary Information 2**. Data Management assessment questions.

**Supplementary Information 3**. Tandem_Projects.

**Supplementary Information 4**. Human_Projects.

**Supplementary Information 5**. Animal_Projects.

## References

[1] Klump J, Bertelmann R, Brase J et al. Data publication in the open access initiative. Data Sci J. 2006;5:79–83.

[2] Marcial LH, Hemminger BM. Scientific data repositories on the web: an initial survey. J Am Soc Inf Sci. 2010;61:2029–48.

[3] Gouwens NW, Sorensen SA, Berg J et al. Classification of electrophysiological and morphological neuron types in the mouse visual cortex. Nat Neurosci. 2019;22:1182–95. 31209381 10.1038/s41593-019-0417-0 PMC8078853

[4] Juavinett AL, Bekheet G, Churchland AK. Chronically implanted Neuropixels probes enable high-yield recordings in freely moving mice. Elife. 2019;8:e47188. 31411559 10.7554/eLife.47188 PMC6707768

[5] Kleinfeld D, Luan L, Mitra PP et al. Can one concurrently record electrical spikes from every neuron in a mammalian brain?. Neuron. 2019;103:1005–15. 31495645 10.1016/j.neuron.2019.08.011 PMC6763354

[6] Sych Y, Chernysheva M, Sumanovski LT et al. High-density multi-fiber photometry for studying large-scale brain circuit dynamics. Nat Methods. 2019;16:553–60. 31086339 10.1038/s41592-019-0400-4

[7] Zeisel A, Hochgerner H, Lonnerberg P, et al. Molecular architecture of the mouse nervous system. Cell. 2018;174:999–1014.e22. 30096314 10.1016/j.cell.2018.06.021 PMC6086934

[8] Asher A, Deards K, Esteva M et al. Research Data Management: Principles, Practices, and Prospects. Council on Library and Information Resources: Washington , DC, USA, 2013.

[9] Tenopir C, Allard S, Douglass K et al. Data sharing by scientists: practices and perceptions. PLoS One. 2011;6:e21101. 21738610 10.1371/journal.pone.0021101 PMC3126798

[10] Rubel O, Dougherty M, Prabhat Denes P et al. Methods for specifying scientific data standards and modeling relationships with applications to neuroscience. Front Neuroinform. 2016;10:48. 27867355 10.3389/fninf.2016.00048 PMC5095137

[11] Lahat D, Adalý T, Jutten C. Challenges in multimodal data fusion. In: 2014 22nd European Signal Processing Conference (EUSIPCO). HAL Open Archive, Lisbonne, Portugal: IEEE, 2014.

[12] Buckow K, Quade M, Rienhoff O et al. Changing requirements and resulting needs for IT-infrastructure for longitudinal research in the neurosciences. Neurosci Res. 2016;102:22–8. 25152316 10.1016/j.neures.2014.08.005

[13] De Martino F, Valente G, de Borst AW et al. Multimodal imaging: an evaluation of univariate and multivariate methods for simultaneous EEG/fMRI. Magn Reson Imaging. 2010;28:1104–12. 20097029 10.1016/j.mri.2009.12.026

[14] King KM, Littlefield AK, McCabe CJ, et al. Longitudinal modeling in developmental neuroimaging research: common challenges, and solutions from developmental psychology. Dev Cogn Neurosci. 2018;33:54–72. 29395939 10.1016/j.dcn.2017.11.009 PMC6969276

[15] Cragg JJ, Haefeli J, Jutzeler CR et al. Effects of pain and pain management on motor recovery of spinal cord-injured patients: a longitudinal study. Neurorehabil Neural Repair. 2016;30:753–61. 26747127 10.1177/1545968315624777

[16] Poldrack RA, Feingold F, Frank MJ et al. The importance of standards for sharing of computational models and data. Comput Brain Behav. 2019;2:229–32. 32440654 10.1007/s42113-019-00062-x PMC7241435

[17] Brain Imaging Data Structure. https://bids.neuroimaging.io/. Accessed 7 June 2023.

[18] Gorgolewski KJ, Auer T, Calhoun VD et al. The brain imaging data structure, a format for organizing and describing outputs of neuroimaging experiments. Sci Data. 2016;3:160044. 27326542 10.1038/sdata.2016.44 PMC4978148

[19] Pernet CR, Appelhoff S, Gorgolewski KJ, et al. EEG-BIDS, an extension to the brain imaging data structure for electroencephalography. Sci Data. 2019;6:103. 31239435 10.1038/s41597-019-0104-8 PMC6592877

[20] Niso G, Botvinik-Nezer R, Appelhoff S et al. Open and reproducible neuroimaging: from study inception to publication. Neuroimage. 2022;263:119623. 36100172 10.1016/j.neuroimage.2022.119623 PMC10008521

[21] Wilkinson MD, Dumontier M, Aalbersberg IJ et al. The FAIR Guiding Principles for scientific data management and stewardship. Sci Data. 2016;3:160018. 26978244 10.1038/sdata.2016.18 PMC4792175

[22] Bouchard KE, Aimone JB, Chun M, et al. High-performance computing in neuroscience for data-driven discovery, integration, and dissemination. Neuron. 2016;92:628–31. 27810006 10.1016/j.neuron.2016.10.035

[23] Dinov ID, Petrosyan P, Liu Z et al. High-throughput neuroimaging-genetics computational infrastructure. Front Neuroinform. 2014;8:41. 24795619 10.3389/fninf.2014.00041 PMC4005931

[24] Perkel JM . Web service makes big data available to neuroscientists. Nature. 2018;563:143. 30377329 10.1038/d41586-018-07195-2

[25] Goecks J, Nekrutenko A, Taylor J et al. Galaxy: a comprehensive approach for supporting accessible, reproducible, and transparent computational research in the life sciences. Genome Biol. 2010;11:R86. 20738864 10.1186/gb-2010-11-8-r86 PMC2945788

[26] Koster J, Rahmann S. Snakemake—a scalable bioinformatics workflow engine. Bioinformatics. 2012;28:2520–2. 22908215 10.1093/bioinformatics/bts480

[27] Brigham TJ. Taking advantage of Google's Web-based applications and services. Med Ref Serv Q. 2014;33:202–10. 24735269 10.1080/02763869.2014.897521

[28] Amari S, Beltrame F, Bjaalie JG et al. Neuroinformatics: the integration of shared databases and tools towards integrative neuroscience. J Integr Neurosci. 2002;1:117–28. 15011281 10.1142/s0219635202000128

[29] Eickhoff S, Nichols TE, Van Horn JD et al. Sharing the wealth: neuroimaging data repositories. Neuroimage. 2016;124:1065–8. 26574120 10.1016/j.neuroimage.2015.10.079 PMC5463741

[30] Van Horn JD. Bridging the brain and data sciences. Big Data. 2021;9:153–87. 33211552 10.1089/big.2020.0065 PMC8233216

[31] Madan CR . Scan once, analyse many: using large open-access neuroimaging datasets to understand the brain. Neuroinformatics. 2022;20:109–37. 33974213 10.1007/s12021-021-09519-6 PMC8111663

[32] Fan J, Han F, Liu H. Challenges of big data analysis. Natl Sci Rev. 2014;1:293–314. 25419469 10.1093/nsr/nwt032 PMC4236847

[33] Li X, Ai L, Giavasis S et al. Moving beyond processing and analysis-related variation in neuroscience. 2021. bioRxiv. doi: 10.1101/2021.12.01.470790. 10.1038/s41562-024-01942-4 39103610

[34] Ferguson AR, Nielson JL, Cragin MH et al. Big data from small data: data-sharing in the ‘long tail’ of neuroscience. Nat Neurosci. 2014;17:1442–7. 25349910 10.1038/nn.3838 PMC4728080

[35] Avberšek LK, Repovš G. Deep learning in neuroimaging data analysis: applications, challenges, and solutions. Front Neuroimaging. 2022;1:23. 10.3389/fnimg.2022.981642 PMC10406264 37555142

[36] Council NR. Frontiers in Massive Data Analysis. Washington, DC: The National Academies Press; 2013.;

[37] bwVisu—a scalable service for remote visualization and interactive applications. https://www.bwvisu.de/. Accessed 7 June 2023.

[38] Bowring A, Nichols TE, Maumet C. Isolating the sources of pipeline-variability in group-level task-fMRI results. Hum Brain Mapp. 2022;43:1112–28. 34773436 10.1002/hbm.25713 PMC8764489

[39] Gronenschild EH, Habets P, Jacobs HI et al. The effects of FreeSurfer version, workstation type, and Macintosh operating system version on anatomical volume and cortical thickness measurements. PLoS One. 2012;7:e38234. 22675527 10.1371/journal.pone.0038234 PMC3365894

[40] Carp J. The secret lives of experiments: methods reporting in the fMRI literature. Neuroimage. 2012;63:289–300. 22796459 10.1016/j.neuroimage.2012.07.004

[41] Botvinik-Nezer R, Holzmeister F, Camerer CF, et al. Variability in the analysis of a single neuroimaging dataset by many teams. Nature. 2020;582:84–88. 32483374 10.1038/s41586-020-2314-9 PMC7771346

[42] Friston KJ. Statistical parametric mapping. In: Kötter R, ed. Neuroscience Databases: A Practical Guide. Boston, MA: Springer US; 2003:237–50.

[43] Smith SM, Jenkinson M, Woolrich MW, et al. Advances in functional and structural MR image analysis and implementation as FSL. Neuroimage. 2004;23(:Suppl 1):S208–19. 15501092 10.1016/j.neuroimage.2004.07.051

[44] Bowring A, Maumet C, Nichols TE. Exploring the impact of analysis software on task fMRI results. Hum Brain Mapp. 2019;40:3362–84. 31050106 10.1002/hbm.24603 PMC6618324

[45] Stall S, Yarmey L, Cutcher-Gershenfeld J, et al. Make scientific data FAIR. Nature. 2019;570:27–29. 31164768 10.1038/d41586-019-01720-7

[46] Stanford NJ, Scharm M, Dobson PD et al. Data management in computational systems biology: exploring standards, tools, databases, and packaging best practices. Methods Mol Biol. 2019;2049:285–314. 31602618 10.1007/978-1-4939-9736-7_17

[47] Grewe J, Wachtler T, Benda J. A bottom-up approach to data annotation in neurophysiology. Front Neuroinform. 2011;5:16. 21941477 10.3389/fninf.2011.00016 PMC3171061

[48] Laine C, Goodman SN, Griswold ME, et al. Reproducible research: moving toward research the public can really trust. Ann Intern Med. 2007;146:450–3. 17339612 10.7326/0003-4819-146-6-200703200-00154

[49] Zehl L, Jaillet F, Stoewer A et al. Handling metadata in a neurophysiology laboratory. Front Neuroinform. 2016;10:26. 27486397 10.3389/fninf.2016.00026 PMC4949266

[50] Zheng CJ, Van Drunen S, Egorova-Brumley N. Neural correlates of co-occurring pain and depression: an activation-likelihood estimation (ALE) meta-analysis and systematic review. Transl Psychiatry. 2022;12:196. 35545623 10.1038/s41398-022-01949-3 PMC9095719

[51] Hashmi JA, Baliki MN, Huang L et al. Shape shifting pain: chronification of back pain shifts brain representation from nociceptive to emotional circuits. Brain. 2013;136:2751–68. 23983029 10.1093/brain/awt211 PMC3754458

[52] Borghi JA, Van Gulick AE. Data management and sharing: practices and perceptions of psychology researchers. PLoS One. 2021;16:e0252047. 34019600 10.1371/journal.pone.0252047 PMC8139478

[53] National Academies of Sciences, Engineering, and Medicine; Health and Medicine Division; Board on Health Sciences Policy; Forum on Neuroscience and Nervous System Disorders . The National Academies Collection: reports funded by National Institutes of Health. In: Stroud C, Gee AW, Bain L, eds. Neuroscience Data in the Cloud: Opportunities and Challenges: Proceedings of a Workshop. Washington, DC: National Academies Press, 2020. 32049471

[54] Rao UH, Nayak U. Data backups and cloud computing. In: Rao UH, Nayak U, eds. The InfoSec Handbook: An Introduction to Information Security. Berkeley, CA: Apress; 2014:263–88.

[55] General Data Protection Regulation GDPR. https://gdpr-info.eu/. Accessed 7 June 2023.

[56] Foster ED, Whipple EC, Rios GR. Implementing an institution-wide electronic lab notebook initiative. J. 2022;110:222–7. 10.5195/jmla.2022.1407 PMC9014952 35440896

[57] Khan AM, Hahn JD, Cheng WC, et al. NeuroScholar's electronic laboratory notebook and its application to neuroendocrinology. Neuroinformatics. 2006;4:139–62. 16845166 10.1385/NI:4:2:139 PMC4476904

[58] Higgins SG, Nogiwa-Valdez AA, Stevens MM. Considerations for implementing electronic laboratory notebooks in an academic research environment. Nat Protoc. 2022;17:179–89. 35031789 10.1038/s41596-021-00645-8

[59] ELN Finder. https://eln-finder.ulb.tu-darmstadt.de/home/. Accessed 7 June 2023.

[60] Electronic Lab Notebook Comparison Matrix. https://zenodo.org/record/4723753/. Accessed 7 June 2023.

[61] Vasilevsky NA, Minnier J, Haendel MA et al. Reproducible and reusable research: are journal data sharing policies meeting the mark?. PeerJ. 2017;5:e3208. 28462024 10.7717/peerj.3208 PMC5407277

[62] Assante M, Candela L, Castelli D et al. Are scientific data repositories coping with research data publishing?. Data Sci J. 2016;15:6.

[63] Sandstrom M, Abrams M, Bjaalie JG et al. Recommendations for repositories and scientific gateways from a neuroscience perspective. Sci Data. 2022;9:212. 35577825 10.1038/s41597-022-01334-1 PMC9110735

[64] Sariyar M, Schluender I, Smee C et al. Sharing and reuse of sensitive data and samples: supporting researchers in identifying ethical and legal requirements. Biopreserv Biobanking. 2015;13:263–70. 10.1089/bio.2015.0014 PMC4559154 26186169

[65] White T, Blok E, Calhoun VD. Data sharing and privacy issues in neuroimaging research: opportunities, obstacles, challenges, and monsters under the bed. Hum Brain Mapp. 2022;43:278–91. 32621651 10.1002/hbm.25120 PMC8675413

[66] Eke DO, Bernard A, Bjaalie JG, et al. International data governance for neuroscience. Neuron. 2022;110:600–12. 34914921 10.1016/j.neuron.2021.11.017 PMC8857067

[67] Managing sensitive data. https://www.imperial.ac.uk/research-and-innovation/support-for-staff/scholarly-communication/research-data-management/data-storage-and-security/storing-sensitive-and-personal-data/. Accessed 7 June 2023.

[68] Voelkl B, Altman NS, Forsman A, et al. Reproducibility of animal research in light of biological variation. Nat Rev Neurosci. 2020;21:384–93. 32488205 10.1038/s41583-020-0313-3

[69] von Ziegler L, Sturman O, Bohacek J. Big behavior: challenges and opportunities in a new era of deep behavior profiling. Neuropsychopharmacology. 2021;46:33–44. 32599604 10.1038/s41386-020-0751-7 PMC7688651

[70] Cakmak E, Plank M, Calovi DS et al. Spatio-Temporal Clustering Benchmark for Collective Animal Behavior. Germany: University of Konstanz, 2021.

[71] Kabra M, Robie AA, Rivera-Alba M, et al. JAABA: interactive machine learning for automatic annotation of animal behavior. Nat Methods. 2013;10:64–67. 23202433 10.1038/nmeth.2281

[72] Sare RM, Lemons A, Smith CB. Behavior testing in rodents: highlighting potential confounds affecting variability and reproducibility. Brain Sci. 2021;11:22. 10.3390/brainsci11040522 PMC8073298 33924037

[73] Jun JJ, Steinmetz NA, Siegle JH et al. Fully integrated silicon probes for high-density recording of neural activity. Nature. 2017;551:232–6. 29120427 10.1038/nature24636 PMC5955206

[74] Steinmetz NA, Koch C, Harris KD et al. Challenges and opportunities for large-scale electrophysiology with Neuropixels probes. Curr Opin Neurobiol. 2018;50:92–100. 29444488 10.1016/j.conb.2018.01.009 PMC5999351

[75] Gangadharan V, Zheng H, Taberner FJ, et al. Neuropathic pain caused by miswiring and abnormal end organ targeting. Nature. 2022;606:137–45. 35614217 10.1038/s41586-022-04777-z PMC9159955

[76] Robbins M, Christensen CN, Kaminski CF, et al. Calcium imaging analysis—how far have we come?. F1000Res. 2021;10:258. 34504683 10.12688/f1000research.51755.1 PMC8406438

[77] Pnevmatikakis EA. Analysis pipelines for calcium imaging data. Curr Opin Neurobiol. 2019;55:15–21. 30529147 10.1016/j.conb.2018.11.004

[78] Giovannucci A, Friedrich J, Gunn P et al. CaIm: an an open source tool for scalable calcium imaging data analysis. Elife. 2019;8:e38173. 30652683 10.7554/eLife.38173 PMC6342523

[79] Cantu DA, Wang B, Gongwer MW, et al. EZcalcium: open-source toolbox for analysis of calcium imaging data. Front Neural Circuits. 2020;14:25. 32499682 10.3389/fncir.2020.00025 PMC7244005

[80] Molter J, Avitan L, Goodhill GJ. Detecting neural assemblies in calcium imaging data. BMC Biol. 2018;16:143. 30486809 10.1186/s12915-018-0606-4 PMC6262979

[81] Akhtar A. The flaws and human harms of animal experimentation. Camb Q Healthc Ethics. 2015;24:407–19. 26364776 10.1017/S0963180115000079 PMC4594046

[82] Stephens DN, Crombag HS, Duka T. The challenge of studying parallel behaviors in humans and animal models. Curr Top Behav Neurosci. 2013;13:611–45. 21671191 10.1007/7854_2011_133

[83] Suvorov A, Takser L. Facing the challenge of data transfer from animal models to humans: the case of persistent organohalogens. Environ Health. 2008;7:58. 19014546 10.1186/1476-069X-7-58 PMC2596097

[84] Igor Pro. https://www.wavemetrics.com/products/igorpro/. Accessed 7 June 2023.

[85] Schneider CA, Rasband WS, Eliceiri KW. NIH Image to ImageJ: 25 years of image analysis. Nat Methods. 2012;9:671–5. 22930834 10.1038/nmeth.2089 PMC5554542

[86] Cachat J, Bandrowski A, Grethe JS et al. A survey of the neuroscience resource landscape: perspectives from the neuroscience information framework. Int Rev Neurobiol. 2012;103:39–68. 23195120 10.1016/B978-0-12-388408-4.00003-4

[87] Litvina E, Adams A, Barth A, et al. BRAIN Initiative: cutting-edge tools and resources for the community. J Neurosci. 2019;39:8275–84. 31619497 10.1523/JNEUROSCI.1169-19.2019 PMC6794930

[88] Nayak L, Dasgupta A, Das R et al. Computational neuroscience and neuroinformatics: recent progress and resources. J Biosci. 2018;43:1037–54. 30541962

[89] Crutzen R, Ygram Peters G-J, Mondschein C. Why and how we should care about the General Data Protection Regulation. Psychol Health. 2019;34:1347–57. 31111730 10.1080/08870446.2019.1606222

[90] Jwa AS, Poldrack RA. Addressing privacy risk in neuroscience data: from data protection to harm prevention. J Law Biosci. 2022;9:lsac025. 36072418 10.1093/jlb/lsac025 PMC9444136

[91] Heidelberg Pain Consortium Resources. https://sfb1158.de/index.php/rdm-resources/. Accessed 7 June 2023.

[92] Colomb J, Arendt T, Mittal D et al. Folder structure template for research repositories (2.1). *Zenodo*. 2020. 10.5281/zenodo.4410128

[93] Mittal D. CRC1158 data management plan templates (1.0). *Zenodo*. 2022. 10.5281/zenodo.6917120

[94] Brand S, Bartlett D, Farley M et al. A model data management plan standard operating procedure: results from the DIA clinical data management community, Committee on Clinical Data Management Plan. Ther Innov Regul Sci. 2015;49:720–9. 30227041 10.1177/2168479015579520

[95] SDS@HD—SCIENTIFIC DATA STORAGE. https://www.urz.uni-heidelberg.de/en/service-catalogue/storage/sdshd-scientific-data-storage/. Accessed 7 June 2023.

[96] Heidelberg Pain Consortium Server Backup. https://www.urz.uni-heidelberg.de/en/service-catalogue/storage/server-backup/. Accessed 7 June 2023.

[97] IBM Spectrum Protect Supported Operating Systems. https://www.ibm.com/support/pages/overview-ibm-spectrum-protect-supported-operating-systems/. Accessed 7 June 2023.

[98] Heidelberg Pain Consortium Client Backup. https://www.urz.uni-heidelberg.de/en/service-catalogue/storage/client-backup/. Accessed 7 June 2023.

[99] Heidelberg Pain Consortium heivol-i. https://www.urz.uni-heidelberg.de/en/service-catalogue/storage/heivol-i/. Accessed 7 June 2023.

[100] Heidelberg Pain Consortium Data Management. https://github.com/SFB1158RDM/. Accessed 7 June 2023.

[101] BWFORCLUSTER MLS&WISO. https://www.urz.uni-heidelberg.de/de/forschung-und-lehre/forschungsnahe-projekte/bwforcluster-mlswiso/. Accessed 7 June 2023.

[102] Heidelberg Pain Consortium HPC Tutorial. https://github.com/SFB1158RDM/HPCtutorial/. Accessed 7 June 2023.

[103] Slurm Documentation. https://slurm.schedmd.com/documentation.html/. Accessed 7 June 2023.

[104] Merkel D. Docker: lightweight Linux containers for consistent development and deployment. Linux J. 2014;2014:2.

[105] Kurtzer GM, Sochat V, Bauer MW. Singularity: scientific containers for mobility of compute. PLoS One. 2017;12:e0177459. 28494014 10.1371/journal.pone.0177459 PMC5426675

[106] BWFORCLUSTER HELIX. https://www.urz.uni-heidelberg.de/de/service-katalog/hochleistungsrechnen/bwforcluster-helix/. Accessed 7 June 2023;

[107] heiCLOUD—Cloud-Infrastruktur. https://heicloud.uni-heidelberg.de/. Accessed 7 June 2023.

[108] heiBOX. https://www.urz.uni-heidelberg.de/de/service-katalog/collaboration-und-digitale-lehre/heibox/. Accessed 7 June 2023.

[109] Seafile. https://www.seafile.com/en/home/. Accessed 7 June 2023.

[110] Solle D. Be FAIR to your data. Anal Bioanal Chem. 2020;412:3961–5. 32300841 10.1007/s00216-020-02526-7 PMC7320032

[111] LabFolder. https://www.labfolder.com/. Accessed 7 June 2023.

[112] eLabFTW. https://www.elabftw.net/. Accessed 7 June 2023.

[113] Heidelberg elabFTW. https://www.sfb1158.de/. Accessed 7 June 2023.

[114] Catalyst Neuro. https://www.catalystneuro.com/. Accessed 7 June 2023.

[115] Catalyst Neuro Heidelberg Metadata-gui. https://github.com/catalystneuro/heidelberg-metadata-gui/. Accessed 7 June 2023.

[116] Tauffer L, Vaz V, Dichter B. SFB1158 Metadata GUI. 2022;

[117] Neuralynx. https://neuralynx.com/. Accessed 7 June 2023.

[118] SpikeGLX. https://github.com/billkarsh/SpikeGLX/. Accessed 7 June 2023.

[119] Siegle JH, Lopez AC, Patel YA et al. Open Ephys: an open-source, plugin-based platform for multichannel electrophysiology. J Neural Eng. 2017;14:045003. 28169219 10.1088/1741-2552/aa5eea

[120] Teeters Jeffery L, Godfrey K, Young R et al. Neurodata Without Borders: creating a common data format for neurophysiology. Neuron. 2015;88:629–34. 26590340 10.1016/j.neuron.2015.10.025

[121] SpikeInterface. https://spikeinterface.readthedocs.io/en/latest/. Accessed 7 June 2023.

[122] Buccino A, Hurwitz C, Garcia S et al. SpikeInterface, a unified framework for spike sorting. eLife. 2020;9:e61834. 33170122 10.7554/eLife.61834 PMC7704107

[123] Manz T, Gold I, Patterson NH et al. Viv: multiscale visualization of high-resolution multiplexed bioimaging data on the web. Nat Methods. 2022;19:515–6. 35545714 10.1038/s41592-022-01482-7 PMC9637380

[124] Schindelin J, Arganda-Carreras I, Frise E et al. Fiji: an open-source platform for biological-image analysis. Nat Methods. 2012;9:676–82. 22743772 10.1038/nmeth.2019 PMC3855844

[125] Fiji. https://imagej.net/software/fiji/. Accessed 7 June 2023.

[126] Imagej Bio-Formats. https://imagej.net/formats/bio-formats/. Accessed 7 June 2023.

[127] Sarkans U, Chiu W, Collinson L, et al. REMBI: recommended Metadata for Biological Images-enabling reuse of microscopy data in biology. Nat Methods. 2021;18:1418–22. 34021280 10.1038/s41592-021-01166-8 PMC8606015

[128] Moore J, Allan C, Besson S et al. OME-NGFF: a next-generation file format for expanding bioimaging data-access strategies. Nat Methods. 2021;18:1496–8. 34845388 10.1038/s41592-021-01326-w PMC8648559

[129] Bourget MH, Kamentsky L, Ghosh SS et al. Microscopy-BIDS: an extension to the brain imaging data structure for microscopy data. Front Neurosci. 2022;16:871228. 35516811 10.3389/fnins.2022.871228 PMC9063519

[130] ZIPP: The Center for Innovative Psychiatric and Psychotherapeutic Research. https://www.zi-mannheim.de/en/research/zipp-e.html/. Accessed 7 June 2023.

[131] Flor H, Rudy TE, Birbaumer N et al. Zur Anwendbarkeit des West Haven-Yale Multidimensional Pain Inventory im deutschen Sprachraum: daten zur Reliabilitat und Validitat des MPI-D [The Applicability of the West Haven-Yale Multidimensional Pain Inventory in German speaking countries: data on the reliability and validity of the MPI-D]. Der Schmerz. 1990;4:82–7. 18415223 10.1007/BF02527839

[132] Herrmann C, Buss U, Snait R. Hospital Anxiety and Depression Scale–Deutsche Version: ein Fragebogen zur Erfasung von Angst une Depressivitat in der somatischen Medizin [HADS-D—Hospital Anxiety and Depression Scale—German Version: A Questionnaire to Assess Anxiety and Depression in Somatic Medicine]. Bern, Germany: Huber; 1995.;

[133] Gorgolewski KJ, Alfaro-Almagro F, Auer T, et al. BIDS apps: improving ease of use, accessibility, and reproducibility of neuroimaging data analysis methods. PLoS Comput Biol. 2017;13:e1005209. 28278228 10.1371/journal.pcbi.1005209 PMC5363996

[134] dcm2nii. https://www.nitrc.org/projects/dcm2nii/. Accessed 7 June 2023.

[135] FreeSurfer. https://surfer.nmr.mgh.harvard.edu/. Accessed 7 June 2023.

[136] FMRIB Software Library. https://fsl.fmrib.ox.ac.uk/. Accessed 7 June 2023.

[137] fMRIPrep: A Robust Preprocessing Pipeline for fMRI Data. https://fmriprep.org/. Accessed 7 June 2023. 10.1038/s41592-018-0235-4 PMC6319393 30532080

[138] QSIprep: preprocessing and analysis of q-space images. https://qsiprep.readthedocs.io/en/latest/#qsiprep-preprocessing-and-analysis-of-q-space-images/. Accessed 7 June 2023.

[139] Heidelberg Pain Consortium SFB1158_MRHuman. https://github.com/SFB1158RDM/SFB1158_MRHuman/. Accessed 7 June 2023.

[140] BIDS-Validator. https://github.com/INCF/bids-validator/. Accessed 7 June 2023.

[141] Online BIDS Validator. https://bids-standard.github.io/bids-validator/. Accessed 7 June 2023.

[142] International Brain Laboratory, Aguillon-Rodriguez V, Angelaki D et al. Standardized and reproducible measurement of decision-making in mice. Elife. 2021;10:e63711. 34011433 10.7554/eLife.63711 PMC8137147

[143] Maggi S, Garbugino L, Heise I, et al. A cross-laboratory investigation of timing endophenotypes in mouse behavior. Timing Time Percept. 2014;2 1:35–50.

[144] Mandillo S, Tucci V, Holter SM et al. Reliability, robustness, and reproducibility in mouse behavioral phenotyping: a cross-laboratory study. Physiol Genomics. 2008;34:243–55. 18505770 10.1152/physiolgenomics.90207.2008 PMC2519962

[145] Robinson L, Spruijt B, Riedel G. Between and within laboratory reliability of mouse behaviour recorded in home-cage and open-field. J Neurosci Methods. 2018;300:10–19. 29233658 10.1016/j.jneumeth.2017.11.019

[146] van der Naald M, Chamuleau SAJ, Menon JML et al. Preregistration of animal research protocols: development and 3-year overview of preclinicaltrials.eu. BMJ Open Sci. 2022;6:e100259. 10.1136/bmjos-2021-100259 PMC8928250 35372701

[147] EBRAINS. https://ebrains.eu/. Accessed 7 June 2023.

[148] NFDI4BIOIMAGE—a consortium in the National Research Data. https://nfdi4bioimage.de/. Accessed 7 June 2023.

[149] heiDATA: an institutional repository for Open Research Data from Heidelberg University. https://heidata.uni-heidelberg.de/. Accessed 7 June 2023.

[150] King G. An introduction to the dataverse network as an infrastructure for data sharing. Sociol Methods Res. 2007;36:173–99.

[151] Brase J. DataCite - A Global Registration Agency for Research Data. 2009 Fourth International Conference on Cooperation and Promotion of Information Resources in Science and Technology. pp. 257–261.. Beijing, China, 2009.

[152] Heidelberg Pain Consortium heiDATA. https://heidata.uni-heidelberg.de/dataverse/data-sfb1158/. Accessed 7 June 2023.

[153] heiARCHIVE: heidelberg Archives. https://heiarchive.uni-heidelberg.de/de/node/1/. Accessed 7 June 2023.

[154] Heidelberg Pain Consortium: heiBOOKS. https://books.ub.uni-heidelberg.de/heibooks Accessed 7 June 2023.

[155] Kanza S, Knight NJ. Behind every great research project is great data management. BMC Res Notes. 2022;15:20. 35063017 10.1186/s13104-022-05908-5 PMC8781028

[156] Federer LM, Lu YL, Joubert DJ et al. Biomedical data sharing and reuse: attitudes and practices of clinical and scientific research staff. PLoS One. 2015;10:e0129506. 26107811 10.1371/journal.pone.0129506 PMC4481309

[157] Pasquetto IV, Randles BM, Borgman CL. On the reuse of scientific data. Data Sci J. 2017;16:8.

[158] Weichbrod RH, Thompson GA, Norton JN. Management of Animal Care and Use Programs in Research, Education, and Testing. Boca Raton, FL: CRC Press/Taylor & Francis; 2018. 29787045

[159] Jin IS, Yoon MS, Park C-W et al. Replacement techniques to reduce animal experiments in drug and nanoparticle development. J Pharm Investig. 2020;50:327–35.

[160] Manciocco A, Chiarotti F, Vitale A et al. The application of Russell and Burch 3R principle in rodent models of neurodegenerative disease: the case of Parkinson's disease. Neurosci Biobehav Rev. 2009;33:18–32. 18771685 10.1016/j.neubiorev.2008.08.002

[161] Tremoleda JL, Sosabowski J. Imaging technologies and basic considerations for welfare of laboratory rodents. Lab Anim. 2015;44:97–105. 10.1038/laban.665 25693107

[162] DFG Guidelines on the Handling of Research Data. https://www.dfg.de/download/pdf/foerderung/grundlagen_dfg_foerderung/forschungsdaten/guidelines_research_data.pdf/. Accessed 7 June 2023.

[163] Peng G, Privette JL, Tilmes C et al. A conceptual enterprise framework for managing scientific data stewardship. Data Sci J. 2018;17:15. 33101400 10.5334/dsj-2018-015 PMC7580807

[164] INCF International Neuroinformatics Coordinating Facility. https://www.incf.org/. Accessed 7 June 2023.

[165] Abrams MB, Bjaalie JG, Das S et al. A standards organization for open and FAIR neuroscience: the International Neuroinformatics Coordinating Facility. Neuroinformatics. 2022;20:25–36. 33506383 10.1007/s12021-020-09509-0 PMC9036053

[166] INCF Standards and Best Practices portfolio. https://www.incf.org/resources/sbps/. Accessed 7 June 2023.

[167] FAIRsharing: a curated, informative and educational resource on data and metadata standards, inter-related to databases and data policies. https://fairsharing.org/. Accessed 7 June 2023.

[168] Sansone S-A, McQuilton P, Rocca-Serra P, et al. FAIRsharing as a community approach to standards, repositories and policies. Nat Biotechnol. 2019;37:358–67. 30940948 10.1038/s41587-019-0080-8 PMC6785156

[169] UK Digital Curation Centre. https://www.dcc.ac.uk/. Accessed 7 June 2023.

[170] Research Data Alliance. https://www.rd-alliance.org/. Accessed 7 June 2023.

[171] European Open Science Cloud. https://eosc-portal.eu/. Accessed 7 June 2023.

[172] European Open Science Cloud. Nat Genet. 2016;48:821. 27463394 10.1038/ng.3642

[173] Arendt T. 2021. Concepts and services for the homogenization and management of file structures in collaborative neuroscientific projects [Data set]. Zenodo. https://zenodo.org/record/4818208.

[174] Harris PA, Taylor R, Thielke R et al. Research electronic data capture (REDCap)—a metadata-driven methodology and workflow process for providing translational research informatics support. J Biomed Inform. 2009;42:377–81. 18929686 10.1016/j.jbi.2008.08.010 PMC2700030

[175] Marcus DS, Olsen TR, Ramaratnam M et al. The Extensible Neuroimaging Archive Toolkit: an informatics platform for managing, exploring, and sharing neuroimaging data. Neuroinformatics. 2007;5:11–33. 17426351 10.1385/ni:5:1:11

[176] Das S, Zijdenbos AP, Harlap J et al. LORIS: a web-based data management system for multi-center studies. Front Neuroinform. 2011;5:37. 22319489 10.3389/fninf.2011.00037 PMC3262165

[177] Germany-wide ParaReg registry. www.parareg.de/. Accessed 7 June 2023.

[178] Rupp R, Jersch P, Schuld C et al. Das deutschlandweite, webbasierte ParaReg-Register zur lebenslangen Dokumentation von Querschnittgelähmten—Datenmodell, rechtlich-ethische Voraussetzungen und technische Implementierung. Gesundheitswesen. 2021;83:S18–26. 34731889 10.1055/a-1538-6537

[179] Poline JB, Kennedy DN, Sommer FT et al. Is neuroscience FAIR? A call for collaborative standardisation of neuroscience data. Neuroinformatics. 2022;20:507–12. 35061216 10.1007/s12021-021-09557-0 PMC9300762

[180] Jollans L, Boyle R, Artiges E et al. Quantifying performance of machine learning methods for neuroimaging data. Neuroimage. 2019;199:351–65. 31173905 10.1016/j.neuroimage.2019.05.082 PMC6688909

[181] Stanford SC. The Open Field Test: reinventing the wheel. J Psychopharmacol. 2007;21:134–5. 17329288 10.1177/0269881107073199

[182] Uslu ZSA. Recent advancements in behavioral testing in rodents. MethodsX. 2021;8:101536. 35004195 10.1016/j.mex.2021.101536 PMC8720839

[183] Kuo JY, Denman AJ, Beacher NJ et al. Using deep learning to study emotional behavior in rodent models. Front Behav Neurosci. 2022;16:1044492. 36483523 10.3389/fnbeh.2022.1044492 PMC9722968

[184] van Dam EA, Noldus LPJJ, van Gerven MAJ. Deep learning improves automated rodent behavior recognition within a specific experimental setup. J Neurosci Methods. 2020;332:108536. 31794777 10.1016/j.jneumeth.2019.108536

[185] Spink AJ, Tegelenbosch RA, Buma MO et al. The EthoVision video tracking system—a tool for behavioral phenotyping of transgenic mice. Physiol Behav. 2001;73:731–44. 11566207 10.1016/s0031-9384(01)00530-3

[186] Lopes G, Monteiro P. New open-source tools: using Bonsai for behavioral tracking and closed-loop experiments. Front Behav Neurosci. 2021;15:647640. 33867952 10.3389/fnbeh.2021.647640 PMC8044343

[187] ANY-maze. https://sandiegoinstruments.com/product/any-maze/. Accessed 7 June 2023.

[188] Peirce JW. Generating stimuli for neuroscience using PsychoPy. Front Neuroinform. 2008;2:10. 19198666 10.3389/neuro.11.010.2008 PMC2636899

[189] Mitteer DR, Greer BD. Using GraphPad Prism's heat maps for efficient, fine-grained analyses of single-case data. Behav Anal Pract. 2022;15:505–14. 35692516 10.1007/s40617-021-00664-7 PMC9120324

[190] Mathis A, Mamidanna P, Cury KM et al. DeepLabCut: markerless pose estimation of user-defined body parts with deep learning. Nat Neurosci. 2018;21:1281–9. 30127430 10.1038/s41593-018-0209-y

[191] Sturman O, von Ziegler L, Schläppi C et al. Deep learning-based behavioral analysis reaches human accuracy and is capable of outperforming commercial solutions. Neuropsychopharmacology. 2020;45:1942–52. 32711402 10.1038/s41386-020-0776-y PMC7608249

[192] Mathis MW, Mathis A. Deep learning tools for the measurement of animal behavior in neuroscience. Curr Opin Neurobiol. 2020;60:1–11. 31791006 10.1016/j.conb.2019.10.008

[193] Berman GJ. Measuring behavior across scales. BMC Biol. 2018;16:23. 29475451 10.1186/s12915-018-0494-7 PMC5824583

[194] INCF Working Group on Standardized Data. https://www.incf.org/sig/incf-working-group-standardized-data/. Accessed 7 June 2023.

[195] NIX: neuroscience information exchange format. http://g-node.github.io/nix/. Accessed 7 June 2023.

[196] Martone M, Gerkin R, Moucek R. NIX—neuroscience information exchange format [version 1; not peer reviewed]. F1000Research. 2020;9:358.

[197] Stoewer A, Kellner C, Benda J, et al. File format and library for neuroscience data and metadata. Front Neuroinform. Conference Abstract: Neuroinformatics. 2014. doi: 10.3389/conf.fninf.2014.18.00027.

[198] NWB: Neurodata Without Borders. https://www.nwb.org/. Accessed 7 June 2023.

[199] Rübel O, Tritt A, Ly R et al. The Neurodata Without Borders ecosystem for neurophysiological data science. eLife. 2022;11:e78362. 36193886 10.7554/eLife.78362 PMC9531949

[200] DataLad. https://www.datalad.org/. Accessed 7 June 2023.

[201] Halchenko Y, Meyer K, Poldrack B et al. DataLad: distributed system for joint management of code, data, and their relationship. JOSS. 2021;6:3262. 39469147 10.21105/joss.03262 PMC11514317

[202] GIN: modern Research Data Management for Neuroscience. https://gin.g-node.org/. Accessed 7 June 2023.

[203] CEDAR. https://metadatacenter.org/. Accessed 7 June 2023.

[204] NIDM . http://nidm.nidash.org/. Accessed 7 June 2023.

[205] Sprenger J, Zehl L, Pick J et al. odMLtables: a user-friendly approach for managing metadata of neurophysiological experiments. Front Neuroinform. 2019;13:1–17. 31611781 10.3389/fninf.2019.00062 PMC6776611

[206] Garcia S, Guarino D, Jaillet F, et al. Neo: an object model for handling electrophysiology data in multiple formats. Front Neuroinform. 2014;8:1–10. 24600386 10.3389/fninf.2014.00010 PMC3930095

[207] Elephant. https://python-elephant.org/. Accessed 7 June 2023.

[208] FieldTrip. https://www.fieldtriptoolbox.org/. Accessed 7 June 2023.

[209] Oostenveld R, Fries P, Maris E, et al. FieldTrip: open source software for advanced analysis of MEG, EEG, and invasive electrophysiological data. Comput Intell Neurosci. 2011;2011:1. 21253357 10.1155/2011/156869 PMC3021840

[210] Davison AP, Brüderle D, Eppler J et al. PyNN: a common interface for neuronal network simulators. Front Neuroinform. 2008;2:11. 19194529 10.3389/neuro.11.011.2008 PMC2634533

[211] NWB: conversion Tools. https://github.com/catalystneuro/nwb-conversion-tools/. Accessed 7 June 2023.

[212] BIDS for standardizing animal electrophysiology data. https://nfdi-neuro.de/wp-content/uploads/2021/07/Sprenger_BIDS_Extension_032.pdf/. Accessed 7 June 2023.

[213] DMPOnline. https://dmponline.dcc.ac.uk/. Accessed 7 June 2023.

[214] RDMO. https://rdmorganiser.github.io/. Accessed 7 June 2023.

[215] Bryant M, Blanke T, Hedges M et al. Open Source Historical OCR: The OCRopodium Project. Berlin, Germany: Springer Berlin Heidelberg; 2010.

[216] Donnelly M, Jones S, Pattenden-Fail JW. DMP Online: A Demonstration of the Digital Curation Centre's Web-Based Tool for Creating, Maintaining and Exporting Data Management Plans. Berlin, Germany: Springer Berlin Heidelberg;2010.

[217] Public DMPs. https://dmponline.dcc.ac.uk/public_plans/. Accessed 7 June 2023.

[218] Open Science Framework. https://osf.io/. Accessed 7 June 2023.

[219] Open Science Grid. https://opensciencegrid.org/. Accessed 7 June 2023.

[220] bwVISU. https://www.bwvisu.de/. Accessed 7 June 2023.

[221] Schmidt U, Weigert M, Broaddus C et al. Cell detection with star-convex polygons. In: Frangi AF, Schnabel JA, Davatzikos, et al., eds. Medical Image Computing and Computer Assisted Intervention – MICCAI 2018 : 21st International Conference, Granada, Spain, September 16-20, 2018, Proceedings, Part II. Cham, Switzerland: Springer International Publishing; 2018:265–73.

[222] Krull A, Buchholz T-O, Jug F. Noise2Void—learning denoising from single noisy images. In: 2019 IEEE/CVF Conference on Computer Vision and Pattern Recognition (CVPR). Long Beach, CA, USA: IEEE Computer Society; 2019:2124–32.

[223] Stringer C, Wang T, Michaelos M et al. Cellpose: a generalist algorithm for cellular segmentation. Nat Methods. 2021;18:100–6. 33318659 10.1038/s41592-020-01018-x

[224] Elektronn3. https://github.com/ELEKTRONN/elektronn3/. Accessed 7 June 2023.

[225] FAIRsharing. https://fairsharing.org/. Accessed 7 June 2023.

[226] r3data: registry of research data repositories. https://www.re3data.org/. Accessed 7 June 2023.

[227] Zenodo. https://zenodo.org/. Accessed 7 June 2023.

[228] OpenPain. http://www.openpain.org/. Accessed 7 June 2023.

[229] Pain and Interoception Imaging Network (PAIN) repository. https://www.painrepository.org/. Accessed 7 June 2023.

[230] Labus JS, Naliboff B, Kilpatrick L et al. Pain and Interoception Imaging Network (PAIN): a multimodal, multisite, brain-imaging repository for chronic somatic and visceral pain disorders. Neuroimage. 2016;124:1232–7. 25902408 10.1016/j.neuroimage.2015.04.018 PMC4627849

[231] EMBL-EBI BioImage Archive (BIA). https://www.ebi.ac.uk/bioimage-archive/. Accessed 7 June 2023.

[232] Poldrack RA, Barch DM, Mitchell JP et al. Toward open sharing of task-based fMRI data: the OpenfMRI project. Front Neuroinform. 2013;7:12. 23847528 10.3389/fninf.2013.00012 PMC3703526

[233] OpenfMRI Legacy. https://legacy.openfmri.org/. Accessed Accessed 7 June 2023.

[234] OpenNeuro Project. https://openneuro.org/. Accessed Accessed 7 June 2023.

[235] Markiewicz CJ, Gorgolewski KJ, Feingold F et al. The OpenNeuro resource for sharing of neuroscience data. Elife. 2021;10:e71774. 34658334 10.7554/eLife.71774 PMC8550750

[236] BrainLife. https://brainlife.io/. Accessed 7 June 2023.

[237] Neurophysiology Data Integration (DANDI). https://registry.opendata.aws/dandiarchive/. Accessed 7 June 2023.

[238] Alam S, Bartolome J, Bassini S et al. Fenix: distributed e-infrastructure services for EBRAINS. In: Amunts K, Grandinetti L, Lippert T, et al., eds. Brain-Inspired Computing. Cham, Switzerland: Springer International Publishing; 2021, 81–9.

[239] Dillen M, Groom Q, Agosti D et al. An archive and publishing repository: a tale of two herbarium specimen pilot projects. BISS. 2019;3:1–2.

[240] Figshare. https://figshare.com/. Accessed 7 June 2023.

[241] Hahnel M. Referencing: the reuse factor. Nature. 2013;502:298. 24137833 10.1038/502298a

[242] EMBL SourceData SmartFigure. https://sourcedata.embo.org/ Accessed 7 June 2023;

[243] Gomez-Diaz T, Recio T. Research software vs. research data II: protocols for research data dissemination and evaluation in the open science context. F1000Res. 2022;11:117. 36483317 10.12688/f1000research.78459.1 PMC9706143

[244] Wallace CT, St Croix CM, Watkins SC. Data management and archiving in a large microscopy-and-imaging, multi-user facility: problems and solutions. Mol Reprod Dev. 2015;82:630–4. 26284826 10.1002/mrd.22538 PMC4878693

[245] Collaborative Research Centers (CRC). https://www.dfg.de/foerderung/programme/koordinierte_programme/sfb/. Accessed 7 June 2023.

[246] Heidelberg Pain Consortium. https://www.sfb1158.de/. Accessed 7 June 2023.

[247] SFB 1158 : From nociception to chronic pain: structure-function properties of neural pathways and their reorganization. https://gepris.dfg.de/gepris/projekt/255156212?context=projekt&task=showDetail&id=255156212/. Accessed 7 June 2023.

[248] Heidelberg Pain Consortium: central administration project (Z01). https://gepris.dfg.de/gepris/projekt/278997686/. Accessed 7 June 2023.

[249] Recommendations for the administration of research data at Heidelberg University. https://www.uni-heidelberg.de/de/universitaet/das-profil-der-universitaet-heidelberg/gute-wissenschaftliche-praxis/research-data-policy/. Accessed 7 June 2023.

[250] DFG guidelines for Handling of Research Data. https://www.dfg.de/en/research_funding/principles_dfg_funding/research_data/. Accessed 7 June 2023.

[251] Mittal D, Mease R, Kuner T et al. Supporting data for “Data Management Strategy for a Collaborative Research Centre” GigaScience Database. 2023. http://gigadb.org/dataset/datasetManagement/id/2642. 10.1093/gigascience/giad049 PMC10318494 37401720

