# Supplementary material for: Data management strategy for a collaborative research center
Source: Gigascience. 2023 Jul 4;12:giad049. doi: 10.1093/gigascience/giad049 (PMC10318494; doi:10.1093/gigascience/giad049)
Supplement: giad049_GIGA-D-22-00262_Original_Submission [file giad049_giga-d-22-00262_original_submission.pdf]

GigaScience

Data management strategy for a Collaborative Research Centre

--Manuscript Draft--

**Manuscript Number:**	GIGA-D-22-00262
**Full Title:**	Data management strategy for a Collaborative Research Centre
**Article Type:**	Review
**Funding Information:**	Deutsche Forschungsgemeinschaft (SFB1158/Z Project) — Not applicable
**Abstract:**	The importance of effective research data management (RDM) strategies to support the generation of findable, accessible, interoperable, and reusable (FAIR) neuroscience data grows with each advance in data acquisition techniques and research methods. In order to maximize the impact of diverse research strategies, multi-disciplinary, large-scale neuroscience research consortia face a number of unsolved challenges in RDM. While open science principles are largely accepted, it is practically difficult for researchers to prioritize RDM over other pressing demands. Implementation of a coherent, executable RDM plan for consortia spanning animal, human, and clinical studies is becoming more and more challenging.<br>Here, we present a RDM strategy implemented for the Heidelberg collaborative research consortium (https://www.sfb1158.de/). Our consortium combines basic and clinical research in diverse populations (animals and humans) and produces highly heterogeneous and multimodal research data (e.g., neurophysiology, neuroimaging, genetics, behavior).<br>We present a concrete strategy for initiating early-stage RDM and FAIR data generation for large-scale collaborative research consortia, with a focus on sustainable solutions that incentivize incremental RDM while respecting research-specific requirements.
**Corresponding Author:**	Jamila Andoh<br>Central Institute of Mental Health: Zentralinstitut fur Seelische Gesundheit<br>Mannheim, GERMANY
**Corresponding Author Secondary Information:**	
**Corresponding Author's Institution:**	Central Institute of Mental Health: Zentralinstitut fur Seelische Gesundheit
**Corresponding Author's Secondary Institution:**	
**First Author:**	Deepti Mittal
**First Author Secondary Information:**	
**Order of Authors:**	Deepti Mittal
	Rebecca Mease
	Thomas Kuner
	Herta Flor
	Rohini Kuner
	Jamila Andoh
**Order of Authors Secondary Information:**	
**Additional Information:**	
**Question**	**Response**
Are you submitting this manuscript to a special series or article collection?	No
**Experimental design and statistics**	Yes

Powered by Editorial Manager® and ProduXion Manager® from Aries Systems Corporation

Full details of the experimental design and statistical methods used should be given in the Methods section, as detailed in our Minimum Standards Reporting Checklist. Information essential to interpreting the data presented should be made available in the figure legends.<br><br>Have you included all the information requested in your manuscript?	
**Resources**<br><br>A description of all resources used, including antibodies, cell lines, animals and software tools, with enough information to allow them to be uniquely identified, should be included in the Methods section. Authors are strongly encouraged to cite Research Resource Identifiers (RRIDs) for antibodies, model organisms and tools, where possible.<br><br>Have you included the information requested as detailed in our Minimum Standards Reporting Checklist?	Yes
**Availability of data and materials**<br><br>All datasets and code on which the conclusions of the paper rely must be either included in your submission or deposited in publicly available repositories (where available and ethically appropriate), referencing such data using a unique identifier in the references and in the "Availability of Data and Materials" section of your manuscript.<br><br>Have you have met the above requirement as detailed in our Minimum Standards Reporting Checklist?	Yes

Powered by Editorial Manager® and ProduXion Manager® from Aries Systems Corporation

Manuscript Click here to access/download;Manuscript;Mittal_ms.pdf

Data management strategy for a Collaborative Research Centre

Deepti Mittal[1*], Rebecca Mease[2], Thomas Kuner[3], Herta Flor[4], Rohini Kuner[1], Jamila Andoh[5*]
and the SFB1158 Consortium

[1] Institute of Pharmacology, Heidelberg University, Heidelberg, Germany

[2] Institute of Physiology and Pathophysiology, Heidelberg University, Heidelberg, Germany

[3] Institute for Anatomy and Cell Biology, Heidelberg University, Mannheim, Germany

[4] Department of Cognitive and Clinical Neuroscience, Central Institute of Mental Health, Medical Faculty Mannheim, Heidelberg University, Mannheim, Germany

[5] Department of Psychiatry and Psychotherapy, Central Institute of Mental Health, Medical Faculty Mannheim, Heidelberg University, Mannheim, Germany

*Corresponding authors:
Deepti Mittal: deepti.mittal@pharma.uni-heidelberg.de, Tel: +49 (0) 6221 / 541 6600
Or Jamila Andoh: jamila.andoh@zi-mannheim.de, Tel: +49 (0) 621 / 1703 6506

ABSTRACT

The importance of effective research data management (RDM) strategies to support the generation of findable, accessible, interoperable, and reusable (FAIR) neuroscience data grows with each advance in data acquisition techniques and research methods. In order to maximize the impact of diverse research strategies, multi-disciplinary, large-scale neuroscience research consortia face a number of unsolved challenges in RDM. While open science principles are largely accepted, it is practically difficult for researchers to prioritize RDM over other pressing demands. Implementation of a coherent, executable RDM plan for consortia spanning animal, human, and clinical studies is becoming more and more challenging.

Here, we present a RDM strategy implemented for the Heidelberg collaborative research consortium (https://www.sfb1158.de/). Our consortium combines basic and clinical research in diverse populations (animals and humans) and produces highly heterogeneous and multimodal research data (e.g., neurophysiology, neuroimaging, genetics, behavior).

We present a concrete strategy for initiating early-stage RDM and FAIR data generation for large-scale collaborative research consortia, with a focus on sustainable solutions that incentivize incremental RDM while respecting research-specific requirements.

INTRODUCTION

Extensive efforts have recently been made to promote the reproducibility, replicability, and transparency of scientific research. The evolution of open access publishing, open-source data repositories, and software applications has transformed the work of researchers in various fields. As research became more sophisticated, these developments were inevitable, and large-scale multidisciplinary projects were developed to promote collaborative work. Research institutions are more and more involved in interdisciplinary collaborative research, which frequently generates a significant number of diverse datasets. Such collaborative developments pose new challenges for research data management (RDM), specifically in terms of data harmonization and data sharing.

Research in the field of neuroscience increasingly encompasses a variety of fields, including biophysics, molecular biology, medicine, cognitive neuroscience, psychology, and ethology. Neuroscience datasets are constantly growing as a result of scientific advances in acquisition systems that produce large-scale multimodal datasets [1-5]. Neuroscientific datasets necessitate robust storage and computing resources. Furthermore, integration of neuroscientific datasets and data sharing is one of the greatest obstacles in a large-scale consortium combining multi-modal and multi-site studies, which becomes more challenging if handled as an afterthought. This has a direct effect on research collaborations and the publishing process [6].

To support researchers with good data management practice, the FAIR data principles (Findable, Accessible, Interoperable, and Reproducible) were published in 2016 in the journal Scientific Data [7, 8]. These are guidelines for handling data management and stewardship issues. In addition, they raise significant concerns regarding the repeatability of research findings and the emergence of quality public knowledge through individual research projects. While funding agencies and research organizations are increasingly advocating open science and data sharing, it is rarely as simple as "sharing the data" in large consortia with cooperation projects involving data from multiple populations. Individual scientists might also find it challenging to prioritize FAIR procedures amidst competing research needs. In practice, applying FAIR standards involves enormous constraints on researchers, many of whom are under immense time pressure to deliver outputs and may lack practical or conceptual RDM expertise. Implementing effective data management strategies and ethical rules for the reuse and sharing of high-quality data reduces redundant research, optimizes public research funding, and reduces animal use. A clear set of regulations and guidelines must be established before sharing human data gathered from clinical or non-clinical populations. Specific rules addressing privacy issues, established processes for data protection, data use and reuse, and the preservation of sensitive data are required. It is essential to make data accessible and

understandable to remote (or future) collaborators in order to maximize the potential of existing algorithms and tools and accelerate the creation of new ones. While the majority of collaborative research consortiums collect a wide variety of multidimensional datasets, the majority of these datasets are typically inadequate for modern research methods and infrastructure. To overcome these obstacles, experimentalists, data managers, and computer scientists must work in a close, strategic partnership.

We assembled this report to highlight the strategies and resources that facilitate RDM in our collaborative neuroscience research consortium, comprising independent, multidisciplinary research groups with a common goal-oriented research. In this report, we discuss the importance of incremental data management as a path to making data FAIR early on in the scientific process. This report discusses our experience in developing and implementing a data management strategy and offers concrete solutions to promote multidisciplinary collaborative research and open science objectives.

The Heidelberg Pain Consortium (https://www.sfb1158.de/) is a collaborative research center (SFB 1158) composed of 44 principal investigators in Germany investigating the neurological basis of pain. Since 2015, the German Research Foundation (DFG) has supported SFB1158 (https://gepris.dfg.de/gepris/projekt/255156212?context=projekt&task=showDetail&id=255156212). In June 2019, SFB1158 was successfully renewed and got funding for another four years, 2019–2023, under project number 255156212 from DFG. SFB 1158 has many national (e.g., the University of Heidelberg, the Central Institute for Mental Health (ZI), European Molecular Laboratory, and German Cancer Research Center) and international collaborations (Institutions located in the United States, Canada, England, and France).

The broad scientific goal is to understand the mechanisms of pain and pain chronicity in order to identify causal links and possible therapeutic interventions. This involves a multidisciplinary team of scientists and clinicians working on 23 different projects, including basic research in animals and humans as well as clinical research (Figure 1).

These projects cover a wide range of temporal and anatomical scales, including molecules, single cells, neuronal circuits, and structural and functional brain connectomes. The research also includes acute and chronic pain animal models, with a focus on translating rodent findings to healthy humans and chronic pain patients. For this purpose, tandem projects were included to design the best strategies to facilitate forward and reverse translation of data between rodent and human conditions. The rodent part uses high-resolution fluorescence and magnetic resonance imaging, electrophysiology, optogenetics, and self-stimulation behavioral paradigms to delineate the functions of underlying circuits, while the human part uses fiber tracking approaches and daily life analyses in addition to fluorescence and magnetic resonance imaging. One of the consortium's human-mouse tandem project focuses on

structure/function reorganization in the context of central neuropathic pain caused by spinal cord injury. Ultrastructural visualization and 3D reconstruction are used to investigate structural changes in the mouse part. In the human part, structural changes in the PNS (peripheral nerves and dorsal root ganglia) are determined with advanced MRI techniques.

The consortium further aims to elucidate the structure-function basis by which the transition from acute to chronic pain is impacted by psychological, social, and environmental influences (learning, stress, early life trauma, and social support) and comorbidities (anxiety, fear, depression, addiction). SFB 1158 includes two service projects: the first one (also a tandem project) aims at establishing standard protocols, models, and ethical standards to facilitate homogeneous implementation across all human or rodent projects. The second service project (also an animal project) aims at developing simplified systems to accelerate the analysis of the translational potential of acquired research insights.

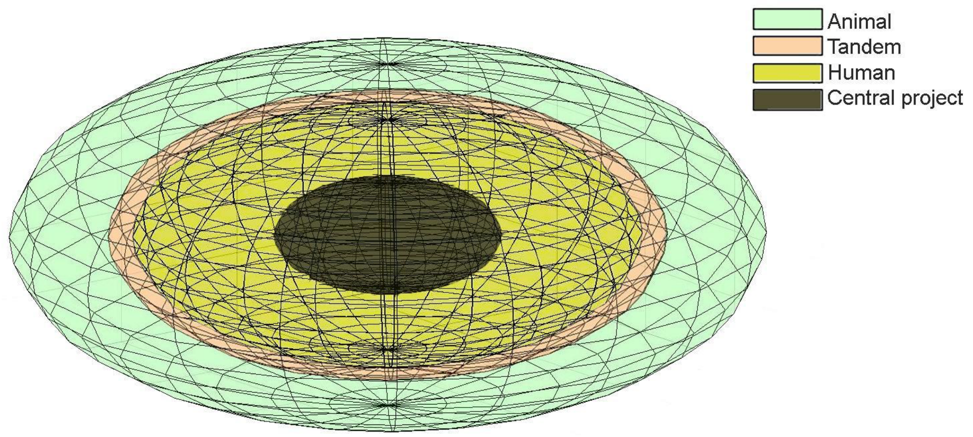

Figure 1: Overview of projects in the Heidelberg Pain Consortium (SFB 1158): a multi-disciplinary group of scientists and clinicians leading 23 separate projects, including 12 animal projects, six tandem (human-animal) projects, four human projects, and one central administrative project. The two service projects are included in the animal and tandem projects, respectively.

RESULTS

A prototype of the RDM infrastructure should be ideally built based on initial requirements at the start of the funding application. The Heidelberg Pain Consortium implemented a development strategy in central administration project (Z01) (https://gepris.dfg.de/gepris/projekt/278997686) to promote RDM as an integral part of the research process in order to maximize the impact of collaborative science. By implementing

this strategy, the consortium is able to take a systematic and standards-based approach to documenting, archiving, and sharing its research data with collaborators and the research community, with the goal of significantly accelerating scientific progress. This RDM model is expected to evolve in response to the development of new and specialized (domain-specific) technical infrastructures.

We focused on strategies that included joint efforts and cooperation between consortium members and other collaborators to develop progressively practical RDM approaches. In the second funding period (2019-2023), the SFB recruited a data manager to act as a liaison between consortium researchers as well as employees of the Competence Center for Research Data (KFD), a joint service facility of the University Library (UB) and the University Computer Center (URZ), and community organizations (e.g., Nationale Forschungsdateninfrastruktur [National Research Data Infrastructure], NFDI), to streamline access to the common research data infrastructure. The data manager is integrated as a member of the consortium, directly and continuously communicating with members of each SFB project, thus gaining first-hand knowledge about member requirements.

Heidelberg University, as an SFB host institution, offers detailed recommendations for the administration of research data (http://www.uni-heidelberg.de/universitaet/profil/researchdata/). We developed a SFB data management policy (see Supplementary Information 1) in accordance with the DFG guidelines for research data handling (https://www.dfg.de/en/research_funding/principles_dfg_funding/research_data/). In order to facilitate the long-term preservation of valuable data sets, the SFB data policy serves as recommended guidelines for individual projects on how to format their data. The policy is applicable to all researchers working in the SFB, including principal investigators (PIs), doctoral and postdoctoral researchers, and student research assistants. It also applies to any research projects carried out within the SFB as well as any data generated or used (from outside sources).

SFB 1158 offers a variety of RDM services to researchers to ensure that RDM for each project adheres to the DFG guidelines. These services include assistance with proper data documentation; integrating and supporting open data management solutions; data storage and accessibility; the development of new tools for the adoption of open data and metadata standards; the sharing of diverse data sets within the consortium and with external collaborators; and dissemination of research outputs into national and international data repositories.

The primary goal of this report is to describe our approach to implementing data management across the consortium's diverse research laboratories, as well as to make recommendations and guidelines for best practices. We specifically describe our ongoing RDM efforts, which are

divided into two sections: 1) RDM Planning Phase: Identifying common RDM procedures across consortium projects; Evaluating common data management challenges; Special RDM challenges in specific projects; 2) RDM Implementation Phase: resource allocation decisions and implementation of key resources and 3) Data Dissemination (data management continues even after the end of the funding period).

RDM Planning Phase-Assessment of data management requirements, challenges, and available resources across the consortium

This section describes the RDM planning processes that can be used effectively at the early stage (planning phase) of research projects. Our priority was to adequately characterize the consortium's needs before committing to specific resources. The implementation of an RDM strategy for a large consortium is primarily based on the various types of data generated across research projects, as well as practical methods for organizing and managing this data. We examine commonalities across projects during the initial planning phase, such as similar populations being studied and common acquisition techniques, in order to define common RDM measures. Then we describe both general and project-specific challenges we encountered while evaluating RDM requirements across multiple projects.

SFB 1158 includes research projects that typically collect a large number of diverse datasets (e.g., neurophysiology, clinical, genetic, neuroimaging, etc.) from multiple species and subjects. One of the main goals of identifying common factors was to ensure that best data management practices can be implemented across the consortium while keeping individual lab practices in mind. The collected information was used while implementing common RDM solutions for individual SFB project groups, such as identifying and targeting resources for data storage, data organization, and data sharing. In addition, we assessed the current data-management measures and processes in individual laboratories. The project-specific assessment included questions regarding the types of experimental models; data modalities and their acquisition methods and techniques; types of analysis tools and software; types of raw and intermediate file formats; workflows for pre-preprocessing and analysis of acquired data; export procedures for sharing and publication of datasets; etc., for each project. This provides us with a strong basis for high-level data requirements and further RDM task categorization. Given the large number of laboratories from various institutions participating in the consortium, as well as the increasing number of requirement changes over the course of a project, information is gathered in a variety of ways (virtual individual interviews with project PIs; discussions during online data seminars led by the SFB data manager; and personal meetings with experimentalists and PhD students). Data discussions and regular

communication with consortium members have greatly aided our assessment approach. As part of the assessment process, the PIs, or project responsible persons, were required to respond to a wide range of data management questions (see Supplementary Information 2).

The initial step was to identify the commonalities between all of the projects, such as the type of population studied (rodents, humans, or tandem), followed by the type of data modalities acquired (e.g., neurophysiology, neuroimaging, and behavior). Our consortium specifically includes 23 projects, comprising 12 animal projects, six human-animal tandem projects, four human projects, and one administrative project. Human projects include data collected from both healthy individuals and patients with various clinical conditions (e.g., chronic back pain, severe depression, diabetic neuropathy etc.) and Rodent projects utilize mouse as animal models (Figure 2 A).

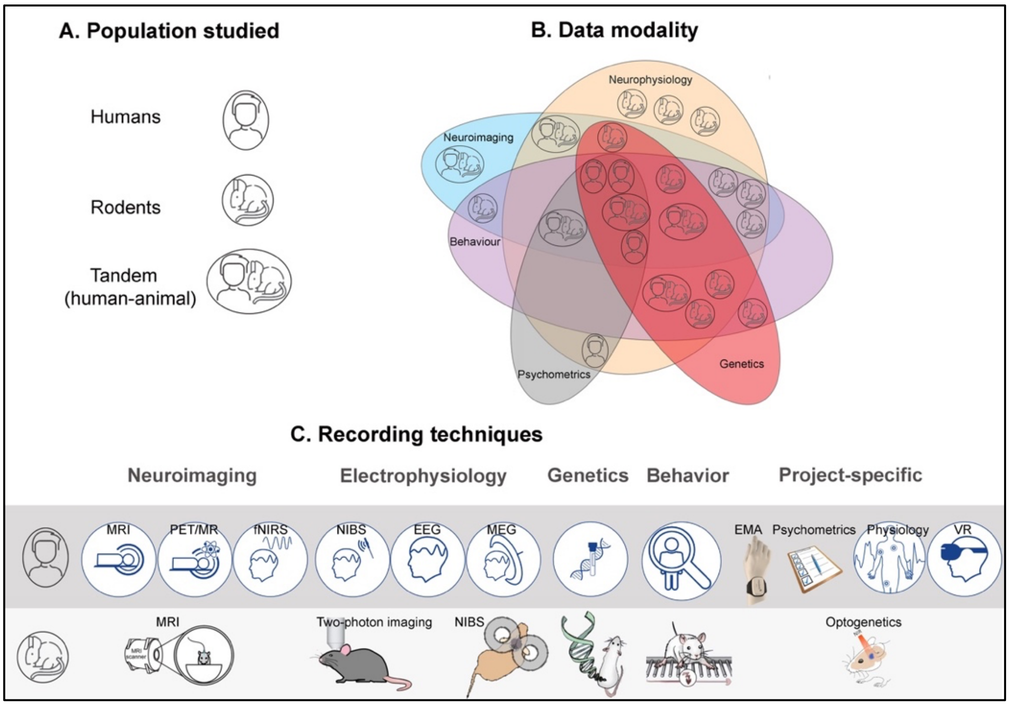

Figure 2: The Heidelberg Pain Consortium investigates various populations: humans, rodents, tandem (A) using various modalities: neuroimaging, neurophysiology, behavior, psychometrics, genetics (B). Each data modality can be recorded using different techniques (C): MRI= magnetic resonance imaging; PET/MR: positron emission tomography/magnetic resonance; fNIRS: functional near infrared spectroscopy; NIBS: non-invasive brain stimulations; EEG: electroencephalography; MEG: magnetoencephalography; Genetics; Two-photon imaging; Behavior; EMA: ecological momentary assessment; Psychometrics; Physiology; VR: virtual reality; optogenetics.

We further categorized projects into subgroups on the basis of common data modalities that were being acquired. Figure 2B depicts an overview of the various data types collected across the consortium projects. Neurophysiology data (including electrophysiology and cellular physiology) are the most frequent data category collected across all studies (i.e., 83% for animal and tandem projects and 100% for human projects). Imaging, behavioral, and genetic data are collected in similar proportions in human projects (75%), whereas psychometric data is collected by all human projects. In 41.2% of the animal projects and 83% of the tandem projects, imaging data are collected. Behavioral data (e.g., various pain models) are collected in 58% of animal projects and 66.67% of tandem projects.

The SFB projects include a wide range of methods and techniques (Figure 2C) such as electrophysiology, neuroimaging, extracellular and intracellular signals, and two-photon imaging for rodents; behavior (including stress and fear assessment in humans and rodents) and multi-omics (genetics) datasets. Rodent and human projects use comparable methods, such as magnetic resonance imaging (MRI) of both the brain and peripheral nerves; electrophysiology, including electroencephalography (EEG) and magnetoencephalography (MEG) for humans, extra- and intracellular signals and two-photon imaging for rodents; brain stimulation methods including transcranial magnetic stimulation (TMS) and transcranial electrical stimulation (tES). Additional specific methods in humans are electronic diaries (EMA), peripheral physiology (e.g., heart rate, blood pressure, sensory profiles); virtual reality; psychometrics and daily assessments of psychological methods such as ecological momentary assessment (EMA). Specific methods in rodents include optogenetics.

We gathered information on optimal storage solutions for projects, and the response was diverse as there were projects acquiring large numbers of datasets (e.g., 1 terabyte of data per month), whereas others acquired relatively smaller datasets (e.g., a few gigabytes per month). We documented that 80% of the projects were already utilizing university infrastructure for data storage, whereas many human projects utilize individual lab servers.

We then collected information about file formats utilized for collecting and preprocessing raw data from different acquisition systems and a wide range of methods. Given the complexity and diversity of experiments and the varied volume of data collected in the consortium, the acquired file formats are most often highly specific to certain data types (such as time series, e.g., voltage traces, image stacks, stimuli or behavior) or acquisition or recording device. Several projects require the development of new tools and software for migration to open data standards, resulting in the need for additional resources and support from the SFB.

Electrophysiology experiments, equipment, and analysis pipelines, in particular, are customized for each project and generate data in a variety of file formats. Data are collected using a variety of techniques and experimental designs, such as patch-clamp to tetrodes in freely moving animals and high-density silicon probe recordings. The steps for preprocessing for intracellular, juxtacellular, and extracellular techniques are frequently customized. Any measures to standardize must be compatible with existing lab analysis tools and data processing methods. Despite the availability and evolution of a number of community-developed electrophysiology meta and data standards, they have not been widely adopted.

We also collected information about the most common preprocessing and analysis software (e.g., IgorPro, ImageJ, Matlab, etc.) utilized across different projects. Our assessment also included information regarding projects using electronic lab notebooks and those using traditional handwritten notebooks. Additionally, we assessed the definition of user permission for data access; protocols for data sharing; short-and long-term storage needs; and implementation costs. This information enables us to tailor the data management solutions to the common needs of the majority of projects (see section II implementation).

Data management challenges are rapidly increasing as a result of new technology trends and rising data volumes. In this section, we highlight the data challenges our consortium encountered while planning RDM solutions. Common challenges include coherent RDM planning for diverse datasets, data and metadata documentation, data sharing and archiving, and handling sensitive data.

Challenges due to multimodal datasets: A central challenge was the variety of data types produced by multidisciplinary approaches. The projects involved balancing data from basic and clinical research as well as data from animals and humans. Diverse signals were collected at various spatial and temporal scales, such as single cell and network data, genetic, geospatial, behavioral and also patient-surveying data such as clinical assessments and psychological questionnaires. All human research projects use a multi-modal approach that combines data from two or more of the following methods: MRI: structural (anatomical and diffusion-weighted imaging) and functional: task-based and resting-state; electro-and magnetoencephalography (EEG and MEG, respectively); behavioral; psychometric; and genetics. These diverse techniques raise manageability issues within and between projects, with broad implications for data interoperability and reuse.

Using diverse and large-scale datasets offers researchers greater statistical power and the opportunity to do more robust secondary data analysis [9]. Massive amounts of high-dimensional data (large sample size, various models and conditions) bring opportunities for

scientific research. Merging multimodal datasets aggregated from multiple sources at different time points and using available technologies improves reliability and offers more insights but introduces new data management issues. There are significant challenges when working with multimodal data, such as integration of various data sources; harmonization of heterogeneous datasets; keeping corresponding timing information; achieving and maintaining good data quality (checking for missing data, duplicates); properly securing all the collected data and adhering to privacy and security regulations while enabling access to specific users. These challenges are compounded by the increasing need to share data with external collaborators. These challenges are present in most projects but are more significant for tandem projects because they require parallel implementation of experimental designs, various methodologies, and data types. In addition, animal data preprocessing guidelines are substantially less well established than those for human data, which makes direct comparisons and meta-analyses very difficult. A systematic effort to develop standardized guidelines for multi-modal data acquisition would facilitate the adoption of FAIR standards across all studies. As different complex measurements proliferate as routine parts of data collection, this problem will only increase.

Another challenge is managing metadata, especially for complex, large, multi-site, heterogeneous datasets. In an ideal world, all the metadata associated with the acquired datasets would be easily available and sufficient for data sharing. In reality, they are not (yet). The associated metadata (such as the origin and type of a sample, experimental conditions, applied measurement techniques, devices used, and calibration methods, units) are frequently missing, incomplete, or only available in fragmentary form. In cases where a laboratory produces a large dataset from a single experiment, and the collected metadata are too complex and stored in multiple files having different formats that are only read by the acquisition software or by customized codes written for internal use. Furthermore, raw data and associated metadata may be distributed in different files or separate directories depending on individual lab practice. It requires additional effort to read and extract the metadata from their original raw data files and integrate them into a single file. Interoperability between file formats can be a technical problem if the proper software does not exist anymore. Finding data that can be potentially pooled remains challenging, let alone assurance that datasets are in standardized formats for meta-analyses by third parties. Strategies for applying FAIR principles are still developing, and standard annotation systems and clear data identifiers are crucially needed.

All of the human projects in SFB 1158 are collecting data at different time points. This is occasionally essential due to time constraints among participants or to prevent volunteer fatigue. Datasets gathered over several days are typically randomized or pseudo-randomized. This is particularly the case in some human projects where longitudinal studies with repeated assessments are conducted. For such studies, the metadata should usually be provided as a set of documents available to download alongside the data itself. The metadata documentation, including the longitudinal and cross-sectional components of the study such as screening, follow-up measures, research teams who conducted the study, sample size and the age of the participant at the time of measurement, time intervals between the assessments, etc., is necessary. It is indeed important that metadata is informative about the dataset to be analyzed while following standardized ethical and quality measures. For instance, some projects investigate pain chronicity by following up pain patients over several days or years while acquiring various data (e.g., MRI) and metadata (psychological and pain questionnaires, sensory profiles). Associations between data and metadata are made to establish causal factors for chronic pain. Such studies could not be performed without sufficient and reliable documentation of metadata.

Although many advances have been made regarding organization, annotation, and description of research datasets, for example using the Brain Imaging Data Structure (BIDS, https://bids.neuroimaging.io/) for neuroimaging data standards [10] EEG-BIDS for neurophysiology data (e.g., electroencephalography), [11], or MEG-BIDS for magnetoencephalography data [12], whereas other data modalities (e.g., EMA) do not have existing standards yet.

Sensitive data: Projects involving human subject data or other sensitive data must maintain strict privacy for storage and sharing of data collected or used for research purposes [13]. Sensitive data with potentially identifying information must be anonymized or pseudonymized prior to making the data public to protect participant confidentiality. Keeping such ethical standards can be costly and time-consuming, adding a further burden to researchers [14].

Long-term preservation and sharing of sensitive data largely depends on informed consent, data reuse agreements and policies, and also the type of archiving solution or data repository being used. Each step of handling sensitive data must protect privacy and identity protection rights, often through de-identification or anonymization. There are distinct set of regulations for full anonymization versus de-identification of data [14]. It is therefore recommended to retain multiple versions of the data: one suitable for public release, and one suitable for further research but available on a highly restricted basis [15]. These considerations can lead to increased data duplication and storage needs.

There is, furthermore, a lack of efficient software to adequately segregate and maintain control over sensitive data. It is important to provide researchers working with sensitive data or samples with truly "useful" tools that do not require pre-existing, in-depth knowledge of legal and ethical requirements, or time to delve into the details. Sensitive data sharing between collaborators located in two different locations requires additional effort as data controllers need to make sure that data protection requirements are met in both the original location where the data were collected and the collaborator's location.

Furthermore, external collaborations across universities can present logistical challenges in the form of access and security entitlements; these concerns are compounded when collection of sensitive data is part of the research project, or for collaborations with researchers embedded in clinical settings. Another major obstacle to sharing confidential data with external parties is the cost involved in adopting secure data sharing platforms and a major risk of participants being identified. In this context, researchers require consistent training and education that promotes responsible research conduct and adheres to institutional and discipline-specific data management policies (risks of data disclosure, confidentiality obligations, privacy principles, and network security).

Data storage and volume: The data volume varies substantially depending on the data modality, ranging from a few megabytes (e.g., questionnaire data) to terabytes (e.g., high resolution fluorescence imaging). Projects involving large amounts of data generated from high-resolution fluorescence imaging, volume electron microscopy, electrophysiology, or fMRI can typically yield terabytes of data. Such data is often stored in dispersed locations and infrastructures in various formats (often proprietary), requiring a significant amount of time and effort to efficiently manage, utilize, and curate the data. Researchers, particularly those working with high-dimensional data, require consistent support for data storage, timely backups, and archival systems. Inefficient data storage processes can lead to data integrity failures, accessibility issues, and an increase in operational costs. Commercial cloud storage solutions are available, but they come with unreliable and slow backup and restoration services, as well as no obvious access paths or interfaces for easy migration onto analysis platforms. Assuring access to secure and optimal storage solutions that can be integrated with workflows encompassing data acquisition, intermediate analysis, and archiving is thus a major challenge. Creating backups and storing multiple copies of large volumes of datasets, the need for collaborative and parallel access by multiple people, and use across a diverse range of computational workflows all pose major challenges to storage servers.

Diversity in preprocessing and analysis approaches: In addition to storage concerns, neuroscience as a whole suffers from a lack of standardized preprocessing and analysis

workflows. Even when datasets are imported into a common file format, data decisions are frequently influenced by the immediate analysis of interest, with no guarantee of compatibility between laboratories or even between projects within a laboratory. An overarching issue we encountered was the inadequate and uneven use of available software resources, ranging from lab-customized preprocessing software to open-source software packages. Often, for neuroscience experiments, despite similarities in experimental design, researchers tend to organize and describe their data in their own way, even within the same research group. Research groups often use specific custom preprocessing pipelines that are developed internally to meet only a lab's current needs. In the analysis stage, researchers must either write custom analysis scripts or spend time and effort converting datasets into supported formats to use publicly available tools for analysis. Various preprocessing or analysis software often results in different file inputs and outputs. These lab-specific custom workflows and pipelines usually prioritize internal needs over the needs of a broader community.

Another general challenge while using lab-customized software or a single computer hardware solution (e.g., routine analysis scripts) is that they do not perform efficiently on large or complex datasets. Running high-quality analysis on large-scale datasets often necessitates high-performance computational resources. In the case of workflows including third-party tools and software, there are issues of broken dependencies because of variability in computing. Problems with reproducibility are made worse by the fact that the original analyses were done in different environments, using different operating systems, and with different versions of software.

Additionally, modern deep-learning applications used for neuroimaging data analysis require more computing power, memory, and storage resources. High-performance computing (HPC) clusters are available to provide these resources, dramatically accelerating the analysis process. However, it is challenging for non-experts to get easy access to these resources and perform scientific computing. Especially for experimentalists, there is a fundamental need to give clear and succinct documentation on how to use these resources efficiently. Applications for image data processing must have application programming interfaces (API) that may be utilized without specialized coding knowledge. It is often suggested to use comparative analysis methods and two or more software packages to obtain reliable and reproducible research results. Developing such tools (e.g., bwVisu, https://www.bwvisu.de/) requires significant customization and software development costs, an investment which may not be possible for individual research labs.

Challenges in data documentation: Data documentation presents a number of challenges, including adoption of digital systems and laboratory inventory management systems for large

consortia. Electronic laboratory notebooks play an important part in the documentation of data (such as hypotheses, methods, observations, experimental protocols, notes, etc.). Several efforts over the last several years have recognized the critical need for institutional-wide adoption and implementation of an electronic laboratory notebook [16]. However, while choosing an electronic laboratory notebook for a large-scale neuroscience consortium spanning diverse experimental protocols, the availability of clear documentation and application-centric features become an overarching issue. The initial challenge is to select an appropriate option that fits into the current laboratory standards. In addition, a usable and sustainable electronic laboratory notebook needs to be interoperable and incorporated into existing data workflows. There are obvious issues of user resistance; expensive costs involved in the implementation; secure configuration and maintenance; and the user will be ultimately responsible for managing the digital system.

There are several open-source and proprietary options available for use. Often, for proprietary options, documentation may exist in the form of vendor's specifications or may be created and maintained within the context of a global community. It might not fulfill domain-specific requirements. A main functionality that could support easy documentation is sometimes missing, and the available features are secondary and not as beneficial to users. Often, there is no automated end-to-end solution that allows users to document experiments, which in turn makes this process time-consuming and tedious to perform manually.

Data sharing and dissemination challenges: There are significant challenges in organizing datasets in a useful manner to enable sharing with collaborators. Even if a dedicated central data storage infrastructure is available, insufficient quality control measures as well as a lack of time have a direct impact on data sharing practices. Especially in small research groups or individual projects, limited funding and sustainable resources directly impact the level of data sharing and reuse. Another significant issue is motivating researchers to share data publicly. Indeed, researchers are also hesitant about openly sharing their data due to the concern of possibly not receiving credit, or fear of reducing their own chances of performing secondary studies, or not having handled data sensitivity properly, and to avoid possible criticism about data quality. Despite the fact that an increasing number of research organizations, academic journals, and large-scale projects are supporting extra efforts to build realistic data sharing techniques, this has not yet become a standard research practice [17].

Moreover, although a large number of journals are enforcing open data sharing and dataset submission to public repositories prior to manuscript submission, there is limited oversight on data sharing policies. Additionally, choosing a suitable public repository could be difficult for a number of reasons. Researchers should confirm that the repository complies with the research data regulations of their host institution before contributing datasets to open repositories.

Finding a suitable subject-specific repository for a given dataset could be challenging. The alternative is to submit the data to a general-purpose repository, but there can be issues regarding data visibility as the particular repository might not be well-recognized in its field of research. Another significant issue with submitting data to general repositories is that they do not have enough support for certain types and formats of data. Even after identifying a suitable repository, bureaucratic procedures and demands for publishing datasets in open data repositories require additional work, including converting files to the required format, compiling consent forms and contracts, removing sensitive information, and preparing documentation. Finally, maintenance funding must be taken into account because many repositories charge a fee based on the data volume. In the latter stages of the data lifecycle, these factors can hamper findability and reusability.

Data archiving: For the long-term preservation of data, researchers need permanent archiving systems, along with sufficient funds to build such archives for both internal usage and to satisfy the open data needs of journals and funding organizations. Ideally, archival systems should be developed with the user's perspective, especially in scientific settings where researchers with limited expertise in digital preservation collaborate on projects that generate a wide range of data. Researchers require assistance with best practices for archiving in addition to access to archival systems. For instance, how to remain informed about the storage, retention, and disposal of all research data, whether in an institutional or external repository, especially as good archival practice includes a scheduled review of items in long-term storage. Support may also be needed to determine that data handling complies with various regulations and/or guidelines: existing discipline-specific privacy and ethical standards; existing copyright or licensing arrangements; and publication and legal requirements. The period for which data should be preserved for research purposes or archiving should be determined by prevailing standards for the specific type of research domain, and should follow the retention policies of any applicable stakeholders (e.g., sponsoring institution, funding agency). For example, in the context of our consortium funded by the DFG, primary research data should be appropriately archived in the researcher's own institution or an appropriate nationwide infrastructure for at least 10 years (DFG Guidelines on the Handling of Research Data).

Special RDM challenges: Across the consortium, several projects presented specific challenges in RDM, either in organization and management, sheer data volume, logistics, or collaborative scale. In these cases, effective data management is integral to project success and may require custom strategies and resources. Below, we list examples representing the consortium extreme cases; we found this identification useful in determining the new developments required.

A few of the animal projects in the consortium make use of new technologies such as high-density Neuropixels probes [18]. This method can produce very large datasets (~80 GB/hour). Data storage requirements rise as a result of the significant amounts of derived data needed for intermediate processing (such as filtering and spike sorting) and stimulation and/or behavioral parameters (such as optogenetic stimulation, motion or whisker tracking, and task performance). Analysis and post-processing may often require computationally intensive algorithms and hardware acceleration to handle data that cannot be loaded into local memory [19]. Real-time processing requirements for closed-loop experiments only serve to exacerbate these issues. Important parameters initially recorded from raw data, e.g., animal arousal/anesthesia level, impedance measurements, can be discarded in derived datasets used for analysis. Complex hierarchies of derived data and multimodal datasets (e.g., accelerometer, whisker or pupil tracking, etc.) collected with different instruments compound these issues.

Some rodent projects within the consortium acquire large amounts of data collected over months [20]. For example, data acquisition using fluorescence imaging or two-photon microscopy calcium imaging (2P imaging) generates large sets of spatiotemporal imaging data (up to 100 GB/hour) and requires rigorous preprocessing steps (image denoising, motion correction, manipulation and handling of large video files, and neural activity deconvolution) using high-throughput computing [21]. The downstream processing and analysis of resulting datasets generated over the course of months is often challenging and requires complex workflows [22]. A few open-source software solutions (CalmAn, EZcalcium, TrackMate etc.) have been proposed to deal with these challenges [23, 24]. However, comparative analysis studies have revealed that the neural assemblies recovered from these datasets can vary significantly depending on the algorithm used, and that certain algorithms are more reliable and faster than others [25]. Another challenge is that many studies include synthetic or benchmarking datasets, but generating and analyzing these datasets requires complex calculations, which in turn increases the computational complexity and cost. This enables the need for more scalable and fully-automated workflows that can be run on high-performance computing clusters [20], which requires ensuring the reproducibility of studies. Existing software solutions can be used for analysis and visualization of datasets, but any adopted data and metadata filed standards must be interoperable with these tools.

Projects collecting data from both human and animal models pose several challenges, such as systematic and parallel implementation of experimental designs, techniques, and analysis tools. Data management processes to create harmonized datasets and analysis workflows while establishing clear linkages between human and animal models are difficult. In

collaborative projects involving multiple laboratories working on multiple species, the integration of data and analysis should happen systematically, not only sporadically. Apart from the sheer scale of such collaborations involving multiple research areas and the multimodal RDM issues discussed above, these tandem projects require a secure platform for data transfer between different sites (e.g., laboratories and clinics) with different security permissions and data handling standards.

The increasing number of collaborative studies may be hampered by challenges in standardizing behavioral experiments (e.g., continuous animal movement recordings, mouse trajectories) across laboratories. There is no specific data standard available for storing behavioural datasets which directly influences data sharing. It is critical to develop a common centralized database for storing methods and experimental protocols of behavioral assays, parameters (e.g., sex, age and strain of the animal, genotype, marking, testing conditions etc.), data and metadata files generated in the task (e.g., behavioral responses and compressed video and audio files), as well as a common framework that supports further analysis and visualization [26].

Consortium-wide RDM implementation phase - key resources, infrastructure, and services

RDM Communication and Exchange: A number of neuroscience-specific RDM solutions already exist, ranging from software and infrastructures for streamlining data collection and acquisition protocols, collaborative data analysis and visualization packages, to data sharing and archiving platforms. Our initial observation while implementing RDM strategies was that many researchers were not aware of the benefits of existing resources, partly due to uncertainties regarding the bureaucratic procedures, the General Data Protection Regulations (GDPR), and more often, technical requirements for easy integration of these resources into existing laboratory practices. Therefore, we put great emphasis on promoting and encouraging the use of pre-existing resources that meet the needs of our consortium or that help in a particular use case. An important aspect was to find a balanced approach which encourages an appropriate degree of integration of existing resources with realistic domain-specificity. We curated a list of both generic and neuroscience specific RDM resources both on the consortium/institutional (internal) and national and international (external) levels. The list can be accessed in the data management section of our SFB website (https://sfb1158.de/index.php/rdm-resources).

Our consortium is actively engaged in a number of international and national RDM initiatives, including the NFDI initiative in Germany, which promotes the development of high-level

infrastructure and services across various scientific disciplines, including neuroscience and bioimaging (NFDI Neuroscience (https://nfdi-neuro.de/) and NFDI4BIOIMAGE (https://nfdi4bioimage.de/). Our involvement with different task areas of these community-led initiatives supports the development of a sustainable and community-based RDM strategy. Our SFB puts efforts in following the recommendations of the International Neuroinformatics Coordinating Facility (INCF) and harmonizing our RDM efforts by using community-developed standards that have been accepted as international standards for neurophysiology and neuroimaging datasets (for example, BIDS, NWB 2.0, etc.). Resources such as FAIRsharing (Sansone et al. 2019) and the UK Digital Curation Centre provide a comparative view of data and metadata standards. In addition to these domain-specific initiatives, we are engaging with Research Data Alliance (RDA) and European Open Science Cloud (EOSC) initiatives to adopt and develop new resources for open data exchange across technologies and scientific disciplines.

Project-Specific Data Management Plans: Funding agencies and research organizations are increasingly requesting data management plans (DMPs) when submitting a grant application. The obligation to submit a DMP and timeline depends on the requirements of the funding organizations. DMPs should be created early on, ideally when applying for funds or at the beginning of a research project, and updated as needed. For example, ERC-funded projects that participate in the Horizon 2020 Open Research Data (ORD) pilot are required to submit the first version of their DMP within six months after the start of their grant. Open access publications are encouraged, and grantees should demonstrate FAIR-compliant data management and resource use. However, some research studies involving sensitive data are exempt from these requirements.

The DMP developed for each research project highlights relevant information regarding research data and associated metadata that is required for research result reproducibility. Preliminary versions of DMPs can surely assist participating labs in making informed decisions about their data management resource requirements (financial support or personnel). DMPOnline (https://dmponline.dcc.ac.uk/) and RDMO (https://rdmorganiser.github.io/) are two commercial open-source software solutions for creating custom DMPs (Bryant et al., 2010; Donnelly et al., 2010). Several DMP templates have already been made available in response to funding agency criteria (https://dmponline.dcc.ac.uk/public templates).

We designed three DMP templates after defining and categorizing the data management needs for each project depending on its experimental model type: human, animal, and human-animal tandem (see supplementary information 3.1, 3.2 and 3.3 respectively). The templates can also be found on Zenodo: https://doi.org/10.5281/zenodo.4410128), [27].

Individual project DMPs can be created using these templates, or if a dataset requires particular RDM resources, a dataset-specific DMP can be created. These DMP templates cover questions about how data is handled at each stage of the project, including a general project description; experimental and dataset descriptions; specific data documentation (types of data and experimental models; methods for acquisition and collection; questionnaires; analysis software); decisions on data and metadata standards and formats; and proposed plans for organization, access, sharing, short- and long-term storage, re-use, and implementation costs. The document, once prepared, explains the management of the research data acquired, reviewed, and processed as part of the SFB1158 initiatives. The template includes some generic questions regarding best practices for each stage of the data management lifecycle that may be answered early in the project, while domain-specific questions can be answered later in the project.

Data storage, organization, and sharing: Projects in the SFB1158 generate massive volumes of data of various types and rely on data interoperability among labs. It is strongly advised that researchers securely store full datasets (e.g. raw, preprocessed, and analysis files, codes, etc.) associated with published findings and results, as this promotes the consortium's goal of further engaging in open science. Many commercial online storage systems are accessible for use, but researchers must ensure that they choose reliable solutions with security measures.

We aimed for simple and efficient solutions for secure data transfer between collaborators, with controlled access, all while balancing ease of access for research. We recommend university-approved data storage services (see Figure 3) to guarantee data privacy and confidentiality. For collaborative projects or work groups, a common storage place is requested to guarantee that data is easily accessible to all project members. Heidelberg University network connects the consortium's labs and university departments, using a 10 GB capacity network to expedite data transfer among institutes and facilities. Similar services are available at the other participating SFB labs from other institutions. Data stored on dedicated storage spaces provided by SDS@hd, a large-scale storage facility with a capacity of 20 PB, can be accessed only by project members with the appropriate authorization and shared with internal collaborators. Similar solutions are available at other SFB research groups and institutions. Regarding human datasets (including clinical data), researchers use a storage server with restricted access. Designated personnel have the authority and responsibility to enable access to internal collaborators. When necessary, access can be given to external collaborators by assigning guest accounts.

Typically, data can be arranged in a logical folder structure. This requires additional simple measures. Ideally, a generic scheme for structuring and organizing data can be applied across all projects or for data of a particular kind. Consistent folder organization will depend on the type of acquired research data in a project as well as the governance procedures. To this end, many SFB 1158 projects are using logical file and folder templates to support systematic data organization. The folder structure templates for research repositories have been developed in collaboration with the DFG-funded NFDI-neuro initiative and three neuroscience SFBs (SFB 1158, SFB 1315, and SFB /TRR 135). Our goal is to provide researchers with an easy way to handle their project digital files and datasets on different data infrastructure services, both locally (e.g., SDS@hd (https://www.urz.uni-heidelberg.de/de/service-katalog/speicher/sdshd-scientific-data-storage)) and also on subject-specific data repositories such as GIN: a Modern Research Data Management System for Neuroscience. The current developments involve the implementation of the GIN-tonic application, an extension of GIN that uses templates and advanced Git technologies to facilitate data sharing, project management, and research collaboration. The template structure can be downloaded and used here: Zenodo. https://doi.org/10.5281/zenodo.4410128), [28]. These folder structure templates can be customized based on the type of experiment or data modality as well as the analysis processes that should be integrated with existing data organization systems. These templates, which are mostly workflow-based, are customizable to meet special requirements for individual experiments and analyses, ensuring that the structure may support rather than restrict research practices. These folder configurations can be utilized on desktop PCs, data versioning systems (e.g., GIN or DataLad), external hard drives, or any storage device to accommodate various data sets generated during experiments, independent of format.

Another service that our SFB1158 members frequently use is heiBOX (https://www.urz.uni-heidelberg.de/de/service-katalog/collaboration-und-digitale-lehre/heibox). This service includes backup, synchronization, and storage of small research data and document files (Text, PDF, and Office files) in unencrypted libraries, which are readily found using a full-text search incorporated into the online interface and app connecting the heiBOX as a remote hard drive. Guest accounts can be requested for data interchange with external collaborators.

In addition to providing support for access and use of internal university resources for data storage and sharing, the SFB1158 also supports adoption of innovative community-developed solutions. For example, considering that some SFB1158 projects are now running for multiple funding periods and are performing extended analyses. This includes, for example, data comparison between various groups of pain patients collected at different funding periods, or associations between various types of data modalities (e.g., data collected using

electroencephalography for the first funding period, and fMRI data during the next funding period); or simply comparisons between various analysis toolboxes (e.g., fMRIPrep vs. SPM). Versioning of data sets, along with software and code, becomes critical for such projects as data files and metadata are updated over time. Even in the case of complete datasets published or submitted in a repository, versioning helps in tracking changes in the data files or metadata that are incorporated after data re-use or re-analysis. There are platforms such as DataLad (a US-German collaboration for computational neuroscience project (https://www.datalad.org/) and GIN (https://gin.g-node.org/) that may effectively compensate for a lack of local resources. These data hosting and sharing platforms can also ensure data versioning and encourage reproducible management of scientific data. Both DataLad and GIN are based on git and git-annex to provide support for large datasets. Datalad and GIN are interoperable, as datasets hosted on either of these platforms can be accessed via git-compatible systems. Moreover, the GIN service can be deployed locally at all the participating labs and can be used as an in-house storage server and web user interface for DataLad datasets. Another example resource is the Open Science Framework (https://osf.io/), which is an open-source web-based application for supporting collaborative workflow development. Open Science Grid (https://opensciencegrid.org/) is another emerging collaborative platform that can integrate both data hosting and processing/analysis computing resources.

Data and metadata documentation and standardization: For collaborative research, the data standardization step becomes crucial for interoperability and data sharing [29], but is quite challenging to implement, given the wide range of methodologies represented in the consortium. It was unrealistic to advocate or propose that a single data standard could accommodate the wide range of data modalities and analysis applications that are frequently used within the consortium. This is one area in which the development of local, customized solutions is essential. Many SFB projects, for example, integrate techniques for electrophysiological recordings and optogenetic manipulations combined with behavioral analyses in rodents, resulting in a variety of disparate file formats and unorganized metadata. As a result, we have devised a set of strategies to ensure that the datasets can be thoroughly documented and converted into open data standards with minimum effort.

In order to encourage standardization of datasets generated within the collaborative studies across the consortium, we focused on data documentation as a key first step. We encouraged consortium-wide adoption of electronic lab notebooks (ELN) to help researchers document experimental protocols in well-annotated electronic form at an early stage of the research project. An ELN promotes the documentation and traceability of the research process along

the lines of good scientific practice. There are several commercial and open-source ELN options available for documentation of experiments and results. The Harvard Biomedical Data Management Group has created an Electronic Lab Notebook Matrix (https://zenodo.org/record/4723753) that contains information on a wide range of currently available software.

We initially tested multiple available ELN options, both licensed and open-source, in order to select an appropriate option for our consortium projects and individual lab requirements. We also considered several parameters, such as licensing options, security aspects, implementation and maintenance costs, ease of access and integration with existing resources, and other domain-specific features that may be required. Based on these factors, we selected two options: elabFTW (https://www.elabftw.net/) and Labfolder (https://www.labfolder.com/). The Competence Centre for Research Data (Kompetenzzentrum Forschungsdaten, KFD), a joint institution run by the Library and Computing Centre at the University of Heidelberg, established a web-based instance of elabFTW with a fully encrypted service and data backed up on university servers: (https://www.urz.uni-heidelberg.de/en/service-catalogue/software-and-applications/elabftw) (https://elabftw.uni-heidelberg.de/login.php).

The service can also be utilized as a central service for documenting shared or collaborative experimental methods or protocols carried out by two or more SFB labs. A dedicated team can be created for each project, where members belonging to participating labs can have access. We emphasized the importance of creating templates for the most common types of experiments (based on design protocols, biological methods, etc.) performed in a single project, and users can easily link the experimental metadata. ELN within our RDM framework can be used as a platform for documenting minimum metadata that is automatically generated while performing an experiment. The experimental metadata (e.g., microscope specifications, data acquisition settings) is stored in a standardized manner using a generic metadata file format such as JSON (JavaScript Object Notation) or XML and is compatible with open file formats.

Recommended data standards for different data types: All human research projects acquire multimodal data (neuroimaging, neurophysiological, behavior, psychometrics, etc.). Within the plan of implementing good practices for data management, we followed recent developments in data standards and methodologies to make our data interoperable.

For this purpose, we developed some standard protocols for each data type with the respective metadata. In the case of MRI, we developed standard MR acquisition protocols for anatomical and functional scans in terms of image resolution, duration, and type of acquisition (e.g., resting-state or task-based functional MRI). Consistent acquisition parameters across

studies allows researchers to use similar preprocessing pipelines and the pooling of data across studies, enabling them to increase sample size or for comparison purposes. For example, we can directly compare the structure and function of various populations acquired in different projects, such as individuals with chronic back pain or fibromyalgia patients.

Our goals of data integration and homogeneity were facilitated by the recent opening of the Center for Innovative Psychiatric and Psychotherapeutic Research (https://www.zi-mannheim.de/en/research/zipp-e.html), an extensive, modern research infrastructure with access to neuroimaging, experimental, pharmacological, and psychotherapeutic techniques. In this center, the researchers share the laboratories and equipment, which allows the collection of homogenous data types and data formats for behavioral (e.g., motor), sensory (e.g., sensory testing), or psychological data. In addition, we set up a core set of standardized assessments (e.g., motor paradigms, the use of electronic diaries for pain assessments, quantitative sensory testing, stress-induced analgesia), and psychological questionnaires (e.g., HADS, MPI [30, 31]) to be used across all relevant studies.

We are currently extending these collaborative efforts by developing a data infrastructure platform that will set up a data registry for human volunteers. This consists of setting up a participant identifier shared by all projects, making it possible to find out if the volunteer participated in different projects, therefore avoiding redundant data acquisition. This collaborative work will be coordinated by the data infrastructure project within the consortium dedicated to implementing, testing, optimizing, and standardizing protocols and models to be implemented in all projects.

For clinical projects involving human studies, we have achieved a considerable amount of progress by adopting the Brain Imaging Data Structure (BIDS) for anonymization, organization, and annotation of neuroimaging and behavioral data. BIDS also includes support for other multimodal data, longitudinal and multi-session studies, and physiological metadata collected during MRI experiments. This feature reduces unnecessary manual input of metadata by researchers. It is beneficial for data handling within the laboratory as well as the development of standardized acquisition and analysis workflows. BIDS is currently being extended to support other types of neuroscientific data, such as electrophysiological data recorded in animals (BIDS-animal-ephys, https://neuroinformatics.incf.org/node/242).

Regarding data storage, we set up a storage server for anonymized data in accordance with accepted ethical and quality standards to maintain data protection and privacy. The original sensitive data are stored separately and with restricted access to reduce the risk of disclosure or unauthorized access. The data are then uploaded to the laboratory server after anonymization. The server is used as a shared infrastructure where a set of open-source software is available for data preprocessing and analysis. We are using custom scripts for the

anonymization of MRI datasets. Currently, these scripts are command line only, but we envision developing a tool with a Graphical User Interface (GUI) that is interactive and user-friendly. The custom codes used for anonymization, preprocessing, and analysis are available online via GitHub and released under the BSD Licenses (https://github.com/SFB1158RDM/SFB1158_MRHuman).

After anonymization, the data are available for sharing within and outside of the laboratory. The data can also be made publicly available with proper security measures and quality control. The user keeps control over the data and has the ability to adjust the sharing policy as needed.

Animal Electrophysiology data: A variety of proprietary file formats for raw data as well as for intermediate preprocessing/analysis, such as Cambridge Electronic Design Spike2 (.smrx), Neuralynx (.ncs) are used to gather electrophysiology datasets. Another observation is that many of these formats can only be read or accessed using proprietary software, imposing additional constraints on focusing on a single or even a number of open data standards. Additionally, many of these formats lacked adequate support for metadata documentation.

To enable thorough metadata description, it was important to adopt an open data format. Yet, it was impractical to include all of these raw and intermediate preprocessing file types in a single standard format. In order to devise practical solutions for standardizing such diverse datasets with varying file formats, we divided our standardization approach into two major goals: 1) metadata documentation and standardization; and 2) conversion of various acquisition raw file formats into standard data formats incorporating all types of metadata (basic experimental details, acquisition settings and parameters, and analysis metadata).

As a result, we started exploring and assessing the available data and metadata standardization resources (open-source tools, data conversion pipelines, file formats and their specifications) that could potentially be utilized for our consortium's use cases. We assessed a number of open-source tools and data formats (specific to neurophysiology), including data standards and models such as Neuroscience Information Exchange (NIX) Format, Neurodata Without Borders 2.0 (NWB) (Rübel et al. 2019) etc., data versioning tools (e.g., DataLad, GIN), metadata collection tools (e.g., CEDAR, NIDM, odML), data representation models (Neo) Python library Electrophysiology Analysis Toolkit (Elephant), data analysis tools (e.g., Elephant, FieldTrip), PyNN (Davison et al (Garcia et al. 2014) etc. The data formats are compatible with a wide range of modern software packages and analysis tools, including

Brainstorm, Elephant, Spike2, and NeuroExplorer, and the majority of these tools are open-source.

The SFB1158 encourages the development of new tools and pipelines to reduce the time and effort usually needed by individual labs. For metadata management across all the neurophysiology projects within the consortium, a web-based GUI was designed in collaboration with (https://www.catalystneuro.com/, funded by SFB internal funds), and the source code and installation guide are available for use at https://github.com/catalystneuro/heidelberg-metadata-gui. The metadata handling GUI allows standardized documentation of metadata collected from neurophysiology experiments [32].

To support the diverse electrophysiological approaches used across the consortium and maintain ease-of-use, an initial set of JSON-schema for a few electrophysiology experimental types, i.e., extracellular electrophysiology and optical physiology, are made available. To expedite routine metadata entry, additional default field values can be set up for each laboratory. The GUI uses key metadata fields and possible values sourced from the consortium and stored in a centralized location. The GUI enables export of metadata in JSON file format detailing the experiment once the experimenter has entered at least a minimal amount of metadata. The parameters for preprocessing procedures, such as spike-sorting or filtering, or machine-readable information provided in raw data files can be combined with JSON files, which can be saved locally or centrally. In order to build data files in the required domain-specific data formats, the metadata must contain the minimum number of metadata fields. Community data standards like NWB 2.0 and the resulting metadata file are both compatible. The resulting JSON files containing standardized experimental metadata are imported in electronic lab notebooks (such as eLabFTW) and also used for submission of complete datasets into open data repositories and archiving later in the research process.

Furthermore, some electrophysiology projects within the consortium focuses on adoption of NWB 2.0 data standard and are leveraging a variety of open-source software community solutions (Buccino et al. 2019; Rübel et al. 2019) to build data conversion tools based on pre-existing tools, such as NWB data conversion tools. (https://github.com/catalystneuro/nwb-conversion-tools). NWB 2.0 supports a wide range of data modalities, including electrophysiology (extracellular recordings, intracellular recordings, and electrocorticography) and optophysiology (2-photon imaging, fluorescent wide-field images, and so on). The NWB 2.0 format contains all of the metadata (for example, voltage has a sampling rate and is connected to electrodes) that is required to specify the neurophysiology experiments parameters, and the data can be shared between labs in a fully standardized format. Another useful strategy was the use of modern analysis and visualization software packages such as

SpikeInterface (Buccino et al. 2019), which supports import and export of data in NWB format. It elegantly solves the problem of importing hardware-specific acquisition formats into a common environment while also providing preprocessing capabilities and streamlined access to a variety of spike-sorting algorithms. Using high-performance computing clusters and remote visualization tools (e.g. bwVisu), it was possible to overcome the challenge of real-time processing of large electrophysiology datasets from multiple recordings.

Animal neuroimaging data standardization: Additional assessment techniques in animals include electroencephalography (EEG), multiple modalities of magnetic resonance imaging (MRI), and positron emission tomography (PET).

The datasets generated by microscopic imaging techniques and from a variety of acquisition devices, such as the repetitive in vivo multiphoton imaging experiments conducted over a 20-24 week period in living mice, are challenging to standardize. Similar to neurophysiology experiments, researchers view and analyze microscopic imaging data in proprietary file formats (such as TIFF and.nd2 formats for images). There are several contributing factors when it comes to the adoption of existing open data standards. Our standardization strategy in this case focuses primarily on tools that are interoperable with pre-existing services such as local data storage platforms used for storing imaging datasets, bioimaging software applications (ImageJ/Fiji, etc.) utilized for analysis of data, type of electronic lab notebooks adopted within the consortium labs, and use of HPC clusters. In order to achieve this, a GUI is under development that will support the import of multiple acquisition file formats that are collected from SFB1158 imaging projects, which will allow automated extraction of metadata from raw files and store data in a standardized format such as OME-XML or JSON. The GUI will also allow the incorporation of missing metadata values and will allow users to create more fields in order to support the various other acquired file formats. The GUI will allow users to export the data in more open-source and standardized formats such as OME-XML, OME-NGFF, or ZARR (https://zarr.readthedocs.io/en/stable/). The objective is to create a feasible level of automatic interoperability with existing data analysis and visualization tools as well as electronic lab notebooks.

Behavioral data standardization: Within the SFB1158, all rodent projects adhere to standardized experimental procedures for behavior assays across all models. Projects in the consortium are collecting behaviour datasets in file formats such as video files (avi), the original raw ASCII log files, text-based file formats, .csv and also the stored detailed stimulus material (e.g., wav and png files) etc. However, there is no single coordinated data standardization strategy for every stage of behavioral data, from data collection to analysis, thus making it difficult to generalize and thus group different behavioral paradigms. To

facilitate some level of data harmonization, we adopted a very simple and intuitive approach. First and foremost, we encourage each project to share its experimental protocols (standardized SOPs were already created during the first funding period), hardware and acquisition settings, and preprocessing and analysis software. We then followed the same procedures as described above for standardizing the dataset for microscopic neuroimaging. We are putting systems in place for automatically extracting metadata from behavioral data. We are incorporating open-source programs that already exist, such as the Bio-Formats Importer plugin for Fiji, which reads data. By using the plugin, the acquisition metadata can be standardized and entered into a single OME data model. It will extract and set basic metadata values such as spatial calibration if they are available in the file. This aids in defining parameters for behavioral paradigms. Another simple step we took was to ensure that if a single project combines behavioral data with any other type of experimental data (e.g., electrophysiological recordings or neuroimaging), we prioritized adopting the exact metadata and data standards that were chosen for other data types. The current approach is not fully automated, but it does allow for an initial level of data documentation, which will encourage further standardization.

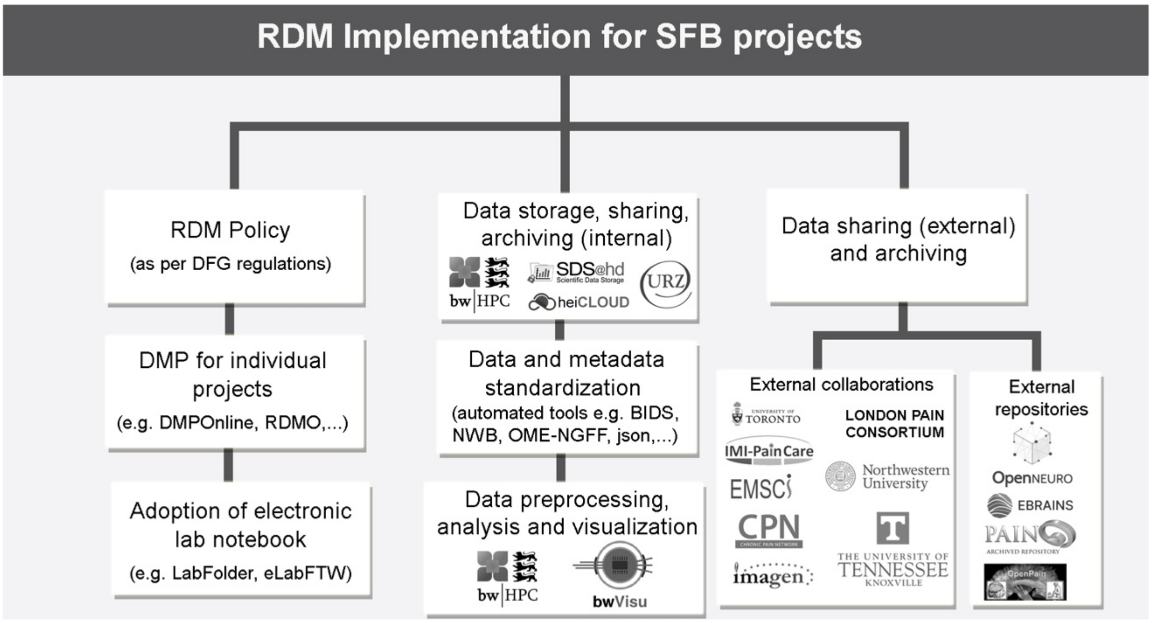

Figure 3: Schematic of the data management implementation for SFB projects.
RDM: research data management; DMP: data management plan.

Data processing, analysis, and visualization: Several projects and laboratories in the consortium use laboratory-based analysis infrastructure, such as local computers, shared

analysis workstations (laboratory computers with GPUs and pre-installed acquisition and analysis tools that are shared by several members), and computational servers that are run by individual laboratories or groups. The SFB1158 highlights the importance of keeping track of every step, from initial data recording to the analysis, and proper documentation of analysis pipelines and scripts. As a constructive starting point, the SFB1158 data manager has set up a dedicated organization account on Github (https://github.com/SFB1158RDM) where multiple repositories with analysis code and scripts can be hosted and shared for each SFB1158 project. The SFB1158's data management organization repositories are maintained by the data manager and access is given to the authorized project members. Our RDM services also include providing technical assistance with the refactoring of lab-customized preprocessing analysis pipelines (MATLAB and Python scripts) into more organized workflows. For example, some electrophysiology projects have adopted Jupyter lab notebooks for data analysis pipelines.

In addition to local infrastructure and computing servers, there is university infrastructure available for more demanding data processing tasks, such as running computationally intensive analyses of heterogeneous and large-scale datasets such as imaging in humans and rodents. An application for access to these services can be made by individual laboratories with application support from the data manager. For instance, some SFB labs are utilizing bwForCluster MLS&WISO (https://www.urz.uni-heidelberg.de/de/service-katalog/hochleistungsrechnen/bwforcluster-mlswiso) and a detailed tutorial on access and use is available at (https://github.com/SFB1158RDM/HPCtutorial). This eliminates administrative and technical barriers to performing computationally intensive tasks such as large scale modelling, simulation, and analysis projects, e.g., Neuropixels systems. Less experienced users can take advantage of machine learning-trained analysis algorithms and create reports. The HPC allows job scheduling using slurm and also sets up reproducible computing environments (ex. docker, singularity) to optionally run the modules on the HPC for particularly large data sets that are streamed directly to SDS@hd during acquisition, e.g., chronic recordings with dense electrode array or image segmentation for chronic imaging (miniscope) with the perspective of running standardized analysis workflows.

Similarly, for processing massive (e.g., neuroimaging) datasets, some projects are utilizing the heiCLOUD (https://heicloud.uni-heidelberg.de/), an Infrastructure-as-a-Service (IaaS) cloud service that provides virtual machines that may be customized and utilized as needed for the project. A possible scenario for heiCLOUD usage within our consortium is to install complex and computationally expensive software packages and perform concurrent

processing of massive neuroimaging datasets. This provides powerful workstations for data analysis.

Another application that some of the SFB1158 projects are utilizing is bwVISU (https://www.bwvisu.de/), a remote service for scientists (universities in Baden-Württemberg state, Germany), as well as the corresponding software stack to deploy such a service on-premises. It has an interactive web front-end that supports large-scale data analysis and visualization without much human intervention. We are currently refactoring and developing new tools for the automated running of deep learning applications on bwVISU. With such extra computational resources, it is possible to set up automated analysis workflows on HPC that could allow for faster, more accurate diagnoses in near-real time. The goal of this project is to implement a deep-learning application programming interface (API) for image data processing and to provide a platform for the scientific community to directly compare and integrate data generated across our consortium projects. The development of an open-source and extensible platform to train and share deep-learning models will guarantee high standards in many image analysis workflows and additionally reduce the amount of annotated data necessary for training. supervised deep-learning algorithms. For example, our initial efforts involve integrating the most commonly used deep-learning image analysis tools to make the initial GUI more flexible for model training and inferences. Some of the considered tools include CARE for image enhancement [33], Noise2Void (image denoising) [34], CellPose (cell segmentation) [35] and Elektronn3 (EM data segmentation, https://github.com/ELEKTRONN/elektronn3).

Data dissemination: The SFB1158 encourages the submission of published datasets to repositories and the publication of open-access articles. Unless specifically exempted datasets, all consortium research data must be made available via a suitable data publishing or archiving platform under appropriate authorization and licensing (for example, a *creative commons* or open source initiative-approved (software) license) to allow for flexible public reuse. Any third-party data gathered by or provided for consortium research activities are equally subject to these standards, unless data use agreements clearly restrict it.

Several data repository options can be found in online resources such as re3data.org and/or FAIRsharing.org. We assist consortium members in archiving and publishing data on heiDATA (https://heidata.uni-heidelberg.de), an institutional repository for research data based on the DataVerse Project (https://dataverse.org/) [36] [37]. This repository supports data documentation as well as administrative, technical, and descriptive metadata; each dataset is

given a persistent identification, a citable address, and a DataCite ID (Brase, 2010). In addition to data publication, the heiDATA repository allows data access via a simple interface. This provides for the permanent publication of data records in the repository while also providing a separate interface for regular data access. Complete datasets are collected in a dataverse established for SFB1158 projects (research data, code, documentation, and metadata). The dataverse is available at https://heidata.uni-heidelberg.de/dataverse/data-sfb1158 .

The KFD also provides specific guidelines and procedures on data repositories, archiving, licensing, and access restrictions in order to provide public access to these datasets. The KFD is currently developing heiARCHIVE (https://heiarchive.uni-heidelberg.de/de/node/1), a digital long-term archive for research data preservation and archiving, which will be available to the SFB1158 during the next funding period or near the end of the current funding period (https://doi.org/10.11588/heibooks.979.c13740; https://doi.org/10.11588/heidok.00029723). It will provide researchers with an easy-to-use end-user platform for archiving their research data (at least for 10 years), as well as the option of performing OAIS-compatible long-term preservation with features such as format recognition, validation, and file conversion of appropriate file formats.

Aside from these institutional solutions, several other public neuroscience repositories are rapidly being developed. It is worthwhile to search both domain/format-specific repositories and scientific repositories (for example, OpenPain http://www.openpain.org/ and PainRepository https://www.painrepository.org). In the context of bioimaging data storage and sharing, the EMBL-EBI BioImage Archive (BIA) is a large-scale, centralized data resource that hosts reference imaging data. The OpenfMRI project [38], which was originally created for the free and open sharing of raw MRI datasets (old datasets available at https://legacy.openfmri.org/), has since expanded to include datasets from other neuroimaging modalities such as MEG, EEG, and PET, and has been renamed the OpenNeuro Project (https://openneuro.org), [39]. Certain repositories require datasets to be submitted in a standardized format; for example, OpenNeuro (which accepts anonymized human-derived datasets), OMEGA (Open MEG Archive, exclusively for MEG data), and MNE-BIDS have all adopted the BIDS format (which links BIDS and MNE-python analysis tool for MEG and EEG data). In addition to providing basic features such as data hosting and support for metadata files, there are repositories that provide restricted data sharing and anonymization services, which are highly suitable for publishing datasets from clinical projects. The Cancer Imaging Archive (TCIA) and the LONI Image Data Archive (IDA) are two examples.

Other neuroscience-focused data repositories with specific purposes include G-node GIN for datasets derived from both human and non-human organisms, BrainLife (human neuroimaging), (https://brainlife.io/), Distributed Archives for Neurophysiology Data Integration (DANDI) CITE, and Fenix-backed EBRAINS (CITE). The HBP EBRAINS data curation team may assist with data submission and integration, as well as provide defined embargo durations to allow for progressive disclosure. The EU-funded Human Brain Project produced EBRAINS, an open European digital research infrastructure that provides one of the most complete platforms for sharing brain research data of various types, spatial and temporal scales.

Aside from these domain-specific repositories, numerous well-known open data repositories, sharing and management platforms accept data from a wide range of disciplines. Zenodo and Dryad, for example, is an online archive that manages research datasets with metadata and allows long-term data access via persistent identification. Figshare, a commercial free data repository with unique features such as custom storage options, version control, visualization, metadata customization, data curation using DOI, and so on, is one example. The EMBL SourceData SmartFigure (https://sourcedata.embo.org/) focuses on the scientific figure as a sharing unit, combining data sharing and visualization. The Harvard Dataverse Network is both a platform for institutions and a data repository implemented on FAIR data principles to publish, share, reference, extract, and analyze research data. Consortiums offering support and access to cloud computing, such as OpenScienceGrid, JetstreamCloud, Fenix, and the European Commision-backed European Open Science Cloud (EOSC), can support analysis if institutional solutions are not available.

CONCLUSION

We present a data management strategy that we developed and put into practice within the framework of a collaborative research center, encompassing both basic and clinical research on humans and animals. In order to foster FAIR and open science, this strategy strives to offer practical solutions for multimodal and multidisciplinary research.

This strategy is composed of three adaptive and incremental phases: planning, implementation, and dissemination. Consistent communication with consortium project members during the planning and implementation phases was crucial in order to identify the most helpful RDM measures. We spent a considerable amount of time learning about publicly accessible tools and services and new developments in the RDM field that could be beneficial for our consortium.

In the planning phase, we evaluate common data management practices across projects. We categorize projects based on the typical population studied and the common measurement methods used. We focused on addressing issues such as metadata management; experimental protocol documentation; preprocessing and analysis pipelines; data storage and volume; data sharing, data dissemination and archiving, sensitive data-related issues that arise when working with highly diverse and heterogeneous data. The complexity was subsequently raised by the major RDM challenges encountered in tandem projects that work with both human and animal populations, such as including data and metadata standardization, the integration of various different data types, and the harmonization of datasets and analysis workflows.

In the implementation phase, we presented some innovative solutions based on pre-existing and customized solutions developed for flexible and incremental data management solutions with a focus on research collaborations. We discuss the implementation of project-specific data management plans, structured based on data acquisition, processing, and analysis methods across the SFB1158 projects. Relatively simple measures, such as offering electronic lab notebook (ELN) options for documenting experimental protocols or tutorials on high-performance computing resource access, regular data seminars on basic RDM tools (such as data versioning tools, code and workflow management software, etc.), can improve data management practices in noticeable ways across the consortium.

We focused on the development of new tools for metadata organization and management depending upon the requirements of each project and the type of data collected. In animal projects, we assisted with migration from proprietary formats and supported experimental annotation and organization. Moreover, for large datasets, we provided easy access to software and tools on large web-based applications to enable interactive analysis and visualization.

For the organization of human projects, we adopted standard protocols to associate various data types with the respective metadata. In the case of MRI, we standardized MR acquisition protocols, data organization, and preprocessing pipelines. Behavioral, sensory testing, and psychological questionnaires were standardized by a service project.

The SFB1158 emphasizes that active communication and engagement with general and domain-specific RDM community initiatives is required for the development of RDM strategies for any large-scale research consortium. Modern research infrastructure and technological advancements, such as web-based technologies for sharing data and analysis tools provide opportunities to increase the reproducibility of research outcomes in both basic and translational neuroscience.

Further development of this RDM model with more specialized technical infrastructure is envisioned for the next period of the consortium. A federated data sharing approach is required

for multi-site and multi-species projects, which will allow for the integration of data from different computer systems for participating labs that are geographically distributed without moving the data to a centralized location.

AUTHORS' CONTRIBUTION

All authors contributed equally to this work in conceptualization and writing.

FUNDING

This work was supported by the Deutsche Forschungsgemeinschaft (SFB1158 /Z Project).

ACKNOWLEDMENTS

The authors gratefully acknowledge the members of the Research Data Competence Center (KFD), University of Heidelberg, especially, Martin Baumann, Jochen Apel, Georg Schwesinger, Alexander Haller for their constant RDM support and services and technical guidance; Carlo Beretta for his support in bioimaging data management, Pooja Gupta and Anne Seller for their help with coordination; and the members of the German NFDI-Neuro and NFDI4Bioimage communities for informative discussions on RDM in neuroscience. We also thank Paul Naser and Philipp Roth for assistance with older versions of figures. We acknowledge the contributions of the catalystneuro team for the development of the metadata GUI (funded by internal funds from SFB1158). The authors acknowledge support by the state of Baden-Württemberg through bwHPC. We fully acknowledge the data services SDS@hd supported by the Ministry of Science, Research and the Arts Baden-Württemberg (MWK) and the German Research Foundation (DFG) through grants INST 35/1314-1 FUGG and INST 35/1503-1 FUGG. Rebecca Mease is supported by the Brigitte-Schlieben-Lange Programm and the Chica Heinz Schaller Foundation.

COMPETING INTERESTS STATEMENT:

This manuscript reflects only the author's views and the funding agencies are not liable for any use that may be made of the information contained therein. The authors declare no conflict of interest.

REFERENCES

1. Gouwens NW, Sorensen SA, Berg J, Lee C, Jarsky T, Ting J, et al. Classification of electrophysiological and morphological neuron types in the mouse visual cortex. Nat Neurosci. 2019;22 7:1182-95. doi:10.1038/s41593-019-0417-0.
2. Juavinett AL, Bekheet G and Churchland AK. Chronically implanted Neuropixels probes enable high-yield recordings in freely moving mice. Elife. 2019;8 doi:10.7554/eLife.47188.
3. Kleinfeld D, Luan L, Mitra PP, Robinson JT, Sarpeshkar R, Shepard K, et al. Can One Concurrently Record Electrical Spikes from Every Neuron in a Mammalian Brain? Neuron. 2019;103 6:1005-15. doi:10.1016/j.neuron.2019.08.011.
4. Sych Y, Chernysheva M, Sumanovski LT and Helmchen F. High-density multi-fiber photometry for studying large-scale brain circuit dynamics. Nat Methods. 2019;16 6:553-60. doi:10.1038/s41592-019-0400-4.
5. Zeisel A, Hochgerner H, Lonnerberg P, Johnsson A, Memic F, van der Zwan J, et al. Molecular Architecture of the Mouse Nervous System. Cell. 2018;174 4:999-1014 e22. doi:10.1016/j.cell.2018.06.021.
6. Rubel O, Dougherty M, Prabhat, Denes P, Conant D, Chang EF, et al. Methods for Specifying Scientific Data Standards and Modeling Relationships with Applications to Neuroscience. Front Neuroinform. 2016;10:48. doi:10.3389/fninf.2016.00048.
7. Wilkinson MD, Dumontier M, Aalbersberg IJ, Appleton G, Axton M, Baak A, et al. The FAIR Guiding Principles for scientific data management and stewardship. Sci Data. 2016;3:160018. doi:10.1038/sdata.2016.18.
8. Wilkinson MD, Dumontier M, Jan Aalbersberg I, Appleton G, Axton M, Baak A, et al. Addendum: The FAIR Guiding Principles for scientific data management and stewardship. Sci Data. 2019;6 1:6. doi:10.1038/s41597-019-0009-6.
9. Madan CR. Scan Once, Analyse Many: Using Large Open-Access Neuroimaging Datasets to Understand the Brain. Neuroinformatics. 2021; doi:10.1007/s12021-021-09519-6.
10. Gorgolewski KJ, Auer T, Calhoun VD, Craddock RC, Das S, Duff EP, et al. The brain imaging data structure, a format for organizing and describing outputs of neuroimaging experiments. Sci Data. 2016;3:160044. doi:10.1038/sdata.2016.44.
11. Pernet CR, Appelhoff S, Gorgolewski KJ, Flandin G, Phillips C, Delorme A, et al. EEG-BIDS, an extension to the brain imaging data structure for electroencephalography. Sci Data. 2019;6 1:103. doi:10.1038/s41597-019-0104-8.
12. Niso G, Gorgolewski KJ, Bock E, Brooks TL, Flandin G, Gramfort A, et al. MEG-BIDS, the brain imaging data structure extended to magnetoencephalography. Sci Data. 2018;5:180110. doi:10.1038/sdata.2018.110.
13. Sariyar M, Schluender I, Smee C and Suhr S. Sharing and Reuse of Sensitive Data and Samples: Supporting Researchers in Identifying Ethical and Legal Requirements. Biopreserv Biobank. 2015;13 4:263-70. doi:10.1089/bio.2015.0014.
14. White T, Blok E and Calhoun VD. Data sharing and privacy issues in neuroimaging research: Opportunities, obstacles, challenges, and monsters under the bed. Hum Brain Mapp. 2022;43 1:278-91. doi:10.1002/hbm.25120.
15. Eke DO, Bernard A, Bjaalie JG, Chavarriaga R, Hanakawa T, Hannan AJ, et al. International data governance for neuroscience. Neuron. 2022;110 4:600-12. doi:10.1016/j.neuron.2021.11.017.

16. Foster ED, Whipple EC and Rios GR. Implementing an institution-wide electronic lab notebook initiative. J Med Libr Assoc. 2022;110 2:222-7. doi:10.5195/jmla.2022.1407.
17. Vasilevsky NA, Minnier J, Haendel MA and Champieux RE. Reproducible and reusable research: are journal data sharing policies meeting the mark? PeerJ. 2017;5:e3208. doi:10.7717/peerj.3208.
18. Jun JJ, Steinmetz NA, Siegle JH, Denman DJ, Bauza M, Barbarits B, et al. Fully integrated silicon probes for high-density recording of neural activity. Nature. 2017;551 7679:232-6. doi:10.1038/nature24636.
19. Steinmetz NA, Koch C, Harris KD and Carandini M. Challenges and opportunities for large-scale electrophysiology with Neuropixels probes. Curr Opin Neurobiol. 2018;50:92-100. doi:10.1016/j.conb.2018.01.009.
20. Gangadharan V, Zheng H, Taberner FJ, Landry J, Nees TA, Pistolic J, et al. Neuropathic pain caused by miswiring and abnormal end organ targeting. Nature. 2022;606 7912:137-45. doi:10.1038/s41586-022-04777-z.
21. Robbins M, Christensen CN, Kaminski CF and Zlatic M. Calcium imaging analysis - how far have we come? F1000Res. 2021;10:258. doi:10.12688/f1000research.51755.2.
22. Pnevmatikakis EA. Analysis pipelines for calcium imaging data. Curr Opin Neurobiol. 2019;55:15-21. doi:10.1016/j.conb.2018.11.004.
23. Cantu DA, Wang B, Gongwer MW, He CX, Goel A, Suresh A, et al. EZcalcium: Open-Source Toolbox for Analysis of Calcium Imaging Data. Front Neural Circuits. 2020;14:25. doi:10.3389/fncir.2020.00025.
24. Giovannucci A, Friedrich J, Gunn P, Kalfon J, Brown BL, Koay SA, et al. CaImAn an open source tool for scalable calcium imaging data analysis. Elife. 2019;8 doi:10.7554/eLife.38173.
25. Molter J, Avitan L and Goodhill GJ. Detecting neural assemblies in calcium imaging data. BMC Biol. 2018;16 1:143. doi:10.1186/s12915-018-0606-4.
26. Sare RM, Lemons A and Smith CB. Behavior Testing in Rodents: Highlighting Potential Confounds Affecting Variability and Reproducibility. Brain Sci. 2021;11 4 doi:10.3390/brainsci11040522.
27. Mittal D. CRC1158 Data Management Plan Templates (1.0). Zenodo. 2022;https://doi.org/10.5281/zenodo.6917120.
28. Colomb J, Arendt T, Mittal D and Sehara K. Folder structure template for research repositories (2.0). Zenodo. 2020;https://doi.org/10.5281/zenodo.4314361.
29. Poline JB, Kennedy DN, Sommer FT, Ascoli GA, Van Essen DC, Ferguson AR, et al. Is Neuroscience FAIR? A Call for Collaborative Standardisation of Neuroscience Data. Neuroinformatics. 2022; doi:10.1007/s12021-021-09557-0.
30. Herrmann C, Buss U and Snait R. Hospital Anxiety and Depression Scale- Deutsche Version: Ein Fragebogen zur Erfasung von Angst une Depressivitat in der somatischen Medizin. [HADS-D - Hospital Anxiety and Depression Scale - German version: A questionnaire to assess anxiety and depression in somatic medicine]. Bern: Huber. 1995.
31. Flor H, Rudy TE, Birbaumer N, Streit B and Schugens MM. Zur Anwendbarkeit des West Haven-Yale Multidimensional Pain Inventory im deutschen Sprachraum: Daten zur Reliabilitat und Validitat des MPI-D, [The Applicability of the West Haven-Yale

Multidimensional Pain Inventory in German speaking countries: data on the reliability and validity of the MPI-D]. Der Schmerz. 1990;4:82-7.
32. Tauffer L, Vaz V and Dichter B. SFB1158 Metadata GUI. 2022.
33. Weigert M, Schmidt U, Boothe T, Muller A, Dibrov A, Jain A, et al. Content-aware image restoration: pushing the limits of fluorescence microscopy. Nat Methods. 2018;15 12:1090-7. doi:10.1038/s41592-018-0216-7.
34. Krull A, Buchholz T-O and Jug F. Noise2Void - Learning Denoising From Single Noisy Images. 2019 IEEE/CVF Conference on Computer Vision and Pattern Recognition (CVPR). 2019:2124-32.
35. Stringer C, Wang T, Michaelos M and Pachitariu M. Cellpose: a generalist algorithm for cellular segmentation. Nat Methods. 2021;18 1:100-6. doi:10.1038/s41592-020-01018-x.
36. re3data.org. heiDATA. Registry of Research Data Repositories 2021;re3data.org doi:http://doi.org/10.17616/R3QW6J.
37. King G. An Introduction to the Dataverse Network as an Infrastructure for Data Sharing. Sociological Methods and Research. 2007;36:173-99.
38. Poldrack RA, Barch DM, Mitchell JP, Wager TD, Wagner AD, Devlin JT, et al. Toward open sharing of task-based fMRI data: the OpenfMRI project. Front Neuroinform. 2013;7:12. doi:10.3389/fninf.2013.00012.
39. Markiewicz CJ, Gorgolewski KJ, Feingold F, Blair R, Halchenko YO, Miller E, et al. The OpenNeuro resource for sharing of neuroscience data. Elife. 2021;10 doi:10.7554/eLife.71774.

Suppl.1

Click here to access/download

Supplementary Material

Suppl1.pdf

Suppl.2

Click here to access/download
Supplementary Material
Suppl2.pdf

Suppl.3.1

Click here to access/download
Supplementary Material
Supp3.1_Tandem_Projects.pdf

Suppl.3.2

Click here to access/download
Supplementary Material
Supp3.2_Human_Projects.pdf

Suppl.3.3

Click here to access/download
Supplementary Material
Supp3.3_Animal_Projects.pdf

CoverLetter

Click here to access/download;Personal Cover;MittalEtAl_coverLetter.pdf

HEIDELBERG
FACULTY OF
MEDICINE

Pharmacology Institute | Im Neuenheimer Feld 366 | 69120 Heidelberg

Deepti Mittal, MS

Data Manager, Heidelberg Pain Consortium, Pharmacology Institute, Heidelberg University

Medical Faculty, Heidelberg University

Phone: +49 15203531055
Secretary office: +49 6221 54 16601
deepti.mittal@pharma.uni-heidelberg.de

www.sfb1158.de

Mannheim, September 23rd, 2022

Dear Editors of GigaScience,

We are a collaborative research consortium dedicated to understanding the neural mechanisms of pain. We are enclosing a manuscript entitled: "Data management strategy for a Collaborative Research Centre".

We had the idea to write this manuscript because we have been recently contacted by many researchers (working in large scale consortiums or in small research groups) to ask for advice about implementation of a data management strategy in their own institutions. We aim therefore to share our consortium's current strategies for research data management with a larger audience.

The Heidelberg Pain Consortium (https://www.sfb1158.de/) comprises a total of 34 principal researchers who have come together to build a collaborative project specifically aimed at understanding the neural circuitry underlying pain. With our goal of adopting and establishing modern data management and standardization practices within the consortium, we are presenting domain-specific strategies, tools and data standards for research data management addressing the following key points:

- Research data management (RDM) policy for a collaborative research consortium.
- Brief overview of general and neuroscience-specific data management resources.
- Introduction of open data and metadata standards and development of reproducible analysis workflows.
- Data and Metadata standardization tools.
- Sensitive data management (anonymization) solutions.
- Data archiving and publishing solutions.

My co-authors and I believe these topics will be of interest to the readers of your journal and would support the establishment of RDM strategies for other large consortia in the neuroscience community. With this pre-submission inquiry, we hope that you will consider evaluating the manuscript to see if it falls within your journal's scope. Our title, and abstract are copied below. Thank you for your consideration and time.

Sincerely,
Deepti Mittal
Rebecca Mease
Thomas Kuner
Herta Flor
Rohini Kuner
Jamila Andoh & the Consortium
