# Supplementary material for: Data management strategy for a collaborative research center
Source: Gigascience. 2023 Jul 4;12:giad049. doi: 10.1093/gigascience/giad049 (PMC10318494; doi:10.1093/gigascience/giad049)
Supplement: giad049_GIGA-D-22-00262_Revision_1 [file giad049_giga-d-22-00262_revision_1.pdf]

GigaScience

Data management strategy for a Collaborative Research Centre

--Manuscript Draft--

**Manuscript Number:**	GIGA-D-22-00262R1
**Full Title:**	Data management strategy for a Collaborative Research Centre
**Article Type:**	Review
**Funding Information:**	Deutsche Forschungsgemeinschaft (SFB1158/Z Project) — Not applicable
**Abstract:**	The importance of effective research data management (RDM) strategies to support the generation of findable, accessible, interoperable, and reusable (FAIR) neuroscience data grows with each advance in data acquisition techniques and research methods. In order to maximize the impact of diverse research strategies, multi-disciplinary, large-scale neuroscience research consortia face a number of unsolved challenges in RDM. While open science principles are largely accepted, it is practically difficult for researchers to prioritize RDM over other pressing demands. Implementation of a coherent, executable RDM plan for consortia spanning animal, human, and clinical studies is becoming more and more challenging.<br>Here, we present a RDM strategy implemented for the Heidelberg collaborative research consortium (https://www.sfb1158.de/). Our consortium combines basic and clinical research in diverse populations (animals and humans) and produces highly heterogeneous and multimodal research data (e.g., neurophysiology, neuroimaging, genetics, behavior).<br>We present a concrete strategy for initiating early-stage RDM and FAIR data generation for large-scale collaborative research consortia, with a focus on sustainable solutions that incentivize incremental RDM while respecting research-specific requirements.
**Corresponding Author:**	Jamila Andoh<br>Central Institute of Mental Health: Zentralinstitut fur Seelische Gesundheit<br>Mannheim, GERMANY
**Corresponding Author Secondary Information:**	
**Corresponding Author's Institution:**	Central Institute of Mental Health: Zentralinstitut fur Seelische Gesundheit
**Corresponding Author's Secondary Institution:**	
**First Author:**	Deepti Mittal
**First Author Secondary Information:**	
**Order of Authors:**	Deepti Mittal<br>Rebecca Mease<br>Thomas Kuner<br>Herta Flor<br>Rohini Kuner<br>Jamila Andoh
**Order of Authors Secondary Information:**	
**Response to Reviewers:**	Reviewer reports:<br><br>1.Reviewer #1:<br>The authors describe a multistage plan that they implemented to improve data standards and management for the Heidelberg Pain Consortium. Having done similar work, I can tell that a great deal of thought and work went into creating this management program, however the paper will need substantial refactoring for that effort to shine through. The pieces of a great paper are already here, they are just

difficult to follow.

Major comments:

1.1 Comment: pg 3, lines 31-33 "Implementing effective data management strategies and ethical rules for the reuse and sharing of high-quality data reduces redundant research, optimizes public research funding and reduces animal use."
This seems like a significant claim supporting the need for RDMs, but has no citation. Is there evidence that RDMs reduce animal use or reduce redundant research? What does optimized public research funding look like? This statement is surrounded by several other assertions with little explanation, e.g.: "A clear set of regulations and guidelines must be established before sharing human data gathered from clinical or non-clinical populations. Specific rules addressing privacy issues, established processes for data protection, data use and reuse, and the preservation of sensitive data are required. It is essential to make data accessible and understandable to remote (or future) collaborators in order to maximize the potential of existing algorithms and tools and accelerate the creation of new ones."

We thank the reviewer for the comments. We now reformulated our sentence and added some references as follows, see p. 27 lines 26-40:
"Implementing effective data management strategies and ethical rules for the reuse and sharing of high-quality data may reduce redundant research [5, 6]. One of DM goals is to make study results freely available through open-access publishing. By investing in collaborative projects with long-term goals, it ensures that the data are organized in a way that makes them easily accessible and retrievable for future use. This makes it easier and faster to develop new research projects, as well as to replicate or build on existing studies, which should have a direct impact on public research funding (https://sor.senate.ca.gov/sites/sor.senate.ca.gov/files/0842%20policy%20matters%20Research%2003.18%20Final.pdf). Moreover, effective DM strategies can also enable to optimize public research funding by pooling resources and infrastructure from multiple sources and bringing together experts from universities, research institutes, and other community organizations to work on long-term interdisciplinary projects. Furthermore, by ensuring proper RDM, researchers should be able to reduce animal use. For example, making informed decisions about which animal models to use for their studies should enable to use the same animals for multiple experiments, instead of having to continuously use new animals for each study. In addition, sharing previously acquired data with adequate metadata, or reuse of control group data from similar studies can avoid repeating in vivo work [1-4]."

1.2 Comment: I would like to see the introduction more thoroughly discuss the problems with data management and elaborate on these asserted solutions.

We agree with the reviewer's comment. We now added a section on data management challenges in the introduction section, starting with the common data management challenges across projects (including challenges due to the diversity in data types, challenges due to diversity in acquisition, preprocessing, and analysis approaches, metadata challenges, data storage and volume, challenges in data documentation, data sharing and dissemination challenges and challenges due to sensitive data). Then we present some RDM challenges for specific projects (including behavioral experiments, electrophysiology with high-density probes, large-scale in vivo two-photon calcium imaging, human-animal tandem projects), see p. 4 lines 6-39, p. 5-7, p. 8 lines 1-13.

1.3 Comment: There are multiple other places throughout the text where there are ideas stated as facts without references or elaboration, that I would like to see evidence for, or at least a discussion of.
For e.g. pg 13, lines 1-2 "There is, furthermore, a lack of efficient software to adequately segregate and maintain control over sensitive data. It is important to provide researchers working with sensitive data or samples with truly "useful" tools that do not require pre-existing, in-depth knowledge of legal and ethical requirements, or

	time to delve into the details.”<br><br>Following the reviewer’s comment, we now added some information and references in the discussion section entitled “Sharing sensitive data from human projects”, p. 30 lines 26-40, p. 31, p. 32 lines 1-7.<br>“Sharing sensitive data from human projects<br>The sharing of human data gathered from clinical or non-clinical populations in neuroscience research is essential for advancing science and producing important public health benefits. A clear set of regulations and guidelines must be established before sharing human data gathered from clinical or non-clinical populations. Specific rules addressing privacy issues, established processes for data protection, data use and reuse, and the preservation of sensitive data are required. It is essential to make data accessible and understandable to remote (or future) collaborators in order to maximize the potential of existing algorithms and tools and accelerate the creation of new ones. Regulations and guidelines should ensure that the data are used for the purpose for which they were gathered and protect the rights of participants in the research. These guidelines should also cover how data should be collected, stored, shared, and destroyed. They also specify the types of data that must be kept confidential and the appropriate methods for handling and safeguarding the data. They should also ensure that the data are secure, kept confidential, and not used for marketing or other commercial purposes. Additionally, regulations should ensure that the data are used responsibly and that they are not used to discriminate against people with disabilities or other vulnerable populations. Ethical rules for the reuse and sharing of data should be based on the principle of informed consent. This includes obtaining consent from the original data collectors or from research participants, as well as obtaining permission from any third parties involved in the data collection. Researchers should also seek to minimize the risk of data misuse or breach of confidentiality, and any data that are shared should be done so in a secure manner.<br>Additionally, there is a lack of efficient software programs to adequately segregate and maintain control over sensitive data. It can be difficult to develop effective software that is secure, user-friendly, and cost-effective. The maintenance of such software requires a significant investment of resources, and often there is a lack of funding available for such measures. Finally, the adoption of such software requires investment in training and resources, which many organizations may be unwilling to do. The legal and ethical requirements surrounding the use of sensitive data are often complex and difficult to understand, leading to confusion and ambiguity about the best way to protect them. It is important to provide researchers working with sensitive data or samples with truly "useful" tools that do not require pre-existing, in-depth knowledge of legal and ethical requirements, or time to delve into the details. Such tools are important to ensure that sensitive data are protected and securely stored. Use of such tools can help researchers to make informed decisions about how to best use and manage sensitive data, allowing them to work with it in an ethical and responsible manner. Finally, these tools can help to reduce the risk of data breaches and data misuse, which can have serious consequences for the people and organizations whose data are affected. By providing such tools, researchers can focus on their research and not on legal and ethical considerations, thus saving their time and resources.<br>There are several software tools that can be used to maintain sensitive patient data in neuroscience research. Some examples include a web-based platform REDCap (Research Electronic Data Capture) [121], an open-source imaging informatics platform XNAT (XNAT Central) [122], LORIS: Longitudinal Online Research and Imaging System [123] etc. It is important to note that the security features of these tools may vary and should be evaluated before use. In addition to software tools, secure data storage and access protocols should also be in place to ensure that sensitive patient data are protected.<br>We are currently expanding our collaborative efforts by creating a data infrastructure platform that will establish a GDPR-compliant data registry (PainReg-registry, based on the Germany-wide ParaReg registry (www.parareg.de) [124] for human volunteers. To facilitate cross-project data merging, a core clinical data set will be defined. This entails assigning a unique identifier to each study participant that is shared by all projects, allowing researchers to determine whether the volunteer participated in multiple projects. This will allow organizations to share a same pool of volunteers for multiple studies, resulting in less redundant data acquisition. This can result in cost and time savings, as well as increased data collection accuracy. We, for example, experienced that a same study participant could be tested twice, and was assigned different IDs

(belonging to different projects), resulting in redundant data acquisition and therefore unnecessary increased costs, particularly for genetic analysis.
Furthermore, the data registry will ensure that data privacy regulations are strictly followed by obtaining participants' consent to access data for secondary or follow-up studies. This will also include an identity management feature to limit access to authorized users. The registry will contain a wide range of data, including brain imaging, genetic, cognitive, and physiological data. This collaborative work will be coordinated by the consortium's future data infrastructure project, which will be tasked with implementing, testing, optimizing, and standardizing data analysis procedures and models that will be utilized in all projects.

1.4 and pg 13, lines 26 - 29; Why do available commercial cloud storage solutions come with unreliable and slow backup and restoration services, as well as no obvious access paths or interfaces for easy migration onto the analysis platforms?

Following the reviewer's comment we now added the following information p. 8 lines 27-38:
"Many commercial cloud storage solutions are designed primarily for convenience and cost savings rather than for robust backup and restoration services. As a result, these services often lack the features necessary for reliable and fast backup and restoration, such as automated data backups, point-in-time recovery, and incremental backups. Additionally, many cloud storage solutions lack the necessary APIs, scripts, and tools that would allow for easy migration of data onto the analysis platforms. This makes data migration difficult and inefficient, and it can significantly slow down the analysis process.
Assuring access to secure and optimal storage solutions that can be integrated with workflows encompassing data acquisition, intermediate analysis, and archiving is thus a major challenge. Creating backups and storing multiple copies of large volumes of datasets, the need for collaborative and parallel access by multiple people, and use across a diverse range of computational workflows all pose major challenges to storage servers."

1.5 pg 15, lines 13 - 19;
We now added a reference p. 9 line 19:
Higgins SG, Nogiwa-Valdez AA, Stevens MM. Considerations for implementing electronic laboratory notebooks in an academic research environment. Nat Protoc. 2022 Feb;17(2):179-189. doi: 10.1038/s41596-021-00645-8. Epub 2022 Jan 14. PMID: 35031789.

1.6 pg 16, lines 4-5; Another significant issue with submitting data to general repositories is that they do not have enough support for certain types and formats of data.

Following the reviewer's comment, we now added some information and some references p. 10 lines 13-28:
Another significant issue with submitting data to general repositories is that the latter might not have adequate support for certain types and formats of data [54, 55]. For example, if the data are in a non-standard format, the repository may not be able to process it correctly or even accept it. Additionally, repositories may not have specialized tools or services to help researchers convert, organize or analyze the data. This can be especially problematic for data that are highly specific, such as medical records or geospatial data. Without the necessary support, researchers may be unable to make full use of the data or even access them. Additionally, general repositories may not have the same level of curation and organization as a specialized repository, which can make it harder for researchers to evaluate the quality and relevance of the data. This can lead to a lack of reproducibility and increased difficulty in building on previous research. Therefore, it is recommended for researchers to submit their data to specialized repositories that are tailored to their specific field. Neuroscience specific repositories, on the other hand, are specifically designed to accommodate the unique needs of neuroscience data (openNeuro). They are often managed by experts in the field and have the necessary infrastructure to ensure the safe and secure storage of data. Furthermore, they often provide additional services such as data analysis, curation and visualization tools, which allow researchers to better understand and use

Powered by Editorial Manager® and ProduXion Manager® from Aries Systems Corporation

	the data.<br><br>1.7 pg 4,lines 2-5 "While the majority of collaborative research consortiums collect a wide variety of multidimensional datasets, the majority of these datasets are typically inadequate for modern research methods and infrastructure." As above, I'm interested in how this is quantified, or if there is a reference, and to have a more complete discussion of this idea.<br><br>Following the reviewer's suggestion, we now added some information and some references p. 6 lines 1-22:<br>"The availability of robust neuroscience resources such as high-performance computing (HPC) clusters [23-25], modern workflow technologies (e.g., Galaxy [26], Snakemake [27] etc.), cloud-enabled storage and computing infrastructures (e.g., Amazon AWS, Google Cloud [28]), secure databases [29], repositories [30], and analysis platforms are fundamentally changing how research in neuroscience is communicated and linked to existing raw data and findings [31]. Such tools are allowing researchers to utilize diverse techniques and produce massive amounts of high-dimensional data (large sample size, various models and conditions), which provides greater statistical power and the opportunity to do more robust secondary data analysis [32]. However, the data-driven neuroscience approach, as a whole, is questioned by a number of technical issues that must be addressed before it can be fully realized.<br>While the majority of collaborative research consortiums collect diverse multidimensional datasets, one of the primary challenges is that the majority of these datasets are typically inadequate for modern research methods and infrastructure [33] . Before committing to any of these tools for processing and analysis of collected datasets, it is important to understand data in terms of the number, volume, size and complexity of data, the types and formats of data, and the accuracy and completeness of the datasets. Despite similarities in experimental design, researchers in neuroscience experiments frequently organize and describe their data in their own way, even within the same research group. As a result, datasets collected from different research groups for a single project addressing the same scientific question may not be in formats suitable for comparison and pooling, limiting their interoperability and reuse. The data formats collected in each project are typically determined by the acquisition and measurement method, intermediate pre-processing, or analysis software. This can lead to data and metadata being stored in different locations."<br><br>1.8 Comment: Beginning on line 27 of page 4, the introduction veers into describing many technical details of specific studies. In my first read-through, I actually thought that I had accidentally flipped to a different paper. None of these details seem relevant to the larger concept of creating an effective RDM strategy, and are very disorienting. I would remove them entirely.<br><br>We agree with the reviewer's comment and have reorganized the introduction section, which is now more focused on presenting RDM challenges (see also comment 1.2).<br><br>1.9 Comment: Figure 1. In order to understand how the authors have implemented an RDM plan, it seems important for the reader to understand the basic structure of the consortium, however, I do not understand how to interpret figure 1. It appears that the animal projects somehow encapsulate all the others? Are the relative sizes of the colored portions meaningful? The number of tandem projects is half that of animals, which seems to agree with the peach width being smaller than the green. However there are only 4 human projects for the very large yellow section, and 1 for the black. I would drop this figure entirely. I think Figure 2 does a much better job of describing the consortia, and is much easier to read. Further, pg 8, lines 5 - 12 are a near copy of the legend for Figure 1, but refer to Figure 2 A anyway.<br><br>Following the reviewer's comment we now removed figure 1 and edited the "old" figure 2 (now figure 1) and adapted it to the current structure of the consortium.

Figure 1: The Heidelberg Pain Consortium investigates various populations: humans, rodents, and tandem using various modalities: neuroimaging, neurophysiology, behavior, psychometrics, and genetics (A). Each data modality can be recorded using different techniques: MRI= magnetic resonance imaging; PET/MR: positron emission tomography/magnetic resonance; fNIRS: functional near infrared spectroscopy; NIBS: non-invasive brain stimulations; EEG: electroencephalography; MEG: magnetoencephalography; Genetics; Two-photon imaging; Behavior; EMA: ecological momentary assessment; psychometrics; physiology; VR: virtual reality; optogenetics (B).

1.10 Comment: Beginning on pg 6, line 35 to pg 7 line 16; and then again beginning on pg 10, line 34 to pg 18, line 16, this entire text is general background of problems in data management, and specifically around the particular challenges of this consortia. It is altogether a much better introduction than the existing introduction. It covers all of the missing context, as well as discussing the types of studies the Heidelberg Pain Consortium needs to work with without delving into over technical details. I recommend excising this from the results and using it to replace everything after the second paragraph of the introduction. With minor adjustments for grammar, it will be much better there. The move will also make your actual results, which currently don't begin until pg 18, line 18, much easier to find and follow.

We thank the reviewer for the suggestion and we now reorganized the introduction which contains the challenges section (see also comment 1.2).

1.11 Comment: In the results (beginning pg 18, line 18), there are several sections where important discussion points are interspersed with the results, making it difficult to tell which are the work of the consortium, and which are simply informational. It would greatly improve readability if these paragraphs were pulled out and integrated into a discussion section, which doesn't currently exist. As I'm finding it difficult to tell which parts are your work, below is my best guess at the paragraphs that should be moved, or dropped:
pg 19, lines 27 - 32
pg 20, lines 1 - 12
pg 21 line 1 - pg 22 line 18
pg 22 lines 19 - 29 (excluding the title, which should be moved to line 30), pg 22 line 34 - pg 23 line 5
pg 23 lines 18 - 28
pg 26 line 10 - pg 27 line 6
pg 27 line 31 - pg 28 line 1 (excluding the title, which should be moved to line 2)
pg 30 line 33 - pg 32 line 21
For the "Recommended data standards for different data types", "Animal neuroimaging data standardization", and "Data processing, analysis, and visualization" sections, the results and discussion pieces are not as easily pulled out as paragraphs, so I have not listed each individual sentence here. Instead, I would suggest looking at these sections with an eye towards picking out which are results of something your consortium worked on, and which are expounding on ideas or comparing it to other work.
Clearly moving all of those to the discussion will require some editing to make it make sense, but I think that if you make the discussion follow the same minor heading structure you have in the results it will be a relatively painless change. The first part of the current results: pg 5, line 23 - pg 6 line 34 would be very good if moved to be the first few paragraphs of the discussion, but are too vague to be part of the results.

We thank the reviewer for the comments. We have now restructured the RDM Implementation (section II in the manuscript) to highlight the strategies used by our consortium. We also added a discussion section.

1.12 Comment: pg 6, line 37 - pg 7 line 5. "We specifically describe our ongoing RDM efforts, which are divided into two sections: 1) RDM Planning Phase: Identifying common RDM procedures across consortium projects; Evaluating common data

	management challenges; Special RDM challenges in specific projects; 2) RDM Implementation Phase: resource allocation decisions and implementation of key resources and 3) Data Dissemination (data management continues even after the end of the funding period)." A minor typo is that it should say three sections, not two. Rather more importantly, both here and in the conclusion, it lists three phases, but phase three does not appear in the actual text. It is not described along with the others in the conclusion, or mentioned at all in the results.<br><br>We now revised the manuscript and divided the main text into two main sections: section 1: RDM planning phase and section II: RDM implementation, followed by a discussion.<br><br><br>Minor comments:<br><br>1.13 Comment: SBF is never defined, and the authors seem to use SFB1158, SFB and SFB 1158 as synonyms, but I'm not sure if that is the intent.<br><br>Thank you for pointing this out. We have now homogenized the terms and use CRC in the entire manuscript. We also added the following information in the funding section p. 40, lines 35-37:<br><br>“Collaborative Research Centers (CRC; short SFB for German ‘Sonderforschungsbereich’) are university research projects which are funded by the German Research Foundation (DFG), generally for a period of up to 12 years. https://www.dfg.de/foerderung/programme/koordinierte_programme/sfb/”<br><br><br>1.14 Comment: I would move all of the funding information on pg 4 to the funding section, it's distracting here<br>We now moved the funding section to the end of the manuscript p. 40, lines 35-40.<br><br><br>1.15 Comment: There are several minor grammar issues. I am not copying them all here, as I'm sure they'll be fixed in the rearrangement, but:<br>pg 3 line 26 should be 'cooperative'<br>We corrected now all typos and the entire manuscript was meticulously proofread.<br><br><br>1.16 Comment: pg 6, line 7; its unclear what "progressively practical RDM approaches" means<br>We apologize for the lack of clarity. We aimed to emphasize how practical our RDM efforts are in terms of allocating resources and creating new services while taking pre-existing services into account.<br>We now clarified the RDM approach p. 30 lines 1-8:<br>“We also focused on developing RDM strategies that included joint efforts and cooperation between consortium members and other large-scale consortiums and collaborative centers within Germany. We acknowledge the common issue of data organization for different projects in collaborative centers. Joint efforts were made for the development of a data organization strategy that works for most of the projects within the consortium working in similar research areas. Our main goal was to engage more directly in several overlooked aspects of managing data in a large collaborative consortium while keeping the global neuroscience community in mind. We encouraged CRC 1158 projects to utilize logical file and folder templates to support systematic data organization.”<br><br><br>1.16 Comment: pg 7, line 1 should be 'three'<br><br>The reviewer is correct. In the current version we however decided to have two phases and one discussion (see also comment 1.12)<br><br><br>1.18 Comment: pg 7, line 21 'can' should be 'could'

Powered by Editorial Manager® and ProduXion Manager® from Aries Systems Corporation

We have now removed the previous sentence.

1.19 Comment: pg 11, lines 23-26, this sentence doesn't seem to finish "In cases where a laboratory produces a large dataset from a single experiment, and the collected metadata are too complex and stored in multiple files having different formats that are only read by the acquisition software or by customized codes written for internal use."
We now reformulated the sentence as follows p. 7 lines 36-40:
“Moreover, a laboratory can generate a large dataset from a single experiment or a single dataset from multiple experiments, and the collected metadata can be very complex and stored in multiple files with different formats only readable by the acquisition software or by customized codes written for internal use. In such cases, a consolidated strategy for unifying data into a single format that can be read by a variety of software applications and analyzed in an efficient and reproducible manner is necessary.”

1.20 Comment: pg 12, lines 17 - 12, this sentence/paragraph seems to be missing words/doesn't finish "Although many advances have been made regarding organization, annotation, and description of research datasets, for example using the Brain Imaging Data Structure (BIDS, https://smex-ctp.trendmicro.com:443/wis/clicktime/v1/query?url=https%3a%2f%2fbids.neuroimaging.io%2f&umid=69f29401-5290-40bc-943f-fc4fb21e8086&auth=9ed9a254ae8a0a504cee5b89eb68d1de87cd0c46-a38d2b534d1942c51ddbf5754176077e75f11c73) for neuroimaging data standards [10] EEG-BIDS for neurophysiology data (e.g., electroencephalography), [11], or MEG-BIDS for magnetoencephalography data [12], whereas other data modalities (e.g., EMA) do not have existing standards yet.

We thank the reviewer for his feedback. We have now rephrased the sentence as follows p. 5 lines 26-28:
“Although many advances have been made regarding the organization, annotation, and description of research datasets, there is still much work to be done to ensure that datasets are fully standardized and can be accurately shared and reused [18].”

1.21 Comment: pg 15, lines 4 - 7, this sentence seems to me missing words: "However, while choosing an electronic laboratory notebook for a large-scale neuroscience consortium spanning diverse experimental protocols, the availability of clear documentation and application-centric features become an overarching issue."

We apologize for the lack of clarity. We have now rephrased the sentence as follows p. 9 lines 3-5:
“For a large-scale neuroscience consortium spanning diverse experimental protocols, it is important to select an ELN that can provide comprehensive support for a wide range of experimental protocols and provides flexibility to add domain-specific features if required [51].”
2.Reviewer #2

2.1 Comment: The authors of the manuscript "Data management strategy for a Collaborative Research Centre" take the reader on a timely tour de force through the various aspects surrounding the establishment of RDM measures in a large, collaborative research project spanning multiple highly heterogeneous subprojects and partners. I found the topic of the paper highly interesting and engaging, as it is based on the experiences and procedures encountered in a real-world scenario. The challenges and solutions are presented in a clear, well-written and structured way in two overarching chapters, respectively, that make the article enjoyable to read. Also, I found the breadth of challenges well captured.
While I am enthusiastic to see the manuscript published, I have several points and suggestions I would like to put up for consideration.
As a general point, I found the article quite lengthy due to a lot of detail that was put into the description of the individual challenges. At the same time, I found many challenges described in the planning phase to be rather high-level statements, and I

	was often hoping to have these more embedded into the actual problems encountered in the SFB to give illustrative examples. I would like suggest to the authors to reconsider if they find specific examples of some of the more abstract challenges that were actually encountered (similar, e.g., to the last part in "planning" section about specific RDM challenges). The reasoning here is that I find the strong point of this article that it comes from actual experience, and I found this not ideally reflected in some of the more abstract descriptions. An example illustrating this suggestion is given in the detailed points below (p10, l31-32). To compensate, perhaps some of the details in the manuscript could be shorted.<br>Also, as a general point I found that many of the existing tools/services/... were not referenced (by either publication, DOI, RRDI, https link or at least some pointer to the resource).<br><br>We thank the reviewer for the helpful comments. Following the feedback from reviewer 1 and reviewer 2 we have now restructured the whole manuscript. We have moved the data management challenges in the introduction, restructured section 1 (planning phase) and section 2 (implementation phase), and added some examples and some references. We also added a discussion section.<br><br>2.2 Comment: - p5, Figure 1: I did not find this illustration very helpful, I think a more standard 2D representation with the individual groups and links between groups forming tandems would be better understandable.<br><br>Following the feedback from reviewer 1 and reviewer 2, we now removed the old Figure 1 and edited the old Figure 2 (now Figure 1).<br><br>Figure 1: The Heidelberg Pain Consortium investigates various populations: humans, rodents, and tandem using various modalities: neuroimaging, neurophysiology, behavior, psychometrics, and genetics (A). Each data modality can be recorded using different techniques: MRI= magnetic resonance imaging; PET/MR: positron emission tomography/magnetic resonance; fNIRS: functional near infrared spectroscopy; NIBS: non-invasive brain stimulations; EEG: electroencephalography; MEG: magnetoencephalography; Genetics; Two-photon imaging; Behavior; EMA: ecological momentary assessment; psychometrics; physiology; VR: virtual reality; optogenetics (B).<br><br>2.3 Comment: - p6, l13: I think it would be extremely useful for other consortia to know more about the day-to-day tasks of the data manager, in particular in terms of the job profile -- what background is more useful: neuroscience, computer science, software engineer, project planning/organization? As the job description of a "data manager" is evolving, it would be great to hear what tasks made this SFB successful in cross-group RDM.<br><br>We have now added a sub-section “Data stewards, Community engagements and collaborations” in the discussion section p. 28 lines 37-40, p. 29, p. 30 lines 1-24.<br><br>“Data stewards, Community engagements and collaborations<br>Data managers and stewards are essential for implementation of data policy and governance procedures, especially for large consortiums [115]. These positions are well-suited to individuals with a background in research or computer science, bioinformatics, and strong communication skills. Furthermore, depending on the needs of the consortium, the candidate should have experience developing high-throughput analysis pipelines, domain-specific data structures and standards, open access publishing, and modern data science approaches, high-performance computing environments, cloud computing, data security, and databases, among other things. The role of data manager is diverse, and it works in close collaboration with core computing and library resources to streamline access to the consortium's common research data infrastructure, which is available at the host institutions of participating labs.<br>Data managers should also support ongoing research, advise on best practices for

data handling, and stay informed about current developments in the RDM field. Furthermore, they should act as an important liaison between consortium researchers, collaborators, the university's RDM planning group and computing center, and community organizations. They aid in bridging the gap between the lab-based scientists and the available technical infrastructure and services. Direct assistance in daily tasks such as data organization, tool selection, workflow development, and standardization is indeed beneficial for individual researchers and research groups. Addressing data management tasks relatively early in research timelines is necessary to make the research process more efficient, and to ensure the interoperability and reusability of data sets. The expert guidance of existing infrastructure and resources, such as scientific repositories, databases, legal and ethical issues, etc., is also necessary to promote an effective data-sharing strategy.

Data managers must maintain consistent communication with various research groups and other similar consortiums to establish a community network and links to other scientific communities such as the national research data infrastructure (NFDI) consortia. As a result, data are disseminated throughout the community, and data management techniques specifically designed to facilitate neuroscience research are developed. For example, our consortium is actively engaged in a number of international and national RDM initiatives, including the EBRAINS ((https://ebrains.eu/), National Node Consortium Germany), the NFDI bioimaging initiative in Germany NFDI4BIOIMAGE (https://nfdi4bioimage.de/), which promotes the development of high-level infrastructure and services across various scientific disciplines. Our involvement with different task areas (e.g., Neuromorphic computing (NMC), Data Analytics, Workflows, GDPR, The Virtual Brain Cloud, etc.) of these community-led initiatives supports the development of a sustainable and community-oriented RDM strategy. Our CRC puts efforts into following the recommendations of The International Neuroinformatics Coordinating Facility (INCF; https://www.incf.org) [116] and harmonizing our RDM efforts by using community-developed standards that have been accepted as international standards for neurophysiology and neuroimaging datasets (for example, BIDS, NWB etc., full list available at https://www.incf.org/resources/sbps). Resources such as FAIRsharing (https://fairsharing.org/ [117] and the UK Digital Curation Centre (https://www.dcc.ac.uk/) provide a comparative view of data and metadata standards. In addition to these domain-specific initiatives, we are engaging with Research Data Alliance (RDA, https://www.rd-alliance.org/) and European Open Science Cloud (EOSC, https://eosc-portal.eu/ [118]) initiatives to adopt and develop new resources for open data exchange across technologies and scientific disciplines.

2.4 Comment: - p6, l23: What level of granularity was put into the guideline in terms of "data formatting"? As outlined in later sections, the data is highly heterogeneous.

The reviewer makes an interesting comment. We now added some information p. 28 lines 22-30:

"The acquisition of heterogeneous data in multiple projects can render the process of data formatting challenging, and the data policy guidelines do not specify a level of granularity for data formatting. However, the guidelines do provide general recommendations for how to format such diverse datasets, such as using standard data formats and tagging data with descriptive metadata. We supported individual research groups by providing resources and funds for the implementation of modern tools and infrastructure for compatibility with community RDM standards. We recommended using "standard" formats for data of similar modalities (e.g., neuroimaging data (MRI) is formatted to NIFTI, electrophysiology data is formatted to NWB, etc.) as described in the data standardization sections."

2.4 Comment: - p8, l2: How was the compliance to the survey? Given the complexity of RDM, did researchers need prior education wrt the meaning of RDM related questions of the survey, and were measures put in place to improve the quality of answers?

The reviewer raises interesting questions. Due to the collaborative nature of the consortium and the individual development of RDM strategies, the researchers were quite aware of the importance of data sharing and many aspects of RDM in general. Moreover, the head of the symposium supported and encouraged our initiatives, which facilitated the interaction with researchers and the compliance to the survey. Overall, researchers were familiar with RDM concepts and they welcomed help to optimize

Powered by Editorial Manager® and ProduXion Manager® from Aries Systems Corporation

	RDM procedure.<br>The goal of the survey was to conduct an assessment to better understand the researcher views', their current practices and needs, project-specific support needed from data manager, computing center and university library to establish proper data management practices and their willingness to share data with external collaborators if data manager offers some support in managing their research data. The survey questions are available in the supplementary file. The survey also included questions about data generation, various types and formats of research data generated, infrastructure for data storage and archive, practices of research data sharing, awareness on research data repositories etc. Also, during the second funding period, we focused more on providing support for data standardization and dissemination. Due to the pandemic, we managed to conduct online data seminars and also one on one RDM zoom meetings with each participating lab of the consortium, which helped us to optimize the survey results.<br><br>We now added the following text in the manuscript p. 13 lines 33-39, p. 14 lines 1-5:<br>"Direct involvement with researchers during the planning and initial implementation phases was crucial in order to identify the most helpful RDM measures, these may be as simple as coordinating communication between core IT staff and researchers, and facilitating access to institutional or other pre-existing resources. We devoted significant time to initially gather information about publicly available tools and services that would be useful to the diverse projects within the consortium. Given the large number of laboratories from various institutions participating in the consortium, as well as the increasing number of requirement changes over the course of a project, information was gathered in a variety of ways (virtual individual interviews with project PIs; discussions during online data seminars led by the CRC data manager; and personal meetings with experimentalists and PhD students). Data discussions and regular communication with consortium members have greatly aided our assessment approach. The PIs, or project responsible persons, were required to respond to a variety of data management questions as part of the assessment process (see Supplementary Information 2)."<br><br>2.5 Comment: - p8, Figure 2C: I found panel 2C not immediately clear in terms of the organization and identifying the row/column structure. A slight improvement visually and/or 1-2 sentences of description of the panel in the caption would help.<br><br>Following the reviewer's comment, we now edited Fig 2 (now Figure 1). See also comment 2.2.<br><br>2.6 Comment: - p10, l31-32: This is one concrete example of the general remark above: "manageability issues" are very abstract, an example of what types of problems precisely triggered this statement would be very illuminating and supportive of the statement.<br><br>We thank the reviewer for bringing up this important point. Some of these data manageability challenges include integration of multi-modal datasets; harmonization of heterogeneous datasets; keeping corresponding timing information; achieving and maintaining good data quality (checking for missing data, duplicates); properly securing all the collected data and adhering to privacy and security regulations while enabling access to specific users. We added some lines p. 4, lines 16-23:<br>"These diverse techniques and collected data types raise multiple data manageability issues within and between projects, with broad implications for data interoperability and reuse. Some of these manageability issues are inconsistent data formats, limited harmonization of heterogeneous datasets, time-consuming data acquisition, especially when dealing with large datasets, integration of multimodal datasets, inaccuracy in the representation of collected datasets, difficulties in achieving and maintaining good data quality (checking for missing data and duplicates), ensuring the security of all collected data, and adhering to privacy and security regulations while enabling access to specific users."

Powered by Editorial Manager® and ProduXion Manager® from Aries Systems Corporation

	2.7 Comment: - p11, l2-: The same as the previous comment holds for the challenges associated with multi-modal data, as its unclear on which level the integration step happens. Moreover, I would welcome a more elaborate description of the level of integration of data in a tandem project: how far could researchers go in integration? Did researchers go as far as analyzing data from their tandem partner and vice versa? To which degree could datasets be actually integrated/compared in practice?<br><br>The reviewer makes an interesting comment. The level of integration of data in a tandem project depends on the scope of the project and the goals of the researchers. We now added a section on data integration p. 32, lines 11-40:<br>"Data integration<br>For collaborative research, the data integration and standardization step becomes crucial for interoperability and data sharing [125], but is quite challenging to implement, given the wide range of methodologies represented in the consortium. Early data standardization can have massive benefits for data integration in collaborative projects, this can be achieved by streamlining the use of tools for more replicable and reproducible analysis. The data integration process often depends on individual projects and their underlying workflow and processes. The degree to which this is possible will depend on the modalities used, the subject population, and the experimental design. Researchers may also integrate the raw data collected from each partner into a core dataset. Integrated datasets can provide a more comprehensive understanding of the research question, as well as allowing the researchers to compare the results of their analyses more directly. Depending on the modalities used, the data may need to be transformed or normalized before integration, and the analysis techniques may need to be adapted to the combined dataset.<br>For some projects, researchers can go as far as analyzing data from their tandem partners and vice versa. Some human projects combine fMRI data from one group and EEG data from another group to gain a better understanding of how the two modalities interact. This could also involve combining the datasets, or running analyses on the combined dataset to identify common patterns or trends. However, this process requires careful consideration of the data sources, data formats, and analysis techniques used by individual labs, as well as the selected methods for data fusion and data mining. At the most basic level, researchers can compare the data collected from each partner to identify commonalities and differences in the data. This could include comparing the number and type of modalities used, the subject population, the experimental design, and the type of analysis performed. For example, they could investigate how brain structure (grey matter volume, cortical thickness) relates to behavior. There are also association studies aiming to compare brain activity between two groups of participants (e.g., healthy, chronic pain) in order to explore neural differences in cognition or behavior. They could also examine associations between neural activity in different brain areas and physiological responses of the subject. "<br><br>2.8 Comment: - p14, l15-: Did these reproducibility issues occur in practice?<br><br>These reproducibility issues did occur indeed in practice. We added some lines p. 7 lines 11-18:<br>"Problems with reproducibility [37] are made worse by the fact that the original analyses were done in different software environments, using different operating systems (e.g., Linux, Macintosh), and with different software or software versions. For example, variability in reproducing results from neuroimaging studies has been often investigated [38, 39]. Often, analyzing fMRI data with software packages such as SPM (Statistical Parametric Mapping) [40] or FMRIB Software Library (FSL) [41] might lead to different outcomes. Efforts are however being made to determine sources of variabilities and develop homogenous and standardized computing environments [42]."<br><br>2.9 Comment: - p15, l1-: One could also mention potential legal/data privacy concerns related to selecting an electronic lab notebook.<br><br>We thank the reviewer for the comment. We now added the following information p. 9 lines 22-30:<br>"When selecting an ELN, there are potential legal and data privacy concerns to consider [52]. An ELN is a digital resource which stores confidential information, and

	can be accessed by multiple users. To ensure legal and data privacy compliance, it is important to design ELNs compliant with all applicable laws, regulations and ethical standards. Additionally, it is important to consider the security measures in place for the ELN, including encryption and user authentication measures, to ensure that the stored data remains secure and private. Finally, it is important to consider the terms of service for the ELN, as this will outline how the data is used, stored and shared, as well as any limitations on the use of the data. By considering these potential legal and data privacy concerns, organizations can ensure that their data remain secure and compliant."<br><br>2.10 Comment: - p17, l24: Please reformulate or define "neural assemblies", as this may be ambiguous<br><br>We now defined neural assemblies p. 13 lines 3-40:<br>"neural assemblies (collection of neurons that are activated simultaneously in response to a particular stimulus and form assemblies)"<br><br><br>2.11 Comment: -p17, l15-: I really like this last subsection as it is very concrete and down-to-earth. However, for this paragraph in the section, I found it unclear where exactly the special challenge lies, i.e., what aspects are truly unique to this situation. Are similar problems not equally present, e.g., when looking at the neuropixel probes (p17, l1-) where different spike sorters will produce different results, and potentially similar computationally demanding workflows are required?<br><br>We thank the reviewer for the positive comment.<br>The section "Special RDM challenges" was added because it includes some DM strategies that were specific to some animal projects, whereas the previous section "Common data management challenges across projects" deals with common data collected across the majority of the projects. As the reviewer points out, challenges for neuropixels and two-photon calcium imaging are comparable in terms of size of data sets generated (around 100 GB/hour), which provides some challenges for data storage. Moreover, producing and analyzing such datasets requires high computational complexity and costs, necessitating an adequate computational infrastructure [34, 35].<br>We separated these two techniques because they differ in their spatial sampling and temporal resolutions, and are used to answer to different research questions.<br>To facilitate comprehension, we now changed the title of the subsection to "RDM challenges for specific projects" see p. 11 line 23. We also reformulated the sections on Electrophysiology and neuroimaging p. 12, lines 15-40 and p 13 lines 1-12:<br>"Electrophysiology with high-density probes<br>A few of the animal projects in the consortium make use of new technologies such as high-density Neuropixels probes [63]. Neuropixels datasets are often large (~80 GB/hour) and computationally demanding, which can make it difficult to scale spike sorting workflows across different labs and datasets. Data storage requirements rise as a result of the significant amounts of derived data needed for intermediate processing (such as filtering and spike sorting) and stimulation and/or behavioral parameters (such as optogenetic stimulation, motion or whisker tracking, and task performance). Analysis and post-processing may often require computationally intensive algorithms and hardware acceleration to handle data that cannot be loaded into local memory [64].<br>Due to differences in...
**Additional Information:**	
**Question**	**Response**
Are you submitting this manuscript to a special series or article collection?	No
**Experimental design and statistics**<br><br><br>Full details of the experimental design and statistical methods used should be given	Yes

in the Methods section, as detailed in our Minimum Standards Reporting Checklist. Information essential to interpreting the data presented should be made available in the figure legends.<br><br>Have you included all the information requested in your manuscript?	
**Resources**<br><br>A description of all resources used, including antibodies, cell lines, animals and software tools, with enough information to allow them to be uniquely identified, should be included in the Methods section. Authors are strongly encouraged to cite Research Resource Identifiers (RRIDs) for antibodies, model organisms and tools, where possible.<br><br>Have you included the information requested as detailed in our Minimum Standards Reporting Checklist?	Yes
**Availability of data and materials**<br><br>All datasets and code on which the conclusions of the paper rely must be either included in your submission or deposited in publicly available repositories (where available and ethically appropriate), referencing such data using a unique identifier in the references and in the "Availability of Data and Materials" section of your manuscript.<br><br>Have you have met the above requirement as detailed in our Minimum Standards Reporting Checklist?	Yes

Powered by Editorial Manager® and ProduXion Manager® from Aries Systems Corporation

Manuscript
Click here to access/download;Manuscript;Mittal_manuscript_revised.pdf

Data management strategy for a Collaborative Research Centre

Deepti Mittal[1*], Rebecca Mease[2], Thomas Kuner[3], Herta Flor[4], Rohini Kuner[1], Jamila Andoh[5*]
and the CRC 1158 Consortium

[1] Institute of Pharmacology, Heidelberg University, Heidelberg, Germany
[2] Institute of Physiology and Pathophysiology, Heidelberg University, Heidelberg, Germany
[3] Institute for Anatomy and Cell Biology, Heidelberg University, Mannheim, Germany
[4] Department of Cognitive and Clinical Neuroscience, Central Institute of Mental Health, Medical Faculty Mannheim, Heidelberg University, Mannheim, Germany
[5] Department of Psychiatry and Psychotherapy, Central Institute of Mental Health, Medical Faculty Mannheim, Heidelberg University, Mannheim, Germany

*Corresponding authors:
Deepti Mittal: deepti.mittal@pharma.uni-heidelberg.de, Tel: +49 (0) 6221 / 541 6600
Or Jamila Andoh: jamila.andoh@zi-mannheim.de, Tel: +49 (0) 621 / 1703 6506

ABSTRACT

The importance of effective Research Data Management (RDM) strategies to support the generation of Findable, Accessible, Interoperable, and Reusable (FAIR) neuroscience data grows with each advance in data acquisition techniques and research methods. To maximize the impact of diverse research strategies, multi-disciplinary, large-scale neuroscience research consortia face a number of unsolved challenges in RDM. While open science principles are largely accepted, it is practically difficult for researchers to prioritize RDM over other pressing demands. The implementation of a coherent, executable RDM plan for consortia spanning animal, human, and clinical studies is becoming increasingly challenging. Here, we present a RDM strategy implemented for the Heidelberg Collaborative Research Consortium (https://www.sfb1158.de/). Our consortium combines basic and clinical research in diverse populations (animals and humans) and produces highly heterogeneous and multimodal research data (e.g., neurophysiology, neuroimaging, genetics, behavior). We present a concrete strategy for initiating early-stage RDM and FAIR data generation for large-scale collaborative research consortia, with a focus on sustainable solutions that incentivize incremental RDM while respecting research-specific requirements.

INTRODUCTION

Extensive efforts have recently been made to promote the reproducibility, replicability, and transparency of scientific research. The evolution of open-access publishing [1], open-source data repositories [2], and software applications has transformed the work of researchers in various fields. As research has become more sophisticated, these developments were inevitable, and large-scale multidisciplinary projects have been developed to promote collaborative work.

Research in the field of neuroscience increasingly encompasses a variety of fields, including biophysics, molecular biology, medicine, cognitive neuroscience, psychology, and ethology. Neuroscience datasets are constantly growing as a result of scientific advances in acquisition systems that produce large-scale multimodal datasets [3-7]. Moreover, research institutions are increasingly involved in interdisciplinary collaborative research. Such collaborative developments pose new challenges for research data management (RDM) [8], specifically in terms of data harmonization, use of computational resources and data sharing [9]. Integration of neuroscientific datasets and data sharing is one of the greatest obstacles in a large-scale consortium combining multi-modal and multi-site studies, which becomes more challenging when handled as an afterthought. This has a direct effect on research collaborations and the publishing process [10].

The primary goal of this report is to describe our approach to implementing data management across a collaborative research consortium (Heidelberg-based Collaborative Research Centres, CRC 1158), comprising independent, multidisciplinary research groups with common goal-oriented research, as well as to make recommendations and guidelines for best practices. We specifically describe our ongoing RDM efforts, which are divided into two sections: 1) RDM Planning Phase: evaluating common data management challenges; RDM challenges in specific projects; evaluating CRC researchers' data management requirements 2) RDM Implementation Phase: implementing common RDM procedures across consortium projects; resource allocation decisions and implementation of key resources. This report discusses our experience in developing and implementing a data management strategy and offers concrete solutions to promote multidisciplinary collaborative research and open science objectives.

I. Consortium-wide RDM Planning Phase

The RDM planning phase is critical to ensuring effective and efficient RDM. During this phase, we assess common and project-specific data challenges, review the scope and objectives of our consortium and its research projects, collect data information, implement data management policies and procedures, and create a comprehensive data management plan. This plan must take into account the type of data being collected, stored, and secured; the analysis methods; and any legal or ethical considerations. We also review the issues that have emerged from our consortium's efforts to create coherent RDM planning for diverse datasets, develop common infrastructure, document data and

metadata, establish procedures for sharing and archiving data, and handle sensitive data. This phase provides the foundation for successful data management and compliance with laws and regulations.

Common data management challenges across projects

Challenges due to the diversity in data types:

While developing a sustainable RDM strategy for our CRC, a central challenge was the variety of data types produced by multidisciplinary approaches utilized in individual projects. The projects involve balancing data from basic and clinical research as well as data from animals and humans. Diverse signals are collected at various spatial and temporal scales, such as single cell and network data, genetic (e.g., Genome-Wide Association Studies (GWAS), gene expression profiles, epigenetic modifications), imaging (e.g., magnetic resonance imaging MRI; positron emission tomography PET), intra and extra-cellular electrophysiology, calcium imaging using fluorescence-based microscopy, confocal light sheet microscopy (CLSM), behavioral data (e.g., task performance) and clinical data (e.g., patient-surveys, medication, cognitive assessments and psychological questionnaires) etc. These diverse techniques and collected data types raise multiple data manageability issues within and between projects, with broad implications for data interoperability and reuse. Some of these manageability issues are inconsistent data formats, limited harmonization of heterogeneous datasets, time-consuming data acquisition, especially when dealing with large datasets, integration of multimodal datasets, inaccuracy in the representation of collected datasets, difficulties in achieving and maintaining good data quality (checking for missing data and duplicates), ensuring the security of all collected data, and adhering to privacy and security regulations while enabling access to specific users.

All human research projects in our consortium use a multimodal approach that collects and combines data from two or more of the following methods: MRI including structural (anatomical and diffusion-weighted imaging) and functional: task-based and resting-state; electro- and magnetoencephalography (EEG and MEG, respectively); behavioral; psychometric; and genetics. The use of multiple data sources allows researchers to identify relationships between different modalities, and to gain a more comprehensive and accurate understanding of the neural processes underlying their research topic. To improve reliability and offer more insights, datasets from multiple sources at different time points can be integrated. However, there are significant problems associated with acquiring and integrating multimodal datasets [11].

The cost of acquiring data from multiple sources can be prohibitively expensive, and the process of collecting and combining the data can be time-consuming because it frequently necessitates the use of specialized equipment and software [12]. For instance, combining fMRI and EEG data [13] into a unified dataset requires knowledge of both data types and use of special software to analyze them. Interpreting the results of multimodal data can be challenging due to the complexity of the data and the potential for bias. This can lead for example to overfitting of the results, and therefore to incorrect conclusions. The

complexity of the data, as well as the need for specialized software, can cause a bottleneck in data processing and analysis.

Additionally, it can be difficult to accurately match data from different sources due to differences in formats, storage locations, scales, and resolutions (spatial et temporal) of the collected data (e.g., MRI, EEG, fMRI). There may be discrepancies between the data collected from different sources, making it difficult to integrate the data into a comprehensive benchmark dataset. The datasets and metadata may not be structured in a consistent way that allows for integration with other datasets or for the use of more sophisticated data analysis techniques. Labeling data points is often difficult and time-consuming, making it difficult to develop accurate models. As different complex measurements proliferate as routine parts of data collection, this problem will only increase.

Moreover, data are often acquired at several time points, for longitudinal assessments or due to time constraints among participants or to prevent volunteer fatigue [14]. Datasets gathered over several days are typically randomized or pseudo-randomized. This is particularly the case in some human projects where longitudinal studies with repeated assessments are conducted [15]. For such studies, the metadata should usually be provided as a set of documents available to download alongside the data itself. The metadata documentation, including the longitudinal and cross-sectional components of the study such as screening, follow-up measures, research teams who conducted the study, sample size and the age of the participant at the time of measurement, time intervals between the assessments, etc., is necessary. It is indeed important that metadata be informative about the dataset to be analyzed while following standardized ethical and quality measures. For instance, some projects examine pain chronicity by monitoring pain patients over a period of days or years and collect various data (such as MRIs and EEGs) and metadata (such as pain ratings, timing, or accuracy information) at multiple time points. Associations between data and metadata are made to establish relationships between, for example, neural alterations and pain variables in patients with chronic pain [16], or changes in pain chronicity and associated neural networks with time [17]. Such studies could not be performed without sufficient and reliable documentation of metadata.

Although many advances have been made regarding the organization, annotation, and description of research datasets, there is still much work to be done to ensure that datasets are fully standardized and can be accurately shared and reused [18]. For example, there are data standards such as Brain Imaging Data Structure (BIDS, https://bids.neuroimaging.io/) for neuroimaging data [19], EEG-BIDS for electroencephalography data [20], or MEG-BIDS for magnetoencephalography data [21], whereas standards for other data modalities (e.g., sensory testing, Ecological Momentary Assessments data (EMA)) do not exist yet. Particularly integrating behavioral data is challenging, as there is a lack of clear standards and ontology which allows us to generalize and thus group different behavioral paradigms (see section behavior data standardization). Finding data that can be potentially pooled remains challenging, let alone assurance that datasets are in standardized formats for meta-analyses by third parties. Strategies for applying Findable, Accessible, Interoperable, and Reusable (FAIR) [22] principles are still developing, and standard annotation systems and clear data identifiers are crucially needed.

Challenges due to diversity in acquisition, preprocessing, and analysis approaches

The availability of robust neuroscience resources such as high-performance computing (HPC) clusters [23-25], modern workflow technologies (e.g., Galaxy [26], Snakemake [27] etc.), cloud-enabled storage and computing infrastructures (e.g., Amazon AWS, Google Cloud [28]), secure databases [29], repositories [30], and analysis platforms are fundamentally changing how research in neuroscience is communicated and linked to existing raw data and findings [31]. Such tools are allowing researchers to utilize diverse techniques and produce massive amounts of high-dimensional data (large sample size, various models and conditions), which provides greater statistical power and the opportunity to do more robust secondary data analysis [32]. However, the data-driven neuroscience approach, as a whole, is questioned by a number of technical issues that must be addressed before it can be fully realized.

While the majority of collaborative research consortiums collect diverse multidimensional datasets, one of the primary challenges is that the majority of these datasets are typically inadequate for modern research methods and infrastructure [33] . Before committing to any of these tools for processing and analysis of collected datasets, it is important to understand data in terms of the number, volume, size and complexity of data, the types and formats of data, and the accuracy and completeness of the datasets. Despite similarities in experimental design, researchers in neuroscience experiments frequently organize and describe their data in their own way, even within the same research group. As a result, datasets collected from different research groups for a single project addressing the same scientific question may not be in formats suitable for comparison and pooling, limiting their interoperability and reuse. The data formats collected in each project are typically determined by the acquisition and measurement method, intermediate pre-processing, or analysis software. This can lead to data and metadata being stored in different locations.

The most pressing challenge we encountered was the lack of standardized preprocessing and analysis approaches [34]. For instance, running modern deep-learning neuroimage analysis tools requires more computing power, memory, and storage, and HPC clusters are available to provide these resources, dramatically accelerating the analysis process [35, 36]. However, it is challenging for non-experts to get easy access to these resources and perform scientific computing [23]. Especially for experimentalists, there is a fundamental need to provide succinct documentation on how to use these resources efficiently. To facilitate this, applications for image data processing must have APIs (application programming interfaces) that may be utilized without specialized coding knowledge. It is often suggested to use comparative analysis methods and two or more software packages to obtain reliable and reproducible research results. Developing such tools (e.g., bwVisu, https://www.bwvisu.de/) requires significant customization and software development costs, an investment which may not be possible for individual research labs. To overcome these obstacles, experimentalists, data managers, and computer scientists must work in a close, strategic partnership.

Even if datasets are imported into a common file format, data decisions are often influenced by the immediate analysis of interest, with no guarantee of compatibility between laboratories or even between projects within a laboratory. A wide variety of variable resources are utilized, ranging from open-source software for analysis to lab-tailored preprocessing workflows.

Research groups often use specific custom preprocessing pipelines that are developed internally to meet specifically a lab's current needs (e.g., python scripts to examine oscillatory frequencies associated with experimental pain, followed by proprietary software for statistical analysis or visualization). In the analysis stage, researchers must either write custom analysis scripts or spend time and effort converting datasets into supported formats to use publicly available tools for analysis. Various preprocessing or analysis software often result in different file inputs and outputs. These lab-specific custom workflows and pipelines usually prioritize internal needs over the needs of a broader community. Another general challenge while using lab-customized software or a single computer hardware solution (e.g., routine analysis scripts) is that they do not perform efficiently on large or complex datasets. In the case of workflows including third-party tools and software, there are issues of broken dependencies because of variability in computing. Problems with reproducibility [37] are made worse by the fact that the original analyses were done in different software environments, using different operating systems (e.g., Linux, Macintosh), and with different software or software versions. For example, variability in reproducing results from neuroimaging studies has been often investigated [38, 39]. Often, analyzing fMRI data with software packages such as SPM (Statistical Parametric Mapping) [40] or FMRIB Software Library (FSL) [41] might lead to different outcomes. Efforts are however being made to determine sources of variabilities and develop homogenous and standardized computing environments [42].

Metadata challenges

Another challenge is managing metadata (http://www.merriam-webster.com/dictionary/metadata), especially for complex, large, multi-site, heterogeneous datasets [43]. In a perfect scenario, all the metadata related to the acquired datasets would be readily accessible and sufficient for data sharing. In reality, they are not (yet). The associated metadata (such as the origin and type of a sample, experimental conditions, applied measurement techniques, devices used, calibration methods, and units) are frequently missing, incomplete, or only available in fragmentary form. In addition, crucial details like the accuracy and variability of the data points or the underlying structure of the data are often missing from datasets. Current datasets, for example, may lack the resolution, annotation, or labeling required for deep learning algorithms to be applied. In some instances, even if the metadata are available, they may lack the necessary tools to extract meaningful insights from the data. Knowledge of the dataset's quality and any accompanying metadata are becoming more and more crucial issues in terms of reproducibility [44, 45].

For most neuroimaging datasets, data annotation is highly essential. For example, when analyzing task-based data, the extent to which events are clearly documented determines an experiment's reproducibility.

Moreover, a laboratory can generate a large dataset from a single experiment or a single dataset from multiple experiments, and the collected metadata can be very complex and stored in multiple files with different formats only readable by the acquisition software or by customized codes written for internal use. In such cases, a consolidated strategy for unifying data into a single format that can be read by a variety of software applications and analyzed in an efficient and reproducible manner is necessary.

Furthermore, depending on individual lab practices, raw data and associated metadata may be distributed in different files or separate directories. It requires additional effort to read and extract the metadata from their original raw data files and integrate them into a single file. Interoperability between file formats can be a technical issue if the appropriate software to read, view, and process the files is no longer available. It is also possible that the format is no longer supported by any software, making it impossible to open the file. Research studies have also demonstrated that there is no single "best" way to process and analyze large-scale single or multimodal datasets. A neuroimaging study, for example, that presented the results of a survey of fMRI experiments revealed substantial differences in how individual labs preprocess and analyze their data, with 70 independent laboratories analyzing the same dataset and producing varying results [46]. This was further supported by another study, which showed how the analytical decisions of individual researchers can significantly affect the findings from an fMRI data set [47]. This emphasizes the potential implications of failing to have standardized pipelines for handling complex data and how it can impact research outcomes.

Data storage and volume

The data volume varies substantially depending on the data modality, ranging from a few megabytes (e.g., questionnaire data) to terabytes (e.g., high-resolution fluorescence imaging). Projects involving large amounts of data generated from high-resolution fluorescence imaging, volume electron microscopy, electrophysiology, or MRI can typically yield terabytes of data. Such data are often stored in dispersed locations and infrastructures in various formats (often proprietary), requiring a significant amount of time and effort to efficiently manage, utilize, and curate the data [48]. Researchers, particularly those working with high-dimensional data, require consistent support for data storage, timely backups, and archival systems. Inefficient data storage processes can lead to data integrity failures, accessibility issues, and increase in operational costs. Commercial cloud storage solutions are available, but they come with unreliable and slow backup and restoration services. They also have no obvious access paths or interfaces for easy migration onto analysis platforms, and have a questionable data protection of sensitive data (e.g. patients data) [49]. Many commercial cloud storage solutions are designed primarily for convenience and cost savings rather than for robust backup and restoration services. As a result, these services often lack the features necessary for reliable and fast backup and restoration, such as automated data backups, point-in-time recovery, and incremental backups. Additionally, many cloud storage solutions lack the necessary APIs, scripts, and tools that would allow for easy migration of data onto the analysis platforms. This makes data migration difficult and inefficient, and it can significantly slow down the analysis process.

Assuring access to secure and optimal storage solutions that can be integrated with workflows encompassing data acquisition, intermediate analysis, and archiving is thus a major challenge. Creating backups and storing multiple copies of large volumes of datasets, the need for collaborative and parallel access by multiple people, and use across a diverse range of computational workflows all pose major challenges to storage servers.

Challenges in data documentation

Data documentation presents a number of challenges, including the adoption of digital systems and laboratory inventory management systems for large consortia. Electronic laboratory notebooks are essential for data documentation (such as hypotheses, methods, observations, experimental protocols, notes, etc). Many efforts over the last years have recognized the critical need for institutional-wide adoption and implementation of an electronic laboratory notebook (ELN) [50].

For a large-scale neuroscience consortium spanning diverse experimental protocols, it is important to select an ELN that can provide comprehensive support for a wide range of experimental protocols and provides flexibility to add domain-specific features if required [51]. The initial challenge is to select an appropriate option that fits into the current laboratory standards. In addition, a usable and sustainable electronic laboratory notebook needs to be interoperable and incorporated into existing data workflows. There are obvious issues of user resistance; expensive costs involved in the implementation; secure configuration and maintenance; and the user will be ultimately responsible for managing the digital system.

There are several open-source and proprietary options available for use [52]. Often, for proprietary options, documentation may exist in the form of vendor's specifications or may be created and maintained within the context of a global community. It might not fulfill domain-specific requirements. A main functionality that could support easy documentation is sometimes missing, and the available features are secondary and not as beneficial to users. Often, there is no automated end-to-end solution that allows users to document experiments, which in turn makes this process time-consuming and tedious when performed manually.

When selecting an ELN, there are potential legal and data privacy concerns to consider [52]. An ELN is a digital resource which stores confidential information, and can be accessed by multiple users. To ensure legal and data privacy compliance, it is important to design ELNs compliant with all applicable laws, regulations and ethical standards. Additionally, it is important to consider the security measures in place for the ELN, including encryption and user authentication measures, to ensure that the stored data remains secure and private. Finally, it is important to consider the terms of service for the ELN, as this will outline how the data is used, stored and shared, as well as any limitations on the use of the data. By considering these potential legal and data privacy concerns, organizations can ensure that their data remain secure and compliant.

Data sharing and dissemination challenges

There are significant challenges in organizing datasets in a useful manner to enable sharing with collaborators. Even if a dedicated central data storage infrastructure is available, insufficient quality control measures as well as time constraints have a direct impact on data sharing practices. Especially in small research groups or individual projects, limited funding and sustainable resources directly impact the level of data sharing and reuse. Another significant issue is motivating researchers to share data publicly. Indeed, researchers are also hesitant about openly sharing their data due to the concern of possibly not receiving credit, or fear of reducing their own chances of performing secondary studies, or not having handled data sensitivity properly, and to avoid possible criticism about data quality. Despite

the fact that an increasing number of research organizations, academic journals, and large-scale projects are supporting extra efforts to build realistic data sharing techniques, this has not yet become a standard research practice [53].

Furthermore, while many journals require open data sharing and dataset submission to public repositories prior to manuscript submission, there is limited oversight on data sharing policies. Additionally, choosing a suitable public repository could be difficult for a number of reasons. Researchers should confirm that the repository complies with the research data regulations of their host institution before contributing datasets to open repositories. Finding a suitable subject-specific repository for a given dataset could be challenging. The alternative is to submit the data to a general-purpose repository, but there can be issues regarding data visibility as the particular repository might not be well-recognized in its field of research.

Another significant issue with submitting data to general repositories is that the latter might not have adequate support for certain types and formats of data [54, 55]. For example, if the data are in a non-standard format, the repository may not be able to process it correctly or even accept it. Additionally, repositories may not have specialized tools or services to help researchers convert, organize or analyze the data. This can be especially problematic for data that are highly specific, such as medical records or geospatial data. Without the necessary support, researchers may be unable to make full use of the data or even access them. Additionally, general repositories may not have the same level of curation and organization as a specialized repository, which can make it harder for researchers to evaluate the quality and relevance of the data. This can lead to a lack of reproducibility and increased difficulty in building on previous research. Therefore, it is recommended for researchers to submit their data to specialized repositories that are tailored to their specific field. Neuroscience specific repositories, on the other hand, are specifically designed to accommodate the unique needs of neuroscience data (openNeuro). They are often managed by experts in the field and have the necessary infrastructure to ensure the safe and secure storage of data. Furthermore, they often provide additional services such as data analysis, curation and visualization tools, which allow researchers to better understand and use the data.

Even after identifying a suitable repository, bureaucratic procedures and demands for publishing datasets in open data repositories require additional work, including converting files to the required format, compiling consent forms and contracts, removing sensitive information, and preparing documentation. Finally, maintenance funding must be taken into account because many repositories charge a fee based on the data volume. In the latter stages of the data lifecycle, these factors can hamper findability and reusability.

Challenges due to sensitive data

Projects involving human subject data or other sensitive data must maintain strict data privacy regulations for the storage, sharing of collected data and use for research purposes [56]. Sensitive data with potentially identifying information must be anonymized or pseudonymized prior to making the data public to protect participant confidentiality. Keeping such high ethical standards can be costly and time-

consuming, adding a further burden to researchers [57]. Long-term preservation and sharing of sensitive data largely depend on informed consent, data reuse agreements and policies, and also the type of archiving solution or data repository being used. Each step of handling sensitive data must protect privacy and identity protection rights, often through de-identification or anonymization. There are distinct sets of regulations for full anonymization versus de-identification of data [57]. It is therefore recommended to retain multiple versions of the data: one suitable for public release, and one suitable for further research but available on a highly restricted basis [58]. These considerations can lead to increased data duplication and storage needs.

Sharing of sensitive data between collaborators located in two different locations requires additional effort as data controllers need to make sure that data protection requirements are met in both the original location where the data were collected and the collaborator's location (https://www.imperial.ac.uk/research-and-innovation/support-for-staff/scholarly-communication/research-data-management/data-storage-and-security/storing-sensitive-and-personal-data/). Furthermore, external collaborations across universities can present logistical challenges in the form of access and security entitlements; these concerns are compounded when collection of sensitive data is part of the research project, or for collaborations with researchers embedded in clinical settings. Another major obstacle to sharing confidential data with external parties is the cost involved in adopting secure data sharing platforms and a major risk of participants being identified. In this context, researchers require consistent training and education that promotes responsible research conduct and adheres to institutional and discipline-specific data management policies (risks of data disclosure, confidentiality obligations, privacy principles, and network security).

RDM challenges for specific projects

Across the consortium, several projects presented specific challenges in RDM, either in organization and management, sheer data volume, logistics, or collaborative scale. In these cases, effective data management is integral to project success and may require custom strategies and resources. Below, we list examples representing the consortium extreme cases; we found this identification useful in determining the new developments required.

Challenges in behavioral experiments

The increasing number of collaborative studies may be hampered by challenges in standardizing behavioral experiments across laboratories (e.g., continuous animal movement recordings, mouse trajectories). There are no specific community data standards for storing behavioral datasets, which has a direct impact on data sharing. Also, research labs often do not have access to modern tools for extracting and analyzing behavior because their implementation usually requires advanced computational skills.

Another challenge that behavioral data present is reproducing experimental results because it is often difficult to replicate the exact same conditions in which the experiment was conducted across the laboratories or even for researchers within the same laboratory [59]. Even unifying features from a single behavioral experiment is difficult due to differences in environment, number of participants, and

experimental conditions [60]. It can be challenging to determine which features should be unified and which should be eliminated across all conditions. The community's methods have not yet been consolidated, and new algorithms transfer poorly between labs.

The lack of publicly available behavioral datasets with accurate annotations is a major impediment to benchmarking different algorithms [61]. This is largely due to the complexity of behavior patterns, which makes it difficult to accurately annotate data with consistent labels. Additionally, the time and resources needed to collect and label datasets for behavior analysis often make it prohibitively expensive for many labs. As a result, it is difficult to obtain high-quality datasets that are sufficiently large and diverse to accurately test and compare algorithms. To address these issues, it is essential to develop a centralized database for storing methods and experimental protocols of behavioral assays, parameters (e.g., sex, age, and strain of the animal, genotype, marking, testing conditions, etc.), data and metadata files generated in the task (e.g., behavioral responses and compressed video and audio files), as well as a common framework that supports further analysis and visualization [62].

Electrophysiology with high-density probes

A few of the animal projects in the consortium make use of new technologies such as high-density Neuropixels probes [63]. Neuropixels datasets are often large (~80 GB/hour) and computationally demanding, which can make it difficult to scale spike sorting workflows across different labs and datasets. Data storage requirements rise as a result of the significant amounts of derived data needed for intermediate processing (such as filtering and spike sorting) and stimulation and/or behavioral parameters (such as optogenetic stimulation, motion or whisker tracking, and task performance). Analysis and post-processing may often require computationally intensive algorithms and hardware acceleration to handle data that cannot be loaded into local memory [64]. Due to differences in recording conditions, spike sorting algorithms and data preprocessing, there can be significant variability in the results produced by different spike sorters. Real-time processing requirements for closed-loop experiments only serve to exacerbate these issues. Important parameters initially recorded from raw data, e.g., animal arousal/anesthesia level, impedance measurements, can be discarded in derived datasets used for analysis. Complex hierarchies of derived data and multimodal datasets (e.g., accelerometer, whisker or pupil tracking, etc.) collected with different instruments compound these issues. It can be challenging to validate and reproduce results, as the various algorithms and parameters used can produce different outcomes.

Large-scale in vivo two-photon calcium imaging

Some rodent projects within the consortium acquire large amounts of data collected over months [65]. For example, data acquisition using imaging techniques such as fluorescence imaging or two-photon microscopy calcium imaging (2P imaging) generates large sets of spatiotemporal imaging data (up to 100 GB/hour) and requires rigorous preprocessing steps (image segmentation, denoising, motion correction, manipulation and handling of large video files, and neural activity deconvolution) using high-throughput computing [66]. The downstream processing and analysis of the resulting datasets generated over the course of months is often challenging and requires complex workflows [67]. A few

open-source software solutions (CalmAn [68], EZCalcium [69], etc.) have been proposed to deal with these challenges. However, comparative analysis studies have revealed that the neural assemblies (collection of neurons that are activated simultaneously in response to a particular stimulus and form assemblies) recovered from 2P imaging datasets can vary significantly depending on the algorithm used, and that certain algorithms are more reliable and faster than others [70]. Specific algorithms have been found to have higher precision and longer run times than others, while others have been found to have faster run times but lower accuracy. Another issue is that many studies include synthetic or benchmarking datasets, but producing and analyzing these datasets requires challenging calculations, raising the computational complexity and costs. This enables the need for more scalable and fully automated workflows that can be run on HPC clusters [65], which requires ensuring the reproducibility of studies [71]. Existing software solutions can be used for analysis and visualization of datasets, but any adopted data and metadata standards must be interoperable with these tools.

Challenges associated with human-animal tandem projects

Projects collecting data from both human and animal models pose several challenges, such as systematic and parallel implementation of experimental designs, techniques, and analysis tools [72, 73]. Data management processes to create harmonized datasets and analysis workflows while establishing clear linkages between human and animal models are difficult and standards for integrating data are somewhat ad hoc. In collaborative projects involving multiple laboratories working on multiple species, the integration of data and analysis should happen systematically, not only sporadically. Apart from the sheer scale of such collaborations involving multiple research areas and the multimodal RDM issues discussed above, these tandem projects require a secure platform for data transfer between different sites (e.g., laboratories and clinics) with different security permissions and data handling standards [74].

II. Consortium-wide RDM implementation phase

The implementation of a RDM strategy for a large consortium is primarily based on the various types of data generated across research projects, as well as on practical methods for organizing and managing the data. Our priority was to adequately characterize the consortium's needs before committing to specific resources. One of the primary goals of identifying common requirements was to ensure that the best data management practices could be implemented across the consortium while taking individual lab practices into account.

Direct involvement with researchers during the planning and initial implementation phases was crucial to identify the most helpful RDM measures, these may be as simple as coordinating communication between core IT staff and researchers, and facilitating access to institutional or other pre-existing resources. We devoted significant time to initially gather information about publicly available tools and services that would be useful to the diverse projects within the consortium. Given the large number of laboratories from various institutions participating in the consortium, as well as the increasing number of requirement changes over the course of a project, information was gathered in a variety of ways

(virtual individual interviews with project PIs; discussions during online data seminars led by the CRC data manager; and personal meetings with experimentalists and PhD students). Data discussions and regular communication with consortium members have greatly aided our assessment approach. The PIs, or project responsible persons, were required to respond to a variety of data management questions as part of the assessment process (see Supplementary Information 2).
The assessment questionnaire was designed to obtain information from researchers about the major challenges they faced when managing data in their labs. The information gathered was used to implement common RDM solutions for individual CRC project groups, such as identifying and targeting data storage, organization, and sharing resources. The project-specific assessment included questions about the types of experimental models, data modalities and their acquisition methods and techniques, types of analysis tools and software, types of raw and intermediate file formats, workflows for pre-preprocessing and analyzing acquired data, export procedures for sharing and publishing datasets, etc. Additionally, we discussed challenges in publishing data and metadata in open data repositories.

In terms of research data and technological advancements, the survey response was extremely diverse. The majority of the consortium's researchers reported that sharing large-scale datasets with collaborators and efficiently curating this data from data archives and repositories after the project is finished were their most frequent challenges. The survey's findings enabled us to tailor the data management solutions to the common needs of the majority of projects.

The broad scientific goal of the Heidelberg Pain Consortium (https://www.sfb1158.de/) is to understand the mechanisms of pain and pain chronicity in order to identify causal links and possible therapeutic interventions. This involves a multidisciplinary team of scientists and clinicians working on 23 different projects, including 12 animal projects, 6 tandem (human-animal) projects, 4 human projects, and one central administrative project. CRC 1158 includes two service projects: the first one (tandem project) aims at establishing standard protocols, models, and ethical standards to facilitate homogeneous implementation across all human or rodent projects. The second service project (animal project) aims at developing simplified systems to accelerate the analysis of the translational potential of acquired research insights.

Assessment of data management requirements in the Heidelberg Pain Consortium

To identify common RDM measures, we examined the commonalities between all projects, such as the type of population studied (rodents, humans, or tandem), followed by the type of data modalities acquired (e.g., neurophysiology, neuroimaging, and behavior). Human projects include data collected from both healthy individuals and patients with various clinical conditions (e.g., chronic back pain, severe depression, diabetic neuropathy etc.) and Rodent projects utilize mice as animal models (Figure 1 A).

We further categorized projects into subgroups based on common data modalities that were being acquired. Figure 1B depicts an overview of the various data types collected across the consortium

projects. Neurophysiology data (including electrophysiology and cellular physiology) are the most frequent data category collected across all studies (i.e., 83% for animal and tandem projects and 100% for human projects). Imaging, behavioral, and genetic data are collected in similar proportions in human projects (75%), whereas psychometric data are collected by all human projects. In 41.2% of the animal projects and 83% of the tandem projects, imaging data are collected. Behavioral data (e.g., various pain models) are collected in 58% of animal projects and 66.67% of tandem projects.

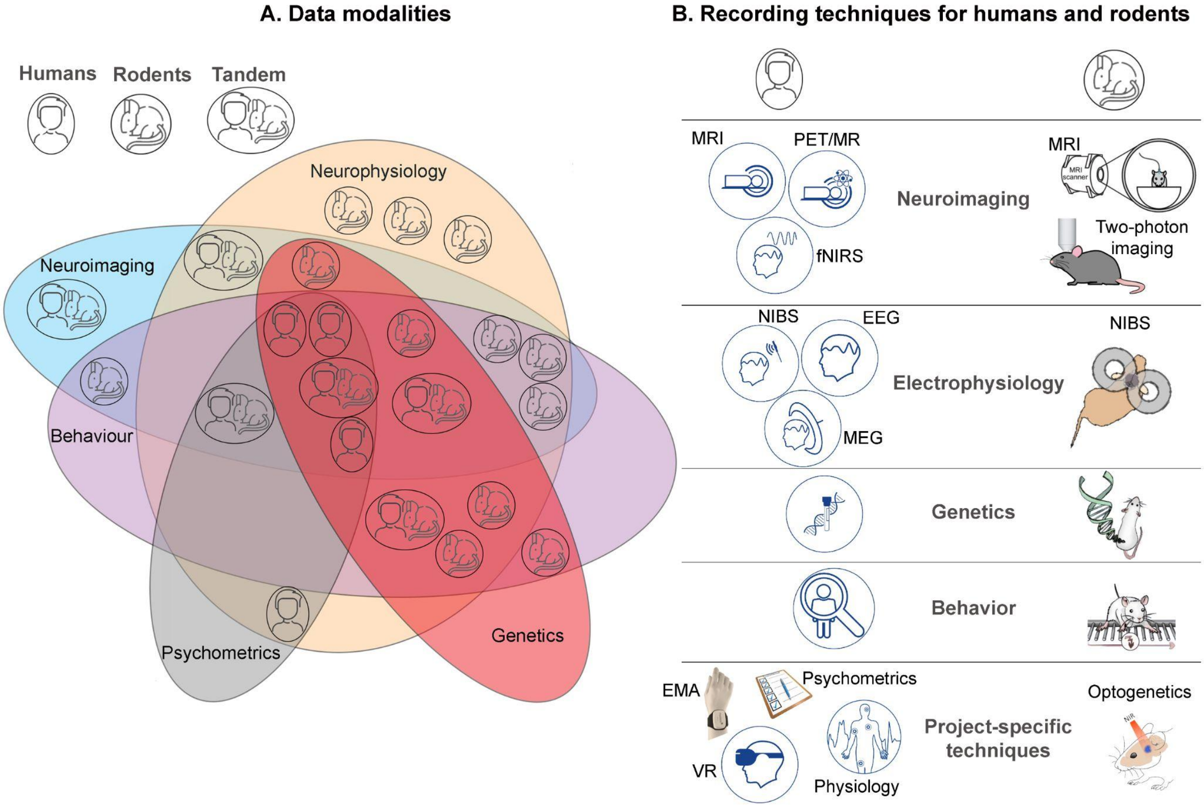

Figure 1: The Heidelberg Pain Consortium investigates various populations: humans, rodents, and tandem using various modalities: neuroimaging, neurophysiology, behavior, psychometrics, and genetics (A). Each data modality can be recorded using different techniques: MRI= magnetic resonance imaging; PET/MR: positron emission tomography/magnetic resonance; fNIRS: functional near infrared spectroscopy; NIBS: non-invasive brain stimulations; EEG: electroencephalography; MEG: magnetoencephalography; Genetics; Two-photon imaging; Behavior; EMA: ecological momentary assessment; psychometrics; physiology; VR: virtual reality; optogenetics (B).

Our consortium includes research projects that typically collect data from a wide range of methods and techniques (Figure 1B), such as electrophysiology, neuroimaging, extracellular and intracellular signals, and two-photon imaging for rodents; behavior (including stress and fear assessment in humans and rodents); and multi-omics (genetics) datasets. Rodent and human projects use comparable methods, such as magnetic resonance imaging (MRI) of both the brain and peripheral nerves; electrophysiology,

including electroencephalography (EEG) and magnetoencephalography (MEG) for humans; extra- and intracellular signals and two-photon imaging for rodents; and brain stimulation methods, including transcranial magnetic stimulation (TMS) and transcranial electrical stimulation (tES). Additional specific methods in humans are electronic diaries (EMA), peripheral physiology (e.g., heart rate, blood pressure, sensory profiles); virtual reality, psychometrics; daily assessments of psychological methods such as ecological momentary assessment (EMA). Specific methods for rodents include optogenetics.

We gathered information on optimal storage solutions for projects, and the response was diverse as there were projects acquiring large numbers of datasets (e.g., ranging from ~5 TB/day to 1 petabytes), whereas other projects acquired relatively smaller datasets (e.g., a few gigabytes per month). For instance, some projects involved continuous recording of neurophysiology datasets from high-density probes for a few days or a week at a time, which can generate up to 100 TBs of data. We documented that 80% of the projects were already utilizing university infrastructure for data storage, whereas many human projects utilize individual lab servers.

We then collected information about file formats utilized for collecting and preprocessing raw data from different acquisition systems and a wide range of methods. Given the complexity and diversity of experiments and the varied volume of data collected in the consortium, the acquired file formats are most often highly specific to certain data types (such as time series, e.g., voltage traces, image stacks, stimuli or behavior) or acquisition or recording device. Several projects require the development of new tools and software for migration to open data standards, resulting in the need for additional resources and support from the CRC.

Electrophysiology experiments, equipment, and analysis pipelines, in particular, are customized for each project and generate data in a variety of file formats. Data are collected using various techniques and experimental designs, such as patch-clamp to tetrodes in freely moving animals and high-density silicon probe recordings. The steps for preprocessing for intracellular, juxtacellular, and extracellular techniques are frequently customized. Any measures to standardize must be compatible with existing lab analysis tools and data processing methods. Despite the fact that a number of community-developed electrophysiology metadata and data standards are available and evolving, they have not yet been widely adopted.

We also collected information about the most common preprocessing and analysis softwares (e.g., IgorPro (https://www.wavemetrics.com/products/igorpro), ImageJ [75], Matlab, etc.) utilized across different projects. Our assessment also included information regarding projects using electronic lab notebooks and those using traditional handwritten notebooks. Additionally, we assessed the definition of user permission for data access, protocols for data sharing, short- and long-term storage needs, and implementation costs.

RDM Communication and Exchange

Several neuroscience-specific RDM solutions already exist, ranging from software and infrastructures for streamlining data collection and acquisition protocols, collaborative data analysis and visualization packages, to data sharing and archiving platforms [58, 76-78]. Our initial observation while implementing RDM strategies was that many researchers were not aware of the benefits of existing

resources, partly due to uncertainties regarding the bureaucratic procedures, the General Data Protection Regulations (GDPR), and more often, the technical requirements for easy integration of these resources into existing laboratory practices [79, 80]. Therefore, we put great emphasis on promoting and encouraging the use of pre-existing resources that meet the needs of our consortium or that help in a particular use case. An important aspect was to find a balanced approach that encourages an appropriate degree of integration of existing resources with realistic domain specificity. We curated a list of both generic and neuroscience-specific RDM resources, both on the consortium/institutional (internal) and national and international (external) levels. The list can be accessed in the data management section of our CRC website (https://sfb1158.de/index.php/rdm-resources).

Data infrastructure (platforms for storage, organization, analysis and sharing of data)

Technical infrastructure was made available to consortium members based on the individual CRC project's needs and demands. We aimed for simple and efficient solutions for secure data transfer between collaborators with controlled access, all while balancing ease of access for research. Each of these services and their underlying technology, properties (e.g., sharing possibilities, availability on HPC, backup, versioning, access), technological foundation, and usage scenario are explained in the next section.

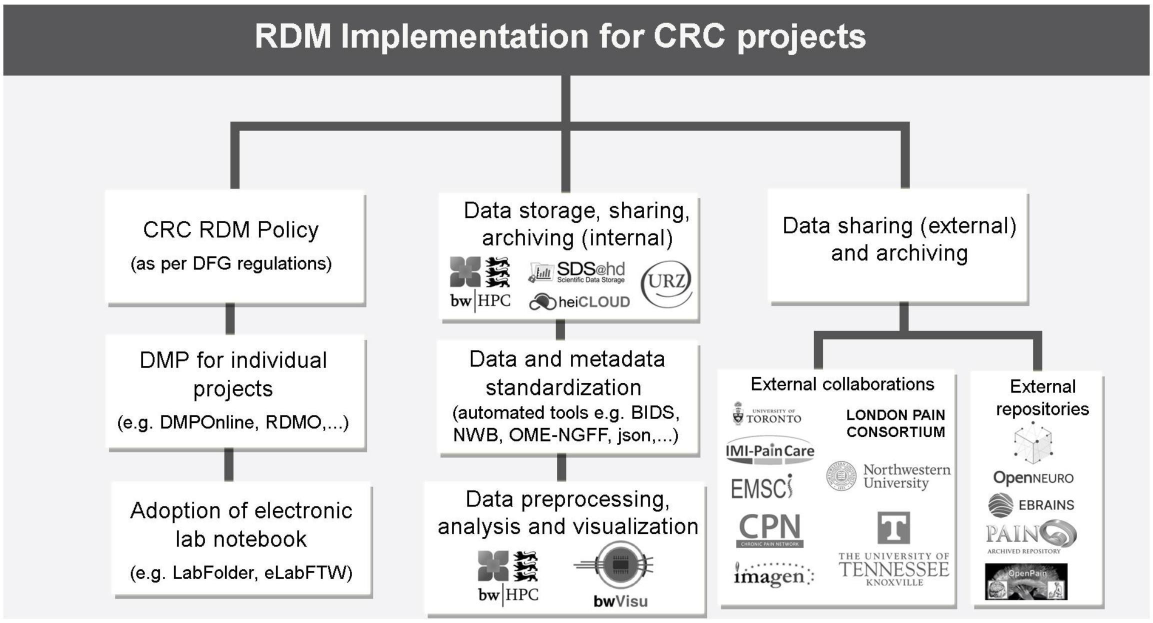

Figure 2: Schematic of the data management implementation for CRC projects.
RDM: research data management; DMP: data management plan.

Data Management Plans for CRC projects

We designed three DMP templates after defining and categorizing the data management needs for each project depending on its experimental model type: human, animal, and human-animal tandem

(see supplementary information 3.1, 3.2, and 3.3, respectively). The templates can also be found on Zenodo: https://doi.org/10.5281/zenodo.4410128), [81]. DMP templates for animal, human, and tandem projects can differ depending on the scope of the project, the type of data collected, and how the data will be managed, but the major difference is the ethical considerations associated with each type. Animal projects, for instance, may necessitate additional safety protocols for the storage and management of animal tissue samples, whereas human projects require more stringent regulations and oversight for the ethical processing of human data [82]. Additionally, DMPs for human projects may include the collection of sensitive data (personally identifiable information), which must be securely stored and shared according to regulations. The DMP should follow General Data Protection Regulation (GDPR) -compliant guidelines for handling of personal data and specify information, authentication measures for reuse and recovery, de-identification of datasets involving human participants before sharing, and publication process for anonymized data. Human-animal tandem projects are more concerned with resource allocation and special protocols for the integration of different data types collected from multiple sources. It is important to ensure accuracy and consistency across all sources. The DMP states minimum requirements for metadata that must be provided for long-term preservation and secondary analysis of research data. It also contains the information about data migration and access by third parties or future collaborators even after the project ends.

Data storage solutions

Projects in the CRC generate massive volumes of data of various types and rely on data interoperability among labs. It is strongly advised that researchers securely store full datasets (e.g. raw, preprocessed, and analysis files, codes, etc.) associated with published findings and results, as this promotes the consortium's goal of further engaging in open science.

The CRC provides support for various storage solutions for all stages of the data life cycle, and the best option for a research project is determined by the type of data being collected, the size of the data, the security requirements, and the scalability of the platform. Factors such as interoperability with the existing infrastructure of the project group, reliability and accessibility for a particular storage solution, and availability of user-training and service support are also taken into consideration. For example, if the research project involves collecting large amounts of data, then a cloud-based storage platform may be the best option. Additionally, cloud-based storage platforms provide access to data from anywhere with an internet connection, making it easy for researchers to collaborate and share data with colleagues. If the data is sensitive, then a secure, on-premise storage solution may be the best choice because it can be customized to meet the specific security needs of the organization, such as encryption, authentication, and access control. Additionally, if the research project (e.g., projects collecting terabytes of data from methods such as optogenetics, electrophysiology, and calcium imaging etc.) requires scalability, then a platform that can easily scale up or down may be the best option. Ultimately, the best data storage platform for a research project will depend on the specific needs of the project, and university-approved data storage services (see Figure 2) are recommended to guarantee data privacy and confidentiality.

Most of the CRC researchers are encouraged to use our internal data storage platform SDS@hd (SDS, Scientific Data Storage, with a capacity of 20 PetaByte), a central service for securely storing scientific data https://www.urz.uni-heidelberg.de/en/service-catalogue/storage/sdshd-scientific-data-storage . To facilitate easy data sharing between internal collaborators, all datasets collected from rodent experiments in collaborative projects are shared across different research teams via SDS@hd, which stores experimental protocols, raw and pre-processed data, session, and mouse information (e.g., session start time, animal weight, etc.), task files (e.g., behavioral responses, videos, audio files), acquisition metadata (e.g., dimensions, pixel types, and instrumentation settings), in a shared directory structure for project members. It is intended to be used for data that are frequently accessed ('hot data'). A common storage place ("Speichervorhaben" (SV) meaning data storage projects) is requested for collaborative projects or work groups to ensure that data are easily accessible to all project members with proper authentications (university credentials). The Heidelberg University network connects the consortium's labs and university departments, using a 10 GB capacity network to expedite data transfer among institutes and facilities. Using SDS@hd drastically increases the ease of data access for shared projects and data safety by virtue of automated mirroring for data backup. Datasets collected from confocal or two-photon microscopes, for example, are stored on acquisition computers for a few days until they are stored on to SDS@hd. Once transferred to SDS@hd, individual users must ensure that the large datasets are timely (usually in few days or a week depending on data volume) removed from acquisition systems to free up the imaging acquisition system for new data or for another user.

Apart from its storage and fast data transfer capacity, another potential reason for storing large datasets on SDS@hd is its direct access to other university platforms such as HPC systems (explained in next section). Other university storage solutions that can be requested via support from CRC data manager include a SERVER BACKUP (https://www.urz.uni-heidelberg.de/en/service-catalogue/storage/server-backup, service for data storage for servers (based on a data protection and recovery software i.e. IBM Spectrum Protect (ISP) https://www.ibm.com/support/pages/overview-ibm-spectrum-protect-supported-operating-systems), CLIENT BACKUP service (https://www.urz.uni-heidelberg.de/en/service-catalogue/storage/client-backup (can be access via on all operating systems via Duplicati software) for secure storage of workstations and PCs, and HEIVOL-I (https://www.urz.uni-heidelberg.de/en/service-catalogue/storage/heivol-i), a service for creating network drives for university institutes and facilities. Similar services are available at the other participating CRC labs from other institutions.

For human data storage (including sensitive data), researchers use a storage server with restricted access (Dell Isilon server with a large storage and archival capacity). Designated personnel have the authority and responsibility to enable access to internal collaborators. When necessary, access can be given to external collaborators by assigning guest accounts with a data sharing agreement in place.

Data processing, analysis, and visualization

Several projects and laboratories in the consortium use laboratory-based analysis infrastructure, such as local computers, shared analysis workstations (laboratory computers with GPUs and pre-installed acquisition and analysis tools that are shared by several members), and computational servers that are run by individual laboratories or groups.

The CRC highlights the importance of keeping track of every step, from initial data recording to the analysis, and proper documentation of analysis code, pipelines, and scripts. As a constructive starting point, the CRC 1158 data manager has set up a dedicated code space on Github (https://github.com/CRC 1158RDM) where multiple repositories with analysis code and scripts can be hosted and shared for each CRC 1158 project. The CRC 1158's data management organization repositories are maintained by the data manager, and access is given only to the authorized project members.

In addition to local infrastructure and computing servers, there is university infrastructure available for more demanding data processing tasks, such as running computationally intensive analyses of heterogeneous and large-scale imaging datasets collected from humans and rodent projects. An application for access to these services can be made by individual laboratories with an initial application and usage support from the data manager. To seamlessly integrate the data analysis with the setup and execution of preclinical experiments, a platform such as the one proposed here was necessary. Some of the CRC projects are utilizing bwForCluster MLS&WISO (https://www.urz.uni-heidelberg.de/de/service-katalog/hochleistungsrechnen/bwforcluster-mlswiso) and a detailed tutorial on access and use is made available (see https://github.com/SFB1158RDM/HPCtutorial). This eliminates administrative and technical barriers to performing computationally intensive tasks such as large-scale modelling, simulation, and analysis projects, e.g., Neuropixels systems. The HPC allows job scheduling using SLURM (https://slurm.schedmd.com/documentation.html) and also sets up reproducible computing environments (e.g, Docker [83], Singularity [84]) to optionally run the modules on the HPC for particularly large data sets that are streamed directly to SDS@hd during acquisition, e.g., chronic recordings with dense electrode array or image segmentation for chronic imaging (miniscope) with the perspective of running standardized analysis workflows. Allowing users to access large datasets stored in SDS@hd without downloading them to their local computer aids in the seamless integration of data analysis procedures, saving overall computational time and costs. The data can be accessed from the bwHPC cluster using the same protocols as for local storage, such as NFS, SMB, and FTP. This service also allows users to access data stored in SDS@hd from multiple bwHPC nodes simultaneously, thus increasing overall speed of data access. By utilizing direct data access, scientists are able to take advantage of the increased computing power available in HPC systems and gain access to data stored in a scientific data storage system.
While writing this manuscript, bwForCluster Helix https://www.urz.uni-heidelberg.de/de/service-katalog/hochleistungsrechnen/bwforcluster-helix, a successor of the current HPC system, was made available to users. The Helix component will enable the use of seamless, cross-system workflows for processing and analysis of large amounts of data.

In our consortium's case, data management efforts had led to an increased user base for the HPC machines. Many factors played into a user's decision to use a particular HPC machine, such as its performance, cost, and availability. Data management efforts had made the HPC machines more attractive to users by providing individual support and training for access and use. The amount of training necessary to promote the use of HPC machines in a research lab depended on the particular needs and existing infrastructure. Generally, training and seminars covered topics such as high-level usage of programming languages, high-performance computing paradigms, and best practices for using HPC machines for neuroscience data processing and analysis. It also included guidance on how to design and optimize applications for HPC systems (e.g., refactoring of tools for direct access or use on HPC and bwVISU). A lab might also need to provide additional training for data management and analysis, or for using specific software packages.

Similarly, for processing massive (e.g., neuroimaging) datasets, some projects are utilizing the heiCLOUD (https://heicloud.uni-heidelberg.de/), an Infrastructure-as-a-Service (IaaS) cloud service that provides virtual machines that may be customized and utilized as needed for the project. A possible scenario for heiCLOUD usage within our consortium is to install complex and computationally expensive software packages and perform concurrent processing of massive neuroimaging datasets. This provides powerful workstations for data analysis and can be especially useful for collaborative research projects that require data sharing between multiple research teams.

Another service that is frequently accessed by CRC members is heiBOX (https://www.urz.uni-heidelberg.de/de/service-katalog/collaboration-und-digitale-lehre/heibox), a secure Sync and share service hosted on heiCLOUD. This service is similar to commercial cloud storage services like Dropbox and Google Drive, and allows users to save, synchronize, share, publish, and jointly edit files. heiBOX is based on the Seafile software (https://www.seafile.com/en/home/), and allows users to search for text files, PDF files, and Office files in unencrypted libraries using a full-text search. Additionally, Office files can be edited by multiple people, and files and folders can be tagged and commented on. Markdown documents can be used to create private or public wikis. heiBOX also provides backup, synchronization, and storage of small research data and document files, and guest accounts can be requested for data exchange with external collaborators. The most common use of heiBOX in our consortium is to share documents such as CRC meetings notes, data seminar and workshops presentations, individual projects DMPs, manuscripts and figures.

Another application that some of the CRC projects are utilizing is bwVISU (https://www.bwvisu.de/), a remote service for scientists (universities in Baden-Württemberg state, Germany), as well as the corresponding software stack to deploy such a service on-premises. It has an interactive web front-end that supports large-scale data analysis and visualization without much human intervention. Our RDM services also include providing technical assistance with the refactoring of lab-customized preprocessing analysis pipelines (MATLAB and Python scripts) into more organized workflows/Graphical User Interface (GUI) that can be run on HPC applications.

Data and metadata documentation and standardization

To encourage standardization of datasets generated within the collaborative studies across the consortium, we focused on data documentation as a key first step. We encouraged consortium-wide adoption of electronic lab notebooks (ELN) to help researchers document experimental protocols in well-annotated electronic form at an early stage of the research project [85]. We initially tested multiple available ELN options, both licensed and open-source, in order to select an appropriate option for our consortium projects and individual lab requirements. We also considered several factors, such as licensing options, security aspects, implementation and maintenance costs, ease of access and integration with existing resources, and other domain-specific features that may be required. Based on these factors, we selected two options: elabFTW (https://www.elabftw.net/) [86] and Labfolder (https://www.labfolder.com/). The Competence Centre for Research Data (Kompetenzzentrum Forschungsdaten, KFD), a joint institution run by the Library and Computing Centre at the University of Heidelberg, established a web-based instance of elabFTW with a fully encrypted service and data backed up on university servers: (https://www.urz.uni-heidelberg.de/en/service-catalogue/software-and-applications/elabftw) (https://elabftw.uni-heidelberg.de/login.php).

Data standardization in human projects

All human research projects acquire multimodal data (neuroimaging, neurophysiological, behavior, psychometrics, etc.). Within the plan of implementing good practices for data management, we followed recent developments in data standards and methodologies to make data interoperable.

For this purpose, we created some standard protocols for each data type, along with the associated metadata. For instance, for some standard acquisition of MRI data, we developed standard MR acquisition protocols for anatomical and functional scans in terms of image resolution, duration, and type of acquisition (e.g., resting-state or task-based functional MRI). The use of same acquisition parameters with consistent terminologies across studies allowed researchers to use similar preprocessing and analysis pipelines, promoting reproducibility and efficiency. Such homogeneity in data acquisition enables also the pooling of data across projects, which is particularly suitable to increase sample size or for comparison purposes. For example, we can directly compare the structure and function of various patient populations acquired in different projects, e.g., to examine commonalities between individuals with chronic back pain and fibromyalgia patients.

Our goals of data integration and homogeneity were facilitated by the recent opening of the Center for Innovative Psychiatric and Psychotherapeutic Research, CIPP (https://www.zi-mannheim.de/en/research/zipp-e.html), an extensive, modern research infrastructure with access to neuroimaging, pharmacological, and psychotherapeutic techniques. In this center, the researchers share the laboratories and equipment, which allows the collection of homogenous data types and data formats for behavioral (e.g., motor) or sensory (e.g., quantitative sensory testing). In addition, we set up a core set of standardized assessments (e.g., motor paradigms, the use of electronic diaries for pain assessments, quantitative sensory testing, stress-induced analgesia), and psychological questionnaires (e.g., HADS, MPI [87, 88]) to be used across all relevant studies.

We have made significant progress in clinical projects involving human studies by using the Brain Imaging Data Structure (BIDS) (http://bids.neuroimaging.io) data standard for anonymization, organization, and annotation of neuroimaging and behavioral data [19, 89]. BIDS also includes support for other multimodal data, longitudinal and multi-session studies, and physiological metadata collected during MRI experiments. In addition, using BIDS standards for the organization of MRI data enabled a consistent metadata structure, minimizing the number of manual inputs of metadata required and, hence, reducing the number of errors arising from misinterpretation of those inputs. For example, a typical MR brain acquisition includes numerous different protocols, such as anatomical and functional. In BIDS, metadata fields common across all subjects are specified in a single JavaScript Object Notation (JSON) file in the root directory instead of multiple files repeated for each subject. By organizing the data in this way, researchers can easily and quickly access the relevant information without having to manually input the metadata. Moreover, adoption of BIDS enabled the development of workflows for automated data extraction, curation and labeling [90]. For example, automatic extraction of a minimal set of BIDS compatible metadata can be performed using dcm2niix (https://www.nitrc.org/projects/dcm2nii/).

Regarding data storage, a secure storage server is used for anonymized data in accordance with accepted ethical and quality standards to maintain data protection and privacy. The original sensitive data are stored separately with restricted access to reduce the risk of disclosure or unauthorized access. The anonymized data to be analyzed are then uploaded to the laboratory server. The server is used as a shared infrastructure where a set of open-source software, e.g., freesurfer (https://surfer.nmr.mgh.harvard.edu/), FSL (https://fsl.fmrib.ox.ac.uk/), fMRIPrep (https://fmriprep.org/), DMRIPrep (https://www.nitrc.org/projects/dtiprep/) are installed for data preprocessing and analysis. We are using custom scripts for the anonymization of MRI datasets. Currently, these scripts are written in Matlab but we envision developing a modular automated tool with an interactive GUI. The custom codes used for anonymization, preprocessing, and analysis are available online via GitHub and released under the BSD license (https://github.com/SFB1158RDM/SFB1158_MRHuman). The resulting datasets in their BIDS format can also be validated using BIDS-Validator (open source code available at GitHub: https://github.com/INCF/bids-validator and the online tool available at: https://bids-standard.github.io/bids-validator/). After anonymization and quality control, the datasets are available for sharing within and outside of the laboratory. The data can also be made publicly available with proper security measures.

Data standardization in rodent projects

In order to devise practical solutions for harmonizing diverse neurophysiology datasets with varying file formats, we divided our standardization approach into three main goals: 1) consistent metadata documentation; and 2) adoption of open data standards, and 3) running similar pre-processing and analysis workflows HPC environment.

We developed standardized metadata templates that can be used to document animal electrophysiology and optophysiology datasets collected across the consortium projects. A web-based

GUI was designed in collaboration with (https://www.catalystneuro.com/, and the source code and installation guide are available for use at https://github.com/catalystneuro/heidelberg-metadata-gui. The metadata handling GUI allows standardized documentation of metadata (experimental information, acquisition and analytical parameters etc.) collected from neurophysiology experiments [91].

An initial set of JSON-schema is made available for a few electrophysiology experimental types, namely extracellular electrophysiology and optical physiology. These templates use ontologies (structured and controlled vocabularies) that can be used to describe the data and its associated metadata and include fields such as the type of animal, type of data collected, date of the experiment, duration of the experiment, type of equipment used, and any other relevant information. This will help ensure that the metadata is consistent across datasets and can be easily understood by researchers.

To expedite routine metadata entry, additional default field values can be set up for each laboratory. The GUI uses key metadata fields and possible values sourced from the consortium and stored in a centralized location (a data dictionary). The GUI enables export of metadata in JSON file format detailing the experiment once the experimenter has entered at least a minimal amount of metadata. The parameters for preprocessing procedures, such as spike-sorting or filtering, or machine-readable information provided in raw data files can be combined with JSON files, which can be saved locally or centrally.

The resulting metadata JSON files containing standardized metadata (all types of metadata: basic experimental details, acquisition settings, parameters, and analysis parameters are incorporated) are imported in electronic lab notebooks (e.g., eLabFTW) and also used for submission of complete datasets into open data repositories and archiving later in the research process.

Standardizing electrophysiology datasets recorded with Neuropixel probes involves the use of an open source acquisition system (Neuralynx system, https://neuralynx.com/) and open source acquisition software (SpikeGLX [63] (https://github.com/billkarsh/SpikeGLX) and Open Ephys [92]). Some electrophysiology projects within the consortium focus on using NWB (Neurodata Without Borders) [93] format, It is a HDF5-based format (Hierarchical Data Format version 5) and organizes files in a hierarchical structure and contains metadata, data, and processing code. NWB 2.0 supports a wide range of data modalities, including electrophysiology (extracellular recordings, intracellular recordings, and electrocorticography) and optophysiology (2-photon imaging, fluorescent wide-field images, and so on). The NWB 2.0 format contains all of the metadata (for example, voltage has a sampling rate and is connected to electrodes) that is required to specify the neurophysiology experiments parameters, and the data can be shared between labs in a fully standardized format.

Another useful strategy was the use of open-source analysis and visualization software packages such as SpikeInterface (https://spikeinterface.readthedocs.io) [94, 95], which supports import and export of data in NWB format. It elegantly solves the problem of importing hardware-specific acquisition formats into a common environment while also providing preprocessing capabilities and streamlined access to a variety of spike-sorting algorithms. The best way to ensure accuracy and reliability in the results from different spike sorters when looking at the neuropixel probes is to use a consistent, well-defined analysis workflow. Using HPC clusters and remote visualization platforms (e.g., bwVisu), it was possible to overcome the challenge of real-time processing of large electrophysiology datasets from multiple

recordings. Running similar pre-processing and analysis workflows in a single HPC environment allows for efficient data processing and ensures that the results are consistent and reproducible.

Additional assessment techniques in animals include electroencephalography (EEG), multiple modalities of magnetic resonance imaging (MRI), and positron emission tomography (PET). The datasets generated by microscopic imaging techniques and from a variety of acquisition devices, such as the repetitive in vivo multiphoton imaging experiments conducted over a 20-24 week period in living mice, are challenging to standardize. Researchers collect and view microscopic imaging data from different vendor specific acquisition software in varying file formats (e.g., TIFF, multi-page TIFF, Nikon ND2, Leica LIF, Leica CZI or ZVI etc). It is often difficult to read metadata from these files in other softwares. While TIFF is the most commonly used file format because it is easily accessible by many current analysis software platforms, it has some limitations such as long latency and delayed data access while working with large batches of files [96].

Our standardization strategy focuses primarily on tools that are interoperable with pre-existing services such as local data storage platforms used for storing imaging datasets, bioimaging software applications (ImageJ/Fiji, etc.) [75, 97] utilized for analysis of data, the type of ELNs adopted within the consortium labs, and the use of HPC clusters. The objective is to create a feasible level of automatic interoperability with existing data analysis and visualization tools as well as ELNs. We utilize existing bioimaging application Fiji that supports the import and export of multiple imaging acquisition file formats, and allows automated extraction and display of metadata from raw files (e.g., .TIFF, .LIFF) using its own Bio-Formats plugin (https://imagej.net/formats/bio-formats, including Bio-Formats Importer and Exporter, Bio-Formats Macro Extensions, Data Browser etc.). We have not yet decided to use one single format across the imaging projects but evaluating these community proposed formats will enable us to implement solutions that will allows users to link the experimental metadata (design protocols, biological methods, etc.) with microscope specifications, image acquisition settings, and analysis workflows in a more comprehensive metadata file (JSON or OME-XML format (Open microscopy environment-XML)) [98]. We are currently setting up a modular pipeline for exporting the data into more open-source and standardized formats such as a next-generation file format OME-NGFF [99] and Microscopy-BIDS format (an extension to BIDS for microscopic imaging data) [100]. Our standardization pipeline will also allow the incorporation of missing metadata values and will allow users to create more fields in order to support the various other acquired file formats.

Behavioral data standardization

To facilitate some level of behavioral data harmonization within CRC, we have adopted a very simple and intuitive approach. Numerous studies from the past have shown that adopting standard operating procedures and standardizing experimental conditions across labs for multisite, large-scale projects led to more accurate and reproducible results [101-105]. All rodent projects adhere to standardized experimental procedures for behavior assays across all models. We strongly encourage each project to share its Standard Operating Protocols (SOPs), experimental site conditions, hardware, software,

acquisition software, and preprocessing and analysis pipelines. To ensure consistency, we have provided SOPs to control variables such as mouse strain, age, and weight range. We have adopted a simple approach for storing any metadata for behavior datasets. For projects that combine behavioral data with any other type of experimental data (e.g., electrophysiological recordings or neuroimaging), we prioritize using the same metadata file format (e.g., JSON, XML) that is incorporated in the adopted data standard (e.g., NWB, BIDS) for other data types. This helps to define parameters for behavioral paradigms and facilitates the integration of datasets. Although this approach is not fully automated, it does provide an initial level of data documentation, which will help to promote further standardization [61].

Acquisition of standard human behavioral data has been facilitated by a service project aiming at training researchers and homogenizing acquisition protocols. Moreover, the CIPP infrastructure enabled researchers to collect homogenous data, acquired using similar equipment, resulting in similar formats. For example, quantitative sensory testing experiments have been standardized across projects in terms of measured variables, and output saved as .csv files. However, some projects collect additionally data which are specific to their patient population and therefore do not have any standards yet (e.g., defining body markers that trigger referred sensations or defining modality (i.e., sensory, motor) that evokes phantom pain in amputees). Additionally, framerates or resolutions of videos recording tracking data during virtual reality experiments are also project-specific and should be documented.

Data dissemination in CRC

Our consortium's collaborations with national and international neuroscience initiatives (e.g., NFDI4BIOIMAGE, EBRAINS) promote data sharing and encourages all projects to share large datasets (such as electrophysiological datasets from Neuropixels, cellular imaging datasets from preclinical projects, etc.) with external collaborators from the international community. Similarly, data harmonization efforts significantly aid in the sharing of large human imaging datasets in open data repositories.

Consortium's data policy encourages the submission of published datasets to repositories and the publication of open-access articles. Unless specifically exempted datasets, all consortium research data must be made available via a suitable data publishing or archiving platform under appropriate authorization and licensing (for example, a creative commons or open source initiative-approved (software) license) to allow for flexible public reuse. Any third-party data gathered by or provided for consortium research activities are equally subject to these standards, unless data use agreements clearly restrict it. We assist consortium members in archiving and publishing data on heiDATA (https://heidata.uni-heidelberg.de), an institutional repository for research data based on the DataVerse Project (https://dataverse.org/) [106, 107]. This repository supports data documentation as well as administrative, technical, and descriptive metadata; each dataset is given a persistent identification, a citable address, and a DataCite ID (Brase, 2010). In addition to data publication, the heiDATA repository allows data access via a simple interface. This provides for the permanent publication of data records in the repository while also providing a separate interface for regular data access. Complete datasets

are collected in a dataverse established for CRC 1158 projects (research data, code, documentation, and metadata). The dataverse is available at https://heidata.uni-heidelberg.de/dataverse/data-sfb1158. The Research Data Competence Center (KFD) also provides specific guidelines and procedures on data repositories, archiving, licensing, and access restrictions in order to provide public access to these datasets. The KFD is currently developing heiARCHIVE (https://heiarchive.uni-heidelberg.de/de/node/1), a digital long-term archive for research data preservation and archiving, which will be available to the CRC 1158 during the next funding period or near the end of the current funding period (https://doi.org/10.11588/heibooks.979.c13740; https://doi.org/10.11588/heidok.00029723). This service will provide researchers with an easy-to-use end-user platform for archiving their research data (at least for 10 years), as well as the option of performing OAIS-compatible long-term preservation with features such as format recognition, validation, and file conversion of appropriate file formats.

DISCUSSION

Open science and data sharing are increasingly being promoted by funding organizations and research groups. However, Individual scientists often find it challenging to prioritize FAIR procedures amidst competing research needs. In practice, applying FAIR standards involves enormous constraints on researchers, many of whom are under immense time pressure to deliver outputs and may lack practical or conceptual RDM expertise. Knowledge transfer, expertise, and experience sharing between data management teams of other similar collaborative centers can help researchers better understand the perspectives of other researchers. Implementing effective RDM strategies have the potential to improve the efficiency and accuracy of research and also to reduce the amount of time spent on data management by individual researchers [108].

Implementing effective data management strategies and ethical rules for the reuse and sharing of high-quality data may reduce redundant research [109, 110]. One of DM goals is to make study results freely available through open-access publishing. By investing in collaborative projects with long-term goals, it ensures that the data are organized in a way that makes them easily accessible and retrievable for future use. This makes it easier and faster to develop new research projects, as well as to replicate or build on existing studies, which should have a direct impact on public research funding (https://sor.senate.ca.gov/sites/sor.senate.ca.gov/files/0842%20policy%20matters%20Research%2003.18%20Final.pdf). Moreover, effective DM strategies can also enable to optimize public research funding by pooling resources and infrastructure from multiple sources and bringing together experts from universities, research institutes, and other community organizations to work on long-term interdisciplinary projects. Furthermore, by ensuring proper RDM, researchers should be able to reduce animal use. For example, making informed decisions about which animal models to use for their studies should enable them to use the same animals for multiple experiments, instead of having to continuously use new animals for each study. In addition, sharing previously acquired data with adequate metadata, or reuse of control group data from similar studies can avoid repeating in vivo work [111-114].

We learned from our own experience of implementing RDM activities across the consortium that the benefits of existing infrastructure and resources are often not fully realized for many projects, partly due to uncertainties regarding the organizational and technical requirements and a lack of knowledge of existing resources. Therefore, we put a great emphasis on promoting and encouraging the use of available generic tools and infrastructures when possible. Our RDM implementation strategy is based on the flexible and easy integration of existing, maximally generic components to support researchers in implementing specific solutions for data collection, processing, analysis, storage, publication, and, when appropriate, the development of sustainable, project-specific infrastructures, such as data and metadata standardization tools.

The central strategy of our CRC involves coordinated, cross-species analyses in experimental animal models and human subjects as well as patients with chronic pain using multi-scale imaging, electrophysiological, psychometric and behavioural readouts and number of interventional strategies across rodent and human populations. Our CRC involves translational research projects, with the goal of translating animal research to humans or from basic science to treatments and therapies that benefit patients. In order to facilitate the long-term preservation of valuable data sets, we developed a CRC research data management policy (see section funding information and Supplementary Information 1) in accordance with the German Research Foundation, DFG, guidelines for research data handling (https://www.dfg.de/download/pdf/foerderung/grundlagen_dfg_foerderung/forschungsdaten/guidelines_research_data.pdf). The CRC data policy serves as recommended guidelines for individual projects on how to format their data. The acquisition of heterogeneous data in multiple projects can render the process of data formatting challenging, and the data policy guidelines do not specify a level of granularity for data formatting. However, the guidelines do provide general recommendations for how to format such diverse datasets, such as using standard data formats and tagging data with descriptive metadata. We supported individual research groups by providing resources and funds for the implementation of modern tools and infrastructure for compatibility with community RDM standards. We recommended using "standard" formats for data of similar modalities (e.g., neuroimaging data (MRI) is formatted to NIfTI, electrophysiology data is formatted to NWB, etc.) as described in the data standardization sections. The data policy outlines the importance of data documentation, and data sharing and encourages the use of open-access repositories for data sharing. Our RDM services also provides general support for research groups such as help in deploying cloud-based data storage solutions (e.g., Amazon S3, Google Cloud Storage, and Microsoft Azure), establishing data governance policies, using automated software to streamline data storage and retrieval, and educating researchers about data privacy and GDPR regulations, etc.

Data stewards, Community engagements and collaborations

Data managers and stewards are essential for implementation of data policy and governance procedures, especially for large consortiums [115]. These positions are well-suited to individuals with a background in research or computer science, bioinformatics, and strong communication skills.

Furthermore, depending on the needs of the consortium, the candidate should have experience developing high-throughput analysis pipelines, domain-specific data structures and standards, open access publishing, and modern data science approaches, high-performance computing environments, cloud computing, data security, and databases, among other things. The role of data manager is diverse, and it works in close collaboration with core computing and library resources to streamline access to the consortium's common research data infrastructure, which is available at the host institutions of participating labs.

Data managers should also support ongoing research, advise on best practices for data handling, and stay informed about current developments in the RDM field. Furthermore, they should act as an important liaison between consortium researchers, collaborators, the university's RDM planning group and computing center, and community organizations. They aid in bridging the gap between the lab-based scientists and the available technical infrastructure and services. Direct assistance in daily tasks such as data organization, tool selection, workflow development, and standardization is indeed beneficial for individual researchers and research groups. Addressing data management tasks relatively early in research timelines is necessary to make the research process more efficient, and to ensure the interoperability and reusability of data sets. The expert guidance of existing infrastructure and resources, such as scientific repositories, databases, legal and ethical issues, etc., is also necessary to promote an effective data-sharing strategy.

Data managers must maintain consistent communication with various research groups and other similar consortiums to establish a community network and links to other scientific communities such as the national research data infrastructure (NFDI) consortia. As a result, data are disseminated throughout the community, and data management techniques specifically designed to facilitate neuroscience research are developed. For example, our consortium is actively engaged in a number of international and national RDM initiatives, including the EBRAINS ((https://ebrains.eu/), National Node Consortium Germany), the NFDI bioimaging initiative in Germany NFDI4BIOIMAGE (https://nfdi4bioimage.de/), which promotes the development of high-level infrastructure and services across various scientific disciplines. Our involvement with different task areas (e.g., Neuromorphic computing (NMC), Data Analytics, Workflows, GDPR, The Virtual Brain Cloud, etc.) of these community-led initiatives supports the development of a sustainable and community-oriented RDM strategy. Our CRC puts efforts into following the recommendations of The International Neuroinformatics Coordinating Facility (INCF; https://www.incf.org) [116] and harmonizing our RDM efforts by using community-developed standards that have been accepted as international standards for neurophysiology and neuroimaging datasets (for example, BIDS, NWB etc., full list available at https://www.incf.org/resources/sbps). Resources such as FAIRsharing (https://fairsharing.org/ [117] and the UK Digital Curation Centre (https://www.dcc.ac.uk/) provide a comparative view of data and metadata standards. In addition to these domain-specific initiatives, we are engaging with Research Data Alliance (RDA, https://www.rd-alliance.org/) and European Open Science Cloud (EOSC, https://eosc-portal.eu/ [118]) initiatives to adopt and develop new resources for open data exchange across technologies and scientific disciplines.

We also focused on developing RDM strategies that included joint efforts and cooperation between consortium members and other large-scale consortiums and collaborative centers within Germany. We acknowledge the common issue of data organization for different projects in collaborative centers. Joint efforts were made for the development of a data organization strategy that works for most of the projects within the consortium working in similar research areas. Our main goal was to engage more directly in several overlooked aspects of managing data in a large collaborative consortium while keeping the global neuroscience community in mind. We encouraged CRC 1158 projects to utilize logical file and folder templates to support systematic data organization. Consistent folder organization depends on the type of research data acquired for a project as well as the governance procedures. The folder structure templates for research repositories have been developed in collaboration with the DFG-funded NFDI-neuro initiative and three neuroscience CRCs (CRC 1158, CRC 1315, and CRC/TRR 135) [119]. Our goal is to provide researchers with an easy way to handle their project digital files and datasets on different data infrastructure services, both locally and on subject-specific data repositories such as GIN: a Modern Research Data Management System for Neuroscience. The template structure can be downloaded and used here: Zenodo https://doi.org/10.5281/zenodo.4410128 [120]. These folder structure templates can be customized based on the type of experiment or data modality as well as the analysis processes that should be integrated with existing data organization systems. Neuroimaging data stored in the BIDS convention, for example, can be saved in the section “03_data” for the raw data (e.g., NIfTI and JSON) and in the section “04_data_analysis” for derivatives (i.e., analyzed data). These templates, which are mostly workflow-based, are customizable to meet special requirements for individual experiments and analyses, ensuring that the structure may support rather than restrict research practices. These folder configurations can be utilized on desktop PCs, data versioning systems (e.g., GIN or DataLad), external hard drives, or any storage device to accommodate various data sets generated during experiments, independent of their format.

Sharing sensitive data from human projects

The sharing of human data gathered from clinical or non-clinical populations in neuroscience research is essential for advancing science and producing important public health benefits. A clear set of regulations and guidelines must be established before sharing human data gathered from clinical or non-clinical populations. Specific rules addressing privacy issues, established processes for data protection, data use and reuse, and the preservation of sensitive data are required. It is essential to make data accessible and understandable to remote (or future) collaborators in order to maximize the potential of existing algorithms and tools and accelerate the creation of new ones. Regulations and guidelines should ensure that the data are used for the purpose for which they were gathered and protect the rights of participants in the research. These guidelines should also cover how data should be collected, stored, shared, and destroyed. They also specify the types of data that must be kept confidential and the appropriate methods for handling and safeguarding the data. They should also ensure that the data are secure, kept confidential, and not used for marketing or other commercial purposes. Additionally, regulations should ensure that the data are used responsibly and that they are not used to discriminate against people with disabilities or other vulnerable populations. Ethical rules

for the reuse and sharing of data should be based on the principle of informed consent. This includes obtaining consent from the original data collectors or from research participants, as well as obtaining permission from any third parties involved in the data collection. Researchers should also seek to minimize the risk of data misuse or breach of confidentiality, and any data that are shared should be done so in a secure manner.

Additionally, there is a lack of efficient software programs to adequately segregate and maintain control over sensitive data. It can be difficult to develop effective software that is secure, user-friendly, and cost-effective. The maintenance of such software requires a significant investment of resources, and often there is a lack of funding available for such measures. Finally, the adoption of such software requires investment in training and resources, which many organizations may be unwilling to do. The legal and ethical requirements surrounding the use of sensitive data are often complex and difficult to understand, leading to confusion and ambiguity about the best way to protect them. It is important to provide researchers working with sensitive data or samples with truly "useful" tools that do not require pre-existing, in-depth knowledge of legal and ethical requirements, or time to delve into the details. Such tools are important to ensure that sensitive data are protected and securely stored. Use of such tools can help researchers to make informed decisions about how to best use and manage sensitive data, allowing them to work with it in an ethical and responsible manner. Finally, these tools can help to reduce the risk of data breaches and data misuse, which can have serious consequences for the people and organizations whose data are affected. By providing such tools, researchers can focus on their research and not on legal and ethical considerations, thus saving their time and resources.

There are several software tools that can be used to maintain sensitive patient data in neuroscience research. Some examples include a web-based platform REDCap (Research Electronic Data Capture) [121], an open-source imaging informatics platform XNAT (XNAT Central) [122], LORIS: Longitudinal Online Research and Imaging System [123] etc. It is important to note that the security features of these tools may vary and should be evaluated before use. In addition to software tools, secure data storage and access protocols should also be in place to ensure that sensitive patient data are protected.

We are currently expanding our collaborative efforts by creating a data infrastructure platform that will establish a GDPR-compliant data registry (PainReg-registry, based on the Germany-wide ParaReg registry (www.parareg.de) [124] for human volunteers. To facilitate cross-project data merging, a core clinical data set will be defined. This entails assigning a unique identifier to each study participant that is shared by all projects, allowing researchers to determine whether the volunteer participated in multiple projects. This will allow organizations to share the same pool of volunteers for multiple studies, resulting in less redundant data acquisition. This can result in cost and time savings, as well as increased data collection accuracy. We, for example, experienced that a same study participant could be tested twice, and was assigned different IDs (belonging to different projects), resulting in redundant data acquisition and therefore unnecessary increased costs, particularly for genetic analysis.

Furthermore, the data registry will ensure that data privacy regulations are strictly followed by obtaining participants' consent to access data for secondary or follow-up studies. This will also include an identity management feature to limit access to authorized users. The registry will contain a wide range of data, including brain imaging, genetic, cognitive, and physiological data. This collaborative work will be coordinated by the consortium's future data infrastructure project, which will be tasked with implementing, testing, optimizing, and standardizing data analysis procedures and models that will be utilized in all projects.

Data integration

For collaborative research, the data integration and standardization step becomes crucial for interoperability and data sharing [125], but is quite challenging to implement, given the wide range of methodologies represented in the consortium. Early data standardization can have massive benefits for data integration in collaborative projects, this can be achieved by streamlining the use of tools for more replicable and reproducible analysis. The data integration process often depends on individual projects and their underlying workflow and processes. The degree to which this is possible will depend on the modalities used, the subject population, and the experimental design. Researchers may also integrate the raw data collected from each partner into a core dataset. Integrated datasets can provide a more comprehensive understanding of the research question, as well as allowing the researchers to compare the results of their analyses more directly. Depending on the modalities used, the data may need to be transformed or normalized before integration, and the analysis techniques may need to be adapted to the combined dataset.

For some projects, researchers can go as far as analyzing data from their tandem partners and vice versa. Some human projects combine fMRI data from one group and EEG data from another group to gain a better understanding of how the two modalities interact. This could also involve combining the datasets, or running analyses on the combined dataset to identify common patterns or trends. However, this process requires careful consideration of the data sources, data formats, and analysis techniques used by individual labs, as well as the selected methods for data fusion and data mining. At the most basic level, researchers can compare the data collected from each partner to identify commonalities and differences in the data. This could include comparing the number and type of modalities used, the subject population, the experimental design, and the type of analysis performed. For example, they could investigate how brain structure (grey matter volume, cortical thickness) relates to behavior. There are also association studies aiming to compare brain activity between two groups of participants (e.g., healthy, chronic pain) in order to explore neural differences in cognition or behavior. They could also examine associations between neural activity in different brain areas and physiological responses of the subject.

In addition, researchers could use deep learning algorithms to look for patterns in the data and try to find insights that can improve the understanding of the brain. Deep learning algorithms, for example, can be used to detect patterns in EEG data that can be used to identify different states of consciousness or seizures. Finally, researchers could also use artificial intelligence techniques to combine multiple

datasets in order to gain a better understanding of the complex relationship between the brain and behavior.

From our own experiences, we came to realize that a systematic effort to develop standardized guidelines for multimodal data acquisition would strongly facilitate the data integration process and also adoption of FAIR data standards across all studies. Our CRC is developing a multimodal digital intervention platform aiming to combine data collected by the CRC projects for further analyses. This platform benefits from increased sample size, which should result in improved prediction accuracy and allow for optimized therapies [126] such as the use of invasive or non-invasive neurostimulations. Our current efforts in harmonizing and standardizing datasets (e.g., using BIDS) and preprocessing approaches would also facilitate this development and further improve the data analysis.

Data standardization

We have devised a set of strategies to ensure that the datasets can be thoroughly documented and converted into open data standards with the minimum amount of effort. The consortium's projects combine datasets from electrophysiological recordings, optogenetic manipulations, rodent behavior assays such as sensory testing (von Frey filaments), cold plate test, and open field test (OFT) [127, 128], two-photon in-vivo (2P), and MRI, resulting in a plethora of disparate file formats and unorganized metadata. These datasets are saved in a variety of file formats, including video files (avi), the original raw ASCII log files, text-based file formats,.csv, and detailed stimulus material (e.g., wav and png files), among others. We are utilizing deep-learning based approaches for behavior data acquisition, analysis, pose estimation etc. for both human and rodent experimental models [129, 130]. This includes software packages tools such as Noldus EthoVision XT [131] (behavior data acquisition and analysis from rodents), Bonsai [132] (behavioral tracking and closed-loop experiments), ANY-maze (RRID:SCR_014289, http://www.sandiegoinstruments.com/any-maze-video-tracking/) (automated video tracking software), PsychoPy [133] (data acquisition and analysis from humans), SPSS (Statistical Package for the Social Sciences), Matlab and GraphPad Prism [134] (data analysis and visualization), DeepLabCut (DLC) (markerless pose estimation) [135, 136] for measuring rodent behavior [137]. However, there is no single coordinated data standardization strategy for every stage of behavioral data collection, from data acquisition to analysis, making harmonization and thus grouping different behavioral paradigms difficult [61, 138].

Electrophysiology datasets are collected using a variety of proprietary file formats for raw data as well as intermediate preprocessing/analysis, such as Cambridge Electronic Design Spike2 (.smrx) and Neuralynx (.ncs). Another observation is that many of these formats can only be read or accessed by proprietary software, imposing additional constraints on focusing on a single or even a few open data standards. Development of open data standards are often supported by the community and have a variety of actively developed interoperable tools for data import and export, validation, and analysis which can form the core of standard workflows. However, including all of these raw and intermediate preprocessing file types in a single standard format was impractical. It was unrealistic in our consortium to advocate or propose that a single data standard could accommodate the wide range of data

modalities and analysis applications that are commonly used within the consortium. To enable thorough metadata description, it was important to adopt an open data format that includes consistent metadata structures. This is one area in which the development of local, customized solutions is essential. Additionally, we started exploring and assessing available data and metadata standardization resources, such as open-source tools, data conversion pipelines, and file formats and their specifications, we identified a number of open-source tools and data formats specific to neurophysiology that could potentially be utilized for our consortium's use cases. This includes the Neuroscience Information Exchange (NIX) Format [139], Neurodata Without Borders (NWB; https://www.nwb.org) [93, 140] etc., data versioning tools (e.g., DataLad (https://www.datalad.org/) [141], and GIN (https://gin.g-node.org/), metadata collection tools (e.g., CEDAR, NIDM, Open metadata markup language (odML) [43], data representation models (Neo) Python library Electrophysiology Analysis Toolkit (Elephant), data analysis tools (e.g., Elephant, FieldTrip), PyNN [142, 143], etc. The data formats are compatible with a wide range of modern software packages and analysis tools, including Brainstorm, Elephant, Spike2, and NeuroExplorer, and the majority of these tools are open-source. The CRC also encouraged the development of new tools and pipelines to reduce the time and effort usually needed by individual labs.

For example, NWB data conversion tools (https://github.com/catalystneuro/nwb-conversion-tools) can be utilized to build conversion pipelines for putting additional raw file formats (e.g., .rhd from Intan RHD recording system, .smrx from Spike2 etc.) into standard data formats. It is beneficial for data handling within the laboratory as well as the development of standardized acquisition and analysis workflows. Another useful resource might be (BIDS-animal-ephys) (https://nfdi-neuro.de/wp-content/uploads/2021/07/Sprenger_BIDS_Extension_032.pdf), which is an extension of BIDS to support other types of neuroscientific data, such as electrophysiological data recorded in animals.

Support for the adoption of an electronic lab notebook across the entire consortium was one of the first steps in metadata documentation and standardization. There are several commercial and open-source ELN options available for documentation of experiments and results. The Harvard Biomedical Data Management Group has created an ELN Matrix (https://zenodo.org/record/4723753) and ELN Finder (https://eln-finder.ulb.tu-darmstadt.de/home) provides information on a wide range of currently available software. In our consortium, Individual researchers have benefited greatly from our computing center's local installation of the elabFTW, which includes secure cloud-based data storage. The service can also be used to serve as a central repository for documenting shared or collaborative experimental methods or protocols used by two or more CRC labs. Each project can have its own team, with members from participating labs having access to it. Transfer of these protocols from traditional notebooks or digital documents into ELN needed additional time and effort from project members. In order to ensure that the ELN is not seen as an additional burden, careful consideration had to be taken when automating the workflow. For example, the ELN must be designed to integrate seamlessly with existing protocols and should provide easy access to data at all stages of the experiment. Overall, most labs find that the benefits of ELNs outweigh any additional burden, as the system can save time and effort in the long run by automating data entry and providing easy access to protocols and data.

Within our RDM framework, ELN can be used as a platform for documenting minimal metadata that is generated automatically while performing an experiment. ELN are frequently not developed as full-fledged metadata systems and lack domain-specific features (such as support for reading or viewing neuroscience-specific file formats). Another issue is that most datasets lack consistent metadata schemas, even at the most basic level. In this particular instance, we use elabFTW, which accepts JSON files. elabFTW acts as a "notebook," tracking both primary data (experiment findings, measurements, etc.) and metadata (date, time, author, units, used inventory, etc.). The experimental metadata (e.g., microscope specifications, data acquisition settings) is stored in a standardized manner using a generic metadata file format that is compatible with open file formats, such as JSON or XML. However, from our experience, the integration of metadata from complex experimental protocols, and analysis procedures into an ELN has proven to be a challenge. Support for various data or metadata file formats generated during acquisition and analysis is not within the scope of elabFTW. Users frequently choose to standardize their datasets at any stage of their project lifecycle; by this, we mean that users standardize datasets either immediately after data acquisition (raw files), or during the pre-processing or final analysis stage before sharing or publishing. Adding any additional metadata information into the ELNs (e.g., elabFTW JSON file) without an automated tool or API can also be time-consuming. So, if a user needs to add, search, filter, or use various types of metadata on a regular basis, they will require a specialized tool.

To address these issues, we emphasized the importance of developing templates for the most common types of experiments performed in a single project (based on design protocols, biological methods, and so on). The development of a metadata GUI also enabled us to automate the process of metadata standardization in an experiment-specific manner.

We would also like to highlight some of our consortium's use cases. The experimental protocol for a project that collected a large amount of electrophysiological data was available on elabFTW, along with a JSON file containing basic experimental metadata. We made the decision to standardize raw data files such as .rhd and convert them to NWB format. On elabFTW, the basic experimental information/metadata was available, but not in a format compatible with NWB metadata. When we standardize raw data files, we frequently discover that the associated metadata files lack analysis parameters. The challenge now is to integrate all types of extensive metadata (experimental, acquisition, and analytical metadata) in a single file with a consistent format to ensure image data quality, reproducibility, and scientific outcome.

In this case, the metadata GUI's goal was to generate a JSON file based on custom JSON schemas substantial enough to generate a NWB file for a given experiment. These pre-defined JSON schemas are built around a specific experiment setup, such as intracellular electrophysiology data collected from the Intan RHD acquisition system. Once the JSON-schemas are created, all the labs conducting similar experiments could use these schemas to feed metadata collected from various acquisition systems and generate a JSON file. This also allows for customized validation of the data, which can ensure that the data is valid and accurate before being entered into the ELN. By specifying the data in a custom GUI,

a more efficient and reliable workflow within the lab is enabled, as well as ensuring that all necessary information is included and kept in a consistent format.

Another scenario is where users can use the bioformat library to extract metadata from TIFF and other imaging file formats into JSON format, then add any additional fields using a custom tool and feed this file back into elabFTW if necessary.

Data infrastructure:

Adoption of Project-specific DMPs:

Funding agencies and research organizations are increasingly requesting data management plans (DMPs) when submitting a grant application. The obligation to submit a DMP and timeline depends on the requirements of the funding organizations. DMPs should be created early on, ideally when applying for funds or at the beginning of a research project, and updated as needed. For example, ERC-funded projects that participate in the Horizon 2020 Open Research Data (ORD) pilot are required to submit the first version of their DMP within six months after the start of their grant. Open access publications are encouraged, and grantees should demonstrate FAIR-compliant data management and resource use. However, some research studies involving sensitive data are exempt from these requirements.

The DMP developed for each research project highlights relevant information regarding research data and associated metadata that is required for research result reproducibility. Preliminary versions of DMPs can surely assist participating labs in making informed decisions about their data management resource requirements (financial support or personnel). DMPOnline (https://dmponline.dcc.ac.uk/) and RDMO (https://rdmorganiser.github.io/) are two commercial open-source software solutions for creating custom DMPs [144, 145]. Several DMP templates have already been made available in response to funding agency criteria (https://dmponline.dcc.ac.uk/public templates).

Individual project DMPs can be created using these templates, or if a dataset requires particular RDM resources, a dataset-specific DMP can be created. These DMP templates cover questions about how data is handled at each stage of the project, including a general project description; experimental and dataset descriptions; specific data documentation (types of data and experimental models; methods for acquisition and collection; questionnaires; analysis software); decisions on data and metadata standards and formats; and proposed plans for organization, access, sharing, short- and long-term storage, re-use, and implementation costs. The document, once prepared, explains the management of the research data acquired, reviewed, and processed as part of the CRC 1158 initiatives. The template includes some generic questions regarding best practices for each stage of the data management lifecycle that may be answered early in the project, while domain-specific questions can be answered later in the project.

Data storage, organization and sharing:

In addition to providing support for access and use of internal university resources for data storage and sharing, the CRC also supports adoption of innovative community-developed solutions. Versioning of data sets, along with software and code, becomes critical for such projects as data files and metadata are updated over time. Even in the case of complete datasets published or submitted in a repository,

versioning helps in tracking changes in the data files or metadata that are incorporated after data re-use or re-analysis. For example, considering that some CRC projects are now running for multiple funding periods and are performing extended analyses. This includes, for example, data comparison between various groups of pain patients collected at different funding periods, or associations between various types of data modalities (e.g., data collected using electroencephalography for the first funding period, and fMRI data during the next funding period); or simply comparisons between various analysis toolboxes (e.g., fMRIPrep [146] vs. SPM).

There are platforms such as DataLad (a US-German collaboration for computational neuroscience project (https://www.datalad.org/) and GIN (https://gin.g-node.org/) that may effectively compensate for a lack of local resources. These data hosting and sharing platforms can also ensure data versioning and encourage reproducible management of scientific data. Both DataLad and GIN are based on git and git-annex to provide a decentralized system for the exchange of large datasets. Datalad and GIN are interoperable, as datasets hosted on either of these platforms can be accessed via git-compatible systems. Datalad is an open source software package for the management of distributed datasets. It facilitates the sharing, retrieval, and organization of data in a distributed environment. Moreover, the GIN service can be deployed locally at all the participating labs and can be used as an in-house storage server and web user interface for DataLad datasets. Datalad does not offer storage, but it provides tools for the acquisition, organization, and management of data stored in remote repositories. Another example resource is the Open Science Framework (https://osf.io/), which is an open-source web-based application for supporting collaborative workflow development. Open Science Grid (https://opensciencegrid.org/) is another emerging collaborative platform that can integrate both data hosting and processing/analysis computing resources.

Data analysis and visualization

We are currently refactoring and developing image analysis tools for the automated running of deep learning applications on bwVISU. With such extra computational resources, it is possible to set up automated analysis workflows on HPC that could allow for faster, more accurate diagnoses in near-real time. The goal of this project is to implement a deep-learning application programming interface (API) for image data processing and to provide a platform for the scientific community to directly compare and integrate data generated across our consortium projects. The development of an open-source and extensible platform to train and share deep-learning models will guarantee high standards in many image analysis workflows and additionally reduce the amount of annotated data necessary for training. supervised deep-learning algorithms. For example, our initial efforts involve integrating the most commonly used deep-learning image analysis tools to make the initial GUI more flexible for model training and inferences. Some of the considered tools include StarDist [147], Noise2Void (image denoising) [148], CellPose (cell segmentation) [149] and Elektronn3 (EM data segmentation, https://github.com/ELEKTRONN/elektronn3).

Data dissemination

Public neuroscience repositories are rapidly being developed and a lot of progress has been made in this direction. Several data repository options can be found in online resources such as https://www.re3data.org/ and/or https://fairsharing.org/. It is worthwhile to search both domain/format-specific repositories and scientific repositories (for example, OpenPain (http://www.openpain.org/) and Pain and Interoception Imaging Network (PAIN) repository (https://www.painrepository.org/) [150]. In the context of bioimaging data storage and sharing, the EMBL-EBI BioImage Archive (BIA) is a large-scale, centralized data resource that hosts reference imaging data. The OpenfMRI project [18], which was originally created for the free and open sharing of raw MRI datasets (old datasets available at https://legacy.openfmri.org/), has since expanded to include datasets from other neuroimaging modalities such as MEG, EEG, and PET, and has been renamed the OpenNeuro Project (https://openneuro.org), [151]. Certain repositories require datasets to be submitted in a standardized format; for example, OpenNeuro (which accepts anonymized human-derived datasets), OMEGA (Open MEG Archive, exclusively for MEG data), and MNE-BIDS have all adopted the BIDS format (which links BIDS and MNE-python analysis tool for MEG and EEG data). In addition to providing basic features such as data hosting and support for metadata files, there are repositories that provide restricted data sharing and anonymization services, which are highly suitable for publishing datasets from clinical projects. The Cancer Imaging Archive (TCIA) and the LONI Image Data Archive (IDA) are two examples.

Other neuroscience-focused data repositories with specific purposes include G-node GIN for datasets derived from both human and non-human organisms, BrainLife (human neuroimaging), (https://brainlife.io/), Distributed Archives for Neurophysiology Data Integration (DANDI) (https://registry.opendata.aws/dandiarchive/), and Fenix-backed EBRAINS [152]. The HBP EBRAINS data curation team may assist with data submission and integration, as well as provide defined embargo durations to allow for progressive disclosure. The EU-funded Human Brain Project produced EBRAINS, an open European digital research infrastructure that provides one of the most complete platforms for sharing brain research data of various types, spatial and temporal scales.

Aside from these domain-specific repositories, numerous well-known open data repositories, sharing and management platforms accept data from a wide range of disciplines. Zenodo [153] and Dryad, for example, is an online archive that manages research datasets with metadata and allows long-term data access via persistent identification. Figshare (https://figshare.com/) [154], a commercial free data repository with unique features such as custom storage options, version control, visualization, metadata customization, data curation using DOI, and so on, is one example. The EMBL SourceData SmartFigure (https://sourcedata.embo.org/) focuses on the scientific figure as a sharing unit, combining data sharing and visualization. The Harvard Dataverse Network is both a platform for institutions and a data repository implemented on FAIR data principles to publish, share, reference, extract, and analyze research data. Consortiums offering support and access to cloud computing, such as OpenScienceGrid, JetstreamCloud, Fenix, and the European Commision-backed European Open Science Cloud (EOSC), can support analysis if institutional solutions are not available.

Apart from contributing data to repositories, dissemination of source code, experimental protocols, research software etc. is of utmost importance in order to replicate or reproduce research findings [155]. This approach can be combined with standardization of complete existing datasets and facilitating contribution to repositories. There are some particular cases where data sharing for a complex project that includes sensitive data is much more complicated in practice and is not as straightforward. It requires some additional steps of consent forms, access regulations, and anonymization strategies to maintain the confidentiality of the data.

For the long-term preservation of data, researchers need permanent archiving systems, along with sufficient funds to build such archives for both internal usage and to satisfy the open data needs of journals and funding organizations. Ideally, archival systems should be developed with the user's perspective, especially in scientific settings where researchers with limited expertise in digital preservation collaborate on projects that generate a wide range of data [156].
Researchers require assistance with best practices for archiving in addition to access to archival systems. For instance, how to remain informed about the storage, retention, and disposal of all research data, whether in an institutional or external repository, especially as good archival practice includes a scheduled review of items in long-term storage. Support may also be needed to determine that data handling complies with various regulations and/or guidelines: existing discipline-specific privacy and ethical standards; existing copyright or licensing arrangements; and publication and legal requirements. The period for which data should be preserved for research purposes or archiving should be determined by prevailing standards for the specific type of research domain, and should follow the retention policies of any applicable stakeholders (e.g., sponsoring institution, funding agency). For example, in the context of our consortium funded by the DFG, primary research data should be appropriately archived in the researcher's own institution or an appropriate nationwide infrastructure for at least 10 years (DFG Guidelines on the Handling of Research Data).

CONCLUSION

We have presented a data management strategy that we developed and put into practice within the framework of a collaborative research center, encompassing both basic and clinical research on humans and animals. To foster FAIR and open science, this strategy strives to offer practical solutions for multimodal and multidisciplinary research.
This strategy is composed of adaptive and incremental phases: planning, implementation, and dissemination. Consistent communication with consortium project members during the planning and implementation phases was crucial to identify the most helpful RDM measures. We spent a considerable amount of time learning about publicly accessible tools and services and new developments in the RDM field that could be beneficial for our consortium.
In the planning phase, we evaluated common data management practices across projects. We categorized projects based on the typical population studied and the common measurement methods used. We focused on addressing issues such as metadata management; experimental protocol

documentation; preprocessing and analysis pipelines; data storage and volume; data sharing, data dissemination and archiving, sensitive data-related issues that arise when working with highly diverse and heterogeneous data. The complexity was subsequently raised by the major RDM challenges encountered in tandem projects that work with both human and animal populations, such as including data and metadata standardization, the integration of various different data types, and the harmonization of datasets and analysis workflows.

In the implementation phase, we presented some innovative solutions based on pre-existing and customized solutions developed for flexible and incremental data management solutions with a focus on research collaborations. We discuss the implementation of project-specific data management plans, structured based on data acquisition, processing, and analysis methods across the CRC 1158 projects. Relatively simple measures, such as offering ELN options for documenting experimental protocols or tutorials on HPC resource access, regular data seminars on basic RDM tools (such as data versioning tools, code and workflow management software, etc.), can improve data management practices in noticeable ways across the consortium. We focused on the development of new tools for metadata organization and management depending upon the requirements of each project and the type of data collected. In animal projects, we assisted with migration from proprietary formats and supported experimental annotation and organization. Moreover, for large datasets, we provided easy access to software and tools on large web-based applications to enable interactive analysis and visualization.

For the organization of human projects, we adopted standard protocols to associate various data types with the respective metadata. In the case of MRI, we standardized MR acquisition protocols, data organization, and preprocessing pipelines. Behavioral, sensory testing, and psychological questionnaires were standardized by a service project.

The CRC 1158 emphasizes that active communication and engagement with general and domain-specific RDM community initiatives is required for the development of RDM strategies for any large-scale research consortium. Modern research infrastructure and technological advancements, such as web-based technologies for sharing data and analysis tools provide opportunities to increase the reproducibility of research outcomes in both basic and translational neuroscience.

Further development of this RDM model with more specialized technical infrastructure is envisioned for the next period of the consortium. A federated data sharing approach is required for multi-site and multi-species projects, which will allow for the integration of data from different computer systems for participating labs that are geographically distributed without moving the data to a centralized location.

CRC 1158 Data management policies and funding information:

Collaborative Research Centers (CRC; short SFB for German ‘Sonderforschungsbereich’) are university research projects which are funded by the German Research Foundation (DFG), generally for a period of up to 12 years. https://www.dfg.de/foerderung/programme/koordinierte_programme/sfb/.

The Heidelberg Pain Consortium (https://www.sfb1158.de/) is a collaborative research center (CRC 1158) composed of 44 principal investigators in Germany investigating the neurological basis of pain. Since 2015, the German Research Foundation (DFG) has supported CRC 1158

(https://gepris.dfg.de/gepris/projekt/255156212?context=projekt&task=showDetail&id=255156212). In June 2019, CRC 1158 was successfully renewed and got funding for another four years, 2019–2023, under project number 255156212 from DFG. CRC 1158 has many national (e.g., the University of Heidelberg, the Central Institute for Mental Health (ZI), European Molecular Laboratory, and German Cancer Research Center) and international collaborations (Institutions located in the United States, Canada, England, and France).

The Heidelberg Pain Consortium implemented a development strategy in central administration project (Z01) (https://gepris.dfg.de/gepris/projekt/278997686) to promote RDM as an integral part of the research process in order to maximize the impact of collaborative science. By implementing this strategy, the consortium is able to take a systematic and standards-based approach to documenting, archiving, and sharing its research data with collaborators and the research community, with the goal of significantly accelerating scientific progress. This RDM model is expected to evolve in response to the development of new and specialized (domain-specific) technical infrastructures.

As a CRC host institution, Heidelberg University offers comprehensive recommendations for the administration of research data (http://www.uni-heidelberg.de/universitaet/profil/researchdata/). CRC's data policy (available in supplementary data 1) highlights the use of a variety of RDM services to researchers to ensure that RDM for each project adheres to the DFG guidelines (https://www.dfg.de/en/research_funding/principles_dfg_funding/research_data/). These services include aid with proper data documentation, the integration and support of open data management solutions, data storage and accessibility, the development of new tools for the adoption of open data and metadata standards, the sharing of various diverse data sets within the consortium and with external collaborators, and the dissemination of research outcomes into national and international data repositories. The policy is applicable to all researchers working in the CRC, including principal investigators (PIs), doctoral and postdoctoral researchers, and student research assistants. It also applies to any research projects carried out within the CRC as well as any data generated or used (from outside sources).

ACKNOWLEDGMENTS

This work was supported by the Deutsche Forschungsgemeinschaft (255156212 – CRC 1158 TP Z01). GIN project has been partially funded by Deutsche Forschungsgemeinschaft (DFG), project numbers 222641018 – CRC/TRR 135 TP INF, 255156212 – CRC 1158 TP Z01, and 327654276 – CRC 1315 TP Z.

The authors gratefully acknowledge the members of the Research Data Competence Center (KFD), University of Heidelberg, especially, Martin Baumann, Jochen Apel, Georg Schwesinger, Alexander Haller for their constant RDM support and technical guidance. We fully acknowledge the data services SDS@hd supported by the Ministry of Science, Research and the Arts Baden-Württemberg (MWK) and the German Research Foundation (DFG) through grants INST 35/1314-1 FUGG and INST 35/1503-1 FUGG. We acknowledge the members of the NFDI-Neuro and NFDI4BIOIMAGE communities for

informative discussions on RDM developments. We acknowledge the contributions of the catalystneuro team for the development of the metadata GUI (funded by internal funds from CRC 1158). The authors acknowledge support by the state of Baden-Württemberg through bwHPC. We also thank Pooja Gupta, Christl Gartner and Anne Seller for their help with coordination; Carlo Beretta and Keval Paida for discussions on image analysis; Paul Naser and Philipp Roth for assistance with older versions of figures. Rebecca Mease is supported by the Brigitte-Schlieben-Lange Programm and the Chica Heinz Schaller Foundation.

We thank the reviewers for their thoughtful and thorough review and we believe their input helped us in improving the quality of the manuscript.

COMPETING INTERESTS STATEMENT:

This manuscript reflects only the author's views and the funding agencies are not liable for any use that may be made of the information contained therein. The authors declare no conflict of interest.

REFERENCES

1. Klump J, Bertelmann R, Brase J, Diepenbroek M, Grobe H, Höck H, et al. Data publication in the open access initiative. Data Sci J. 2006;5:79-83.
2. Marcial LH and Hemminger BM. Scientific data repositories on the Web: An initial survey. Journal of the American Society for Information Science and Technology. 2010;61 10:2029-48. doi: 10.1002/asi.21339.
3. Gouwens NW, Sorensen SA, Berg J, Lee C, Jarsky T, Ting J, et al. Classification of electrophysiological and morphological neuron types in the mouse visual cortex. Nat Neurosci. 2019;22 7:1182-95. doi:10.1038/s41593-019-0417-0.
4. Juavinett AL, Bekheet G and Churchland AK. Chronically implanted Neuropixels probes enable high-yield recordings in freely moving mice. Elife. 2019;8 doi:10.7554/eLife.47188.
5. Kleinfeld D, Luan L, Mitra PP, Robinson JT, Sarpeshkar R, Shepard K, et al. Can One Concurrently Record Electrical Spikes from Every Neuron in a Mammalian Brain? Neuron. 2019;103 6:1005-15. doi:10.1016/j.neuron.2019.08.011.
6. Sych Y, Chernysheva M, Sumanovski LT and Helmchen F. High-density multi-fiber photometry for studying large-scale brain circuit dynamics. Nat Methods. 2019;16 6:553-60. doi:10.1038/s41592-019-0400-4.
7. Zeisel A, Hochgerner H, Lonnerberg P, Johnsson A, Memic F, van der Zwan J, et al. Molecular Architecture of the Mouse Nervous System. Cell. 2018;174 4:999-1014 e22. doi:10.1016/j.cell.2018.06.021.
8. Asher A, Deards K, Esteva M, Halbert M, Jahnke L, Jordan C, et al. Research data management: Principles, practices, and prospects. Tech rep Council on Library and Information Resources. 2013.
9. Tenopir C, Allard S, Douglass K, Aydinoglu AU, Wu L, Read E, et al. Data sharing by scientists: practices and perceptions. PLoS One. 2011;6 6:e21101. doi:10.1371/journal.pone.0021101.
10. Rubel O, Dougherty M, Prabhat, Denes P, Conant D, Chang EF, et al. Methods for Specifying Scientific Data Standards and Modeling Relationships with Applications to Neuroscience. Front Neuroinform. 2016;10:48. doi:10.3389/fninf.2016.00048.
11. Lahat D, Adalý T and Jutten C. Challenges in multimodal data fusion. In: *2014 22nd European Signal Processing Conference (EUSIPCO)* 1-5 Sept. 2014 2014, pp.101-5.
12. Buckow K, Quade M, Rienhoff O and Nussbeck SY. Changing requirements and resulting needs for IT-infrastructure for longitudinal research in the neurosciences. Neurosci Res. 2016;102:22-8. doi:10.1016/j.neures.2014.08.005.
13. De Martino F, Valente G, de Borst AW, Esposito F, Roebroeck A, Goebel R, et al. Multimodal imaging: an evaluation of univariate and multivariate methods for simultaneous EEG/fMRI. Magn Reson Imaging. 2010;28 8:1104-12. doi:10.1016/j.mri.2009.12.026.
14. King KM, Littlefield AK, McCabe CJ, Mills KL, Flournoy J and Chassin L. Longitudinal modeling in developmental neuroimaging research: Common challenges, and solutions from developmental psychology. Dev Cogn Neurosci. 2018;33:54-72. doi:10.1016/j.dcn.2017.11.009.

15. Cragg JJ, Haefeli J, Jutzeler CR, Rohrich F, Weidner N, Saur M, et al. Effects of Pain and Pain Management on Motor Recovery of Spinal Cord-Injured Patients: A Longitudinal Study. Neurorehabil Neural Repair. 2016;30 8:753-61. doi:10.1177/1545968315624777.
16. Zheng CJ, Van Drunen S and Egorova-Brumley N. Neural correlates of co-occurring pain and depression: an activation-likelihood estimation (ALE) meta-analysis and systematic review. Transl Psychiatry. 2022;12 1:196. doi:10.1038/s41398-022-01949-3.
17. Hashmi JA, Baliki MN, Huang L, Baria AT, Torbey S, Hermann KM, et al. Shape shifting pain: chronification of back pain shifts brain representation from nociceptive to emotional circuits. Brain. 2013;136 Pt 9:2751-68. doi:10.1093/brain/awt211.
18. Poldrack RA, Barch DM, Mitchell JP, Wager TD, Wagner AD, Devlin JT, et al. Toward open sharing of task-based fMRI data: the OpenfMRI project. Front Neuroinform. 2013;7:12. doi:10.3389/fninf.2013.00012.
19. Gorgolewski KJ, Auer T, Calhoun VD, Craddock RC, Das S, Duff EP, et al. The brain imaging data structure, a format for organizing and describing outputs of neuroimaging experiments. Sci Data. 2016;3:160044. doi:10.1038/sdata.2016.44.
20. Pernet CR, Appelhoff S, Gorgolewski KJ, Flandin G, Phillips C, Delorme A, et al. EEG-BIDS, an extension to the brain imaging data structure for electroencephalography. Sci Data. 2019;6 1:103. doi:10.1038/s41597-019-0104-8.
21. Niso G, Gorgolewski KJ, Bock E, Brooks TL, Flandin G, Gramfort A, et al. MEG-BIDS, the brain imaging data structure extended to magnetoencephalography. Sci Data. 2018;5:180110. doi:10.1038/sdata.2018.110.
22. Wilkinson MD, Dumontier M, Aalbersberg IJ, Appleton G, Axton M, Baak A, et al. The FAIR Guiding Principles for scientific data management and stewardship. Sci Data. 2016;3:160018. doi:10.1038/sdata.2016.18.
23. Bouchard KE, Aimone JB, Chun M, Dean T, Denker M, Diesmann M, et al. High-Performance Computing in Neuroscience for Data-Driven Discovery, Integration, and Dissemination. Neuron. 2016;92 3:628-31. doi:10.1016/j.neuron.2016.10.035.
24. Dinov ID, Petrosyan P, Liu Z, Eggert P, Hobel S, Vespa P, et al. High-throughput neuroimaging-genetics computational infrastructure. Front Neuroinform. 2014;8:41. doi:10.3389/fninf.2014.00041.
25. Perkel JM. Web service makes big data available to neuroscientists. Nature. 2018;563 7729:143. doi:10.1038/d41586-018-07195-2.
26. Goecks J, Nekrutenko A, Taylor J and Galaxy T. Galaxy: a comprehensive approach for supporting accessible, reproducible, and transparent computational research in the life sciences. Genome Biol. 2010;11 8:R86. doi:10.1186/gb-2010-11-8-r86.
27. Koster J and Rahmann S. Snakemake--a scalable bioinformatics workflow engine. Bioinformatics. 2012;28 19:2520-2. doi:10.1093/bioinformatics/bts480.
28. Brigham TJ. Taking advantage of Google's Web-based applications and services. Med Ref Serv Q. 2014;33 2:202-10. doi:10.1080/02763869.2014.897521.
29. Amari S, Beltrame F, Bjaalie JG, Dalkara T, De Schutter E, Egan GF, et al. Neuroinformatics: the integration of shared databases and tools towards integrative neuroscience. J Integr Neurosci. 2002;1 2:117-28. doi:10.1142/s0219635202000128.
30. Eickhoff S, Nichols TE, Van Horn JD and Turner JA. Sharing the wealth: Neuroimaging data repositories. Neuroimage. 2016;124 Pt B:1065-8. doi:10.1016/j.neuroimage.2015.10.079.
31. Van Horn JD. Bridging the Brain and Data Sciences. Big Data. 2021;9 3:153-87. doi:10.1089/big.2020.0065.
32. Madan CR. Scan Once, Analyse Many: Using Large Open-Access Neuroimaging Datasets to Understand the Brain. Neuroinformatics. 2021; doi:10.1007/s12021-021-09519-6.
33. Fan J, Han F and Liu H. Challenges of Big Data Analysis. Natl Sci Rev. 2014;1 2:293-314. doi:10.1093/nsr/nwt032.
34. Ferguson AR, Nielson JL, Cragin MH, Bandrowski AE and Martone ME. Big data from small data: data-sharing in the 'long tail' of neuroscience. Nat Neurosci. 2014;17 11:1442-7. doi:10.1038/nn.3838.
35. Avberšek LK and Repovš G. Deep learning in neuroimaging data analysis: Applications, challenges, and solutions. Frontiers in Neuroimaging. 2022;1 doi:10.3389/fnimg.2022.981642.
36. Council NR. Frontiers in Massive Data Analysis. Washington, DC: The National Academies Press; 2013.
37. Li X, Ai L, Giavasis S, Jin H, Feczko E, Xu T, et al. Moving Beyond Processing and Analysis-Related Variation in Neuroscience. 2021.
38. Bowring A, Nichols TE and Maumet C. Isolating the sources of pipeline-variability in group-level task-fMRI results. Hum Brain Mapp. 2022;43 3:1112-28. doi:10.1002/hbm.25713.
39. Gronenschild EH, Habets P, Jacobs HI, Mengelers R, Rozendaal N, van Os J, et al. The effects of FreeSurfer version, workstation type, and Macintosh operating system version on anatomical volume and cortical thickness measurements. PLoS One. 2012;7 6:e38234. doi:10.1371/journal.pone.0038234.
40. Friston KJ. Statistical Parametric Mapping. In: Kötter R, editor. Neuroscience Databases: A Practical Guide. Boston, MA: Springer US; 2003. p. 237-50.
41. Smith SM, Jenkinson M, Woolrich MW, Beckmann CF, Behrens TE, Johansen-Berg H, et al. Advances in functional and structural MR image analysis and implementation as FSL. Neuroimage. 2004;23 Suppl 1:S208-19. doi:10.1016/j.neuroimage.2004.07.051.

42. Bowring A, Maumet C and Nichols TE. Exploring the impact of analysis software on task fMRI results. Hum Brain Mapp. 2019;40 11:3362-84. doi:10.1002/hbm.24603.
43. Grewe J, Wachtler T and Benda J. A Bottom-up Approach to Data Annotation in Neurophysiology. Front Neuroinform. 2011;5:16. doi:10.3389/fninf.2011.00016.
44. Laine C, Goodman SN, Griswold ME and Sox HC. Reproducible research: moving toward research the public can really trust. Ann Intern Med. 2007;146 6:450-3. doi:10.7326/0003-4819-146-6-200703200-00154.
45. Zehl L, Jaillet F, Stoewer A, Grewe J, Sobolev A, Wachtler T, et al. Handling Metadata in a Neurophysiology Laboratory. Front Neuroinform. 2016;10:26. doi:10.3389/fninf.2016.00026.
46. Carp J. The secret lives of experiments: methods reporting in the fMRI literature. Neuroimage. 2012;63 1:289-300. doi:10.1016/j.neuroimage.2012.07.004.
47. Botvinik-Nezer R, Holzmeister F, Camerer CF, Dreber A, Huber J, Johannesson M, et al. Variability in the analysis of a single neuroimaging dataset by many teams. Nature. 2020;582 7810:84-8. doi:10.1038/s41586-020-2314-9.
48. Borghi JA and Van Gulick AE. Data management and sharing: Practices and perceptions of psychology researchers. PLoS One. 2021;16 5:e0252047. doi:10.1371/journal.pone.0252047.
49. Rao UH and Nayak U. Data Backups and Cloud Computing. In: Rao UH and Nayak U, editors. The InfoSec Handbook: An Introduction to Information Security. Berkeley, CA: Apress; 2014. p. 263-88.
50. Foster ED, Whipple EC and Rios GR. Implementing an institution-wide electronic lab notebook initiative. J Med Libr Assoc. 2022;110 2:222-7. doi:10.5195/jmla.2022.1407.
51. Khan AM, Hahn JD, Cheng WC, Watts AG and Burns GA. NeuroScholar's electronic laboratory notebook and its application to neuroendocrinology. Neuroinformatics. 2006;4 2:139-62. doi:10.1385/NI:4:2:139.
52. Higgins SG, Nogiwa-Valdez AA and Stevens MM. Considerations for implementing electronic laboratory notebooks in an academic research environment. Nat Protoc. 2022;17 2:179-89. doi:10.1038/s41596-021-00645-8.
53. Vasilevsky NA, Minnier J, Haendel MA and Champieux RE. Reproducible and reusable research: are journal data sharing policies meeting the mark? PeerJ. 2017;5:e3208. doi:10.7717/peerj.3208.
54. Assante M, Candela L, Castelli D and Tani A. Are Scientific Data Repositories Coping with Research Data Publishing? Data Science Journal. 2016;15:6. doi:10.5334/dsj-2016-006.
55. Sandstrom M, Abrams M, Bjaalie JG, Hicks M, Kennedy DN, Kumar A, et al. Recommendations for repositories and scientific gateways from a neuroscience perspective. Sci Data. 2022;9 1:212. doi:10.1038/s41597-022-01334-1.
56. Sariyar M, Schluender I, Smee C and Suhr S. Sharing and Reuse of Sensitive Data and Samples: Supporting Researchers in Identifying Ethical and Legal Requirements. Biopreserv Biobank. 2015;13 4:263-70. doi:10.1089/bio.2015.0014.
57. White T, Blok E and Calhoun VD. Data sharing and privacy issues in neuroimaging research: Opportunities, obstacles, challenges, and monsters under the bed. Hum Brain Mapp. 2022;43 1:278-91. doi:10.1002/hbm.25120.
58. Eke DO, Bernard A, Bjaalie JG, Chavarriaga R, Hanakawa T, Hannan AJ, et al. International data governance for neuroscience. Neuron. 2022;110 4:600-12. doi:10.1016/j.neuron.2021.11.017.
59. Voelkl B, Altman NS, Forsman A, Forstmeier W, Gurevitch J, Jaric I, et al. Reproducibility of animal research in light of biological variation. Nat Rev Neurosci. 2020;21 7:384-93. doi:10.1038/s41583-020-0313-3.
60. Nigri M, Ahlgren J, Wolfer DP and Voikar V. Role of Environment and Experimenter in Reproducibility of Behavioral Studies With Laboratory Mice. Front Behav Neurosci. 2022;16:835444. doi:10.3389/fnbeh.2022.835444.
61. von Ziegler L, Sturman O and Bohacek J. Big behavior: challenges and opportunities in a new era of deep behavior profiling. Neuropsychopharmacology. 2021;46 1:33-44. doi:10.1038/s41386-020-0751-7.
62. Sare RM, Lemons A and Smith CB. Behavior Testing in Rodents: Highlighting Potential Confounds Affecting Variability and Reproducibility. Brain Sci. 2021;11 4 doi:10.3390/brainsci11040522.
63. Jun JJ, Steinmetz NA, Siegle JH, Denman DJ, Bauza M, Barbarits B, et al. Fully integrated silicon probes for high-density recording of neural activity. Nature. 2017;551 7679:232-6. doi:10.1038/nature24636.
64. Steinmetz NA, Koch C, Harris KD and Carandini M. Challenges and opportunities for large-scale electrophysiology with Neuropixels probes. Curr Opin Neurobiol. 2018;50:92-100. doi:10.1016/j.conb.2018.01.009.
65. Gangadharan V, Zheng H, Taberner FJ, Landry J, Nees TA, Pistolic J, et al. Neuropathic pain caused by miswiring and abnormal end organ targeting. Nature. 2022;606 7912:137-45. doi:10.1038/s41586-022-04777-z.
66. Robbins M, Christensen CN, Kaminski CF and Zlatic M. Calcium imaging analysis - how far have we come? F1000Res. 2021;10:258. doi:10.12688/f1000research.51755.2.
67. Pnevmatikakis EA. Analysis pipelines for calcium imaging data. Curr Opin Neurobiol. 2019;55:15-21. doi:10.1016/j.conb.2018.11.004.
68. Giovannucci A, Friedrich J, Gunn P, Kalfon J, Brown BL, Koay SA, et al. CaImAn an open source tool for scalable calcium imaging data analysis. Elife. 2019;8 doi:10.7554/eLife.38173.
69. Cantu DA, Wang B, Gongwer MW, He CX, Goel A, Suresh A, et al. EZcalcium: Open-Source Toolbox for Analysis of Calcium Imaging Data. Front Neural Circuits. 2020;14:25. doi:10.3389/fncir.2020.00025.

70. Molter J, Avitan L and Goodhill GJ. Detecting neural assemblies in calcium imaging data. BMC Biol. 2018;16 1:143. doi:10.1186/s12915-018-0606-4.
71. Niso G, Botvinik-Nezer R, Appelhoff S, De La Vega A, Esteban O, Etzel JA, et al. Open and reproducible neuroimaging: From study inception to publication. Neuroimage. 2022;263:119623. doi:10.1016/j.neuroimage.2022.119623.
72. Akhtar A. The flaws and human harms of animal experimentation. Camb Q Healthc Ethics. 2015;24 4:407-19. doi:10.1017/S0963180115000079.
73. Stephens DN, Crombag HS and Duka T. The challenge of studying parallel behaviors in humans and animal models. Curr Top Behav Neurosci. 2013;13:611-45. doi:10.1007/7854_2011_133.
74. Suvorov A and Takser L. Facing the challenge of data transfer from animal models to humans: the case of persistent organohalogens. Environ Health. 2008;7:58. doi:10.1186/1476-069X-7-58.
75. Schneider CA, Rasband WS and Eliceiri KW. NIH Image to ImageJ: 25 years of image analysis. Nat Methods. 2012;9 7:671-5. doi:10.1038/nmeth.2089.
76. Cachat J, Bandrowski A, Grethe JS, Gupta A, Astakhov V, Imam F, et al. A survey of the neuroscience resource landscape: perspectives from the neuroscience information framework. Int Rev Neurobiol. 2012;103:39-68. doi:10.1016/B978-0-12-388408-4.00003-4.
77. Litvina E, Adams A, Barth A, Bruchez M, Carson J, Chung JE, et al. BRAIN Initiative: Cutting-Edge Tools and Resources for the Community. J Neurosci. 2019;39 42:8275-84. doi:10.1523/JNEUROSCI.1169-19.2019.
78. Nayak L, Dasgupta A, Das R, Ghosh K and De RK. Computational neuroscience and neuroinformatics: Recent progress and resources. J Biosci. 2018;43 5:1037-54.
79. Crutzen R, Ygram Peters G-J and Mondschein C. Why and how we should care about the General Data Protection Regulation. Psychology & Health. 2019;34 11:1347-57. doi:10.1080/08870446.2019.1606222.
80. Jwa AS and Poldrack RA. Addressing privacy risk in neuroscience data: from data protection to harm prevention. J Law Biosci. 2022;9 2:lsac025. doi:10.1093/jlb/lsac025.
81. Mittal D. CRC1158 Data Management Plan Templates (1.0). Zenodo. 2022;https://doi.org/10.5281/zenodo.6917120.
82. Brand S, Bartlett D, Farley M, Fogelson M, Hak JB, Hu G, et al. A Model Data Management Plan Standard Operating Procedure: Results From the DIA Clinical Data Management Community, Committee on Clinical Data Management Plan. Ther Innov Regul Sci. 2015;49 5:720-9. doi:10.1177/2168479015579520.
83. Merkel D. Docker: lightweight Linux containers for consistent development and deployment. Linux Journal. 2014;2014:2.
84. Kurtzer GM, Sochat V and Bauer MW. Singularity: Scientific containers for mobility of compute. PLoS One. 2017;12 5:e0177459. doi:10.1371/journal.pone.0177459.
85. Solle D. Be FAIR to your data. Anal Bioanal Chem. 2020;412 17:3961-5. doi:10.1007/s00216-020-02526-7.
86. CARPi N, Minges A and Piel M. eLabFTW: An open source laboratory notebook for research labs. J Open Source Softw. 2017;2:146.
87. Flor H, Rudy TE, Birbaumer N, Streit B and Schugens MM. Zur Anwendbarkeit des West Haven-Yale Multidimensional Pain Inventory im deutschen Sprachraum: Daten zur Reliabilitat und Validitat des MPI-D, [The Applicability of the West Haven-Yale Multidimensional Pain Inventory in German speaking countries: data on the reliability and validity of the MPI-D]. Der Schmerz. 1990;4:82-7.
88. Herrmann C, Buss U and Snait R. Hospital Anxiety and Depression Scale- Deutsche Version: Ein Fragebogen zur Erfasung von Angst une Depressivitat in der somatischen Medizin. [HADS-D - Hospital Anxiety and Depression Scale - German version: A questionnaire to assess anxiety and depression in somatic medicine]. Bern: Huber. 1995.
89. Gorgolewski KJ, Alfaro-Almagro F, Auer T, Bellec P, Capota M, Chakravarty MM, et al. BIDS apps: Improving ease of use, accessibility, and reproducibility of neuroimaging data analysis methods. PLoS Comput Biol. 2017;13 3:e1005209. doi:10.1371/journal.pcbi.1005209.
90. Covitz S, Tapera TM, Adebimpe A, Alexander-Bloch AF, Bertolero MA, Feczko E, et al. Curation of BIDS (CuBIDS): A workflow and software package for streamlining reproducible curation of large BIDS datasets. Neuroimage. 2022;263:119609. doi:10.1016/j.neuroimage.2022.119609.
91. Tauffer L, Vaz V and Dichter B. SFB1158 Metadata GUI. 2022.
92. Siegle JH, Lopez AC, Patel YA, Abramov K, Ohayon S and Voigts J. Open Ephys: an open-source, plugin-based platform for multichannel electrophysiology. J Neural Eng. 2017;14 4:045003. doi:10.1088/1741-2552/aa5eea.
93. Teeters Jeffery L, Godfrey K, Young R, Dang C, Friedsam C, Wark B, et al. Neurodata Without Borders: Creating a Common Data Format for Neurophysiology. Neuron. 2015;88 4:629-34. doi:https://doi.org/10.1016/j.neuron.2015.10.025.
94. Buccino A, Hurwitz C, Garcia S, Magland J, Siegle J, Hurwitz R, et al. SpikeInterface, a unified framework for spike sorting. eLife. 2020;9 doi:10.7554/eLife.61834.
95. Ruebel O, Tritt A, Dichter B, Braun T, Cain N, Clack N, et al. NWB:N 2.0: An Accessible Data Standard for Neurophysiology. 2019.
96. Manz T, Gold I, Patterson NH, McCallum C, Keller MS, Herr BW, 2nd, et al. Viv: multiscale visualization of high-resolution multiplexed bioimaging data on the web. Nat Methods. 2022;19 5:515-6. doi:10.1038/s41592-022-01482-7.

97. Schindelin J, Arganda-Carreras I, Frise E, Kaynig V, Longair M, Pietzsch T, et al. Fiji: an open-source platform for biological-image analysis. Nat Methods. 2012;9 7:676-82. doi:10.1038/nmeth.2019.
98. Sarkans U, Chiu W, Collinson L, Darrow MC, Ellenberg J, Grunwald D, et al. REMBI: Recommended Metadata for Biological Images-enabling reuse of microscopy data in biology. Nat Methods. 2021;18 12:1418-22. doi:10.1038/s41592-021-01166-8.
99. Moore J, Allan C, Besson S, Burel JM, Diel E, Gault D, et al. OME-NGFF: a next-generation file format for expanding bioimaging data-access strategies. Nat Methods. 2021;18 12:1496-8. doi:10.1038/s41592-021-01326-w.
100. Bourget MH, Kamentsky L, Ghosh SS, Mazzamuto G, Lazari A, Markiewicz CJ, et al. Microscopy-BIDS: An Extension to the Brain Imaging Data Structure for Microscopy Data. Front Neurosci. 2022;16:871228. doi:10.3389/fnins.2022.871228.
101. International Brain L, Aguillon-Rodriguez V, Angelaki D, Bayer H, Bonacchi N, Carandini M, et al. Standardized and reproducible measurement of decision-making in mice. Elife. 2021;10 doi:10.7554/eLife.63711.
102. Maggi S, Garbugino L, Heise I, Nieus T, Balcı F, Wells S, et al. A Cross-Laboratory Investigation of Timing Endophenotypes in Mouse Behavior. Timing & Time Perception. 2014;2 1:35-50. doi:https://doi.org/10.1163/22134468-00002007.
103. Mandillo S, Tucci V, Holter SM, Meziane H, Banchaabouchi MA, Kallnik M, et al. Reliability, robustness, and reproducibility in mouse behavioral phenotyping: a cross-laboratory study. Physiol Genomics. 2008;34 3:243-55. doi:10.1152/physiolgenomics.90207.2008.
104. Robinson L, Spruijt B and Riedel G. Between and within laboratory reliability of mouse behaviour recorded in home-cage and open-field. J Neurosci Methods. 2018;300:10-9. doi:10.1016/j.jneumeth.2017.11.019.
105. van der Naald M, Chamuleau SAJ, Menon JML, de Leeuw W, de Haan J, Duncker DJ, et al. Preregistration of animal research protocols: development and 3-year overview of preclinicaltrials.eu. BMJ Open Sci. 2022;6 1:e100259. doi:10.1136/bmjos-2021-100259.
106. King G. An Introduction to the Dataverse Network as an Infrastructure for Data Sharing. Sociological Methods and Research. 2007;36:173-99.
107. re3data.org. heiDATA. Registry of Research Data Repositories 2021;re3data.org doi:http://doi.org/10.17616/R3QW6J.
108. Kanza S and Knight NJ. Behind every great research project is great data management. BMC Research Notes. 2022;15 1:20. doi:10.1186/s13104-022-05908-5.
109. Federer LM, Lu YL, Joubert DJ, Welsh J and Brandys B. Biomedical Data Sharing and Reuse: Attitudes and Practices of Clinical and Scientific Research Staff. PLoS One. 2015;10 6:e0129506. doi:10.1371/journal.pone.0129506.
110. Pasquetto IV, Randles BM and Borgman CL. On the Reuse of Scientific Data. Data Science Journal. 2017;16:8. doi:10.5334/dsj-2017-008.
111. In: Weichbrod RH, Thompson GA and Norton JN, editors. Management of Animal Care and Use Programs in Research, Education, and Testing. Boca Raton (FL); 2018.
112. Jin IS, Yoon MS, Park C-W, Hong JT, Chung YB, Kim J-S, et al. Replacement techniques to reduce animal experiments in drug and nanoparticle development. Journal of Pharmaceutical Investigation. 2020;50 3:327-35. doi:10.1007/s40005-020-00487-8.
113. Manciocco A, Chiarotti F, Vitale A, Calamandrei G, Laviola G and Alleva E. The application of Russell and Burch 3R principle in rodent models of neurodegenerative disease: the case of Parkinson's disease. Neurosci Biobehav Rev. 2009;33 1:18-32. doi:10.1016/j.neubiorev.2008.08.002.
114. Tremoleda JL and Sosabowski J. Imaging technologies and basic considerations for welfare of laboratory rodents. Lab Anim (NY). 2015;44 3:97-105. doi:10.1038/laban.665.
115. Peng G, Privette JL, Tilmes C, Bristol S, Maycock T, Bates JJ, et al. A Conceptual Enterprise Framework for Managing Scientific Data Stewardship. Data Sci J. 2018;17:15. doi:10.5334/dsj-2018-015.
116. Abrams MB, Bjaalie JG, Das S, Egan GF, Ghosh SS, Goscinski WJ, et al. A Standards Organization for Open and FAIR Neuroscience: the International Neuroinformatics Coordinating Facility. Neuroinformatics. 2022;20 1:25-36. doi:10.1007/s12021-020-09509-0.
117. Sansone S-A, McQuilton P, Rocca-Serra P, Gonzalez-Beltran A, Izzo M, Lister AL, et al. FAIRsharing as a community approach to standards, repositories and policies. Nature Biotechnology. 2019;37 4:358-67. doi:10.1038/s41587-019-0080-8.
118. European Open Science Cloud. Nat Genet. 2016;48 8:821. doi:10.1038/ng.3642.
119. Arendt T. Concepts and services for the homogenization and management of file structures in collaborative neuroscientific projects [Data set]. Zenodo. 2021; doi:10.5281/zenodo.
120. Colomb J, Arendt T, Mittal D and Sehara K. Folder structure template for research repositories (2.0). Zenodo. 2020; doi:10.5281/zenodo.4314361.
121. Harris PA, Taylor R, Thielke R, Payne J, Gonzalez N and Conde JG. Research electronic data capture (REDCap)--a metadata-driven methodology and workflow process for providing translational research informatics support. J Biomed Inform. 2009;42 2:377-81. doi:10.1016/j.jbi.2008.08.010.
122. Marcus DS, Olsen TR, Ramaratnam M and Buckner RL. The Extensible Neuroimaging Archive Toolkit: an informatics platform for managing, exploring, and sharing neuroimaging data. Neuroinformatics. 2007;5 1:11-34. doi:10.1385/ni:5:1:11.
123. Das S, Zijdenbos AP, Harlap J, Vins D and Evans AC. LORIS: a web-based data management system for multi-center studies. Front Neuroinform. 2011;5:37. doi:10.3389/fninf.2011.00037.

124. Rupp R, Jersch P, Schuld C, Schweidler J, Benning N, Knaup P, et al. Das deutschlandweite, webbasierte ParaReg-Register zur lebenslangen Dokumentation von Querschnittgelähmten – Datenmodell, rechtlich-ethische Voraussetzungen und technische Implementierung. Das Gesundheitswesen. 2021;83:S18-S26. doi:10.1055/a-1538-6537.
125. Poline JB, Kennedy DN, Sommer FT, Ascoli GA, Van Essen DC, Ferguson AR, et al. Is Neuroscience FAIR? A Call for Collaborative Standardisation of Neuroscience Data. Neuroinformatics. 2022; doi:10.1007/s12021-021-09557-0.
126. Jollans L, Boyle R, Artiges E, Banaschewski T, Desrivières S, Grigis A, et al. Quantifying performance of machine learning methods for neuroimaging data. Neuroimage. 2019;199:351-65. doi:10.1016/j.neuroimage.2019.05.082.
127. Stanford SC. The Open Field Test: reinventing the wheel. J Psychopharmacol. 2007;21 2:134-5. doi:10.1177/0269881107073199.
128. Uslu ZSA. Recent advancements in behavioral testing in rodents. MethodsX. 2021;8:101536. doi:10.1016/j.mex.2021.101536.
129. Kuo JY, Denman AJ, Beacher NJ, Glanzberg JT, Zhang Y, Li Y, et al. Using deep learning to study emotional behavior in rodent models. Front Behav Neurosci. 2022;16:1044492. doi:10.3389/fnbeh.2022.1044492.
130. van Dam EA, Noldus LPJJ and van Gerven MAJ. Deep learning improves automated rodent behavior recognition within a specific experimental setup. Journal of Neuroscience Methods. 2020;332:108536. doi:https://doi.org/10.1016/j.jneumeth.2019.108536.
131. Spink AJ, Tegelenbosch RA, Buma MO and Noldus LP. The EthoVision video tracking system--a tool for behavioral phenotyping of transgenic mice. Physiol Behav. 2001;73 5:731-44. doi:10.1016/s0031-9384(01)00530-3.
132. Lopes G and Monteiro P. New Open-Source Tools: Using Bonsai for Behavioral Tracking and Closed-Loop Experiments. Front Behav Neurosci. 2021;15:647640. doi:10.3389/fnbeh.2021.647640.
133. Peirce JW. Generating Stimuli for Neuroscience Using PsychoPy. Front Neuroinform. 2008;2:10. doi:10.3389/neuro.11.010.2008.
134. Mitteer DR and Greer BD. Using GraphPad Prism's Heat Maps for Efficient, Fine-Grained Analyses of Single-Case Data. Behav Anal Pract. 2022;15 2:505-14. doi:10.1007/s40617-021-00664-7.
135. Mathis A, Mamidanna P, Cury KM, Abe T, Murthy VN, Mathis MW, et al. DeepLabCut: markerless pose estimation of user-defined body parts with deep learning. Nat Neurosci. 2018;21 9:1281-9. doi:10.1038/s41593-018-0209-y.
136. Sturman O, von Ziegler L, Schläppi C, Akyol F, Privitera M, Slominski D, et al. Deep learning-based behavioral analysis reaches human accuracy and is capable of outperforming commercial solutions. Neuropsychopharmacology. 2020;45 11:1942-52. doi:10.1038/s41386-020-0776-y.
137. Mathis MW and Mathis A. Deep learning tools for the measurement of animal behavior in neuroscience. Curr Opin Neurobiol. 2020;60:1-11. doi:10.1016/j.conb.2019.10.008.
138. Berman GJ. Measuring behavior across scales. BMC Biol. 2018;16 1:23. doi:10.1186/s12915-018-0494-7.
139. Martone M, Gerkin R and Moucek R. NIX –Neuroscience information exchange format [version 1; not peer reviewed]. F1000Research. 2020:9:358 doi:10.7490/f1000research.1117858.1.
140. Rübel O, Tritt A, Ly R, Dichter BK, Ghosh S, Niu L, et al. The Neurodata Without Borders ecosystem for neurophysiological data science. eLife. 2022;11:e78362. doi:10.7554/eLife.78362.
141. Halchenko Y, Meyer K, Poldrack B, Solanky D, Wagner A, Gors J, et al. DataLad: distributed system for joint management of code, data, and their relationship. The Journal of Open Source Software. 2021;6:3262. doi:10.21105/joss.03262.
142. Davison AP, Brüderle D, Eppler J, Kremkow J, Muller E, Pecevski D, et al. PyNN: A Common Interface for Neuronal Network Simulators. Front Neuroinform. 2008;2:11. doi:10.3389/neuro.11.011.2008.
143. Garcia S, Guarino D, Jaillet F, Jennings T, Pröpper R, Rautenberg P, et al. Neo: an object model for handling electrophysiology data in multiple formats. Frontiers in Neuroinformatics. 2014;8 doi:10.3389/fninf.2014.00010.
144. Bryant M, Blanke T, Hedges M and Palmer R. Open Source Historical OCR: The OCRopodium Project. In: Berlin, Heidelberg, 2010, pp.522-5. Springer Berlin Heidelberg.
145. Donnelly M, Jones S and Pattenden-Fail JW. DMP Online: A Demonstration of the Digital Curation Centre's Web-Based Tool for Creating, Maintaining and Exporting Data Management Plans. In: Berlin, Heidelberg, 2010, pp.530-3. Springer Berlin Heidelberg.
146. Esteban O, Markiewicz CJ, Blair RW, Moodie CA, Isik AI, Erramuzpe A, et al. fMRIPrep: a robust preprocessing pipeline for functional MRI. Nat Methods. 2019;16 1:111-6. doi:10.1038/s41592-018-0235-4.
147. Schmidt U, Weigert M, Broaddus C and Myers G. Cell Detection with Star-Convex Polygons. In: *Medical Image Computing and Computer Assisted Intervention – MICCAI 2018* (eds Frangi AF, Schnabel JA, Davatzikos C, Alberola-López C and Fichtinger G), Cham, 2018// 2018, pp.265-73. Springer International Publishing.
148. Krull A, Buchholz T-O and Jug F. Noise2Void - Learning Denoising From Single Noisy Images. 2019 IEEE/CVF Conference on Computer Vision and Pattern Recognition (CVPR). 2019:2124-32.
149. Stringer C, Wang T, Michaelos M and Pachitariu M. Cellpose: a generalist algorithm for cellular segmentation. Nat Methods. 2021;18 1:100-6. doi:10.1038/s41592-020-01018-x.

150. Labus JS, Naliboff B, Kilpatrick L, Liu C, Ashe-McNalley C, Dos Santos IR, et al. Pain and Interoception Imaging Network (PAIN): A multimodal, multisite, brain-imaging repository for chronic somatic and visceral pain disorders. Neuroimage. 2016;124 Pt B:1232-7. doi:10.1016/j.neuroimage.2015.04.018.
151. Markiewicz CJ, Gorgolewski KJ, Feingold F, Blair R, Halchenko YO, Miller E, et al. The OpenNeuro resource for sharing of neuroscience data. Elife. 2021;10 doi:10.7554/eLife.71774.
152. Alam S, Bartolome J, Bassini S, Carpene M, Cestari M, Combeau F, et al. Fenix: Distributed e-Infrastructure Services for EBRAINS. In: *Brain-Inspired Computing* (eds Amunts K, Grandinetti L, Lippert T and Petkov N), Cham, 2021// 2021, pp.81-9. Springer International Publishing.
153. Dillen M, Groom Q, Agosti D and Nielsen L. Zenodo, an Archive and Publishing Repository: A tale of two herbarium specimen pilot projects. Biodiversity Information Science and Standards. 2019;3 doi:10.3897/biss.3.37080.
154. Hahnel M. Referencing: The reuse factor. Nature. 2013;502 7471:298. doi:10.1038/502298a.
155. Gomez-Diaz T and Recio T. Research Software vs. Research Data II: Protocols for Research Data dissemination and evaluation in the Open Science context. F1000Res. 2022;11:117. doi:10.12688/f1000research.78459.2.
156. Wallace CT, St Croix CM and Watkins SC. Data management and archiving in a large microscopy-and-imaging, multi-user facility: Problems and solutions. Mol Reprod Dev. 2015;82 9:630-4. doi:10.1002/mrd.22538.

Suppl.1

Click here to access/download
Supplementary Material
Suppl1.pdf

Suppl.2

Click here to access/download
Supplementary Material
Suppl2.pdf

Click here to access/download
Supplementary Material
Supp3.1_Tandem_Projects.pdf

Suppl.3.2

Click here to access/download
Supplementary Material
Supp3.2_Human_Projects.pdf

Suppl.3.3

Click here to access/download
Supplementary Material
Supp3.3_Animal_Projects.pdf

Personal Cover
Click here to access/download;Personal Cover;Mittal_Cover_RespToReviewers.pdf

HEIDELBERG FACULTY OF MEDICINE
Pharmacology Institute | Im Neuenheimer Feld 366 | 69120 Heidelberg

Deepti Mittal, MS

Data Manager, Heidelberg Pain Consortium, Pharmacology Institute, Heidelberg University

Medical Faculty, Heidelberg University

Phone: +49 15203531055
Secretary office: +49 6221 54 16601
deepti.mittal@pharma.uni-heidelberg.de

www.sfb1158.de

Heidelberg, February 20th, 2023

Subject: Manuscript GIGA-D-22-00262: Resubmission by Deepti Mittal; Rebecca Mease; Thomas Kuner; Herta Flor; Rohini Kuner and Jamila Andoh

Dear Editors,

Thank you very much for the opportunity to further revise the manuscript. We have addressed the issues raised by the reviewers on this manuscript.

Attached please find our detailed responses to the editor and reviewer, along with a revised version of the manuscript.

Sincerely,
Deepti Mittal
Rebecca Mease
Thomas Kuner
Herta Flor
Rohini Kuner
Jamila Andoh & the Consortium

Reviewer reports:

1. Reviewer #1:

The authors describe a multistage plan that they implemented to improve data standards and management for the Heidelberg Pain Consortium. Having done similar work, I can tell that a great deal of thought and work went into creating this management program, however the paper will need substantial refactoring for that effort to shine through. The pieces of a great paper are already here, they are just difficult to follow.

Major comments:

1.1 Comment: pg 3, lines 31-33 "Implementing effective data management strategies and ethical rules for the reuse and sharing of high-quality data reduces redundant research, optimizes public research funding and reduces animal use."
This seems like a significant claim supporting the need for RDMs, but has no citation. Is there evidence that RDMs reduce animal use or reduce redundant research? What does optimized public research funding look like? This statement is surrounded by several other assertions with little explanation, e.g.: "A clear set of regulations and guidelines must be established before sharing human data gathered from clinical or non-clinical populations. Specific rules addressing privacy issues, established processes for data protection, data use and reuse, and the preservation of sensitive data are required. It is essential to make data accessible and understandable to remote (or future) collaborators in order to maximize the potential of existing algorithms and tools and accelerate the creation of new ones."

We thank the reviewer for the comments. We now reformulated our sentence and added some references as follows, see p. 27 lines 26-40:
"Implementing effective data management strategies and ethical rules for the reuse and sharing of high-quality data may reduce redundant research [5, 6]. One of DM goals is to make study results freely available through open-access publishing. By investing in collaborative projects with long-term goals, it ensures that the data are organized in a way that makes them easily accessible and retrievable for future use. This makes it easier and faster to develop new research projects, as well as to replicate or build on existing studies, which should have a direct impact on public research funding (https://sor.senate.ca.gov/sites/sor.senate.ca.gov/files/0842%20policy%20matters%20Research%2003.18%20Final.pdf). Moreover, effective DM strategies can also enable to optimize public research funding by pooling resources and infrastructure from multiple sources and bringing together experts from universities, research institutes, and other community organizations to work on long-term interdisciplinary projects. Furthermore, by ensuring proper RDM, researchers should be able to reduce animal use. For example, making informed decisions about which animal models to use for their studies should enable to use the same animals for multiple experiments, instead of having to continuously use new animals for each study. In addition, sharing previously acquired data with adequate metadata, or reuse of control group data from similar studies can avoid repeating in vivo work [1-4]."

1.2 Comment: I would like to see the introduction more thoroughly discuss the problems with data management and elaborate on these asserted solutions.

We agree with the reviewer's comment. We now added a section on data management challenges in the introduction section, starting with the common data management challenges across projects (including challenges due to the diversity in data types, challenges due to diversity in acquisition, preprocessing, and analysis approaches, metadata challenges, data storage and volume, challenges in data documentation, data sharing and dissemination challenges and challenges due to sensitive data). Then we present some RDM challenges for specific projects (including behavioral experiments, electrophysiology with high-density probes, large-scale in vivo two-photon calcium imaging, human-animal tandem projects), see p. 4 lines 6-39, p. 5-7, p. 8 lines 1-13.

1.3 Comment: There are multiple other places throughout the text where there are ideas stated as facts without references or elaboration, that I would like to see evidence for, or at least a discussion of.
For e.g. pg 13, lines 1-2 "There is, furthermore, a lack of efficient software to adequately segregate and maintain control over sensitive data. It is important to provide researchers working with sensitive data or

samples with truly "useful" tools that do not require pre-existing, in-depth knowledge of legal and ethical requirements, or time to delve into the details."

Following the reviewer's comment, we now added some information and references in the discussion section entitled "Sharing sensitive data from human projects", p. 30 lines 26-40, p. 31, p. 32 lines 1-7.

*"**Sharing sensitive data from human projects***

The sharing of human data gathered from clinical or non-clinical populations in neuroscience research is essential for advancing science and producing important public health benefits. A clear set of regulations and guidelines must be established before sharing human data gathered from clinical or non-clinical populations. Specific rules addressing privacy issues, established processes for data protection, data use and reuse, and the preservation of sensitive data are required. It is essential to make data accessible and understandable to remote (or future) collaborators in order to maximize the potential of existing algorithms and tools and accelerate the creation of new ones. Regulations and guidelines should ensure that the data are used for the purpose for which they were gathered and protect the rights of participants in the research. These guidelines should also cover how data should be collected, stored, shared, and destroyed. They also specify the types of data that must be kept confidential and the appropriate methods for handling and safeguarding the data. They should also ensure that the data are secure, kept confidential, and not used for marketing or other commercial purposes. Additionally, regulations should ensure that the data are used responsibly and that they are not used to discriminate against people with disabilities or other vulnerable populations. Ethical rules for the reuse and sharing of data should be based on the principle of informed consent. This includes obtaining consent from the original data collectors or from research participants, as well as obtaining permission from any third parties involved in the data collection. Researchers should also seek to minimize the risk of data misuse or breach of confidentiality, and any data that are shared should be done so in a secure manner.

Additionally, there is a lack of efficient software programs to adequately segregate and maintain control over sensitive data. It can be difficult to develop effective software that is secure, user-friendly, and cost-effective. The maintenance of such software requires a significant investment of resources, and often there is a lack of funding available for such measures. Finally, the adoption of such software requires investment in training and resources, which many organizations may be unwilling to do. The legal and ethical requirements surrounding the use of sensitive data are often complex and difficult to understand, leading to confusion and ambiguity about the best way to protect them. It is important to provide researchers working with sensitive data or samples with truly "useful" tools that do not require pre-existing, in-depth knowledge of legal and ethical requirements, or time to delve into the details. Such tools are important to ensure that sensitive data are protected and securely stored. Use of such tools can help researchers to make informed decisions about how to best use and manage sensitive data, allowing them to work with it in an ethical and responsible manner. Finally, these tools can help to reduce the risk of data breaches and data misuse, which can have serious consequences for the people and organizations whose data are affected. By providing such tools, researchers can focus on their research and not on legal and ethical considerations, thus saving their time and resources.

There are several software tools that can be used to maintain sensitive patient data in neuroscience research. Some examples include a web-based platform REDCap (Research Electronic Data Capture) [121], an open-source imaging informatics platform XNAT (XNAT Central) [122], LORIS: Longitudinal Online Research and Imaging System [123] etc. It is important to note that the security features of these tools may vary and should be evaluated before use. In addition to software tools, secure data storage and access protocols should also be in place to ensure that sensitive patient data are protected.

We are currently expanding our collaborative efforts by creating a data infrastructure platform that will establish a GDPR-compliant data registry (PainReg-registry, based on the Germany-wide ParaReg registry (www.parareg.de) [124] for human volunteers. To facilitate cross-project data merging, a core clinical data set will be defined. This entails assigning a unique identifier to each study participant that is shared by all projects, allowing researchers to determine whether the volunteer participated in multiple projects. This will allow organizations to share a same pool of volunteers for multiple studies, resulting in less redundant data acquisition. This can result in cost and time savings, as well as increased data collection accuracy. We, for example, experienced that a same study participant could be tested twice, and was assigned different IDs (belonging to different projects), resulting in redundant data acquisition and therefore unnecessary increased costs, particularly for genetic analysis.

Furthermore, the data registry will ensure that data privacy regulations are strictly followed by obtaining participants' consent to access data for secondary or follow-up studies. This will also include an identity management feature to limit access to authorized users. The registry will contain a wide range of data, including brain imaging, genetic, cognitive, and physiological data. This collaborative work will be

coordinated by the consortium's future data infrastructure project, which will be tasked with implementing, testing, optimizing, and standardizing data analysis procedures and models that will be utilized in all projects.

1.4 and pg 13, lines 26 - 29; Why do available commercial cloud storage solutions come with unreliable and slow backup and restoration services, as well as no obvious access paths or interfaces for easy migration onto the analysis platforms?

Following the reviewer's comment we now added the following information p. 8 lines 27-38:
"Many commercial cloud storage solutions are designed primarily for convenience and cost savings rather than for robust backup and restoration services. As a result, these services often lack the features necessary for reliable and fast backup and restoration, such as automated data backups, point-in-time recovery, and incremental backups. Additionally, many cloud storage solutions lack the necessary APIs, scripts, and tools that would allow for easy migration of data onto the analysis platforms. This makes data migration difficult and inefficient, and it can significantly slow down the analysis process.
Assuring access to secure and optimal storage solutions that can be integrated with workflows encompassing data acquisition, intermediate analysis, and archiving is thus a major challenge. Creating backups and storing multiple copies of large volumes of datasets, the need for collaborative and parallel access by multiple people, and use across a diverse range of computational workflows all pose major challenges to storage servers."

1.5 pg 15, lines 13 - 19;
We now added a reference p. 9 line 19:
Higgins SG, Nogiwa-Valdez AA, Stevens MM. Considerations for implementing electronic laboratory notebooks in an academic research environment. Nat Protoc. 2022 Feb;17(2):179-189. doi: 10.1038/s41596-021-00645-8. Epub 2022 Jan 14. PMID: 35031789.

1.6 pg 16, lines 4-5; Another significant issue with submitting data to general repositories is that they do not have enough support for certain types and formats of data.

Following the reviewer's comment, we now added some information and some references p. 10 lines 13-28:
Another significant issue with submitting data to general repositories is that the latter might not have adequate support for certain types and formats of data [54, 55]. For example, if the data are in a non-standard format, the repository may not be able to process it correctly or even accept it. Additionally, repositories may not have specialized tools or services to help researchers convert, organize or analyze the data. This can be especially problematic for data that are highly specific, such as medical records or geospatial data. Without the necessary support, researchers may be unable to make full use of the data or even access them. Additionally, general repositories may not have the same level of curation and organization as a specialized repository, which can make it harder for researchers to evaluate the quality and relevance of the data. This can lead to a lack of reproducibility and increased difficulty in building on previous research. Therefore, it is recommended for researchers to submit their data to specialized repositories that are tailored to their specific field. Neuroscience specific repositories, on the other hand, are specifically designed to accommodate the unique needs of neuroscience data (openNeuro). They are often managed by experts in the field and have the necessary infrastructure to ensure the safe and secure storage of data. Furthermore, they often provide additional services such as data analysis, curation and visualization tools, which allow researchers to better understand and use the data.

1.7 pg 4,lines 2-5 "While the majority of collaborative research consortiums collect a wide variety of multidimensional datasets, the majority of these datasets are typically inadequate for modern research methods and infrastructure." As above, I'm interested in how this is quantified, or if there is a reference, and to have a more complete discussion of this idea.

Following the reviewer's suggestion, we now added some information and some references p. 6 lines 1-22:

“The availability of robust neuroscience resources such as high-performance computing (HPC) clusters [23-25], modern workflow technologies (e.g., Galaxy [26], Snakemake [27] etc.), cloud-enabled storage and computing infrastructures (e.g., Amazon AWS, Google Cloud [28]), secure databases [29], repositories [30], and analysis platforms are fundamentally changing how research in neuroscience is communicated and linked to existing raw data and findings [31]. Such tools are allowing researchers to utilize diverse techniques and produce massive amounts of high-dimensional data (large sample size, various models and conditions), which provides greater statistical power and the opportunity to do more robust secondary data analysis [32]. However, the data-driven neuroscience approach, as a whole, is questioned by a number of technical issues that must be addressed before it can be fully realized.

While the majority of collaborative research consortiums collect diverse multidimensional datasets, one of the primary challenges is that the majority of these datasets are typically inadequate for modern research methods and infrastructure [33] . Before committing to any of these tools for processing and analysis of collected datasets, it is important to understand data in terms of the number, volume, size and complexity of data, the types and formats of data, and the accuracy and completeness of the datasets. Despite similarities in experimental design, researchers in neuroscience experiments frequently organize and describe their data in their own way, even within the same research group. As a result, datasets collected from different research groups for a single project addressing the same scientific question may not be in formats suitable for comparison and pooling, limiting their interoperability and reuse. The data formats collected in each project are typically determined by the acquisition and measurement method, intermediate pre-processing, or analysis software. This can lead to data and metadata being stored in different locations.”

1.8 Comment: Beginning on line 27 of page 4, the introduction veers into describing many technical details of specific studies. In my first read-through, I actually thought that I had accidentally flipped to a different paper. None of these details seem relevant to the larger concept of creating an effective RDM strategy, and are very disorienting. I would remove them entirely.

We agree with the reviewer’s comment and have reorganized the introduction section, which is now more focused on presenting RDM challenges (see also comment 1.2).

1.9 Comment: Figure 1. In order to understand how the authors have implemented an RDM plan, it seems important for the reader to understand the basic structure of the consortium, however, I do not understand how to interpret figure 1. It appears that the animal projects somehow encapsulate all the others? Are the relative sizes of the colored portions meaningful? The number of tandem projects is half that of animals, which seems to agree with the peach width being smaller than the green. However there are only 4 human projects for the very large yellow section, and 1 for the black. I would drop this figure entirely. I think Figure 2 does a much better job of describing the consortia, and is much easier to read. Further, pg 8, lines 5 - 12 are a near copy of the legend for Figure 1, but refer to Figure 2 A anyway.

Following the reviewer’s comment we now removed figure 1 and edited the “old” figure 2 (now figure 1) and adapted it to the current structure of the consortium.

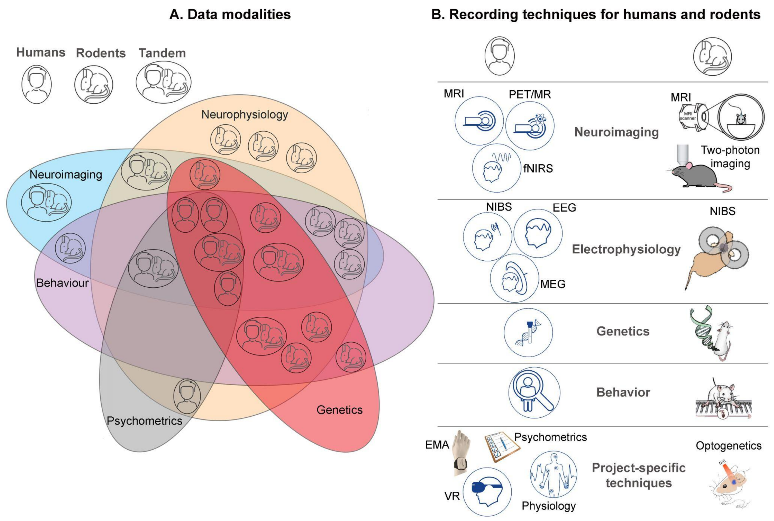

Figure 1: The Heidelberg Pain Consortium investigates various populations: humans, rodents, and tandem using various modalities: neuroimaging, neurophysiology, behavior, psychometrics, and genetics (A). Each data modality can be recorded using different techniques: MRI= magnetic resonance imaging; PET/MR: positron emission tomography/magnetic resonance; fNIRS: functional near infrared spectroscopy; NIBS: non-invasive brain stimulations; EEG: electroencephalography; MEG: magnetoencephalography; Genetics; Two-photon imaging; Behavior; EMA: ecological momentary assessment; psychometrics; physiology; VR: virtual reality; optogenetics (B).

1.10 Comment: Beginning on pg 6, line 35 to pg 7 line 16; and then again beginning on pg 10, line 34 to pg 18, line 16, this entire text is general background of problems in data management, and specifically around the particular challenges of this consortia. It is altogether a much better introduction than the existing introduction. It covers all of the missing context, as well as discussing the types of studies the Heidelberg Pain Consortium needs to work with without delving into over technical details. I recommend excising this from the results and using it to replace everything after the second paragraph of the introduction. With minor adjustments for grammar, it will be much better there. The move will also make your actual results, which currently don't begin until pg 18, line 18, much easier to find and follow.

We thank the reviewer for the suggestion and we now reorganized the introduction which contains the challenges section (see also comment 1.2).

1.11 Comment: In the results (beginning pg 18, line 18), there are several sections where important discussion points are interspersed with the results, making it difficult to tell which are the work of the consortium, and which are simply informational. It would greatly improve readability if these paragraphs were pulled out and integrated into a discussion section, which doesn't currently exist. As I'm finding it difficult to tell which parts are your work, below is my best guess at the paragraphs that should be moved, or dropped:
pg 19, lines 27 - 32
pg 20, lines 1 - 12
pg 21 line 1 - pg 22 line 18
pg 22 lines 19 - 29 (excluding the title, which should be moved to line 30), pg 22 line 34 - pg 23 line 5
pg 23 lines 18 - 28
pg 26 line 10 - pg 27 line 6
pg 27 line 31 - pg 28 line 1 (excluding the title, which should be moved to line 2)
pg 30 line 33 - pg 32 line 21
For the "Recommended data standards for different data types", "Animal neuroimaging data standardization", and "Data processing, analysis, and visualization" sections, the results and discussion pieces are not as easily pulled out as paragraphs, so I have not listed each individual sentence here.

Instead, I would suggest looking at these sections with an eye towards picking out which are results of something your consortium worked on, and which are expounding on ideas or comparing it to other work. Clearly moving all of those to the discussion will require some editing to make it make sense, but I think that if you make the discussion follow the same minor heading structure you have in the results it will be a relatively painless change. The first part of the current results: pg 5, line 23 - pg 6 line 34 would be very good if moved to be the first few paragraphs of the discussion, but are too vague to be part of the results.

We thank the reviewer for the comments. We have now restructured the RDM Implementation (section II in the manuscript) to highlight the strategies used by our consortium. We also added a discussion section.

1.12 Comment: pg 6, line 37 - pg 7 line 5. "We specifically describe our ongoing RDM efforts, which are divided into two sections: 1) RDM Planning Phase: Identifying common RDM procedures across consortium projects; Evaluating common data management challenges; Special RDM challenges in specific projects; 2) RDM Implementation Phase: resource allocation decisions and implementation of key resources and 3) Data Dissemination (data management continues even after the end of the funding period)." A minor typo is that it should say three sections, not two. Rather more importantly, both here and in the conclusion, it lists three phases, but phase three does not appear in the actual text. It is not described along with the others in the conclusion, or mentioned at all in the results.

We now revised the manuscript and divided the main text into two main sections: section 1: RDM planning phase and section II: RDM implementation, followed by a discussion.

Minor comments:

1.13 Comment: SBF is never defined, and the authors seem to use SFB1158, SFB and SFB 1158 as synonyms, but I'm not sure if that is the intent.

Thank you for pointing this out. We have now homogenized the terms and use CRC in the entire manuscript. We also added the following information in the funding section p. 40, lines 35-37:

"Collaborative Research Centers (CRC; short SFB for German 'Sonderforschungsbereich') are university research projects which are funded by the German Research Foundation (DFG), generally for a period of up to 12 years. https://www.dfg.de/foerderung/programme/koordinierte_programme/sfb/"

1.14 Comment: I would move all of the funding information on pg 4 to the funding section, it's distracting here

We now moved the funding section to the end of the manuscript p. 40, lines 35-40.

1.15 Comment: There are several minor grammar issues. I am not copying them all here, as I'm sure they'll be fixed in the rearrangement, but:
pg 3 line 26 should be 'cooperative'

We corrected now all typos and the entire manuscript was meticulously proofread.

1.16 Comment: pg 6, line 7; its unclear what "progressively practical RDM approaches" means

We apologize for the lack of clarity. We aimed to emphasize how practical our RDM efforts are in terms of allocating resources and creating new services while taking pre-existing services into account.
We now clarified the RDM approach p. 30 lines 1-8:
"We also focused on developing RDM strategies that included joint efforts and cooperation between consortium members and other large-scale consortiums and collaborative centers within Germany. We acknowledge the common issue of data organization for different projects in collaborative centers. Joint efforts were made for the development of a data organization strategy that works for most of the projects within the consortium working in similar research areas. Our main goal was to engage more directly in several overlooked aspects of managing data in a large collaborative consortium while keeping the global

neuroscience community in mind. We encouraged CRC 1158 projects to utilize logical file and folder templates to support systematic data organization."

1.16 Comment: pg 7, line 1 should be 'three'

The reviewer is correct. In the current version we however decided to have two phases and one discussion (see also comment 1.12)

1.18 Comment: pg 7, line 21 'can' should be 'could'

We have now removed the previous sentence.

1.19 Comment: pg 11, lines 23-26, this sentence doesn't seem to finish "In cases where a laboratory produces a large dataset from a single experiment, and the collected metadata are too complex and stored in multiple files having different formats that are only read by the acquisition software or by customized codes written for internal use."

We now reformulated the sentence as follows p. 7 lines 36-40:
"Moreover, a laboratory can generate a large dataset from a single experiment or a single dataset from multiple experiments, and the collected metadata can be very complex and stored in multiple files with different formats only readable by the acquisition software or by customized codes written for internal use. In such cases, a consolidated strategy for unifying data into a single format that can be read by a variety of software applications and analyzed in an efficient and reproducible manner is necessary."

1.20 Comment: pg 12, lines 17 - 12, this sentence/paragraph seems to be missing words/doesn't finish "Although many advances have been made regarding organization, annotation, and description of research datasets, for example using the Brain Imaging Data Structure (BIDS, https://smex-ctp.trendmicro.com:443/wis/clicktime/v1/query?url=https%3a%2f%2fbids.neuroimaging.io%2f&umid=69f29401-5290-40bc-943f-fc4fb21e8086&auth=9ed9a254ae8a0a504cee5b89eb68d1de87cd0c46-a38d2b534d1942c51ddbf5754176077e75f11c73) for neuroimaging data standards [10] EEG-BIDS for neurophysiology data (e.g., electroencephalography), [11], or MEG-BIDS for magnetoencephalography data [12], whereas other data modalities (e.g., EMA) do not have existing standards yet.

We thank the reviewer for his feedback. We have now rephrased the sentence as follows p. 5 lines 26-28:
"Although many advances have been made regarding the organization, annotation, and description of research datasets, there is still much work to be done to ensure that datasets are fully standardized and can be accurately shared and reused [18]."

1.21 Comment: pg 15, lines 4 - 7, this sentence seems to me missing words: "However, while choosing an electronic laboratory notebook for a large-scale neuroscience consortium spanning diverse experimental protocols, the availability of clear documentation and application-centric features become an overarching issue."

We apologize for the lack of clarity. We have now rephrased the sentence as follows p. 9 lines 3-5:
"For a large-scale neuroscience consortium spanning diverse experimental protocols, it is important to select an ELN that can provide comprehensive support for a wide range of experimental protocols and provides flexibility to add domain-specific features if required [51]."

2. **Reviewer #2**

2.1 Comment: The authors of the manuscript "Data management strategy for a Collaborative Research Centre" take the reader on a timely tour de force through the various aspects surrounding the establishment of RDM measures in a large, collaborative research project spanning multiple highly heterogeneous subprojects and partners. I found the topic of the paper highly interesting and engaging, as it is based on the experiences and procedures encountered in a real-world scenario. The challenges and solutions are presented in a clear, well-written and structured way in two overarching chapters, respectively, that make the article enjoyable to read. Also, I found the breadth of challenges well captured.
While I am enthusiastic to see the manuscript published, I have several points and suggestions I would like to put up for consideration.
As a general point, I found the article quite lengthy due to a lot of detail that was put into the description of the individual challenges. At the same time, I found many challenges described in the planning phase to be rather high-level statements, and I was often hoping to have these more embedded into the actual problems encountered in the SFB to give illustrative examples. I would like suggest to the authors to reconsider if they find specific examples of some of the more abstract challenges that were actually encountered (similar, e.g., to the last part in "planning" section about specific RDM challenges). The reasoning here is that I find the strong point of this article that it comes from actual experience, and I found this not ideally reflected in some of the more abstract descriptions. An example illustrating this suggestion is given in the detailed points below (p10, l31-32). To compensate, perhaps some of the details in the manuscript could be shorted. Also, as a general point I found that many of the existing tools/services/... were not referenced (by either publication, DOI, RRDI, https link or at least some pointer to the resource).

We thank the reviewer for the helpful comments. Following the feedback from reviewer 1 and reviewer 2 we have now restructured the whole manuscript. We have moved the data management challenges in the introduction, restructured section 1 (planning phase) and section 2 (implementation phase), and added some examples and some references. We also added a discussion section.

2.2 Comment: - p5, Figure 1: I did not find this illustration very helpful, I think a more standard 2D representation with the individual groups and links between groups forming tandems would be better understandable.

Following the feedback from reviewer 1 and reviewer 2, we now removed the old Figure 1 and edited the old Figure 2 (now Figure 1).

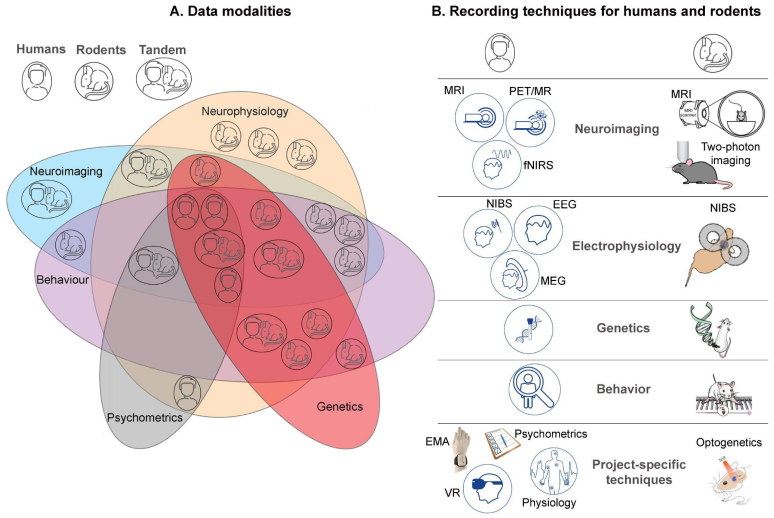

Figure 1: The Heidelberg Pain Consortium investigates various populations: humans, rodents, and tandem using various modalities: neuroimaging, neurophysiology, behavior, psychometrics, and genetics (A). Each data modality can be recorded using different techniques: MRI= magnetic resonance imaging; PET/MR: positron emission tomography/magnetic resonance; fNIRS: functional near infrared spectroscopy; NIBS:

non-invasive brain stimulations; EEG: electroencephalography; MEG: magnetoencephalography; Genetics; Two-photon imaging; Behavior; EMA: ecological momentary assessment; psychometrics; physiology; VR: virtual reality; optogenetics (B).

2.3 Comment: - p6, l13: I think it would be extremely useful for other consortia to know more about the day-to-day tasks of the data manager, in particular in terms of the job profile -- what background is more useful: neuroscience, computer science, software engineer, project planning/organization? As the job description of a "data manager" is evolving, it would be great to hear what tasks made this SFB successful in cross-group RDM.

We have now added a sub-section "Data stewards, Community engagements and collaborations" in the discussion section p. 28 lines 37-40, p. 29, p. 30 lines 1-24.

"Data stewards, Community engagements and collaborations

Data managers and stewards are essential for implementation of data policy and governance procedures, especially for large consortiums [115]. These positions are well-suited to individuals with a background in research or computer science, bioinformatics, and strong communication skills. Furthermore, depending on the needs of the consortium, the candidate should have experience developing high-throughput analysis pipelines, domain-specific data structures and standards, open access publishing, and modern data science approaches, high-performance computing environments, cloud computing, data security, and databases, among other things. The role of data manager is diverse, and it works in close collaboration with core computing and library resources to streamline access to the consortium's common research data infrastructure, which is available at the host institutions of participating labs.

Data managers should also support ongoing research, advise on best practices for data handling, and stay informed about current developments in the RDM field. Furthermore, they should act as an important liaison between consortium researchers, collaborators, the university's RDM planning group and computing center, and community organizations. They aid in bridging the gap between the lab-based scientists and the available technical infrastructure and services. Direct assistance in daily tasks such as data organization, tool selection, workflow development, and standardization is indeed beneficial for individual researchers and research groups. Addressing data management tasks relatively early in research timelines is necessary to make the research process more efficient, and to ensure the interoperability and reusability of data sets. The expert guidance of existing infrastructure and resources, such as scientific repositories, databases, legal and ethical issues, etc., is also necessary to promote an effective data-sharing strategy.

Data managers must maintain consistent communication with various research groups and other similar consortiums to establish a community network and links to other scientific communities such as the national research data infrastructure (NFDI) consortia. As a result, data are disseminated throughout the community, and data management techniques specifically designed to facilitate neuroscience research are developed. For example, our consortium is actively engaged in a number of international and national RDM initiatives, including the EBRAINS ((https://ebrains.eu/), National Node Consortium Germany), the NFDI bioimaging initiative in Germany NFDI4BIOIMAGE (https://nfdi4bioimage.de/), which promotes the development of high-level infrastructure and services across various scientific disciplines. Our involvement with different task areas (e.g., Neuromorphic computing (NMC), Data Analytics, Workflows, GDPR, The Virtual Brain Cloud, etc.) of these community-led initiatives supports the development of a sustainable and community-oriented RDM strategy. Our CRC puts efforts into following the recommendations of The International Neuroinformatics Coordinating Facility (INCF; https://www.incf.org) [116] and harmonizing our RDM efforts by using community-developed standards that have been accepted as international standards for neurophysiology and neuroimaging datasets (for example, BIDS, NWB etc., full list available at https://www.incf.org/resources/sbps). Resources such as FAIRsharing (https://fairsharing.org/ [117] and the UK Digital Curation Centre (https://www.dcc.ac.uk/) provide a comparative view of data and metadata standards. In addition to these domain-specific initiatives, we are engaging with Research Data Alliance (RDA, https://www.rd-alliance.org/) and European Open Science Cloud (EOSC,

https://eosc-portal.eu/ [118]) initiatives to adopt and develop new resources for open data exchange across technologies and scientific disciplines.

2.4 Comment: - p6, l23: What level of granularity was put into the guideline in terms of "data formatting"? As outlined in later sections, the data is highly heterogeneous.

The reviewer makes an interesting comment. We now added some information p. 28 lines 22-30:
"The acquisition of heterogeneous data in multiple projects can render the process of data formatting challenging, and the data policy guidelines do not specify a level of granularity for data formatting. However, the guidelines do provide general recommendations for how to format such diverse datasets, such as using standard data formats and tagging data with descriptive metadata. We supported individual research groups by providing resources and funds for the implementation of modern tools and infrastructure for compatibility with community RDM standards. We recommended using "standard" formats for data of similar modalities (e.g., neuroimaging data (MRI) is formatted to NIFTI, electrophysiology data is formatted to NWB, etc.) as described in the data standardization sections."

2.4 Comment: - p8, l2: How was the compliance to the survey? Given the complexity of RDM, did researchers need prior education wrt the meaning of RDM related questions of the survey, and were measures put in place to improve the quality of answers?

The reviewer raises interesting questions. Due to the collaborative nature of the consortium and the individual development of RDM strategies, the researchers were quite aware of the importance of data sharing and many aspects of RDM in general. Moreover, the head of the symposium supported and encouraged our initiatives, which facilitated the interaction with researchers and the compliance to the survey. Overall, researchers were familiar with RDM concepts and they welcomed help to optimize RDM procedure.
The goal of the survey was to conduct an assessment to better understand the researcher views', their current practices and needs, project-specific support needed from data manager, computing center and university library to establish proper data management practices and their willingness to share data with external collaborators if data manager offers some support in managing their research data. The survey questions are available in the supplementary file. The survey also included questions about data generation, various types and formats of research data generated, infrastructure for data storage and archive, practices of research data sharing, awareness on research data repositories etc. Also, during the second funding period, we focused more on providing support for data standardization and dissemination. Due to the pandemic, we managed to conduct online data seminars and also one on one RDM zoom meetings with each participating lab of the consortium, which helped us to optimize the survey results.

We now added the following text in the manuscript p. 13 lines 33-39, p. 14 lines 1-5:
"Direct involvement with researchers during the planning and initial implementation phases was crucial in order to identify the most helpful RDM measures, these may be as simple as coordinating communication between core IT staff and researchers, and facilitating access to institutional or other pre-existing resources. We devoted significant time to initially gather information about publicly available tools and services that would be useful to the diverse projects within the consortium. Given the large number of laboratories from various institutions participating in the consortium, as well as the increasing number of requirement changes over the course of a project, information was gathered in a variety of ways (virtual individual interviews with project PIs; discussions during online data seminars led by the CRC data manager; and personal meetings with experimentalists and PhD students). Data discussions and regular communication with consortium members have greatly aided our assessment approach. The PIs, or project responsible persons, were required to respond to a variety of data management questions as part of the assessment process (see Supplementary Information 2)."

2.5 Comment: - p8, Figure 2C: I found panel 2C not immediately clear in terms of the organization and identifying the row/column structure. A slight improvement visually and/or 1-2 sentences of description of the panel in the caption would help.

Following the reviewer's comment, we now edited Fig 2 (now Figure 1). See also comment 2.2.

2.6 Comment: - p10, l31-32: This is one concrete example of the general remark above: "manageability issues" are very abstract, an example of what types of problems precisely triggered this statement would be very illuminating and supportive of the statement.

We thank the reviewer for bringing up this important point. Some of these data manageability challenges include integration of multi-modal datasets; harmonization of heterogeneous datasets; keeping corresponding timing information; achieving and maintaining good data quality (checking for missing data, duplicates); properly securing all the collected data and adhering to privacy and security regulations while enabling access to specific users. We added some lines p. 4, lines 16-23:
"These diverse techniques and collected data types raise multiple data manageability issues within and between projects, with broad implications for data interoperability and reuse. Some of these manageability issues are inconsistent data formats, limited harmonization of heterogeneous datasets, time-consuming data acquisition, especially when dealing with large datasets, integration of multimodal datasets, inaccuracy in the representation of collected datasets, difficulties in achieving and maintaining good data quality (checking for missing data and duplicates), ensuring the security of all collected data, and adhering to privacy and security regulations while enabling access to specific users."

2.7 Comment: - p11, l2-: The same as the previous comment holds for the challenges associated with multi-modal data, as its unclear on which level the integration step happens. Moreover, I would welcome a more elaborate description of the level of integration of data in a tandem project: how far could researchers go in integration? Did researchers go as far as analyzing data from their tandem partner and vice versa? To which degree could datasets be actually integrated/compared in practice?

The reviewer makes an interesting comment. The level of integration of data in a tandem project depends on the scope of the project and the goals of the researchers. We now added a section on data integration p. 32, lines 11-40:
*"**Data integration***
For collaborative research, the data integration and standardization step becomes crucial for interoperability and data sharing [125], but is quite challenging to implement, given the wide range of methodologies represented in the consortium. Early data standardization can have massive benefits for data integration in collaborative projects, this can be achieved by streamlining the use of tools for more replicable and reproducible analysis. The data integration process often depends on individual projects and their underlying workflow and processes. The degree to which this is possible will depend on the modalities used, the subject population, and the experimental design. Researchers may also integrate the raw data collected from each partner into a core dataset. Integrated datasets can provide a more comprehensive understanding of the research question, as well as allowing the researchers to compare the results of their analyses more directly. Depending on the modalities used, the data may need to be transformed or normalized before integration, and the analysis techniques may need to be adapted to the combined dataset.
For some projects, researchers can go as far as analyzing data from their tandem partners and vice versa. Some human projects combine fMRI data from one group and EEG data from another group to gain a better understanding of how the two modalities interact. This could also involve combining the datasets, or running analyses on the combined dataset to identify common patterns or trends. However, this process requires careful consideration of the data sources, data formats, and analysis techniques used by individual labs, as well as the selected methods for data fusion and data mining. At the most basic level, researchers can compare the data collected from each partner to identify commonalities and differences in the data. This could include comparing the number and type of modalities used, the subject population, the experimental design, and the type of analysis performed. For example, they could investigate how brain structure (grey matter volume, cortical thickness) relates to behavior. There are also association studies aiming to compare brain activity between two groups of participants (e.g., healthy, chronic pain) in order to explore neural differences in cognition or behavior. They could also examine associations between neural activity in different brain areas and physiological responses of the subject. "

2.8 Comment: - p14, l15-: Did these reproducibility issues occur in practice?

These reproducibility issues did occur indeed in practice. We added some lines p. 7 lines 11-18:
"Problems with reproducibility [37] are made worse by the fact that the original analyses were done in different software environments, using different operating systems (e.g., Linux, Macintosh), and with different software or software versions. For example, variability in reproducing results from neuroimaging studies has been often investigated [38, 39]. Often, analyzing fMRI data with software packages such as SPM (Statistical Parametric Mapping) [40] or FMRIB Software Library (FSL) [41] might lead to different outcomes. Efforts are however being made to determine sources of variabilities and develop homogenous and standardized computing environments [42]."

2.9 Comment: - p15, l1-: One could also mention potential legal/data privacy concerns related to selecting an electronic lab notebook.

We thank the reviewer for the comment. We now added the following information p. 9 lines 22-30:
"When selecting an ELN, there are potential legal and data privacy concerns to consider [52]. An ELN is a digital resource which stores confidential information, and can be accessed by multiple users. To ensure legal and data privacy compliance, it is important to design ELNs compliant with all applicable laws, regulations and ethical standards. Additionally, it is important to consider the security measures in place for the ELN, including encryption and user authentication measures, to ensure that the stored data remains secure and private. Finally, it is important to consider the terms of service for the ELN, as this will outline how the data is used, stored and shared, as well as any limitations on the use of the data. By considering these potential legal and data privacy concerns, organizations can ensure that their data remain secure and compliant."

2.10 Comment: - p17, l24: Please reformulate or define "neural assemblies", as this may be ambiguous

We now defined *neural assemblies* p. 13 lines 3-40*:*
"neural assemblies (collection of neurons that are activated simultaneously in response to a particular stimulus and form assemblies)"

2.11 Comment: -p17, l15-: I really like this last subsection as it is very concrete and down-to-earth. However, for this paragraph in the section, I found it unclear where exactly the special challenge lies, i.e., what aspects are truly unique to this situation. Are similar problems not equally present, e.g., when looking at the neuropixel probes (p17, l1-) where different spike sorters will produce different results, and potentially similar computationally demanding workflows are required?

We thank the reviewer for the positive comment.
The section "Special RDM challenges" was added because it includes some DM strategies that were specific to some animal projects, whereas the previous section "Common data management challenges across projects" deals with common data collected across the majority of the projects. As the reviewer points out, challenges for neuropixels and two-photon calcium imaging are comparable in terms of size of data sets generated (around 100 GB/hour), which provides some challenges for data storage. Moreover, producing and analyzing such datasets requires high computational complexity and costs, necessitating an adequate computational infrastructure [34, 35].
We separated these two techniques because they differ in their spatial sampling and temporal resolutions, and are used to answer to different research questions.
To facilitate comprehension, we now changed the title of the subsection to "RDM challenges for specific projects" see p. 11 line 23. We also reformulated the sections on Electrophysiology and neuroimaging p. 12, lines 15-40 and p 13 lines 1-12:
"Electrophysiology with high-density probes
A few of the animal projects in the consortium make use of new technologies such as high-density Neuropixels probes [63]. Neuropixels datasets are often large (~80 GB/hour) and computationally demanding, which can make it difficult to scale spike sorting workflows across different labs and datasets. Data storage requirements rise as a result of the significant amounts of derived data needed for intermediate processing (such as filtering and spike sorting) and stimulation and/or behavioral parameters (such as optogenetic stimulation, motion or whisker tracking, and task performance). Analysis and post-processing may often require computationally intensive algorithms and hardware acceleration to handle data that

cannot be loaded into local memory [64]. Due to differences in recording conditions, spike sorting algorithms and data preprocessing, there can be significant variability in the results produced by different spike sorters. Real-time processing requirements for closed-loop experiments only serve to exacerbate these issues. Important parameters initially recorded from raw data, e.g., animal arousal/anesthesia level, impedance measurements, can be discarded in derived datasets used for analysis. Complex hierarchies of derived data and multimodal datasets (e.g., accelerometer, whisker or pupil tracking, etc.) collected with different instruments compound these issues. It can be challenging to validate and reproduce results, as the various algorithms and parameters used can produce different outcomes.

Large-scale in vivo two-photon calcium imaging
Some rodent projects within the consortium acquire large amounts of data collected over months [65]. For example, data acquisition using imaging techniques such as fluorescence imaging or two-photon microscopy calcium imaging (2P imaging) generates large sets of spatiotemporal imaging data (up to 100 GB/hour) and requires rigorous preprocessing steps (image segmentation, denoising, motion correction, manipulation and handling of large video files, and neural activity deconvolution) using high-throughput computing [66]. The downstream processing and analysis of the resulting datasets generated over the course of months is often challenging and requires complex workflows [67]. A few open-source software solutions (CalmAn [68], EZCalcium [69], etc.) have been proposed to deal with these challenges. However, comparative analysis studies have revealed that the neural assemblies (collection of neurons that are activated simultaneously in response to a particular stimulus and form assemblies) recovered from 2P imaging datasets can vary significantly depending on the algorithm used, and that certain algorithms are more reliable and faster than others [70]. Specific algorithms have been found to have higher precision and longer run times than others, while others have been found to have faster run times but lower accuracy. Another issue is that many studies include synthetic or benchmarking datasets, but producing and analyzing these datasets requires challenging calculations, raising the computational complexity and costs. This enables the need for more scalable and fully automated workflows that can be run on HPC clusters [65], which requires ensuring the reproducibility of studies [71]. Existing software solutions can be used for analysis and visualization of datasets, but any adopted data and metadata standards must be interoperable with these tools."

2.12 Comment: - p20, l1-: What distinguishes the three templates? Are DMPs considered living documents that accompany the project duration, and if so, how are they followed up?

To answer to the reviewer's questions, we now added the following information p. 17 lines 24-26, p. 18 lines 1-17:
*"**Data Management Plans for CRC projects***
We designed three DMP templates after defining and categorizing the data management needs for each project depending on its experimental model type: human, animal, and human-animal tandem (see supplementary information 3.1, 3.2, and 3.3, respectively). The templates can also be found on Zenodo: https://doi.org/10.5281/zenodo.4410128), [44]. DMP templates for animal, human, and tandem projects can differ depending on the scope of the project, the type of data collected, and how the data will be managed, but the major difference is the ethical considerations associated with each type. Animal projects, for instance, may necessitate additional safety protocols for the storage and management of animal tissue samples, whereas human projects require more stringent regulations and oversight for the ethical processing of human data [45]. Additionally, DMPs for human projects may include the collection of sensitive data (personally identifiable information), which must be securely stored and shared according to regulations. The DMP should follow General Data Protection Regulation (GDPR) -compliant guidelines for handling of personal data and specify information, authentication measures for reuse and recovery, de-identification of datasets involving human participants before sharing, and publication process for anonymized data. Human-animal tandem projects are more concerned with resource allocation and special protocols for the integration of different data types collected from multiple sources. It is important to ensure accuracy and consistency across all sources.
The DMP states minimum requirements for metadata that must be provided for long-term preservation and secondary analysis of research data. It also contains the information about data migration and access by third parties or future collaborators even after the project ends."

2.13 Comment: - p20, l21-: I found the listing of storage locations somewhat confusing. I would suggest to list first, the available options, underlying technology (e.g., nextcloud, ssh,...) and their properties (e.g., sharing possibilities, availability on HPC, backup, versioning, access,..) and, second, how these factors influenced the choice for labs in the consortium. Which features where actually decisive?

We thank the reviewer for the suggestion. We have now restructured sections on "Data storage" (p. 18 lines 19-40, p. 19 lines 1-37) and "Data processing, analysis, and visualization" p. 19 lines 39, p. 20, p. 21.

"*Data storage solutions*

Projects in the CRC generate massive volumes of data of various types and rely on data interoperability among labs. It is strongly advised that researchers securely store full datasets (e.g. raw, preprocessed, and analysis files, codes, etc.) associated with published findings and results, as this promotes the consortium's goal of further engaging in open science.

The CRC provides support for various storage solutions for all stages of the data life cycle, and the best option for a research project is determined by the type of data being collected, the size of the data, the security requirements, and the scalability of the platform. Factors such as interoperability with the existing infrastructure of the project group, reliability and accessibility for a particular storage solution, and availability of user-training and service support are also taken into consideration. For example, if the research project involves collecting large amounts of data, then a cloud-based storage platform may be the best option. Additionally, cloud-based storage platforms provide access to data from anywhere with an internet connection, making it easy for researchers to collaborate and share data with colleagues. If the data is sensitive, then a secure, on-premise storage solution may be the best choice because it can be customized to meet the specific security needs of the organization, such as encryption, authentication, and access control. Additionally, if the research project (e.g., projects collecting terabytes of data from methods such as optogenetics, electrophysiology, and calcium imaging etc.) requires scalability, then a platform that can easily scale up or down may be the best option. Ultimately, the best data storage platform for a research project will depend on the specific needs of the project, and university-approved data storage services (see Figure 2) are recommended to guarantee data privacy and confidentiality.

Most of the CRC researchers are encouraged to use our internal data storage platform SDS@hd (SDS, Scientific Data Storage, with a capacity of 20 PetaByte), a central service for securely storing scientific data https://www.urz.uni-heidelberg.de/en/service-catalogue/storage/sdshd-scientific-data-storage . To facilitate easy data sharing between internal collaborators, all datasets collected from rodent experiments in collaborative projects are shared across different research teams via SDS@hd, which stores experimental protocols, raw and pre-processed data, session, and mouse information (e.g., session start time, animal weight, etc.), task files (e.g., behavioral responses, videos, audio files), acquisition metadata (e.g., dimensions, pixel types, and instrumentation settings), in a shared directory structure for project members. It is intended to be used for data that are frequently accessed ('hot data'). A common storage place ("Speichervorhaben" (SV) meaning data storage projects) is requested for collaborative projects or work groups to ensure that data are easily accessible to all project members with proper authentications (university credentials). The Heidelberg University network connects the consortium's labs and university departments, using a 10 GB capacity network to expedite data transfer among institutes and facilities. Using SDS@hd drastically increases the ease of data access for shared projects and data safety by virtue of automated mirroring for data backup. Datasets collected from confocal or two-photon microscopes, for example, are stored on acquisition computers for a few days until they are stored on to SDS@hd. Once transferred to SDS@hd, individual users must ensure that the large datasets are timely (usually in few days or a week depending on data volume) removed from acquisition systems to free up the imaging acquisition system for new data or for another user.

Apart from its storage and fast data transfer capacity, another potential reason for storing large datasets on SDS@hd is its direct access to other university platforms such as HPC systems (explained in next section). Other university storage solutions that can be requested via support from CRC data manager include a SERVER BACKUP (https://www.urz.uni-heidelberg.de/en/service-catalogue/storage/server-backup, service for data storage for servers (based on a data protection and recovery software i.e. IBM Spectrum Protect (ISP) https://www.ibm.com/support/pages/overview-ibm-spectrum-protect-supported-operating-systems), CLIENT BACKUP service (https://www.urz.uni-heidelberg.de/en/service-catalogue/storage/client-backup (can be access via on all operating systems via Duplicati software) for secure storage of workstations and PCs, and HEIVOL-I (https://www.urz.uni-heidelberg.de/en/service-catalogue/storage/heivol-i), a service for creating network drives for university institutes and facilities. Similar services are available at the other participating CRC labs from other institutions.

For human data storage (including sensitive data), researchers use a storage server with restricted access (Dell Isilon server with a large storage and archival capacity). Designated personnel have the authority and responsibility to enable access to internal collaborators. When necessary, access can be given to external collaborators by assigning guest accounts with a data sharing agreement in place.

Data processing, analysis, and visualization

Several projects and laboratories in the consortium use laboratory-based analysis infrastructure, such as local computers, shared analysis workstations (laboratory computers with GPUs and pre-installed acquisition and analysis tools that are shared by several members), and computational servers that are run by individual laboratories or groups.

The CRC highlights the importance of keeping track of every step, from initial data recording to the analysis, and proper documentation of analysis code, pipelines, and scripts. As a constructive starting point, the CRC 1158 data manager has set up a dedicated code space on Github (https://github.com/CRC 1158RDM) where multiple repositories with analysis code and scripts can be hosted and shared for each CRC 1158 project. The CRC 1158's data management organization repositories are maintained by the data manager, and access is given only to the authorized project members.

In addition to local infrastructure and computing servers, there is university infrastructure available for more demanding data processing tasks, such as running computationally intensive analyses of heterogeneous and large-scale imaging datasets collected from humans and rodent projects. An application for access to these services can be made by individual laboratories with an initial application and usage support from the data manager. To seamlessly integrate the data analysis with the setup and execution of preclinical experiments, a platform such as the one proposed here was necessary. Some of the CRC projects are utilizing bwForCluster MLS&WISO (https://www.urz.uni-heidelberg.de/de/service-katalog/hochleistungsrechnen/bwforcluster-mlswiso) and a detailed tutorial on access and use is made available (see https://github.com/SFB1158RDM/HPCtutorial). This eliminates administrative and technical barriers to performing computationally intensive tasks such as large-scale modelling, simulation, and analysis projects, e.g., Neuropixels systems. The HPC allows job scheduling using SLURM (https://slurm.schedmd.com/documentation.html) and also sets up reproducible computing environments (e.g, Docker [83], Singularity [84]) to optionally run the modules on the HPC for particularly large data sets that are streamed directly to SDS@hd during acquisition, e.g., chronic recordings with dense electrode array or image segmentation for chronic imaging (miniscope) with the perspective of running standardized analysis workflows. Allowing users to access large datasets stored in SDS@hd without downloading them to their local computer aids in the seamless integration of data analysis procedures, saving overall computational time and costs. The data can be accessed from the bwHPC cluster using the same protocols as for local storage, such as NFS, SMB, and FTP. This service also allows users to access data stored in SDS@hd from multiple bwHPC nodes simultaneously, thus increasing overall speed of data access. By utilizing direct data access, scientists are able to take advantage of the increased computing power available in HPC systems and gain access to data stored in a scientific data storage system.

While writing this manuscript, bwForCluster Helix https://www.urz.uni-heidelberg.de/de/service-katalog/hochleistungsrechnen/bwforcluster-helix, a successor of the current HPC system, was made available to users. The Helix component will enable the use of seamless, cross-system workflows for processing and analysis of large amounts of data.

In our consortium's case, data management efforts had led to an increased user base for the HPC machines. Many factors played into a user's decision to use a particular HPC machine, such as its performance, cost, and availability. Data management efforts had made the HPC machines more attractive to users by providing individual support and training for access and use. The amount of training necessary to promote the use of HPC machines in a research lab depended on the particular needs and existing infrastructure. Generally, training and seminars covered topics such as high-level usage of programming languages, high-performance computing paradigms, and best practices for using HPC machines for neuroscience data processing and analysis. It also included guidance on how to design and optimize applications for HPC systems (e.g., refactoring of tools for direct access or use on HPC and bwVISU). A lab might also need to provide additional training for data management and analysis, or for using specific software packages.

Similarly, for processing massive (e.g., neuroimaging) datasets, some projects are utilizing the heiCLOUD (https://heicloud.uni-heidelberg.de/), an Infrastructure-as-a-Service (IaaS) cloud service that provides virtual machines that may be customized and utilized as needed for the project. A possible scenario for heiCLOUD usage within our consortium is to install complex and computationally expensive software packages and perform concurrent processing of massive neuroimaging datasets. This provides powerful

workstations for data analysis and can be especially useful for collaborative research projects that require data sharing between multiple research teams.

Another service that is frequently accessed by CRC members is heiBOX (https://www.urz.uni-heidelberg.de/de/service-katalog/collaboration-und-digitale-lehre/heibox), a secure Sync and share service hosted on heiCLOUD. This service is similar to commercial cloud storage services like Dropbox and Google Drive, and allows users to save, synchronize, share, publish, and jointly edit files. heiBOX is based on the Seafile software (https://www.seafile.com/en/home/), and allows users to search for text files, PDF files, and Office files in unencrypted libraries using a full-text search. Additionally, Office files can be edited by multiple people, and files and folders can be tagged and commented on. Markdown documents can be used to create private or public wikis. heiBOX also provides backup, synchronization, and storage of small research data and document files, and guest accounts can be requested for data exchange with external collaborators. The most common use of heiBOX in our consortium is to share documents such as CRC meetings notes, data seminar and workshops presentations, individual projects DMPs, manuscripts and figures.
Another application that some of the CRC projects are utilizing is bwVISU (https://www.bwvisu.de/), a remote service for scientists (universities in Baden-Württemberg state, Germany), as well as the corresponding software stack to deploy such a service on-premises. It has an interactive web front-end that supports large-scale data analysis and visualization without much human intervention. Our RDM services also include providing technical assistance with the refactoring of lab-customized preprocessing analysis pipelines (MATLAB and Python scripts) into more organized workflows/Graphical User Interface (GUI) that can be run on HPC applications.

2.14 Comment: - p21, l7: NFDI-Neuro is currently a non-funded consortium.

The reviewer is correct. We were part of two funding applications and we look forward to working collaboratively with other active members of the consortium in the near future.

2.15 Comment: - p21, l11: GIN is not linked at its first occurrence.

We now changed the link p. 34 line 10
"GIN (https://gin.g-node.org/)"

2.16 Comment: - p21, l15: How do the folder structure templates relate to standards such as BIDS?

Folder structure templates for research repositories provide a means of following BIDS standards, as well as other standards, while keeping data organized and accessible. They provide a common way of organizing data that can be used by all researchers, regardless of the tools they are using. These folder structure templates can be customized based on the type of experiment or data modality as well as the analysis processes that should be integrated with existing data organization systems.
We now added the following information p. 30 lines 17-24:
"Neuroimaging data stored in the BIDS convention, for example, can be saved in the section "03_data" for the raw data (e.g., nifti and JSON) and in the section "04_data_analysis" for derivatives (i.e., analyzed data). These templates, which are mostly workflow-based, are customizable to meet special requirements for individual experiments and analyses, ensuring that the structure may support rather than restrict research practices. These folder configurations can be utilized on desktop PCs, data versioning systems (e.g., GIN or DataLad), external hard drives, or any storage device to accommodate various data sets generated during experiments, independent of their format. "

2.17 Comment: - p21, l24: Similar as above, it would be of interest to better understand the technological foundation and usage scenario of HeiBox.

We have now described heiBox p. 21 lines 21-32:

"Another service that is frequently accessed by CRC members is heiBOX (https://www.urz.uni-heidelberg.de/de/service-katalog/collaboration-und-digitale-lehre/heibox), a secure Sync and share service hosted on heiCLOUD. This service is similar to commercial cloud storage services like Dropbox and Google Drive, and allows users to save, synchronize, share, publish, and jointly edit files. heiBOX is based on the Seafile software (https://www.seafile.com/en/home/), and allows users to search for text files, PDF files, and Office files in unencrypted libraries using a full-text search. Additionally, Office files can be edited by multiple people, and files and folders can be tagged and commented on. Markdown documents can be used to create private or public wikis. heiBOX also provides backup, synchronization, and storage of small research data and document files, and guest accounts can be requested for data exchange with external collaborators. The most common use of heiBOX in our consortium is to share documents such as CRC meetings notes, data seminar and workshops presentations, individual projects DMPs, manuscripts and figures. "

2.18 Comment: - p22, l7: I am not certain datalad is properly represented by this description. Does datalad itself offer storage? While I am not expert, I had seen datalad more as an abstraction layer to handle versioned datasets and workflow execution, largely focused on git-based versioning.

We took account the comment of the reviewer and edited some lines p. 37 lines 8-22:
"There are platforms such as DataLad (a US-German collaboration for computational neuroscience project (https://www.datalad.org/) and GIN (https://gin.g-node.org/) that may effectively compensate for a lack of local resources. These data hosting and sharing platforms can also ensure data versioning and encourage reproducible management of scientific data. Both DataLad and GIN are based on git and git-annex to provide a decentralized system for the exchange of large datasets. Datalad and GIN are interoperable, as datasets hosted on either of these platforms can be accessed via git-compatible systems. Datalad is an open source software package for the management of distributed datasets. It facilitates the sharing, retrieval, and organization of data in a distributed environment. Moreover, the GIN service can be deployed locally at all the participating labs and can be used as an in-house storage server and web user interface for DataLad datasets. Datalad does not offer storage, but it provides tools for the acquisition, organization, and management of data stored in remote repositories."

2.19 Comment: - p22: How were storage locations suggested to consortium members? What role did HPC play in this process?

We have now addressed the points raised by the reviewer p. 18 lines 25-40 and p. 20 lines 29-40:
"The CRC provides support for various storage solutions for all stages of the data life cycle, and the best option for a research project is determined by the type of data being collected, the size of the data, the security requirements, and the scalability of the platform. Factors such as interoperability with the existing infrastructure of the project group, reliability and accessibility for a particular storage solution, and availability of user-training and service support are also taken into consideration. For example, if the research project involves collecting large amounts of data, then a cloud-based storage platform may be the best option. Additionally, cloud-based storage platforms provide access to data from anywhere with an internet connection, making it easy for researchers to collaborate and share data with colleagues. If the data is sensitive, then a secure, on-premise storage solution may be the best choice because it can be customized to meet the specific security needs of the organization, such as encryption, authentication, and access control. Additionally, if the research project (e.g., projects collecting terabytes of data from methods such as optogenetics, electrophysiology, and calcium imaging etc.) requires scalability, then a platform that can easily scale up or down may be the best option. Ultimately, the best data storage platform for a research project will depend on the specific needs of the project, and university-approved data storage services (see Figure 2) are recommended to guarantee data privacy and confidentiality."

"Allowing users to access large datasets stored in SDS@hd without downloading them to their local computer aids in the seamless integration of data analysis procedures, saving overall computational time and costs. The data can be accessed from the bwHPC cluster using the same protocols as for local storage, such as NFS, SMB, and FTP. This service also allows users to access data stored in SDS@hd from multiple bwHPC nodes simultaneously, thus increasing overall speed of data access. By utilizing direct data access, scientists are able to take advantage of the increased computing power available in HPC systems and gain access to data stored in a scientific data storage system.

While writing this manuscript, bwForCluster Helix https://www.urz.uni-heidelberg.de/de/service-katalog/hochleistungsrechnen/bwforcluster-helix, a successor of the current HPC system, was made available to users. The Helix component will enable the use of seamless, cross-system workflows for processing and analysis of large amounts of data. "

2.20 Comment: - p23, l2-4: I think https://eln-finder.ulb.tu-darmstadt.de/home is a more recent and complementary resource to the Harvard spreadsheet, that should be also mentioned here.

We thank the reviewer for the information, which we added p. 34 line 29 .

2.21 Comment: - p23, l6-: How was the adoption of a common ELN received in labs? How difficult was the integration into lab workflows (especially for complex experiments) in terms of automation, such that ELNs were not perceived as additional burden?

The reviewer raises important questions, which we now provided some answers in the manuscript p. 34, line 30-40, p. 35, p. 36 lines 1-5:

"In our consortium, Individual researchers have benefited greatly from our computing center's local installation of the elabFTW, which includes secure cloud-based data storage. The service can also be used to serve as a central repository for documenting shared or collaborative experimental methods or protocols used by two or more CRC labs. Each project can have its own team, with members from participating labs having access to it. Transfer of these protocols from traditional notebooks or digital documents into ELN needed additional time and effort from project members. In order to ensure that the ELN is not seen as an additional burden, careful consideration had to be taken when automating the workflow. For example, the ELN must be designed to integrate seamlessly with existing protocols and should provide easy access to data at all stages of the experiment. Overall, most labs find that the benefits of ELNs outweigh any additional burden, as the system can save time and effort in the long run by automating data entry and providing easy access to protocols and data.

Within our RDM framework, ELN can be used as a platform for documenting minimal metadata that is generated automatically while performing an experiment. ELN are frequently not developed as full-fledged metadata systems and lack domain-specific features (such as support for reading or viewing neuroscience-specific file formats). Another issue is that most datasets lack consistent metadata schemas, even at the most basic level. In this particular instance, we use elabFTW, which accepts JSON files. elabFTW acts as a "notebook," tracking both primary data (experiment findings, measurements, etc.) and metadata (date, time, author, units, used inventory, etc.). The experimental metadata (e.g., microscope specifications, data acquisition settings) is stored in a standardized manner using a generic metadata file format that is compatible with open file formats, such as JSON or XML. However, from our experience, the integration of metadata from complex experimental protocols, and analysis procedures into an ELN has proven to be a challenge. Support for various data or metadata file formats generated during acquisition and analysis is not within the scope of elabFTW. Users frequently choose to standardize their datasets at any stage of their project lifecycle; by this, we mean that users standardize datasets either immediately after data acquisition (raw files), or during the pre-processing or final analysis stage before sharing or publishing. Adding any additional metadata information into the ELNs (e.g., elabFTW JSON file) without an automated tool or API can also be time-consuming. So, if a user needs to add, search, filter, or use various types of metadata on a regular basis, they will require a specialized tool.

To address these issues, we emphasized the importance of developing templates for the most common types of experiments performed in a single project (based on design protocols, biological methods, and so on). The development of a metadata GUI also enabled us to automate the process of metadata standardization in an experiment-specific manner.

We would also like to highlight some of our consortium's use cases. The experimental protocol for a project that collected a large amount of electrophysiological data was available on elabFTW, along with a JSON file containing basic experimental metadata. We made the decision to standardize raw data files such as .rhd and convert them to NWB format. On elabFTW, the basic experimental information/metadata was available, but not in a format compatible with NWB metadata. When we standardize raw data files, we frequently discover that the associated metadata files lack analysis parameters. The challenge now is to integrate all types of extensive metadata (experimental, acquisition, and analytical metadata) in a single file with a consistent format to ensure image data quality, reproducibility, and scientific outcome.

In this case, the metadata GUI's goal was to generate a JSON file based on custom JSON schemas substantial enough to generate a NWB file for a given experiment. These predefined JSON schemas are built around a specific experiment setup, such as intracellular electrophysiology data collected from the Intan RHD acquisition system. Once the JSON-schemas are created, all the labs conducting similar experiments could use these schemas to feed metadata collected from various acquisition systems and generate a JSON file. This also allows for customized validation of the data, which can ensure that the data is valid and accurate before being entered into the ELN. By specifying the data in a custom GUI, a more efficient and reliable workflow within the lab is enabled, as well as ensuring that all necessary information is included and kept in a consistent format.
Another scenario is where users can use the bioformat library to extract metadata from TIFF and other imaging file formats into JSON format, then add any additional fields using a custom tool and feed this file back into elabFTW if necessary. "

2.22 Comment: - p24, l2-4: I did not understand this sentence. Which structure and function in which populations?

We apologize for the lack of clarity. We now added the following information p. 22 lines 29-31:
"For example, we can directly compare the structure and function of various patient populations acquired in different projects, e.g., to examine commonalities between individuals with chronic back pain and fibromyalgia patients."

2.23 Comment: - p24, l15: I found the purpose of the volunteer platform somewhat unclear. Which problem concretely does it solve, i.e., which type of redundant data acquisition does it help to avoid?

We have now clarified the use of the data platform p. 31 lines 30-40, p. 32 lines 1-7:
"We are currently expanding our collaborative efforts by creating a data infrastructure platform that will establish a GDPR-compliant data registry (PainReg-registry, based on the Germany-wide ParaReg registry (www.parareg.de) [124] for human volunteers. To facilitate cross-project data merging, a core clinical data set will be defined. This entails assigning a unique identifier to each study participant that is shared by all projects, allowing researchers to determine whether the volunteer participated in multiple projects. This will allow organizations to share the same pool of volunteers for multiple studies, resulting in less redundant data acquisition. This can result in cost and time savings, as well as increased data collection accuracy. We, for example, experienced that a same study participant could be tested twice, and was assigned different IDs (belonging to different projects), resulting in redundant data acquisition and therefore unnecessary increased costs, particularly for genetic analysis.
Furthermore, the data registry will ensure that data privacy regulations are strictly followed by obtaining participants' consent to access data for secondary or follow-up studies. This will also include an identity management feature to limit access to authorized users. The registry will contain a wide range of data, including brain imaging, genetic, cognitive, and physiological data. This collaborative work will be coordinated by the consortium's future data infrastructure project, which will be tasked with implementing, testing, optimizing, and standardizing data analysis procedures and models that will be utilized in all projects."

2.24 Comment: - p24, l26-27: It was not clear to me how BIDS reduces manual metadata input.

Following the reviewer's comment, we have now added the following information lines p. 23 lines 5-15:
"In addition, using BIDS standards for the organization of MRI data enabled a consistent metadata structure, minimizing the number of manual inputs of metadata required and, hence, reducing the number of errors arising from misinterpretation of those inputs. For example, a typical MR brain acquisition includes numerous different protocols, such as anatomical and functional. In BIDS, metadata fields common across all subjects are specified in a single JavaScript Object Notation (JSON) file in the root directory instead of multiple files repeated for each subject. By organizing the data in this way, researchers can easily and quickly access the relevant information without having to manually input the metadata. Moreover, adoption of BIDS enabled the development of workflows for automated data extraction, curation and labeling [49].

For example, automatic extraction of a minimal set of BIDS compatible metadata can be performed using dcm2niix (https://www.nitrc.org/projects/dcm2nii/).”

2.25 Comment: - p25, l4: Typo: ses->license

We have now fixed the typo p. 23 line 27.

2.26 Comment: - p25, l28-32: Typo: Sentence seems broken in the listing of tools.

We have now fixed the typo p. 34 lines 7-12:
“This includes the Neuroscience Information Exchange (NIX) Format [139], Neurodata Without Borders (NWB; https://www.nwb.org) [93, 140] etc., data versioning tools (e.g., DataLad (https://www.datalad.org/) [141], and GIN (https://gin.g-node.org/), metadata collection tools (e.g., CEDAR, NIDM, Open metadata markup language (odML) [43], data representation models (Neo) Python library Electrophysiology Analysis Toolkit (Elephant), data analysis tools (e.g., Elephant, FieldTrip), PyNN [142, 143], etc”

2.27 Comment: - p26, l10-: This paragraph left me very confused. If ELNs are adopted (which have a primary function of enabling users to fill in metadata records via a configurable GUI) why then are metadata entered first in a custom GUI that then produces a JSON that is then fed into the ELN? Would it not be more straight forward to enter metadata in ELN-defined schemas, then export these to JSON (in case they are required outside the ELN)? Also, why is this procedure ephys-specific?

We apologize for this lack of clarity. We began our efforts with standardizing electrophysiology datasets into NWB, which is why the current GUI supports electrophysiology experiments. However, the GUI is extensible and can be extended to provide support for other data types simply by incorporating custom JSON schemas for other experimental types.
We have now added some liens p. 35 lines 33-40, p. 35 lines 1-2:
“In this case, the metadata GUI's goal was to generate a JSON file based on custom JSON schemas substantial enough to generate a NWB file for a given experiment. These predefined JSON schemas are built around a specific experiment setup, such as intracellular electrophysiology data collected from the Intan RHD acquisition system. Once the JSON-schemas are created, all the labs conducting similar experiments could use these schemas to feed metadata collected from various acquisition systems and generate a JSON file. This also allows for customized validation of the data, which can ensure that the data is valid and accurate before being entered into the ELN. By specifying the data in a custom GUI, a more efficient and reliable workflow within the lab is enabled, as well as ensuring that all necessary information is included and kept in a consistent format.”

2.28 Comment: - p26, l10-: Which types of metadata are we talking about -- generic or experiment specific?

We mean both type of metadata, i.e., generic and experimental. Here are some available examples: https://github.com/catalystneuro/heidelberg-metadata-gui/tree/main/heidelberg_metadata_gui/examples

2.29 Comment: - p27, l15: I would argue that while TIFF is a complex/potent file format, it's not really propitiatory. Also, the number of image file formats is not particularly large. Therefore, I would argue, the situation is somewhat different from neurophysiology.

We agree with the reviewer and made some changes to describe our efforts in targeting open formats for microscopic imaging datasets. Indeed we realized that the data harmonization process is quite different for neuroimaging and neurophysiology but it is indeed difficult to argue about the complexity in data standardization between these two modalities as there are several contributing factors. In our experience, it really depends on the diversity of acquisition systems and software used within an organization. Also, in many cases, neurophysiology often requires more complex data analysis than microscopic imaging, which can require additional file formats for storing and analyzing the data. Evolution of BIDS format and its extension to support other modalities such as PET, EEG, MEG and microscopic imaging data (Microscopy-BIDS) as well as for electrophysiology datasets are also contributing to our current standardization efforts.

For example BIDS-animal-ephys, https://neuroinformatics.incf.org/node/242) formats is not fully developed yet, but there is an ongoing effort from the INCF working group (https://incf.org/sig/incf-working-group-standardized-data) towards achieving interoperability between NWB, NIX and BIDS formats.
We added some lines p. 25 lines 8-13:
"Researchers collect and view microscopic imaging data from different vendor specific acquisition software in varying file formats (e.g., TIFF, multi-page TIFF, Nikon ND2, Leica LIF, Leica CZI or ZVI etc). It is often difficult to read metadata from these files in other softwares. While TIFF is the most commonly used file format because it is easily accessible by many current analysis software platforms, it has some limitations such as long latency and delayed data access while working with large batches of files [96]."

2.30 Comment: - p29, l15-: In this paragraph, it was unclear to me if the data management efforts actually led to an increased user base of the HPC machines -- or if essentially those groups used the HPC resources who know how to use them? In other words, how much training was necessary to promote use of HPC machines?

The reviewer raises interesting points. We have now added some information p. 21 lines 1-11:
"In our consortium's case, data management efforts had led to an increased user base for the HPC machines. Many factors played into a user's decision to use a particular HPC machine, such as its performance, cost, and availability. Data management efforts had made the HPC machines more attractive to users by providing individual support and training for access and use. The amount of training necessary to promote the use of HPC machines in a research lab depended on the particular lab's needs and existing infrastructure. Generally, training and seminars covered topics such as high-level usage of programming languages, high-performance computing paradigms, and best practices for using HPC machines for neuroscience data processing and analysis. It also included guidance on how to design and optimize applications for HPC systems (e.g., refactoring of tools for direct access or use on HPC and bwVISU). A lab might also need to provide additional training for data management and analysis, or for using specific software packages."
