# Supplementary material for: Data management strategy for a collaborative research center
Source: Gigascience. 2023 Jul 4;12:giad049. doi: 10.1093/gigascience/giad049 (PMC10318494; doi:10.1093/gigascience/giad049)
Supplement: giad049_Supplemental_Files [file giad049_supplemental_files.zip › Supp3.2_Human_Projects.pdf]

Plan Overview

A Data Management Plan created using DMPonline

Title: CRC1158 Data Management Plan Template: Human Projects

Creator:DEEPTI MITTAL

Affiliation: Other

Template: DCC Template

Project abstract:

Introduction

This data management plan (DMP) template has been designed for CRC1158 projects that generate, collect, and/or process research data from **human models**.

A CRC 1158 data policy is designed in order to follow the funding agency and host institution's regulations. The points described in the CRC data policy shall be followed by all members of the consortium and ensure that data on human subjects are transferred and used in a secure setting; that use of the data is compliant with ethical and legal requirements (including signed informed consent, ethics approval, and the applicable data protection laws, furthermore the EU data protection regulation, which is applicable from May 2018); and that the use of both existing as well as new data occurs in agreement with the Data Owner/Data Provider. Management of datasets that include personal information and health information of study participants will be compliant with the General Data Protection Regulation (GDPR, Regulation (EU) 2016/679).

The policy also mentions the creation of a data management plan for individual projects. This DMP template cover questions about how data is handled at each stage of the project, including a general project description, experimental and dataset descriptions, specific data documentation (types of data and experimental models, methods for acquisition/collection, questionnaires, analysis software), decisions on data and metadata standards and formats, proposed plans for the organization, access, sharing, short- and long-term storage and archiving.

The general questions addressing basic data management information such as data storage, project responsible, etc. can be answered at the initial stages of the project whereas domain-specific questions can be answered as the project progresses.

ID: 82101

Start date: 20-06-2019

End date: 20-06-2023

Last modified: 27-07-2022

Grant number / URL: https://gepris.dfg.de/gepris/projekt/255156212?context=projekt&task=showDetail&id=255156212&

CRC1158 Data Management Plan Template: Human Projects

Data Collection

What data will you collect or create?

Information about types of data to be collected or acquired during the course of the project.

- **What type of data modalities will be used to acquire data in this study?**

For example, MRI, fMRI, EEG, MEG, PET, Questionnaires, clinical assessments, etc.

- **What other types of data will be acquired in this study?**

For example, behavioral datasets, and biological samples.

- **Does the study incorporate any data acquired externally?**

For example, If there are any existing datasets (Data from external databases, external research centers or labs, medical hospitals or diagnostic labs, data acquired in multi-center studies, or from any kind of literature) used in this study, please list the data sources here. If there is a legal obligation in accessing or using this data, mention those obligations here. Also provide identifying parameters (DOI, URL, citation).

How will the data be collected or created?

Information about how the data type mentioned above will be collected, what will be the data formats/standards etc.

- **What standards or methodologies will you use? What type of data formats are acquired or used in this study?**

Please include datasets formats, raw or proprietary file formats, standards, file name extensions, etc. Highlight why a certain format is being collected or used. Format acquired directly from the scanner, acquisition software, or after pre-processing of any kind of data formatting, etc.

Proprietary file formats requiring specialized software or hardware to use are not recommended for preservation but may be necessary for certain data collection or analysis methods. Use open file formats where possible, or at least industry-standard formats such as Dicom, NIFTI, European data format (.edf), or the BrainVision data format (.eeg/.vhdr/.vmrk).

Ex: DICOM, nifti, etc.

- **What type of open data formats or standards are or can be used in this study (with the developing technologies)? Will the data be converted into other formats?**

Please include information about open data formats or file formats that can be utilized for the collected datasets. Also list software, and tools that are used for migration of these datasets into another standard format. This section can describe your future envisioned plan and would support in estimating costs involved for new developments.

For example, the Conversion of datasets into standard formats such as BIDS, metadata files into JSON, etc.

Information about naming and versioning of data files, folders, etc.

- **How will you structure and name your folders and files?**

Note: Researchers can use a folder structure template for organizing their data. https://zenodo.org/record/3987768#.YrGG6uxBxhE

Julien Colomb, Thorsten Arendt, Deepti Mittal, & Keisuke Sehara. (2020). Folder structure templates for research repositories (1.0). Zenodo. https://doi.org/10.5281/zenodo.3987768

- **How will you version control both datasets and other files such as code, analysis scripts, etc.?**

For example, Using GitHub/GitLab for Matlab or Python scripts. Different versions of the same datasets should be clearly specified.

Documentation and Metadata

What documentation and metadata will accompany the data?

Information related to data documentation, metadata standards, etc.

- **Will this study use any lab inventory management system or data documentation software?**

For example, mention any type of electronic lab notebooks, paper notebooks, digital systems, lab inventory management tools, or software utilized in this study.

- **What are the methods that will be utilized in this study?**

Documenting methods are encouraged for supporting the reproducibility of the studies involved in this project. In preparation for publishing, consider creating an online document or folder (e.g. open neuro, Github, zenodo, OSF) where your project methods can be gathered/prepared. If appropriate, provide a link to that space here.

- **What other file formats and standards will be used to help you and others better understand how your data are organized? What types of metadata are being stored alongside the acquisition data?**

Metadata files and their formats are listed here. It is important to highlight if there is any additional information required for the replicability of the datasets.
Ex: JSON, OME-XML, XML etc.

- **Is there any study identifier associated with the project?**

For example, Identifier for accessing datasets or a particular study from an internal/external database.

Ethics and Legal Compliance

How will you manage any ethical issues?

Information about data protection

- **Does this data contain any sensitive information?**

Ex: Patient age, date of birth, etc.

Note: If the project contains any datasets that contain sensitive information, there are additional steps of data management that need to be followed.

- **Does this project uses any software or tools for data anonymization or de-anonymization (before storage or sharing)?**

Give examples of the tools or software will you use to clean DICOM headers of personally identifiable information. E.g. PyDeface is a tool that can be used to strip facial structures from the brain.

Ex: Any type of Software, lab-written scripts (Matlab, python, etc.).

- **Please provide the name and a web link for the research ethics board (REB) that is responsible for reviewing and overseeing the legal and ethical compliance of this study. Give the file identifier of the application if possible.**

The Ethics and data protection guidelines by the international and national funding agencies and organizations such as DFG, European Commission, are responsible for setting standards and coordinating data regulations across Germany. Follow the discipline-specific standards and ethical regulations relevant to your project. The finding agency provides some regulations and best practices for ensuring the privacy of human participants.

- **If the project includes sensitive data, how will you ensure that it is securely managed and accessible only to approved members of the project? Who is responsible for data acquisition, and processing, and who is the data protection officer?**

Project members, data stewards, study nurses, data protection officers, and all the stakeholders who are involved in the project and have access to sensitive data or any kind of personal information should be listed here in this section. State how you will prepare, store, share and archive the data in a way that ensures participant information is protected, from disclosure, harmful use, or inappropriate linkages with other personal data.

How will you manage copyright and Intellectual Property Rights (IPR) issues?

Information about GDPR, IPR, etc.

- **Who owns the data?**

Please write the name of the data controller.

- **How will the data be licensed for reuse?**

This is important when it comes to data publication, archiving, sharing, and re-use. There are multiple types of licenses available for each type of dataset. Consider the appropriate means for sharing your data, including any funding, ethical, and/or confidentiality requirements.

ex: Licensing using Creative Commons (CC) Licenses 4.0 or CC0 (Public Domain) or CC BY (Attribution)

Storage and Backup

How will the data be stored and backed up during the research?

- **What are the anticipated storage requirements for your project (in gigabytes, terabytes, etc.)?**

For example, an estimated storage space needed during the project lifecycle. Please provide details about the data solutions. If your data is subject to strict rules governing human subjects and anonymity, an on-site solution installed on your institution's server may be required.

How will you manage access and security?

- **What anonymization measures are taken during data collection and storage?**

You may need to encrypt your data to ensure it is not accessible by those outside the project. For more information, see the DFG’s Guidelines for researchers on securing research participants' data

“Within the framework of privacy protection, the degree of anonymization of the data is an important consideration and thus is an aspect incorporated in privacy regulations. Different rules apply to data, which are dependent on whether the data is considered personal data, fully anonymized, or de-identified. Fully anonymized data has all personalized data removed, is given a separate identification code, and the key between the fully anonymized dataset and any path back to the original data is deleted such that it would be extremely difficult to trace the data back to an individual” (White et al., 2020). The technical steps for anonymizing neuroimaging data should be designed to achieve the level of privacy required by ethics protocols governing the study. See here for a selection of resources pertaining to anonymization.

- **What are the security measures and access protocols that are needed for data sharing with external collaborators?**

Please list all the details of data sharing platforms and security measures required for sharing the data. For example, for internal data sharing within the consortium labs, researchers might need access to a common data-storage platform such as SDS@hd.

In the case of external collaborators, more measures are required. Give details about data-sharing platforms that are being utilized, security protocols, etc.

Selection and Preservation

Which data are of long-term value and should be retained, shared, and/or preserved?

- **How will you store and retain your data after the active phase of data collection?**

All datasets collected in a project do not need long-term preservation. The decision to store or discard datasets will be based on their potential for reuse, their long-term significance, any contractual responsibilities to funders or partners, and the resources needed to keep them usable in the future. Deletion of datasets would require additional permission from project partners and should strictly follow funding agencies' rules and regulations.

What is the long-term preservation plan for the dataset?

- **Please describe the process for long-term data preservation, which platform or repository will be used for this**

purpose?

Tools such as DataCite's repository finder tool and re3data.org are useful for finding an appropriate repository for your data.

- **Please mention the types of data format that you plan to use for preservation?**

Data cleaning is an important step for preservation. Organize all the data files including data and metadata files, any software if used, scripts, etc., and document all the associated files properly. For example, use open, non-proprietary formats, wherever possible. If you convert data and file format from its original format, please mention the process used for the conversion and also document the new file format.
Note: Data should be anonymized or pseudo-anonymized depending upon the type of data.

Data Sharing

How will you share the data?

- **What is the process of data sharing in this project?**

Please highlight the process and purpose of data sharing with internal or any external collaborators.

- **What data will you be sharing and in what form (e.g. raw, processed, analyzed, final)?**

For example, In the case of animal neuroimaging datasets, a format such as BIDS is used.

- **Which service do you plan to use for data sharing?**

To choose an appropriate data repository, consult with data managers or librarians, or utilize the repository finder tools at DataCite and re3data.org. To improve findability, accessibility, and citability, select a repository that provides datasets with permanent identifiers (such as DOI).

Are any restrictions on data sharing required?

- **What are the restrictions for data sharing?**

Mention if there are any restrictions on sharing the data. Do these restrictions apply to internal collaborators or only external collaborators? Would you provide restricted access or would allow sharing on a contractual basis? The protection of the privacy of respondents and handling sensitive data, what you can share, and with whom, may depend on what type of consent is obtained from study participants. In a case where some (or all) or the data analyzed was previously acquired (by your research team or by others), what you can share for this current study may also be dependent on the terms under which the original data were provided, and any restrictions that were placed on that data originally. If available and necessary, a copy of your consent forms and licensing terms for any secondary data should be created.

- **Are there any restrictions on the reuse of third-party data?**

Please justify the reasons why it is not possible to share data with or without a license with third parties, such as data repositories or external collaborators.

- **Will data sharing be postponed/restricted e.g. to publish or seek patents?**

This can be answered with yes/no.

Responsibilities and Resources

Who will be responsible for data management?

- **Identify who will be responsible for managing this project's data during and after the project and the major data management tasks for which they will be responsible.**

Data manager, Any individual member of the project, or the Principle investigator.

- **Identify data stewardship roles and responsibilities of project members and other organizations during and after the project.**

Responsibilities can include Project management, Creation/update of the data management plan, data backup, Coordination with project partners / external parties, Metadata/documentation, etc.

- **How will responsibilities be split across partner sites in collaborative research projects?**
- **Will data ownership and responsibilities for RDM be part of any consortium agreement or contract agreed between partners?**

This is highly important for a collaborative research consortium. Please highlight how you plan to achieve this.

What resources will you require to deliver your plan?

- **What resources will you require to implement your data management plan? What do you estimate the overall cost for data management to be?**

This estimate should incorporate data management costs expected during the project as well as those required for the longer-term support for the data after the project is finished.

- Some repositories expect financial reimbursement for large data sets
- Some data centers charge money for the provision of data if you want to use them later (e.g. for sending a storage device with the data)
- There can also be costs for long-term archiving
- These costs can and should be requested from the research funders

- **Is additional specialist expertise (or training for existing staff) required?**

This can be answered by yes or no.

- **Do you require hardware or software which is additional or exceptional to existing institutional provision?**

This will help to identify the involved costs and training requirements.

- **Do you require any additional funds for data management?**

Carefully consider and justify any resources needed to deliver the plan. These may include storage costs, hardware, staff time, costs of preparing data for a deposit, and repository charges.
