# Supplementary material for: Data management strategy for a collaborative research center
Source: Gigascience. 2023 Jul 4;12:giad049. doi: 10.1093/gigascience/giad049 (PMC10318494; doi:10.1093/gigascience/giad049)
Supplement: giad049_Supplemental_Files [file giad049_supplemental_files.zip › Suppl1.pdf]

Supplementary Information 1 for manuscript "Data management strategy for the Collaborative Research Centre (CRC1158) - Heidelberg Pain Consortium"

Supplementary Information 1

Informed RDM Policy and Decision Making for CRC 1158

This document outlines the policy of the Collaborative Research Center 1158 (CRC-1158) with regards to Research data management (RDM) and data sharing of all scientific projects. The RDM policy reflects the commitment of the CRC-1158's researchers. The consortium members adhere to the German Research Foundation's (DFG) research data policy as well as the host universities' research data policies. There are regulations, guidelines, and practices in place that will ensure observance with good scientific practice when guaranteeing researchers with ideal information on general RDM -related topics, for instance, data sharing and archival.

In particular, to ensure the reproducibility of the research done, our policy encourages participating project members to document the complete workflow that lead to the published data. This document serves to specify this requirement for our purposes considering the feasibility for the different applications. A minimal set of metadata associated with data for each project that shall be stored is defined below. In general, "data" refers here to any result from research that cannot easily be reproduced, that includes software, notes on analytical calculations, scripts as well as actual raw and condensed data displayed in plots in publications. The PIs are responsible for ensuring the implementation of the data policy.

In addition, The Z project provides help with supporting infrastructure and resources for documenting the data (electronic lab notebook licenses), support for software development and educational and training support by conducting regular data seminars. Awareness and sensibility to good scientific practice and proper data management are a responsibility of all members of the CRC and will be ensured by regular communication through the data manager.

In particular, the RDM policy includes:

1. The university's research data competency center helps PIs to the best of its capacity. The center provides guidance and assistance in the creation of data management principles. This requires early contact, ideally, while applying for the grants or at the start of the project.
2. While project leaders and independent researchers are largely responsible for RDM (long-term archiving) of their projects, research support services provide researchers with access to relevant databases. All consortium members working collaboratively on a particular research project are accountable for the validity and reliability of the data acquired during the project and for adhering to the regulations of their respective host institutions involved.
3. Open access to research data is encouraged by the CRC1158. In compliance with Heidelberg University's Research Data Policy, CRC1158 advocates making both research data and scientific papers publicly accessible as soon as possible. Every research project requires a data management plan (DMP) to ensure the accuracy, provenance, and completeness of the given data.
4. The RDM policy ensures access and availability of data in compliance with open-access, ethics and privacy regulations by implementing appropriate security measures. The members of the CRC1158 observe ethical, data protection, and copyright or confidentiality issues in RDM. The personal data of any subjects, patients, and other persons involved in data collection are protected in accordance with the data

protection guidelines. When subsequent use or publication rights are transferred, special care is taken to guarantee that the data remains freely available for research purposes. However, researchers are not required to make research data accessible to others outside the members of the team prior to processing, analysis, and dissemination. Subject-specific characteristics are taken into consideration.

5. The CRC1158 is adapted to the basic research data infrastructure of the Heidelberg University and thus ensures appropriate storage and technical availability of the digital research data. The digital research data is stored and archived in the IT infrastructure of the university computer center (URZ, https://www.urz.uni-heidelberg.de/de) or in recognized external or internal specialist repositories. External partners can use the IT infrastructure of the URZ or guarantee to use a similar infrastructure to ensure adequate storage of digital research data. Data held outside the university as part of the DMP should be registered with the Research Data Competence Center. The Competence Center for Research Data (KFD, https://data.uni-heidelberg.de/index.en.html) offers data registration that provides evidence of data sets from both university and external repositories. All rights to data, in particular the right to further use or publish the data, should be reserved for the PIs and should not be assigned to third parties.
6. Research data can be published in the institutional data repository i.e. heiDATA (https://heidata.uni-heidelberg.de/). The long-term archiving of research data is important for the quality assurance of any scientific work, but also a basic requirement to enable the reuse of research results. Each project leader can have easy access to this platform and is supported by the library staff in publishing their research data. The research data should be archived and/or published in a suitable, trustworthy data archive or repository in the long term (minimum 10 years).
7. The documentation of research data should follow subject-specific standards. The PIs of the CRC1158 undertake to comply with discipline-specific data and metadata standards. They are part of the scientific output of the researchers of the CRC1158. The consortium appointed a data manager who advises the scientists on entering data and metadata into the repository. For this particular purpose, the data manager participates in community organizations and works in coordination with the working group of the NFDI-Neuroscience and NFDI4BIOIMAGE.
