# Supplementary material for: Data management strategy for a collaborative research center
Source: Gigascience. 2023 Jul 4;12:giad049. doi: 10.1093/gigascience/giad049 (PMC10318494; doi:10.1093/gigascience/giad049)
Supplement: giad049_Supplemental_Files [file giad049_supplemental_files.zip › Suppl2.pdf]

Supplementary Information 2: Data Management assessment questions

CRC Project number and Title:

Name and Email ID of Project responsible:

Objective of this assessment

This template is created for CRC1158 projects.The main objective of this questionnaire is to gather information about the current situation of data used in various CRC1158 projects, namely to identify the current methods of data storage, data sharing, and security, etc. The information contributed by each lab will be used to evaluate the current status of RDM across all the labs involved in the SFB project and develop new data standards. The survey was designed from a researcher's perspective rather than the data manager's point of view. The survey questionnaires consists of some general questions about the current practices concerning each step of the data management lifecycle (e.g. data volumes, data storage) and domain-specific questions (e.g. data types, data standards) that could help in developing specific solutions.

1. **What term(s) best describe(s) your field of research?** Please mention the neuroscience sub-domain of the project.

2. **What is your experimental model?** (human, mouse, etc.).

3. **Does your lab or research group have a designated contact person for data management?** This can be a person outside of the SFB project who deals with all kinds of data produced from the experiments or data shared with other collaborators. This can also be a person who is working more on developing software or doing more technical tasks.

4. **Please mention the amount (for assessing storage needs) of data that is being generated from SFB projects from your lab.** Please consider all stages of the data lifecycle: data collection, data acquisition, data storage, preprocessing, analysis, repeated analysis, collaborative data exchange and analysis, accessing data and results from completed projects, data used in publications and journals, etc.

5. **Briefly describe the type(s) of data that is being handled in your lab.** For example, electrophysiology, neuroimaging data, sequencing data, gene expression data, etc.

6. **What specific tools, standards, or services (eg., storage platforms, data sharing solutions etc.) are being used in your lab to support the data management tasks in your area?** For example, if the project utlizes host university's infrastructure, please mention the name of those services.

7. Please mention up to 3-5 major data management challenges in your lab. These issues can be related to how data are stored, analysed, or shared with external collaborators or within the consortium.

8. Do you recommend any other data standards, tools or methods in your specific domain? This question will help us to implement or provide access to those services.

9. According to you, which specific topics should be addressed in CRC data seminars? This question will help us to organise regular seminars that will cover both general RDM solutions and also domain-specific tools.

10. Do you have any specific questions or remarks?
