# Supplementary material for: Data management strategy for a collaborative research center
Source: Gigascience. 2023 Jul 4;12:giad049. doi: 10.1093/gigascience/giad049 (PMC10318494; doi:10.1093/gigascience/giad049)
Supplement: giad049_Response_to_Reviewer_Comments_Original_Submission [file giad049_response_to_reviewer_comments_original_submission.pdf]

Author's Response To Reviewer Comments

Close

Reviewer reports:

1. Reviewer #1:
The authors describe a multistage plan that they implemented to improve data standards and management for the Heidelberg Pain Consortium. Having done similar work, I can tell that a great deal of thought and work went into creating this management program, however the paper will need substantial refactoring for that effort to shine through. The pieces of a great paper are already here, they are just difficult to follow.

Major comments:

1.1 Comment: pg 3, lines 31-33 "Implementing effective data management strategies and ethical rules for the reuse and sharing of high-quality data reduces redundant research, optimizes public research funding and reduces animal use."
This seems like a significant claim supporting the need for RDMs, but has no citation. Is there evidence that RDMs reduce animal use or reduce redundant research? What does optimized public research funding look like? This statement is surrounded by several other assertions with little explanation, e.g.: "A clear set of regulations and guidelines must be established before sharing human data gathered from clinical or non-clinical populations. Specific rules addressing privacy issues, established processes for data protection, data use and reuse, and the preservation of sensitive data are required. It is essential to make data accessible and understandable to remote (or future) collaborators in order to maximize the potential of existing algorithms and tools and accelerate the creation of new ones."

We thank the reviewer for the comments. We now reformulated our sentence and added some references as follows, see p. 27 lines 26-40:
"Implementing effective data management strategies and ethical rules for the reuse and sharing of high-quality data may reduce redundant research [5, 6]. One of DM goals is to make study results freely available through open-access publishing. By investing in collaborative projects with long-term goals, it ensures that the data are organized in a way that makes them easily accessible and retrievable for future use. This makes it easier and faster to develop new research projects, as well as to replicate or build on existing studies, which should have a direct impact on public research funding (https://sor.senate.ca.gov/sites/sor.senate.ca.gov/files/0842%20policy%20matters%20Research%2003.18%20Final.pdf). Moreover, effective DM strategies can also enable to optimize public research funding by pooling resources and infrastructure from multiple sources and bringing together experts from universities, research institutes, and other community organizations to work on long-term interdisciplinary projects. Furthermore, by ensuring proper RDM, researchers should be able to reduce animal use. For example, making informed decisions about which animal models to use for their studies should enable to use the same animals for multiple experiments, instead of having to continuously use new animals for each study. In addition, sharing previously acquired data with adequate metadata, or reuse of control group data from similar studies can avoid repeating in vivo work [1-4]."

1.2 Comment: I would like to see the introduction more thoroughly discuss the problems with data management and elaborate on these asserted solutions.

We agree with the reviewer's comment. We now added a section on data management challenges in the introduction section, starting with the common data management challenges across projects (including challenges due to the diversity in data types, challenges due to diversity in acquisition, preprocessing, and analysis approaches, metadata challenges, data storage and volume, challenges in data documentation, data sharing and dissemination challenges and challenges due to sensitive data). Then we present some RDM challenges for specific projects (including behavioral experiments, electrophysiology with high-density probes, large-scale in vivo two-photon calcium imaging, human-animal tandem projects), see p. 4 lines 6-39, p. 5-7, p. 8 lines 1-13.

1.3 Comment: There are multiple other places throughout the text where there are ideas stated as facts without references or elaboration, that I would like to see evidence for, or at least a discussion of.
For e.g. pg 13, lines 1-2 “There is, furthermore, a lack of efficient software to adequately segregate and maintain control over sensitive data. It is important to provide researchers working with sensitive data or samples with truly "useful" tools that do not require pre-existing, in-depth knowledge of legal and ethical requirements, or time to delve into the details.”

Following the reviewer’s comment, we now added some information and references in the discussion section entitled “Sharing sensitive data from human projects”, p. 30 lines 26-40, p. 31, p. 32 lines 1-7.
“Sharing sensitive data from human projects
The sharing of human data gathered from clinical or non-clinical populations in neuroscience research is essential for advancing science and producing important public health benefits. A clear set of regulations and guidelines must be established before sharing human data gathered from clinical or non-clinical populations. Specific rules addressing privacy issues, established processes for data protection, data use and reuse, and the preservation of sensitive data are required. It is essential to make data accessible and understandable to remote (or future) collaborators in order to maximize the potential of existing algorithms and tools and accelerate the creation of new ones. Regulations and guidelines should ensure that the data are used for the purpose for which they were gathered and protect the rights of participants in the research. These guidelines should also cover how data should be collected, stored, shared, and destroyed. They also specify the types of data that must be kept confidential and the appropriate methods for handling and safeguarding the data. They should also ensure that the data are secure, kept confidential, and not used for marketing or other commercial purposes. Additionally, regulations should ensure that the data are used responsibly and that they are not used to discriminate against people with disabilities or other vulnerable populations. Ethical rules for the reuse and sharing of data should be based on the principle of informed consent. This includes obtaining consent from the original data collectors or from research participants, as well as obtaining permission from any third parties involved in the data collection. Researchers should also seek to minimize the risk of data misuse or breach of confidentiality, and any data that are shared should be done so in a secure manner.
Additionally, there is a lack of efficient software programs to adequately segregate and maintain control over sensitive data. It can be difficult to develop effective software that is secure, user-friendly, and cost-effective. The maintenance of such software requires a significant investment of resources, and often there is a lack of funding available for such measures. Finally, the adoption of such software requires investment in training and resources, which many organizations may be unwilling to do. The legal and ethical requirements surrounding the use of sensitive data are often complex and difficult to understand, leading to confusion and ambiguity about the best way to protect them. It is important to provide researchers working with sensitive data or samples with truly "useful" tools that do not require pre-existing, in-depth knowledge of legal and ethical requirements, or time to delve into the details. Such tools are important to ensure that sensitive data are protected and securely stored. Use of such tools can help researchers to make informed decisions about how to best use and manage sensitive data, allowing them to work with it in an ethical and responsible manner. Finally, these tools can help to reduce the risk of data breaches and data misuse, which can have serious consequences for the people and organizations whose data are affected. By providing such tools, researchers can focus on their research and not on legal and ethical considerations, thus saving their time and resources.
There are several software tools that can be used to maintain sensitive patient data in neuroscience research. Some examples include a web-based platform REDCap (Research Electronic Data Capture) [121], an open-source imaging informatics platform XNAT (XNAT Central) [122], LORIS: Longitudinal Online Research and Imaging System [123] etc. It is important to note that the security features of these tools may vary and should be evaluated before use. In addition to software tools, secure data storage and access protocols should also be in place to ensure that sensitive patient data are protected.
We are currently expanding our collaborative efforts by creating a data infrastructure platform that will establish a GDPR-compliant data registry (PainReg-registry, based on the Germany-wide ParaReg registry (www.parareg.de) [124] for human volunteers. To facilitate cross-project data merging, a core clinical data set will be defined. This entails assigning a unique identifier to each study participant that is shared by all projects, allowing researchers to determine whether the volunteer participated in multiple projects. This will allow organizations to share a same pool of volunteers for multiple studies, resulting in less redundant data acquisition. This can result in cost and time savings, as well as increased data collection accuracy. We, for example, experienced that a same study participant could be tested twice, and was assigned different IDs (belonging to different projects), resulting in redundant data acquisition and therefore unnecessary increased costs, particularly for genetic analysis.
Furthermore, the data registry will ensure that data privacy regulations are strictly followed by obtaining participants' consent to access data for secondary or follow-up studies. This will also include an identity management feature to limit access to authorized users. The registry will contain a wide range of data, including brain imaging, genetic, cognitive, and

physiological data. This collaborative work will be coordinated by the consortium's future data infrastructure project, which will be tasked with implementing, testing, optimizing, and standardizing data analysis procedures and models that will be utilized in all projects.

1.4 and pg 13, lines 26 - 29; Why do available commercial cloud storage solutions come with unreliable and slow backup and restoration services, as well as no obvious access paths or interfaces for easy migration onto the analysis platforms?

Following the reviewer's comment we now added the following information p. 8 lines 27-38:
"Many commercial cloud storage solutions are designed primarily for convenience and cost savings rather than for robust backup and restoration services. As a result, these services often lack the features necessary for reliable and fast backup and restoration, such as automated data backups, point-in-time recovery, and incremental backups. Additionally, many cloud storage solutions lack the necessary APIs, scripts, and tools that would allow for easy migration of data onto the analysis platforms. This makes data migration difficult and inefficient, and it can significantly slow down the analysis process.
Assuring access to secure and optimal storage solutions that can be integrated with workflows encompassing data acquisition, intermediate analysis, and archiving is thus a major challenge. Creating backups and storing multiple copies of large volumes of datasets, the need for collaborative and parallel access by multiple people, and use across a diverse range of computational workflows all pose major challenges to storage servers."

1.5 pg 15, lines 13 - 19;
We now added a reference p. 9 line 19:
Higgins SG, Nogiwa-Valdez AA, Stevens MM. Considerations for implementing electronic laboratory notebooks in an academic research environment. Nat Protoc. 2022 Feb;17(2):179-189. doi: 10.1038/s41596-021-00645-8. Epub 2022 Jan 14. PMID: 35031789.

1.6 pg 16, lines 4-5; Another significant issue with submitting data to general repositories is that they do not have enough support for certain types and formats of data.

Following the reviewer's comment, we now added some information and some references p. 10 lines 13-28:
Another significant issue with submitting data to general repositories is that the latter might not have adequate support for certain types and formats of data [54, 55]. For example, if the data are in a non-standard format, the repository may not be able to process it correctly or even accept it. Additionally, repositories may not have specialized tools or services to help researchers convert, organize or analyze the data. This can be especially problematic for data that are highly specific, such as medical records or geospatial data. Without the necessary support, researchers may be unable to make full use of the data or even access them. Additionally, general repositories may not have the same level of curation and organization as a specialized repository, which can make it harder for researchers to evaluate the quality and relevance of the data. This can lead to a lack of reproducibility and increased difficulty in building on previous research. Therefore, it is recommended for researchers to submit their data to specialized repositories that are tailored to their specific field. Neuroscience specific repositories, on the other hand, are specifically designed to accommodate the unique needs of neuroscience data (openNeuro). They are often managed by experts in the field and have the necessary infrastructure to ensure the safe and secure storage of data. Furthermore, they often provide additional services such as data analysis, curation and visualization tools, which allow researchers to better understand and use the data.

1.7 pg 4,lines 2-5 "While the majority of collaborative research consortiums collect a wide variety of multidimensional datasets, the majority of these datasets are typically inadequate for modern research methods and infrastructure." As above, I'm interested in how this is quantified, or if there is a reference, and to have a more complete discussion of this idea.

Following the reviewer's suggestion, we now added some information and some references p. 6 lines 1-22:
"The availability of robust neuroscience resources such as high-performance computing (HPC) clusters [23-25], modern workflow technologies (e.g., Galaxy [26], Snakemake [27] etc.), cloud-enabled storage and computing infrastructures (e.g., Amazon AWS, Google Cloud [28]), secure databases [29], repositories [30], and analysis platforms are fundamentally changing how research in neuroscience is communicated and linked to existing raw data and findings [31]. Such tools are allowing researchers to utilize diverse techniques and produce massive amounts of high-dimensional data (large sample size, various models and conditions), which provides greater statistical power and the opportunity to do

more robust secondary data analysis [32]. However, the data-driven neuroscience approach, as a whole, is questioned by a number of technical issues that must be addressed before it can be fully realized.
While the majority of collaborative research consortiums collect diverse multidimensional datasets, one of the primary challenges is that the majority of these datasets are typically inadequate for modern research methods and infrastructure [33] . Before committing to any of these tools for processing and analysis of collected datasets, it is important to understand data in terms of the number, volume, size and complexity of data, the types and formats of data, and the accuracy and completeness of the datasets. Despite similarities in experimental design, researchers in neuroscience experiments frequently organize and describe their data in their own way, even within the same research group. As a result, datasets collected from different research groups for a single project addressing the same scientific question may not be in formats suitable for comparison and pooling, limiting their interoperability and reuse. The data formats collected in each project are typically determined by the acquisition and measurement method, intermediate pre-processing, or analysis software. This can lead to data and metadata being stored in different locations."

1.8 Comment: Beginning on line 27 of page 4, the introduction veers into describing many technical details of specific studies. In my first read-through, I actually thought that I had accidentally flipped to a different paper. None of these details seem relevant to the larger concept of creating an effective RDM strategy, and are very disorienting. I would remove them entirely.

We agree with the reviewer's comment and have reorganized the introduction section, which is now more focused on presenting RDM challenges (see also comment 1.2).

1.9 Comment: Figure 1. In order to understand how the authors have implemented an RDM plan, it seems important for the reader to understand the basic structure of the consortium, however, I do not understand how to interpret figure 1. It appears that the animal projects somehow encapsulate all the others? Are the relative sizes of the colored portions meaningful? The number of tandem projects is half that of animals, which seems to agree with the peach width being smaller than the green. However there are only 4 human projects for the very large yellow section, and 1 for the black. I would drop this figure entirely. I think Figure 2 does a much better job of describing the consortia, and is much easier to read. Further, pg 8, lines 5 - 12 are a near copy of the legend for Figure 1, but refer to Figure 2 A anyway.

Following the reviewer's comment we now removed figure 1 and edited the "old" figure 2 (now figure 1) and adapted it to the current structure of the consortium.

Figure 1: The Heidelberg Pain Consortium investigates various populations: humans, rodents, and tandem using various modalities: neuroimaging, neurophysiology, behavior, psychometrics, and genetics (A). Each data modality can be recorded using different techniques: MRI= magnetic resonance imaging; PET/MR: positron emission tomography/magnetic resonance; fNIRS: functional near infrared spectroscopy; NIBS: non-invasive brain stimulations; EEG: electroencephalography; MEG: magnetoencephalography; Genetics; Two-photon imaging; Behavior; EMA: ecological momentary assessment; psychometrics; physiology; VR: virtual reality; optogenetics (B).

1.10 Comment: Beginning on pg 6, line 35 to pg 7 line 16; and then again beginning on pg 10, line 34 to pg 18, line 16, this entire text is general background of problems in data management, and specifically around the particular challenges of this consortia. It is altogether a much better introduction than the existing introduction. It covers all of the missing context, as well as discussing the types of studies the Heidelberg Pain Consortium needs to work with without delving into over technical details. I recommend excising this from the results and using it to replace everything after the second paragraph of the introduction. With minor adjustments for grammar, it will be much better there. The move will also make your actual results, which currently don't begin until pg 18, line 18, much easier to find and follow.

We thank the reviewer for the suggestion and we now reorganized the introduction which contains the challenges section (see also comment 1.2).

1.11 Comment: In the results (beginning pg 18, line 18), there are several sections where important discussion points are

interspersed with the results, making it difficult to tell which are the work of the consortium, and which are simply informational. It would greatly improve readability if these paragraphs were pulled out and integrated into a discussion section, which doesn't currently exist. As I'm finding it difficult to tell which parts are your work, below is my best guess at the paragraphs that should be moved, or dropped:
pg 19, lines 27 - 32
pg 20, lines 1 - 12
pg 21 line 1 - pg 22 line 18
pg 22 lines 19 - 29 (excluding the title, which should be moved to line 30), pg 22 line 34 - pg 23 line 5
pg 23 lines 18 - 28
pg 26 line 10 - pg 27 line 6
pg 27 line 31 - pg 28 line 1 (excluding the title, which should be moved to line 2)
pg 30 line 33 - pg 32 line 21
For the "Recommended data standards for different data types", "Animal neuroimaging data standardization", and "Data processing, analysis, and visualization" sections, the results and discussion pieces are not as easily pulled out as paragraphs, so I have not listed each individual sentence here. Instead, I would suggest looking at these sections with an eye towards picking out which are results of something your consortium worked on, and which are expounding on ideas or comparing it to other work.
Clearly moving all of those to the discussion will require some editing to make it make sense, but I think that if you make the discussion follow the same minor heading structure you have in the results it will be a relatively painless change. The first part of the current results: pg 5, line 23 - pg 6 line 34 would be very good if moved to be the first few paragraphs of the discussion, but are too vague to be part of the results.

We thank the reviewer for the comments. We have now restructured the RDM Implementation (section II in the manuscript) to highlight the strategies used by our consortium. We also added a discussion section.

1.12 Comment: pg 6, line 37 - pg 7 line 5. "We specifically describe our ongoing RDM efforts, which are divided into two sections: 1) RDM Planning Phase: Identifying common RDM procedures across consortium projects; Evaluating common data management challenges; Special RDM challenges in specific projects; 2) RDM Implementation Phase: resource allocation decisions and implementation of key resources and 3) Data Dissemination (data management continues even after the end of the funding period)." A minor typo is that it should say three sections, not two. Rather more importantly, both here and in the conclusion, it lists three phases, but phase three does not appear in the actual text. It is not described along with the others in the conclusion, or mentioned at all in the results.

We now revised the manuscript and divided the main text into two main sections: section 1: RDM planning phase and section II: RDM implementation, followed by a discussion.

Minor comments:

1.13 Comment: SBF is never defined, and the authors seem to use SFB1158, SFB and SFB 1158 as synonyms, but I'm not sure if that is the intent.

Thank you for pointing this out. We have now homogenized the terms and use CRC in the entire manuscript. We also added the following information in the funding section p. 40, lines 35-37:

“Collaborative Research Centers (CRC; short SFB for German ‘Sonderforschungsbereich’) are university research projects which are funded by the German Research Foundation (DFG), generally for a period of up to 12 years.
https://www.dfg.de/foerderung/programme/koordinierte_programme/sfb/”

1.14 Comment: I would move all of the funding information on pg 4 to the funding section, it's distracting here
We now moved the funding section to the end of the manuscript p. 40, lines 35-40.

1.15 Comment: There are several minor grammar issues. I am not copying them all here, as I'm sure they'll be fixed in the rearrangement, but:

pg 3 line 26 should be 'cooperative'
We corrected now all typos and the entire manuscript was meticulously proofread.

1.16 Comment: pg 6, line 7; its unclear what "progressively practical RDM approaches" means
We apologize for the lack of clarity. We aimed to emphasize how practical our RDM efforts are in terms of allocating resources and creating new services while taking pre-existing services into account.
We now clarified the RDM approach p. 30 lines 1-8:
“We also focused on developing RDM strategies that included joint efforts and cooperation between consortium members and other large-scale consortiums and collaborative centers within Germany. We acknowledge the common issue of data organization for different projects in collaborative centers. Joint efforts were made for the development of a data organization strategy that works for most of the projects within the consortium working in similar research areas. Our main goal was to engage more directly in several overlooked aspects of managing data in a large collaborative consortium while keeping the global neuroscience community in mind. We encouraged CRC 1158 projects to utilize logical file and folder templates to support systematic data organization.”

1.16 Comment: pg 7, line 1 should be 'three'

The reviewer is correct. In the current version we however decided to have two phases and one discussion (see also comment 1.12)

1.18 Comment: pg 7, line 21 'can' should be 'could'

We have now removed the previous sentence.

1.19 Comment: pg 11, lines 23-26, this sentence doesn't seem to finish "In cases where a laboratory produces a large dataset from a single experiment, and the collected metadata are too complex and stored in multiple files having different formats that are only read by the acquisition software or by customized codes written for internal use."
We now reformulated the sentence as follows p. 7 lines 36-40:
“Moreover, a laboratory can generate a large dataset from a single experiment or a single dataset from multiple experiments, and the collected metadata can be very complex and stored in multiple files with different formats only readable by the acquisition software or by customized codes written for internal use. In such cases, a consolidated strategy for unifying data into a single format that can be read by a variety of software applications and analyzed in an efficient and reproducible manner is necessary.”

1.20 Comment: pg 12, lines 17 - 12, this sentence/paragraph seems to be missing words/doesn't finish "Although many advances have been made regarding organization, annotation, and description of research datasets, for example using the Brain Imaging Data Structure (BIDS, https://smex-ctp.trendmicro.com:443/wis/clicktime/v1/query?url=https%3a%2f%2fbids.neuroimaging.io%2f&umid=69f29401-5290-40bc-943f-fc4fb21e8086&auth=9ed9a254ae8a0a504cee5b89eb68d1de87cd0c46-a38d2b534d1942c51ddbf5754176077e75f11c73) for neuroimaging data standards [10] EEG-BIDS for neurophysiology data (e.g., electroencephalography), [11], or MEG-BIDS for magnetoencephalography data [12], whereas other data modalities (e.g., EMA) do not have existing standards yet.

We thank the reviewer for his feedback. We have now rephrased the sentence as follows p. 5 lines 26-28:
“Although many advances have been made regarding the organization, annotation, and description of research datasets, there is still much work to be done to ensure that datasets are fully standardized and can be accurately shared and reused [18].”

1.21 Comment: pg 15, lines 4 - 7, this sentence seems to me missing words: "However, while choosing an electronic laboratory notebook for a large-scale neuroscience consortium spanning diverse experimental protocols, the availability of clear documentation and application-centric features become an overarching issue."

We apologize for the lack of clarity. We have now rephrased the sentence as follows p. 9 lines 3-5:
“For a large-scale neuroscience consortium spanning diverse experimental protocols, it is important to select an ELN that can provide comprehensive support for a wide range of experimental protocols and provides flexibility to add domain-specific features if required [51].”
2. Reviewer #2

2.1 Comment: The authors of the manuscript "Data management strategy for a Collaborative Research Centre" take the reader on a timely tour de force through the various aspects surrounding the establishment of RDM measures in a large, collaborative research project spanning multiple highly heterogeneous subprojects and partners. I found the topic of the paper highly interesting and engaging, as it is based on the experiences and procedures encountered in a real-world scenario. The challenges and solutions are presented in a clear, well-written and structured way in two overarching chapters, respectively, that make the article enjoyable to read. Also, I found the breadth of challenges well captured. While I am enthusiastic to see the manuscript published, I have several points and suggestions I would like to put up for consideration.
As a general point, I found the article quite lengthy due to a lot of detail that was put into the description of the individual challenges. At the same time, I found many challenges described in the planning phase to be rather high-level statements, and I was often hoping to have these more embedded into the actual problems encountered in the SFB to give illustrative examples. I would like suggest to the authors to reconsider if they find specific examples of some of the more abstract challenges that were actually encountered (similar, e.g., to the last part in "planning" section about specific RDM challenges). The reasoning here is that I find the strong point of this article that it comes from actual experience, and I found this not ideally reflected in some of the more abstract descriptions. An example illustrating this suggestion is given in the detailed points below (p10, l31-32). To compensate, perhaps some of the details in the manuscript could be shorted.
Also, as a general point I found that many of the existing tools/services/... were not referenced (by either publication, DOI, RRDI, https link or at least some pointer to the resource).

We thank the reviewer for the helpful comments. Following the feedback from reviewer 1 and reviewer 2 we have now restructured the whole manuscript. We have moved the data management challenges in the introduction, restructured section 1 (planning phase) and section 2 (implementation phase), and added some examples and some references. We also added a discussion section.

2.2 Comment: - p5, Figure 1: I did not find this illustration very helpful, I think a more standard 2D representation with the individual groups and links between groups forming tandems would be better understandable.

Following the feedback from reviewer 1 and reviewer 2, we now removed the old Figure 1 and edited the old Figure 2 (now Figure 1).

Figure 1: The Heidelberg Pain Consortium investigates various populations: humans, rodents, and tandem using various modalities: neuroimaging, neurophysiology, behavior, psychometrics, and genetics (A). Each data modality can be recorded using different techniques: MRI= magnetic resonance imaging; PET/MR: positron emission tomography/magnetic resonance; fNIRS: functional near infrared spectroscopy; NIBS: non-invasive brain stimulations; EEG: electroencephalography; MEG: magnetoencephalography; Genetics; Two-photon imaging; Behavior; EMA: ecological momentary assessment; psychometrics; physiology; VR: virtual reality; optogenetics (B).

2.3 Comment: - p6, l13: I think it would be extremely useful for other consortia to know more about the day-to-day tasks of the data manager, in particular in terms of the job profile -- what background is more useful: neuroscience, computer science, software engineer, project planning/organization? As the job description of a "data manager" is evolving, it would be great to hear what tasks made this SFB successful in cross-group RDM.

We have now added a sub-section “Data stewards, Community engagements and collaborations” in the discussion section p. 28 lines 37-40, p. 29, p. 30 lines 1-24.

“Data stewards, Community engagements and collaborations

Data managers and stewards are essential for implementation of data policy and governance procedures, especially for large consortiums [115]. These positions are well-suited to individuals with a background in research or computer science, bioinformatics, and strong communication skills. Furthermore, depending on the needs of the consortium, the candidate should have experience developing high-throughput analysis pipelines, domain-specific data structures and standards, open access publishing, and modern data science approaches, high-performance computing environments, cloud computing, data security, and databases, among other things. The role of data manager is diverse, and it works in close collaboration with core computing and library resources to streamline access to the consortium's common research data infrastructure, which is available at the host institutions of participating labs.
Data managers should also support ongoing research, advise on best practices for data handling, and stay informed about current developments in the RDM field. Furthermore, they should act as an important liaison between consortium researchers, collaborators, the university’s RDM planning group and computing center, and community organizations. They aid in bridging the gap between the lab-based scientists and the available technical infrastructure and services. Direct assistance in daily tasks such as data organization, tool selection, workflow development, and standardization is indeed beneficial for individual researchers and research groups. Addressing data management tasks relatively early in research timelines is necessary to make the research process more efficient, and to ensure the interoperability and reusability of data sets. The expert guidance of existing infrastructure and resources, such as scientific repositories, databases, legal and ethical issues, etc., is also necessary to promote an effective data-sharing strategy.
Data managers must maintain consistent communication with various research groups and other similar consortiums to establish a community network and links to other scientific communities such as the national research data infrastructure (NFDI) consortia. As a result, data are disseminated throughout the community, and data management techniques specifically designed to facilitate neuroscience research are developed. For example, our consortium is actively engaged in a number of international and national RDM initiatives, including the EBRAINS ((https://ebrains.eu/), National Node Consortium Germany), the NFDI bioimaging initiative in Germany NFDI4BIOIMAGE (https://nfdi4bioimage.de/), which promotes the development of high-level infrastructure and services across various scientific disciplines. Our involvement with different task areas (e.g., Neuromorphic computing (NMC), Data Analytics, Workflows, GDPR, The Virtual Brain Cloud, etc.) of these community-led initiatives supports the development of a sustainable and community-oriented RDM strategy. Our CRC puts efforts into following the recommendations of The International Neuroinformatics Coordinating Facility (INCF; https://www.incf.org) [116] and harmonizing our RDM efforts by using community-developed standards that have been accepted as international standards for neurophysiology and neuroimaging datasets (for example, BIDS, NWB etc., full list available at https://www.incf.org/resources/sbps). Resources such as FAIRsharing (https://fairsharing.org/ [117] and the UK Digital Curation Centre (https://www.dcc.ac.uk/) provide a comparative view of data and metadata standards. In addition to these domain-specific initiatives, we are engaging with Research Data Alliance (RDA, https://www.rd-alliance.org/) and European Open Science Cloud (EOSC, https://eosc-portal.eu/ [118]) initiatives to adopt and develop new resources for open data exchange across technologies and scientific disciplines.

2.4 Comment: - p6, l23: What level of granularity was put into the guideline in terms of "data formatting"? As outlined in later sections, the data is highly heterogeneous.

The reviewer makes an interesting comment. We now added some information p. 28 lines 22-30:
“The acquisition of heterogeneous data in multiple projects can render the process of data formatting challenging, and the data policy guidelines do not specify a level of granularity for data formatting. However, the guidelines do provide general recommendations for how to format such diverse datasets, such as using standard data formats and tagging data with descriptive metadata. We supported individual research groups by providing resources and funds for the implementation of modern tools and infrastructure for compatibility with community RDM standards. We recommended using "standard" formats for data of similar modalities (e.g., neuroimaging data (MRI) is formatted to NIFTI, electrophysiology data is formatted to NWB, etc.) as described in the data standardization sections.”

2.4 Comment: - p8, l2: How was the compliance to the survey? Given the complexity of RDM, did researchers need prior education wrt the meaning of RDM related questions of the survey, and were measures put in place to improve the quality of answers?

The reviewer raises interesting questions. Due to the collaborative nature of the consortium and the individual development of RDM strategies, the researchers were quite aware of the importance of data sharing and many aspects of RDM in general. Moreover, the head of the symposium supported and encouraged our initiatives, which facilitated the interaction with researchers and the compliance to the survey. Overall, researchers were familiar with RDM concepts and they welcomed help to optimize RDM procedure.

The goal of the survey was to conduct an assessment to better understand the researcher views', their current practices and needs, project-specific support needed from data manager, computing center and university library to establish proper data management practices and their willingness to share data with external collaborators if data manager offers some support in managing their research data. The survey questions are available in the supplementary file. The survey also included questions about data generation, various types and formats of research data generated, infrastructure for data storage and archive, practices of research data sharing, awareness on research data repositories etc. Also, during the second funding period, we focused more on providing support for data standardization and dissemination. Due to the pandemic, we managed to conduct online data seminars and also one on one RDM zoom meetings with each participating lab of the consortium, which helped us to optimize the survey results.

We now added the following text in the manuscript p. 13 lines 33-39, p. 14 lines 1-5:
"Direct involvement with researchers during the planning and initial implementation phases was crucial in order to identify the most helpful RDM measures, these may be as simple as coordinating communication between core IT staff and researchers, and facilitating access to institutional or other pre-existing resources. We devoted significant time to initially gather information about publicly available tools and services that would be useful to the diverse projects within the consortium. Given the large number of laboratories from various institutions participating in the consortium, as well as the increasing number of requirement changes over the course of a project, information was gathered in a variety of ways (virtual individual interviews with project PIs; discussions during online data seminars led by the CRC data manager; and personal meetings with experimentalists and PhD students). Data discussions and regular communication with consortium members have greatly aided our assessment approach. The PIs, or project responsible persons, were required to respond to a variety of data management questions as part of the assessment process (see Supplementary Information 2)."

2.5 Comment: - p8, Figure 2C: I found panel 2C not immediately clear in terms of the organization and identifying the row/column structure. A slight improvement visually and/or 1-2 sentences of description of the panel in the caption would help.

Following the reviewer's comment, we now edited Fig 2 (now Figure 1). See also comment 2.2.

2.6 Comment: - p10, l31-32: This is one concrete example of the general remark above: "manageability issues" are very abstract, an example of what types of problems precisely triggered this statement would be very illuminating and supportive of the statement.

We thank the reviewer for bringing up this important point. Some of these data manageability challenges include integration of multi-modal datasets; harmonization of heterogeneous datasets; keeping corresponding timing information; achieving and maintaining good data quality (checking for missing data, duplicates); properly securing all the collected data and adhering to privacy and security regulations while enabling access to specific users. We added some lines p. 4, lines 16-23:
"These diverse techniques and collected data types raise multiple data manageability issues within and between projects, with broad implications for data interoperability and reuse. Some of these manageability issues are inconsistent data formats, limited harmonization of heterogeneous datasets, time-consuming data acquisition, especially when dealing with large datasets, integration of multimodal datasets, inaccuracy in the representation of collected datasets, difficulties in achieving and maintaining good data quality (checking for missing data and duplicates), ensuring the security of all collected data, and adhering to privacy and security regulations while enabling access to specific users."

2.7 Comment: - p11, l2-: The same as the previous comment holds for the challenges associated with multi-modal data, as its unclear on which level the integration step happens. Moreover, I would welcome a more elaborate description of the level of integration of data in a tandem project: how far could researchers go in integration? Did researchers go as far as analyzing data from their tandem partner and vice versa? To which degree could datasets be actually integrated/compared in practice?

The reviewer makes an interesting comment. The level of integration of data in a tandem project depends on the scope of the project and the goals of the researchers. We now added a section on data integration p. 32, lines 11-40:
"Data integration

For collaborative research, the data integration and standardization step becomes crucial for interoperability and data sharing [125], but is quite challenging to implement, given the wide range of methodologies represented in the consortium. Early data standardization can have massive benefits for data integration in collaborative projects, this can be achieved by streamlining the use of tools for more replicable and reproducible analysis. The data integration process often depends on individual projects and their underlying workflow and processes. The degree to which this is possible will depend on the modalities used, the subject population, and the experimental design. Researchers may also integrate the raw data collected from each partner into a core dataset. Integrated datasets can provide a more comprehensive understanding of the research question, as well as allowing the researchers to compare the results of their analyses more directly. Depending on the modalities used, the data may need to be transformed or normalized before integration, and the analysis techniques may need to be adapted to the combined dataset.
For some projects, researchers can go as far as analyzing data from their tandem partners and vice versa. Some human projects combine fMRI data from one group and EEG data from another group to gain a better understanding of how the two modalities interact. This could also involve combining the datasets, or running analyses on the combined dataset to identify common patterns or trends. However, this process requires careful consideration of the data sources, data formats, and analysis techniques used by individual labs, as well as the selected methods for data fusion and data mining. At the most basic level, researchers can compare the data collected from each partner to identify commonalities and differences in the data. This could include comparing the number and type of modalities used, the subject population, the experimental design, and the type of analysis performed. For example, they could investigate how brain structure (grey matter volume, cortical thickness) relates to behavior. There are also association studies aiming to compare brain activity between two groups of participants (e.g., healthy, chronic pain) in order to explore neural differences in cognition or behavior. They could also examine associations between neural activity in different brain areas and physiological responses of the subject. “

2.8 Comment: - p14, l15-: Did these reproducibility issues occur in practice?

These reproducibility issues did occur indeed in practice. We added some lines p. 7 lines 11-18:
“Problems with reproducibility [37] are made worse by the fact that the original analyses were done in different software environments, using different operating systems (e.g., Linux, Macintosh), and with different software or software versions. For example, variability in reproducing results from neuroimaging studies has been often investigated [38, 39]. Often, analyzing fMRI data with software packages such as SPM (Statistical Parametric Mapping) [40] or FMRIB Software Library (FSL) [41] might lead to different outcomes. Efforts are however being made to determine sources of variabilities and develop homogenous and standardized computing environments [42].”

2.9 Comment: - p15, l1-: One could also mention potential legal/data privacy concerns related to selecting an electronic lab notebook.

We thank the reviewer for the comment. We now added the following information p. 9 lines 22-30:
“When selecting an ELN, there are potential legal and data privacy concerns to consider [52]. An ELN is a digital resource which stores confidential information, and can be accessed by multiple users. To ensure legal and data privacy compliance, it is important to design ELNs compliant with all applicable laws, regulations and ethical standards. Additionally, it is important to consider the security measures in place for the ELN, including encryption and user authentication measures, to ensure that the stored data remains secure and private. Finally, it is important to consider the terms of service for the ELN, as this will outline how the data is used, stored and shared, as well as any limitations on the use of the data. By considering these potential legal and data privacy concerns, organizations can ensure that their data remain secure and compliant.”

2.10 Comment: - p17, l24: Please reformulate or define "neural assemblies", as this may be ambiguous

We now defined neural assemblies p. 13 lines 3-40:
“neural assemblies (collection of neurons that are activated simultaneously in response to a particular stimulus and form assemblies)”

2.11 Comment: -p17, l15-: I really like this last subsection as it is very concrete and down-to-earth. However, for this paragraph in the section, I found it unclear where exactly the special challenge lies, i.e., what aspects are truly unique to

this situation. Are similar problems not equally present, e.g., when looking at the neuropixel probes (p17, l1-) where different spike sorters will produce different results, and potentially similar computationally demanding workflows are required?

We thank the reviewer for the positive comment.
The section “Special RDM challenges” was added because it includes some DM strategies that were specific to some animal projects, whereas the previous section “Common data management challenges across projects” deals with common data collected across the majority of the projects. As the reviewer points out, challenges for neuropixels and two-photon calcium imaging are comparable in terms of size of data sets generated (around 100 GB/hour), which provides some challenges for data storage. Moreover, producing and analyzing such datasets requires high computational complexity and costs, necessitating an adequate computational infrastructure [34, 35].
We separated these two techniques because they differ in their spatial sampling and temporal resolutions, and are used to answer to different research questions.
To facilitate comprehension, we now changed the title of the subsection to “RDM challenges for specific projects” see p. 11 line 23. We also reformulated the sections on Electrophysiology and neuroimaging p. 12, lines 15-40 and p 13 lines 1-12:
“Electrophysiology with high-density probes
A few of the animal projects in the consortium make use of new technologies such as high-density Neuropixels probes [63]. Neuropixels datasets are often large (~80 GB/hour) and computationally demanding, which can make it difficult to scale spike sorting workflows across different labs and datasets. Data storage requirements rise as a result of the significant amounts of derived data needed for intermediate processing (such as filtering and spike sorting) and stimulation and/or behavioral parameters (such as optogenetic stimulation, motion or whisker tracking, and task performance). Analysis and post-processing may often require computationally intensive algorithms and hardware acceleration to handle data that cannot be loaded into local memory [64]. Due to differences in recording conditions, spike sorting algorithms and data preprocessing, there can be significant variability in the results produced by different spike sorters. Real-time processing requirements for closed-loop experiments only serve to exacerbate these issues. Important parameters initially recorded from raw data, e.g., animal arousal/anesthesia level, impedance measurements, can be discarded in derived datasets used for analysis. Complex hierarchies of derived data and multimodal datasets (e.g., accelerometer, whisker or pupil tracking, etc.) collected with different instruments compound these issues. It can be challenging to validate and reproduce results, as the various algorithms and parameters used can produce different outcomes.

Large-scale in vivo two-photon calcium imaging
Some rodent projects within the consortium acquire large amounts of data collected over months [65]. For example, data acquisition using imaging techniques such as fluorescence imaging or two-photon microscopy calcium imaging (2P imaging) generates large sets of spatiotemporal imaging data (up to 100 GB/hour) and requires rigorous preprocessing steps (image segmentation, denoising, motion correction, manipulation and handling of large video files, and neural activity deconvolution) using high-throughput computing [66]. The downstream processing and analysis of the resulting datasets generated over the course of months is often challenging and requires complex workflows [67]. A few open-source software solutions (CalmAn [68], EZCalcium [69], etc.) have been proposed to deal with these challenges. However, comparative analysis studies have revealed that the neural assemblies (collection of neurons that are activated simultaneously in response to a particular stimulus and form assemblies) recovered from 2P imaging datasets can vary significantly depending on the algorithm used, and that certain algorithms are more reliable and faster than others [70]. Specific algorithms have been found to have higher precision and longer run times than others, while others have been found to have faster run times but lower accuracy. Another issue is that many studies include synthetic or benchmarking datasets, but producing and analyzing these datasets requires challenging calculations, raising the computational complexity and costs. This enables the need for more scalable and fully automated workflows that can be run on HPC clusters [65], which requires ensuring the reproducibility of studies [71]. Existing software solutions can be used for analysis and visualization of datasets, but any adopted data and metadata standards must be interoperable with these tools.”

2.12 Comment: - p20, l1-: What distinguishes the three templates? Are DMPs considered living documents that accompany the project duration, and if so, how are they followed up?

To answer to the reviewer’s questions, we now added the following information p. 17 lines 24-26, p. 18 lines 1-17:
“Data Management Plans for CRC projects
We designed three DMP templates after defining and categorizing the data management needs for each project depending

on its experimental model type: human, animal, and human-animal tandem (see supplementary information 3.1, 3.2, and 3.3, respectively). The templates can also be found on Zenodo: https://doi.org/10.5281/zenodo.4410128), [44]. DMP templates for animal, human, and tandem projects can differ depending on the scope of the project, the type of data collected, and how the data will be managed, but the major difference is the ethical considerations associated with each type. Animal projects, for instance, may necessitate additional safety protocols for the storage and management of animal tissue samples, whereas human projects require more stringent regulations and oversight for the ethical processing of human data [45]. Additionally, DMPs for human projects may include the collection of sensitive data (personally identifiable information), which must be securely stored and shared according to regulations. The DMP should follow General Data Protection Regulation (GDPR) -compliant guidelines for handling of personal data and specify information, authentication measures for reuse and recovery, de-identification of datasets involving human participants before sharing, and publication process for anonymized data. Human-animal tandem projects are more concerned with resource allocation and special protocols for the integration of different data types collected from multiple sources. It is important to ensure accuracy and consistency across all sources.
The DMP states minimum requirements for metadata that must be provided for long-term preservation and secondary analysis of research data. It also contains the information about data migration and access by third parties or future collaborators even after the project ends."

2.13 Comment: - p20, l21-: I found the listing of storage locations somewhat confusing. I would suggest to list first, the available options, underlying technology (e.g., nextcloud, ssh,...) and their properties (e.g., sharing possibilities, availability on HPC, backup, versioning, access,..) and, second, how these factors influenced the choice for labs in the consortium. Which features where actually decisive?

We thank the reviewer for the suggestion. We have now restructured sections on "Data storage" (p. 18 lines 19-40, p. 19 lines 1-37) and "Data processing, analysis, and visualization" p. 19 lines 39, p. 20, p. 21.
"Data storage solutions
Projects in the CRC generate massive volumes of data of various types and rely on data interoperability among labs. It is strongly advised that researchers securely store full datasets (e.g. raw, preprocessed, and analysis files, codes, etc.) associated with published findings and results, as this promotes the consortium's goal of further engaging in open science. The CRC provides support for various storage solutions for all stages of the data life cycle, and the best option for a research project is determined by the type of data being collected, the size of the data, the security requirements, and the scalability of the platform. Factors such as interoperability with the existing infrastructure of the project group, reliability and accessibility for a particular storage solution, and availability of user-training and service support are also taken into consideration. For example, if the research project involves collecting large amounts of data, then a cloud-based storage platform may be the best option. Additionally, cloud-based storage platforms provide access to data from anywhere with an internet connection, making it easy for researchers to collaborate and share data with colleagues. If the data is sensitive, then a secure, on-premise storage solution may be the best choice because it can be customized to meet the specific security needs of the organization, such as encryption, authentication, and access control. Additionally, if the research project (e.g., projects collecting terabytes of data from methods such as optogenetics, electrophysiology, and calcium imaging etc.) requires scalability, then a platform that can easily scale up or down may be the best option. Ultimately, the best data storage platform for a research project will depend on the specific needs of the project, and university-approved data storage services (see Figure 2) are recommended to guarantee data privacy and confidentiality.

Most of the CRC researchers are encouraged to use our internal data storage platform SDS@hd (SDS, Scientific Data Storage, with a capacity of 20 PetaByte), a central service for securely storing scientific data https://www.urz.uni-heidelberg.de/en/service-catalogue/storage/sdshd-scientific-data-storage . To facilitate easy data sharing between internal collaborators, all datasets collected from rodent experiments in collaborative projects are shared across different research teams via SDS@hd, which stores experimental protocols, raw and pre-processed data, session, and mouse information (e.g., session start time, animal weight, etc.), task files (e.g., behavioral responses, videos, audio files), acquisition metadata (e.g., dimensions, pixel types, and instrumentation settings), in a shared directory structure for project members. It is intended to be used for data that are frequently accessed ('hot data'). A common storage place ("Speichervorhaben" (SV) meaning data storage projects) is requested for collaborative projects or work groups to ensure that data are easily accessible to all project members with proper authentications (university credentials). The Heidelberg University network connects the consortium's labs and university departments, using a 10 GB capacity network to expedite data transfer among institutes and facilities. Using SDS@hd drastically increases the ease of data access for shared projects and data safety by virtue of automated mirroring for data backup. Datasets collected from confocal or two-photon microscopes, for example, are stored on acquisition computers for a few days until they are stored on to

SDS@hd. Once transferred to SDS@hd, individual users must ensure that the large datasets are timely (usually in few days or a week depending on data volume) removed from acquisition systems to free up the imaging acquisition system for new data or for another user.

Apart from its storage and fast data transfer capacity, another potential reason for storing large datasets on SDS@hd is its direct access to other university platforms such as HPC systems (explained in next section). Other university storage solutions that can be requested via support from CRC data manager include a SERVER BACKUP (https://www.urz.uni-heidelberg.de/en/service-catalogue/storage/server-backup, service for data storage for servers (based on a data protection and recovery software i.e. IBM Spectrum Protect (ISP) https://www.ibm.com/support/pages/overview-ibm-spectrum-protect-supported-operating-systems), CLIENT BACKUP service (https://www.urz.uni-heidelberg.de/en/service-catalogue/storage/client-backup (can be access via on all operating systems via Duplicati software) for secure storage of workstations and PCs, and HEIVOL-I (https://www.urz.uni-heidelberg.de/en/service-catalogue/storage/heivol-i), a service for creating network drives for university institutes and facilities. Similar services are available at the other participating CRC labs from other institutions.

For human data storage (including sensitive data), researchers use a storage server with restricted access (Dell Isilon server with a large storage and archival capacity). Designated personnel have the authority and responsibility to enable access to internal collaborators. When necessary, access can be given to external collaborators by assigning guest accounts with a data sharing agreement in place.

Data processing, analysis, and visualization

Several projects and laboratories in the consortium use laboratory-based analysis infrastructure, such as local computers, shared analysis workstations (laboratory computers with GPUs and pre-installed acquisition and analysis tools that are shared by several members), and computational servers that are run by individual laboratories or groups.

The CRC highlights the importance of keeping track of every step, from initial data recording to the analysis, and proper documentation of analysis code, pipelines, and scripts. As a constructive starting point, the CRC 1158 data manager has set up a dedicated code space on Github (https://github.com/CRC 1158RDM) where multiple repositories with analysis code and scripts can be hosted and shared for each CRC 1158 project. The CRC 1158's data management organization repositories are maintained by the data manager, and access is given only to the authorized project members.

In addition to local infrastructure and computing servers, there is university infrastructure available for more demanding data processing tasks, such as running computationally intensive analyses of heterogeneous and large-scale imaging datasets collected from humans and rodent projects. An application for access to these services can be made by individual laboratories with an initial application and usage support from the data manager. To seamlessly integrate the data analysis with the setup and execution of preclinical experiments, a platform such as the one proposed here was necessary. Some of the CRC projects are utilizing bwForCluster MLS&WISO (https://www.urz.uni-heidelberg.de/de/service-katalog/hochleistungsrechnen/bwforcluster-mlswiso) and a detailed tutorial on access and use is made available (see https://github.com/SFB1158RDM/HPCtutorial). This eliminates administrative and technical barriers to performing computationally intensive tasks such as large-scale modelling, simulation, and analysis projects, e.g., Neuropixels systems. The HPC allows job scheduling using SLURM (https://slurm.schedmd.com/documentation.html) and also sets up reproducible computing environments (e.g, Docker [83], Singularity [84]) to optionally run the modules on the HPC for particularly large data sets that are streamed directly to SDS@hd during acquisition, e.g., chronic recordings with dense electrode array or image segmentation for chronic imaging (miniscope) with the perspective of running standardized analysis workflows. Allowing users to access large datasets stored in SDS@hd without downloading them to their local computer aids in the seamless integration of data analysis procedures, saving overall computational time and costs. The data can be accessed from the bwHPC cluster using the same protocols as for local storage, such as NFS, SMB, and FTP. This service also allows users to access data stored in SDS@hd from multiple bwHPC nodes simultaneously, thus increasing overall speed of data access. By utilizing direct data access, scientists are able to take advantage of the increased computing power available in HPC systems and gain access to data stored in a scientific data storage system. While writing this manuscript, bwForCluster Helix https://www.urz.uni-heidelberg.de/de/service-katalog/hochleistungsrechnen/bwforcluster-helix, a successor of the current HPC system, was made available to users. The Helix component will enable the use of seamless, cross-system workflows for processing and analysis of large amounts of data.

In our consortium's case, data management efforts had led to an increased user base for the HPC machines. Many factors played into a user's decision to use a particular HPC machine, such as its performance, cost, and availability. Data management efforts had made the HPC machines more attractive to users by providing individual support and training for access and use. The amount of training necessary to promote the use of HPC machines in a research lab depended on the particular needs and existing infrastructure. Generally, training and seminars covered topics such as high-level usage of programming languages, high-performance computing paradigms, and best practices for using HPC machines for neuroscience data processing and analysis. It also included guidance on how to design and optimize applications for HPC

systems (e.g., refactoring of tools for direct access or use on HPC and bwVISU). A lab might also need to provide additional training for data management and analysis, or for using specific software packages.
Similarly, for processing massive (e.g., neuroimaging) datasets, some projects are utilizing the heiCLOUD (https://heicloud.uni-heidelberg.de/), an Infrastructure-as-a-Service (IaaS) cloud service that provides virtual machines that may be customized and utilized as needed for the project. A possible scenario for heiCLOUD usage within our consortium is to install complex and computationally expensive software packages and perform concurrent processing of massive neuroimaging datasets. This provides powerful workstations for data analysis and can be especially useful for collaborative research projects that require data sharing between multiple research teams.

Another service that is frequently accessed by CRC members is heiBOX (https://www.urz.uni-heidelberg.de/de/service-katalog/collaboration-und-digitale-lehre/heibox), a secure Sync and share service hosted on heiCLOUD. This service is similar to commercial cloud storage services like Dropbox and Google Drive, and allows users to save, synchronize, share, publish, and jointly edit files. heiBOX is based on the Seafile software (https://www.seafile.com/en/home/), and allows users to search for text files, PDF files, and Office files in unencrypted libraries using a full-text search. Additionally, Office files can be edited by multiple people, and files and folders can be tagged and commented on. Markdown documents can be used to create private or public wikis. heiBOX also provides backup, synchronization, and storage of small research data and document files, and guest accounts can be requested for data exchange with external collaborators. The most common use of heiBOX in our consortium is to share documents such as CRC meetings notes, data seminar and workshops presentations, individual projects DMPs, manuscripts and figures.
Another application that some of the CRC projects are utilizing is bwVISU (https://www.bwvisu.de/), a remote service for scientists (universities in Baden-Württemberg state, Germany), as well as the corresponding software stack to deploy such a service on-premises. It has an interactive web front-end that supports large-scale data analysis and visualization without much human intervention. Our RDM services also include providing technical assistance with the refactoring of lab-customized preprocessing analysis pipelines (MATLAB and Python scripts) into more organized workflows/Graphical User Interface (GUI) that can be run on HPC applications.

2.14 Comment: - p21, l7: NFDI-Neuro is currently a non-funded consortium.

The reviewer is correct. We were part of two funding applications and we look forward to working collaboratively with other active members of the consortium in the near future.

2.15 Comment: - p21, l11: GIN is not linked at its first occurrence.

We now changed the link p. 34 line 10
"GIN (https://gin.g-node.org/)"

2.16 Comment: - p21, l15: How do the folder structure templates relate to standards such as BIDS?

Folder structure templates for research repositories provide a means of following BIDS standards, as well as other standards, while keeping data organized and accessible. They provide a common way of organizing data that can be used by all researchers, regardless of the tools they are using. These folder structure templates can be customized based on the type of experiment or data modality as well as the analysis processes that should be integrated with existing data organization systems.
We now added the following information p. 30 lines 17-24:
"Neuroimaging data stored in the BIDS convention, for example, can be saved in the section "03_data" for the raw data (e.g., nifti and JSON) and in the section "04_data_analysis" for derivatives (i.e., analyzed data). These templates, which are mostly workflow-based, are customizable to meet special requirements for individual experiments and analyses, ensuring that the structure may support rather than restrict research practices. These folder configurations can be utilized on desktop PCs, data versioning systems (e.g., GIN or DataLad), external hard drives, or any storage device to accommodate various data sets generated during experiments, independent of their format. "

2.17 Comment: - p21, l24: Similar as above, it would be of interest to better understand the technological foundation and

usage scenario of HeiBox.

We have now described heiBox p. 21 lines 21-32:
“Another service that is frequently accessed by CRC members is heiBOX (https://www.urz.uni-heidelberg.de/de/service-katalog/collaboration-und-digitale-lehre/heibox), a secure Sync and share service hosted on heiCLOUD. This service is similar to commercial cloud storage services like Dropbox and Google Drive, and allows users to save, synchronize, share, publish, and jointly edit files. heiBOX is based on the Seafile software (https://www.seafile.com/en/home/), and allows users to search for text files, PDF files, and Office files in unencrypted libraries using a full-text search. Additionally, Office files can be edited by multiple people, and files and folders can be tagged and commented on. Markdown documents can be used to create private or public wikis. heiBOX also provides backup, synchronization, and storage of small research data and document files, and guest accounts can be requested for data exchange with external collaborators. The most common use of heiBOX in our consortium is to share documents such as CRC meetings notes, data seminar and workshops presentations, individual projects DMPs, manuscripts and figures. “

2.18 Comment: - p22, l7: I am not certain datalad is properly represented by this description. Does datalad itself offer storage? While I am not expert, I had seen datalad more as an abstraction layer to handle versioned datasets and workflow execution, largely focused on git-based versioning.

We took account the comment of the reviewer and edited some lines p. 37 lines 8-22:
“There are platforms such as DataLad (a US-German collaboration for computational neuroscience project (https://www.datalad.org/) and GIN (https://gin.g-node.org/) that may effectively compensate for a lack of local resources. These data hosting and sharing platforms can also ensure data versioning and encourage reproducible management of scientific data. Both DataLad and GIN are based on git and git-annex to provide a decentralized system for the exchange of large datasets. Datalad and GIN are interoperable, as datasets hosted on either of these platforms can be accessed via git-compatible systems. Datalad is an open source software package for the management of distributed datasets. It facilitates the sharing, retrieval, and organization of data in a distributed environment. Moreover, the GIN service can be deployed locally at all the participating labs and can be used as an in-house storage server and web user interface for DataLad datasets. Datalad does not offer storage, but it provides tools for the acquisition, organization, and management of data stored in remote repositories.”

2.19 Comment: - p22: How were storage locations suggested to consortium members? What role did HPC play in this process?

We have now addressed the points raised by the reviewer p. 18 lines 25-40 and p. 20 lines 29-40:
“The CRC provides support for various storage solutions for all stages of the data life cycle, and the best option for a research project is determined by the type of data being collected, the size of the data, the security requirements, and the scalability of the platform. Factors such as interoperability with the existing infrastructure of the project group, reliability and accessibility for a particular storage solution, and availability of user-training and service support are also taken into consideration. For example, if the research project involves collecting large amounts of data, then a cloud-based storage platform may be the best option. Additionally, cloud-based storage platforms provide access to data from anywhere with an internet connection, making it easy for researchers to collaborate and share data with colleagues. If the data is sensitive, then a secure, on-premise storage solution may be the best choice because it can be customized to meet the specific security needs of the organization, such as encryption, authentication, and access control. Additionally, if the research project (e.g., projects collecting terabytes of data from methods such as optogenetics, electrophysiology, and calcium imaging etc.) requires scalability, then a platform that can easily scale up or down may be the best option. Ultimately, the best data storage platform for a research project will depend on the specific needs of the project, and university-approved data storage services (see Figure 2) are recommended to guarantee data privacy and confidentiality.”

“Allowing users to access large datasets stored in SDS@hd without downloading them to their local computer aids in the seamless integration of data analysis procedures, saving overall computational time and costs. The data can be accessed from the bwHPC cluster using the same protocols as for local storage, such as NFS, SMB, and FTP. This service also allows users to access data stored in SDS@hd from multiple bwHPC nodes simultaneously, thus increasing overall speed of data access. By utilizing direct data access, scientists are able to take advantage of the increased computing power available in HPC systems and gain access to data stored in a scientific data storage system.
While writing this manuscript, bwForCluster Helix https://www.urz.uni-heidelberg.de/de/service-

katalog/hochleistungsrechnen/bwforcluster-helix, a successor of the current HPC system, was made available to users. The Helix component will enable the use of seamless, cross-system workflows for processing and analysis of large amounts of data. "

2.20 Comment: - p23, l2-4: I think https://eln-finder.ulb.tu-darmstadt.de/home is a more recent and complementary resource to the Harvard spreadsheet, that should be also mentioned here.

We thank the reviewer for the information, which we added p. 34 line 29 .

2.21 Comment: - p23, l6-: How was the adoption of a common ELN received in labs? How difficult was the integration into lab workflows (especially for complex experiments) in terms of automation, such that ELNs were not perceived as additional burden?

The reviewer raises important questions, which we now provided some answers in the manuscript p. 34, line 30-40, p. 35, p. 36 lines 1-5:
"In our consortium, Individual researchers have benefited greatly from our computing center's local installation of the elabFTW, which includes secure cloud-based data storage. The service can also be used to serve as a central repository for documenting shared or collaborative experimental methods or protocols used by two or more CRC labs. Each project can have its own team, with members from participating labs having access to it. Transfer of these protocols from traditional notebooks or digital documents into ELN needed additional time and effort from project members. In order to ensure that the ELN is not seen as an additional burden, careful consideration had to be taken when automating the workflow. For example, the ELN must be designed to integrate seamlessly with existing protocols and should provide easy access to data at all stages of the experiment. Overall, most labs find that the benefits of ELNs outweigh any additional burden, as the system can save time and effort in the long run by automating data entry and providing easy access to protocols and data.
Within our RDM framework, ELN can be used as a platform for documenting minimal metadata that is generated automatically while performing an experiment. ELN are frequently not developed as full-fledged metadata systems and lack domain-specific features (such as support for reading or viewing neuroscience-specific file formats). Another issue is that most datasets lack consistent metadata schemas, even at the most basic level. In this particular instance, we use elabFTW, which accepts JSON files. elabFTW acts as a "notebook," tracking both primary data (experiment findings, measurements, etc.) and metadata (date, time, author, units, used inventory, etc.). The experimental metadata (e.g., microscope specifications, data acquisition settings) is stored in a standardized manner using a generic metadata file format that is compatible with open file formats, such as JSON or XML. However, from our experience, the integration of metadata from complex experimental protocols, and analysis procedures into an ELN has proven to be a challenge. Support for various data or metadata file formats generated during acquisition and analysis is not within the scope of elabFTW. Users frequently choose to standardize their datasets at any stage of their project lifecycle; by this, we mean that users standardize datasets either immediately after data acquisition (raw files), or during the pre-processing or final analysis stage before sharing or publishing. Adding any additional metadata information into the ELNs (e.g., elabFTW JSON file) without an automated tool or API can also be time-consuming. So, if a user needs to add, search, filter, or use various types of metadata on a regular basis, they will require a specialized tool.
To address these issues, we emphasized the importance of developing templates for the most common types of experiments performed in a single project (based on design protocols, biological methods, and so on). The development of a metadata GUI also enabled us to automate the process of metadata standardization in an experiment-specific manner.
We would also like to highlight some of our consortium's use cases. The experimental protocol for a project that collected a large amount of electrophysiological data was available on elabFTW, along with a JSON file containing basic experimental metadata. We made the decision to standardize raw data files such as .rhd and convert them to NWB format. On elabFTW, the basic experimental information/metadata was available, but not in a format compatible with NWB metadata. When we standardize raw data files, we frequently discover that the associated metadata files lack analysis parameters. The challenge now is to integrate all types of extensive metadata (experimental, acquisition, and analytical metadata) in a single file with a consistent format to ensure image data quality, reproducibility, and scientific outcome.
In this case, the metadata GUI's goal was to generate a JSON file based on custom JSON schemas substantial enough to generate a NWB file for a given experiment. These predefined JSON schemas are built around a specific experiment setup, such as intracellular electrophysiology data collected from the Intan RHD acquisition system. Once the JSON-schemas are created, all the labs conducting similar experiments could use these schemas to feed metadata collected from various acquisition systems and generate a JSON file. This also allows for customized validation of the data, which can ensure that

the data is valid and accurate before being entered into the ELN. By specifying the data in a custom GUI, a more efficient and reliable workflow within the lab is enabled, as well as ensuring that all necessary information is included and kept in a consistent format.
Another scenario is where users can use the bioformat library to extract metadata from TIFF and other imaging file formats into JSON format, then add any additional fields using a custom tool and feed this file back into elabFTW if necessary. "

2.22 Comment: - p24, l2-4: I did not understand this sentence. Which structure and function in which populations?

We apologize for the lack of clarity. We now added the following information p. 22 lines 29-31:
"For example, we can directly compare the structure and function of various patient populations acquired in different projects, e.g., to examine commonalities between individuals with chronic back pain and fibromyalgia patients."

2.23 Comment: - p24, l15: I found the purpose of the volunteer platform somewhat unclear. Which problem concretely does it solve, i.e., which type of redundant data acquisition does it help to avoid?

We have now clarified the use of the data platform p. 31 lines 30-40, p. 32 lines 1-7:
"We are currently expanding our collaborative efforts by creating a data infrastructure platform that will establish a GDPR-compliant data registry (PainReg-registry, based on the Germany-wide ParaReg registry (www.parareg.de) [124] for human volunteers. To facilitate cross-project data merging, a core clinical data set will be defined. This entails assigning a unique identifier to each study participant that is shared by all projects, allowing researchers to determine whether the volunteer participated in multiple projects. This will allow organizations to share the same pool of volunteers for multiple studies, resulting in less redundant data acquisition. This can result in cost and time savings, as well as increased data collection accuracy. We, for example, experienced that a same study participant could be tested twice, and was assigned different IDs (belonging to different projects), resulting in redundant data acquisition and therefore unnecessary increased costs, particularly for genetic analysis.
Furthermore, the data registry will ensure that data privacy regulations are strictly followed by obtaining participants' consent to access data for secondary or follow-up studies. This will also include an identity management feature to limit access to authorized users. The registry will contain a wide range of data, including brain imaging, genetic, cognitive, and physiological data. This collaborative work will be coordinated by the consortium's future data infrastructure project, which will be tasked with implementing, testing, optimizing, and standardizing data analysis procedures and models that will be utilized in all projects."

2.24 Comment: - p24, l26-27: It was not clear to me how BIDS reduces manual metadata input.

Following the reviewer's comment, we have now added the following information lines p. 23 lines 5-15:
"In addition, using BIDS standards for the organization of MRI data enabled a consistent metadata structure, minimizing the number of manual inputs of metadata required and, hence, reducing the number of errors arising from misinterpretation of those inputs. For example, a typical MR brain acquisition includes numerous different protocols, such as anatomical and functional. In BIDS, metadata fields common across all subjects are specified in a single JavaScript Object Notation (JSON) file in the root directory instead of multiple files repeated for each subject. By organizing the data in this way, researchers can easily and quickly access the relevant information without having to manually input the metadata. Moreover, adoption of BIDS enabled the development of workflows for automated data extraction, curation and labeling [49]. For example, automatic extraction of a minimal set of BIDS compatible metadata can be performed using dcm2niix (https://www.nitrc.org/projects/dcm2nii/)."

2.25 Comment: - p25, l4: Typo: ses->license

We have now fixed the typo p. 23 line 27.

2.26 Comment: - p25, l28-32: Typo: Sentence seems broken in the listing of tools.

We have now fixed the typo p. 34 lines 7-12:
"This includes the Neuroscience Information Exchange (NIX) Format [139], Neurodata Without Borders (NWB; https://www.nwb.org) [93, 140] etc., data versioning tools (e.g., DataLad (https://www.datalad.org/) [141], and GIN (https://gin.g-node.org/), metadata collection tools (e.g., CEDAR, NIDM, Open metadata markup language (odML) [43], data representation models (Neo) Python library Electrophysiology Analysis Toolkit (Elephant), data analysis tools (e.g., Elephant, FieldTrip), PyNN [142, 143], etc"

2.27 Comment: - p26, l10-: This paragraph left me very confused. If ELNs are adopted (which have a primary function of enabling users to fill in metadata records via a configurable GUI) why then are metadata entered first in a custom GUI that then produces a JSON that is then fed into the ELN? Would it not be more straight forward to enter metadata in ELN-defined schemas, then export these to JSON (in case they are required outside the ELN)? Also, why is this procedure ephys-specific?

We apologize for this lack of clarity. We began our efforts with standardizing electrophysiology datasets into NWB, which is why the current GUI supports electrophysiology experiments. However, the GUI is extensible and can be extended to provide support for other data types simply by incorporating custom JSON schemas for other experimental types.
We have now added some liens p. 35 lines 33-40, p. 35 lines 1-2:
"In this case, the metadata GUI's goal was to generate a JSON file based on custom JSON schemas substantial enough to generate a NWB file for a given experiment. These predefined JSON schemas are built around a specific experiment setup, such as intracellular electrophysiology data collected from the Intan RHD acquisition system. Once the JSON-schemas are created, all the labs conducting similar experiments could use these schemas to feed metadata collected from various acquisition systems and generate a JSON file. This also allows for customized validation of the data, which can ensure that the data is valid and accurate before being entered into the ELN. By specifying the data in a custom GUI, a more efficient and reliable workflow within the lab is enabled, as well as ensuring that all necessary information is included and kept in a consistent format."

2.28 Comment: - p26, l10-: Which types of metadata are we talking about -- generic or experiment specific?

We mean both type of metadata, i.e., generic and experimental. Here are some available examples: https://github.com/catalystneuro/heidelberg-metadata-gui/tree/main/heidelberg_metadata_gui/examples

2.29 Comment: - p27, l15: I would argue that while TIFF is a complex/potent file format, it's not really propitiatory. Also, the number of image file formats is not particularly large. Therefore, I would argue, the situation is somewhat different from neurophysiology.

We agree with the reviewer and made some changes to describe our efforts in targeting open formats for microscopic imaging datasets. Indeed we realized that the data harmonization process is quite different for neuroimaging and neurophysiology but it is indeed difficult to argue about the complexity in data standardization between these two modalities as there are several contributing factors. In our experience, it really depends on the diversity of acquisition systems and software used within an organization. Also, in many cases, neurophysiology often requires more complex data analysis than microscopic imaging, which can require additional file formats for storing and analyzing the data. Evolution of BIDS format and its extension to support other modalities such as PET, EEG, MEG and microscopic imaging data (Microscopy-BIDS) as well as for electrophysiology datasets are also contributing to our current standardization efforts. For example BIDS-animal-ephys, https://neuroinformatics.incf.org/node/242) formats is not fully developed yet, but there is an ongoing effort from the INCF working group (https://incf.org/sig/incf-working-group-standardized-data) towards achieving interoperability between NWB, NIX and BIDS formats.
We added some lines p. 25 lines 8-13:
"Researchers collect and view microscopic imaging data from different vendor specific acquisition software in varying file formats (e.g., TIFF, multi-page TIFF, Nikon ND2, Leica LIF, Leica CZI or ZVI etc). It is often difficult to read metadata from these files in other softwares. While TIFF is the most commonly used file format because it is easily accessible by many current analysis software platforms, it has some limitations such as long latency and delayed data access while working with large batches of files [96]."

2.30 Comment: - p29, l15-: In this paragraph, it was unclear to me if the data management efforts actually led to an

increased user base of the HPC machines -- or if essentially those groups used the HPC resources who know how to use them? In other words, how much training was necessary to promote use of HPC machines?

The reviewer raises interesting points. We have now added some information p. 21 lines 1-11:
“In our consortium's case, data management efforts had led to an increased user base for the HPC machines. Many factors played into a user's decision to use a particular HPC machine, such as its performance, cost, and availability. Data management efforts had made the HPC machines more attractive to users by providing individual support and training for access and use. The amount of training necessary to promote the use of HPC machines in a research lab depended on the particular lab's needs and existing infrastructure. Generally, training and seminars covered topics such as high-level usage of programming languages, high-performance computing paradigms, and best practices for using HPC machines for neuroscience data processing and analysis. It also included guidance on how to design and optimize applications for HPC systems (e.g., refactoring of tools for direct access or use on HPC and bwVISU). A lab might also need to provide additional training for data management and analysis, or for using specific software packages.”

Close
