# Supplementary material for: Data management strategy for a collaborative research center
Source: Gigascience. 2023 Jul 4;12:giad049. doi: 10.1093/gigascience/giad049 (PMC10318494; doi:10.1093/gigascience/giad049)
Supplement: giad049_Response_to_Reviewer_Comments_Revision_1 [file giad049_response_to_reviewer_comments_revision_1.pdf]

Author's Response To Reviewer Comments

Close

Reviewer reports:

1. Reviewer #1:

The manuscript is much better after the rearrangement. I have only a couple minor comments:

1.1 Comment: On page 6, lines 9-10 I can't quite understand this sentence, please consider rephrasing: "However, the data-driven neuroscience approach, as a whole, is questioned by a number of technical issues that must be addressed before it can be fully realized."

We thank the reviewer for the comments. We now rephrased the above-mentioned sentence, see p. 6 lines 4-5: “However, the data-driven neuroscience approach faces several technical issues that need to be resolved before its full potential can be realized.”

1.2 Comment: On page 8, lines 24-33 there is a discussion of cloud resources that does not ring true to me.

We thank the reviewer for the comments. We now revised this section with more up-to-date information, p. 8, lines 27-37; p.9 lines 1-11: “Commercial cloud storage solutions are available, offering a wide range of general-purpose data backup and restoration services. However, their adoption may be challenging and limited for individual research labs or universities due to differences in data types, volumes, privacy regulations, and budgets [51]. Cloud solutions can be expensive, especially when large amounts of data or complex computing workloads need to be stored. Besides, many cloud providers may lack the necessary APIs, scripts, and tools to facilitate data migration onto analysis platforms, and may not have sufficient data protection support for sensitive data (e.g., clinical data) [52].
Handling protected clinical data requires additional layers of security, privacy, and regulatory compliance. Universities may deal with sensitive data, such as research data, or intellectual property, subject to strict regulations such as the EU and the General Data Protection Regulation (GDPR, https://gdpr-info.eu) and the Health Insurance Portability and Accountability Act (HIPAA). Cloud solutions require a reliable and fast internet connection, and universities may have limited bandwidth that affects research activities. Besides, it can be difficult to migrate to a different provider or back to on-premises infrastructure, resulting in long-term dependency on a single vendor and the associated risks. Despite these challenges, cloud solutions can offer significant benefits, but research groups should evaluate their data protection needs, choose compliant cloud storage and backup services, and take steps to ensure that data are stored securely and in compliance with any relevant regulations or institutional policies. To meet these requirements, cloud providers have developed dedicated platforms and specialized backup and storage solutions designed specifically for healthcare organizations. However, these services cannot be easily implemented or adopted by individual labs or consortiums.
Assuring access to secure and optimal storage solutions that can be integrated with workflows encompassing data acquisition, intermediate analysis, and archiving is thus a major challenge.”

2. Reviewer #2

The authors have performed a detailed reworking of the manuscript, answering to most of my suggestions and concerns. I still find that, perhaps due to the length of the text, there are a number of issues left to fix. Primarily, I urge the authors to very carefully and diligently go through the text again as there seem to be a number of inconsistencies, typos, missing links to tools... left to fix, many of which I probably did not

spot. For example regarding inconsistencies, sometimes tools are mentioned briefly in one place, and only later when they appear again in the text the tools are introduced in more detail and with a link. Also, I had the feeling that there would be some space for streamlining the text a bit, maybe cutting on some of the more obvious and less CRC-specific aspects of data management. I would advise to have another fellow reader go through the text to best spot potential redundancies.

We thank the reviewer for the detailed review and valuable feedback. We appreciate the reviewer’s thoroughness in identifying areas where we can improve the manuscript. We carefully checked the text again and addressed the inconsistencies, typos, and missing links to tools that you have mentioned. We also reviewed the text to see where we could streamline and remove any less relevant information. Additionally, we asked another fellow reader to go through the manuscript to identify potential redundancies.
We thank you for taking the time to review our work and for providing us with constructive feedback that helped us improve the manuscript.

2.1 Comment: p.3, line 34: -> information on data

We have now edited the previous text with the following: “collect information on data acquisition” p. 3 line 32.

p.4, line 13: intra-

We have now implemented the reviewer’s suggestion p. 4 line 14.

p.4, line 16: Remove etc.

Changes have been made following the reviewer’s suggestion, p. 4 line 17.

2.2 Comment: p.5, lines 16-21: These sentences read strange and disconnected, perhaps primarily since it doesn't become clear how "necessary" is justified. I suggest rephrasing.

We now propose the following revision to better convey the importance of metadata and its role in facilitating data reuse and interpretation, p. 5 lines 11-19: “Moreover, data are often acquired at multiple time points, either for longitudinal assessments or due to time constraints among participants or to prevent volunteer fatigue [14]. Datasets gathered over several days are typically randomized or pseudo-randomized. This is particularly the case in some human projects where longitudinal studies are conducted [15]. Studies that involve repeated assessments typically span a long period of time (e.g., 10 years). In such studies, it is common that data are collected by different researchers, with different types of software, and new experiments may be added or removed. It is therefore essential to provide sufficient metadata as a set of documents available to download alongside the data itself. This would support data reuse and enable accurate analysis and interpretation.”

2.3 Comment: p.5, line 30: ...such as the Brain...

We corrected the typo following the reviewer’s suggestion, p. 5 line 22.

2.4 p.6, line 24: "more" than what?

We edited the sentence p. 6 lines 33-35: “Deep-learning neuroimage analysis tools require significant computing power, memory, and storage, and HPC clusters can provide these resources, dramatically accelerating the analysis process [34, 35].”

2.5 Comment: p.6. line 36: It is unclear, what "data decisions" refer to. The paragraph as such could be sharpened and clarified more.

Data decisions in the above-mentioned paragraph refer to the choices made regarding preprocessing methods and analysis techniques used on the datasets. We understand the reviewer's concern and have rephrased the sentence p. 6 lines 14-17: "Even if datasets are imported into a common file format, researchers' choices for data preprocessing and analysis may not be compatible between laboratories or even between different projects in the same laboratory. This is further complicated by the use of various resources, such as custom preprocessing workflows and softwares, which can vary widely."

2.6 Comment: p.7, line 21: Is a link to a dictionary useful here? Are there better definitions for scientific metadata?

We agree with the reviewer and now added the following references p.7 line 22:
– Stall S, Yarmey L, Cutcher-Gershenfeld J, Hanson B, Lehnert K, Nosek B, Parsons M, Robinson E, Wyborn L. Make scientific data FAIR. Nature. 2019 Jun;570(7759):27-29. doi: 10.1038/d41586-019-01720-7. PMID: 31164768.
– Stanford NJ, Scharm M, Dobson PD, Golebiewski M, Hucka M, Kothamachu VB, Nickerson D, Owen S, Pahle J, Wittig U, Waltemath D, Goble C, Mendes P, Snoep J. Data Management in Computational Systems Biology: Exploring Standards, Tools, Databases, and Packaging Best Practices. Methods Mol Biol. 2019;2049:285-314. doi: 10.1007/978-1-4939-9736-7_17. PMID: 31602618.

2.7 Comment: p.8, lines 5-13: It's unclear how these last sentences of the paragraph still relate to the topic of the section, "Metadata".

We concur with the reviewer's comment. We moved this paragraph to the section on challenges arising from diversity in acquisition, preprocessing, and analysis approaches p. 7 lines 8-19: "Reproducibility and variability in published results has been a topic of investigation [36, 37], and research has shown that there is no single "best" way to process and analyze large-scale single or multimodal datasets. For instance, a neuroimaging study presented the results of a survey of fMRI experiments that revealed substantial differences in how individual labs preprocess and analyze their data. 70 independent laboratories analyzed the same dataset and produced varying results [38]. Another study supported these findings and showed that analytical decisions made by individual researchers can significantly impact the findings from an fMRI data set [39]. Analyzing fMRI data with software packages such as SPM (Statistical Parametric Mapping) [40] or FMRIB Software Library (FSL) [41] can also lead to different outcomes. These findings emphasize the potential implications of the absence of standardized pipelines for handling complex data and how this can impact research outcomes. Efforts are being made to determine sources of variability and to develop homogenous and standardized computing environments [42]."

2.8 Comment: p.9, line 15ff: I suggest to refer to the resource https://eln-finder.ulb.tu-darmstadt.de

Thank you for your suggestion. We have incorporated this information p. 10 line 7: "Fortunately, resources such as the ELN Matrix created by the Harvard Biomedical Data Management Group and the ELN Finder, which provides information on various software options, can be incredibly useful in this process (https://zenodo.org/record/4723753 and https://eln-finder.ulb.tu-darmstadt.de/home, respectively)."

2.9 Comment: p.12, lines 2: Why would non-unified information be eliminated across conditions - wouldn't it be better to keep it?

We apologize for the lack of clarity. We now rephrased this paragraph p. 11 lines 8-14: "Another challenge that behavioral data present is reproducibility issues with regards to experimental results because it is often difficult to replicate the exact same conditions in which the experiment was conducted across the laboratories or even within the same laboratory [62].
It is indeed difficult to standardize metadata across behavioral experiments due to various factors that are difficult to control (confounding variables), such as laboratory environment (e.g., time of testing during light or dark phases, housing system for rodent experiments, auditory sounds), experimenter bias, which can lead to inconsistencies in data collection."

2.10 Comment: p.12, lines 3 and 5: It's unclear what is meant by algorithm here, please clarify.

We revised it into the following p.12 lines 15-20: “The lack of publicly available behavioral datasets with accurate annotations is a major impediment to benchmarking algorithms used in behavioral analysis [63]. These algorithms can range from simple statistical tests to more complex machine learning models that classify, cluster, or extract features from the behavior data [64, 65]. However, due to the complexity of behavior patterns, consistent labeling of data is challenging, and the time and resources required for collecting and labeling datasets make it expensive for many labs to obtain high-quality datasets for testing and comparing algorithms.”

2.11 Comment: p.12, lines 26-28: Why can important parameters be safely discarded in a derived dataset? Or do you want to say that they may get accidentally lost?

We thank the reviewer for the comment. We agree that the sentence does not reflect our point clearly and we rephrased it, p. 13 lines 4-13: “Differences in recording conditions, spike sorting algorithms and data preprocessing can lead to significant outcome variability. The real-time processing requirements for closed-loop experiments only serve to exacerbate these issues. Important parameters initially recorded from raw data, e.g., animal arousal/anesthesia level, impedance measurements, might be excluded or lost in derived datasets used for analysis, which can affect the accuracy of the results. Complex hierarchies of derived data and multimodal datasets (e.g., accelerometer, whisker or pupil tracking, etc.) collected with different instruments compound these issues. It can be challenging to validate and reproduce results, because the use of various algorithms and parameters used can produce different outcomes. Therefore, it is essential to carefully consider and account for all relevant parameters and sources of variability in the analysis of complex datasets.”

2.12 Comment: p.13, line 27: an RDM strategy

We corrected the typo throughout the manuscript.

2.13 Comment: p.19, line 17: Why are data from acquisition not directly transferred, but only after a few days? Is this important?

We thank the reviewer for the comment. We now clarified the storage procedure p. 20 lines 15-24: “Datasets collected from confocal or two-photon microscopes are stored on acquisition computers temporarily (a few days) allowing researchers to quickly access, check and transfer the data to a secure data storage platform on SDS@hd for further preprocessing and analysis.
Once the datasets are fully copied and backed up, individual users must ensure that the large datasets are timely (usually in a few days or a week depending on data volume) removed from the acquisition computers to free up the acquisition storage system for new data or for another user. The reason to remove data from acquisition computers is that such computers are often not designed for long-term storage and can be vulnerable to hardware failures, data corruption, or security breaches. To mitigate these risks, it is a common practice to copy the data immediately to the data storage platform to ensure long-term data preservation, security, and accessibility. “

2.14 Comment: p.19, line 23: the next

We proofread and fixed grammatical errors throughout the manuscript.

2.15 Comment: p.21, lines 21-32: This paragraph doesn't seem to fit so well in the data processing section. Maybe a better fit in the section before (storage solutions)?

We thank the reviewer for the suggestion. The above-mentioned paragraph deals with storage, analysis and visualization. We therefore believe that it should remain in the section: “Data processing, analysis, and visualization”, p. 21, lines 16-38. The paragraph specifically discusses the way in which data are processed, including the platforms used for analysis such as HPC clusters. Having direct data access from SDS@hd to HPC clusters shows interoperability and is therefore directly relevant to the data processing section, which focuses on the ways in which data is handled and manipulated.

2.16 Comment: p.22, lines 25-16: Fix link formatting

We have now checked all the links in the entire manuscript. E.g., p. 23, lines 16-19: “The Competence Centre for Research Data (Kompetenzzentrum Forschungsdaten, KFD), a joint institution run by the Library and Computing Centre at the University of Heidelberg, established a fully encrypted, web-based elabFTW instance (https://www.urz.uni-heidelberg.de/en/service-catalogue/software-and-applications/elabftw) with secure cloud-based data storage.”

2.17 Comment: p.23, line 8: I am confused about the numerous protocols. If two of them are "anatomical" and "functional", what are all the others? Aren't those a more high-level classification for protocols?

We thank the reviewer for the comment. Other MR acquisitions can include magnetic resonance spectroscopy (measure of neurochemistry) or arterial spin labeling (measure of perfusion). Such acquisitions could indeed also be classified as structural and functional. We therefore rephrased as follows p. 27, lines 14-16: “For instance, to standardize MRI data acquisitions, we developed MR acquisition protocols for anatomical (e.g., T1-weighted images) and functional scans (e.g., echo planar imaging, EPI) in terms of image resolution, type of acquisition and duration.”

2.18 Comment: p.23, lines 10-13: The sentence "By organizing..." is not clear -- why would you ever have to input metadata somewhere in order to access it?

We thank the reviewer for the comment. We rephrased as follows p. 21 lines 37-38, p. 28 lines 1-8: “For example, a typical MR brain acquisition includes anatomical scans (e.g., T1-weighted images) and functional scans (e.g., echo planar imaging) and have predefined directory labels in BIDS nomenclature, resp. “anat” and “func”. In BIDS, metadata fields common across all subjects are specified in a single JavaScript Object Notation (JSON) file in the root directory instead of multiple files repeated for each subject. Organizing the data according to the BIDS standards ensures that the metadata is automatically included in the metadata file, eliminating the need for manual input and saving time and effort in processing large amounts of data. Moreover, adoption of BIDS enabled the development of workflows for automated data extraction, curation and labeling. For example, automatic extraction of a minimal set of BIDS compatible metadata can be performed using dcm2niix (https://www.nitrc.org/projects/dcm2nii/).”

2.19 Comment: p.24, line 1: Sentence broken -- its unclear what the catalyst link refers to. A company?

We have now rephrased the sentence p. 24 lines 35-38, p. 25 lines 1-3: “To ensure comprehensive metadata documentation and standardization of neurophysiology datasets collected across rodent projects of the consortium, we collaborated with Catalyst Neuro, a neuroscience software solutions company (https://www.catalystneuro.com/), to design a web-based metadata standardization GUI. The source code and installation guide are available for use at https://github.com/catalystneuro/heidelberg-metadata-gui. The metadata handling GUI allows standardized documentation of metadata (experimental information, acquisition and analytical parameters etc.) collected from neurophysiology experiments [90].”

2.20 Comment: p.24, lines 18-21: For the reader, the most interesting question may be: Why design a GUI first rather than use elabftw to design metadata templates for the experiments? I can imaging multiple reasons, but what was the reason here that this was not a good approach?

We agree that there are multiple reasons for designing a GUI as compared to using elabFTW. We added the following information in the data and metadata documentation and standardization section p. 23 lines 31-37, p. 24 lines 1-28: “In our RDM framework, we use ELNs as a platform to document experimental protocols alongside minimal metadata generated automatically during an experiment. Most commonly, we use elabFTW, which accepts JSON files. elabFTW acts as a "notebook," tracking both primary data (experiment findings, measurements, etc.) and metadata (date, time, author, units, used inventory, etc.). The experimental metadata (e.g., microscope specifications, data acquisition settings) is stored in a standardized manner using a generic metadata file format that is compatible with open file formats, such as JSON or XML.
However, ELNs are not usually designed as full-fledged metadata systems and may lack domain-specific

functionalities, such as support for file formats specific to neuroscience. Integrating metadata generated during different stages of a neuroscience research project (such as experimental design, data acquisition and preprocessing, statistical analysis, visualization, and dissemination) into ELN presents a significant challenge. Most ELNs do not offer sufficient support for the diverse data or metadata file formats generated during these intermediate steps, which makes incorporating additional metadata challenging without an automated tool or API, which can be time consuming.
In addition to the limitations of ELNs, many neuroscience datasets (e.g., electrophysiology experiments such as single-unit recordings or local field potential (LFP) recordings) lack consistent metadata schema even at the most basic level. This means there is no standardized structure or format and terminologies for storing metadata such as experimental parameters (e.g., stimulus type, duration, intensity, and location), animal or subject information (e.g., species, age, sex, and weight), recording equipment and settings (e.g., amplifier type, sampling rate, and filtering), data processing parameters and analysis methods. To add, search, filter, or use various types of metadata effectively, specialized tools are often necessary. These tools are designed to handle the complexity and diversity of metadata formats, and to streamline the process of metadata management and integration with data analysis workflows. Without such tools, it can be challenging and time-consuming to work with large, complex datasets that contain multiple types of metadata.
For instance, when converting complete datasets, such as raw, pre-processed, and analyzed files from an electrophysiology experiment, into a standardized open data format, the resulting basic metadata file is frequently insufficient and lacks important information such as analysis parameters, spike sorting, or filtering parameters.
Therefore, we face the challenge of integrating comprehensive metadata, including experimental, acquisition, and analytical metadata, into a single file with a format compatible with open data standards.
To address these challenges of inconsistent metadata schemas, lack of tools for metadata management, and insufficient support for domain-specific file formats in ELNs, we developed custom tools (explained in the next section), which enable to standardize experiments or data modality-specific standardization and to automate the data documentation process. "
We have also added the following information in the data standardization in the rodent projects section p. 25, lines 15-19: "While eLabFTW is useful for recording experimental protocols, the GUI provides extra features such as automatic data validation and support for specific metadata formats. It can be easily customized to fit project requirements, facilitating more flexible and standardized metadata management across different platforms and tools. Overall, the GUI improves the efficiency and reliability of workflows within the lab and enables submission of complete datasets for archiving and future use."

2.21 Comment: p.28, lines 12-13: Sentence seems broken

We now restructured the sentence p. 30, lines 7-10: "The central strategy of the consortium involves coordinated cross-species analyses in experimental animal models and in human subjects. To achieve this, we use multi-scale imaging, electrophysiological, psychometric, and behavioral readouts, as well as a range of interventional strategies across both rodent and human populations."

2.22 Comment: p.29, line 34:) missing

The typo is now corrected.

2.23 Comment: p.30, line 10: As pointed out in the first round of reviews, NFDI-Neuro sadly is not funded.

While NFDI-Neuro is not currently funded, we had the opportunity to collaborate with them on a project where we developed a folder structure. We wanted to acknowledge the previous collaboration and the contributions made by NFDI-Neuro members, while also emphasizing the work done independently by us. We edited the sentence as follows p. 33 lines 13-17: For this purpose, we use folder structure templates for research repositories developed in collaboration with the NFDI Neuroscience (NFDI-Neuro) consortium (currently non-funded) and three neuroscience CRCs (CRC 1158, CRC 1315, and CRC/TRR 135), [122]. The template structure is available on Zenodo and can be downloaded here:
https://doi.org/10.5281/zenodo.4410128 [123].

2.24 Comment: p.30, lines 14-: I was surprised to see this in the discussion. I would think that the folder

structure should be explained. In particular, it's not really apparent without and explanation how this folder structure can be made compatible with the BIDS folder structure, given its division in part 03 and 04 as explained on lines 18-19. Also, it's unclear to me how this folder structure will practically work for data worked on by multiple people (i.e., same data folder, but multiple analysis projects).

We appreciate the reviewer's interest in the folder structure templates. We would like to inform the reviewer that a detailed description of the folder structure, along with a step-by-step instruction guide, is the subject of another manuscript currently in preparation. We however added some information to answer to the reviewer’s comments, p. 33 lines 8-31: “We recommended the CRC 1158 project members to utilize logical file and folder templates to support systematic data organization. Consistent folder organization depends on the type of research data acquired for a project as well as on the governance procedures. Our goal is to provide researchers with an easy way to manage their project digital files and datasets on different data infrastructure services, both locally and on subject-specific data repositories such as GIN: a Modern Research Data Management System for Neuroscience. For this purpose, we use folder structure templates for research repositories developed in collaboration with the NFDI Neuroscience (NFDI-Neuro) consortium (currently non-funded) and three neuroscience CRCs (CRC 1158, CRC 1315, and CRC/TRR 135), [122]. The template structure is available on Zenodo and can be downloaded here: https://doi.org/10.5281/zenodo.4410128 [123].
These templates are designed to reflect the typical workflow of a research project. This means that the structure is organized in a way that makes it easy to track the different stages of data acquisition, processing and analysis. Depending on the specific needs of the CRC projects, we customize the templates based on the type of experiment or data modality as well as the analysis processes that should be integrated with existing data organization systems. The template structure includes separate sections for raw data and analyzed data, as well as for documentation and code related to each stage of the workflow. To illustrate, neuroimaging datasets such as MEG or fMRI that are in the BIDS format can be efficiently organized and stored in the "03_data" directory. The raw data (BIDS raw, e.g., NIfTI and JSON) can be stored in the subfolder "001_defaultexp" (default experimental data), and the analyzed data (BIDS derivatives, e.g., fMRIPrep, SPM, FSL, FreeSurfer, QSIprep, etc.) can be stored in the subfolder "999_processed_data". It is recommended to add workflows and code libraries used for data analysis to the designated analysis directory i.e., "04_data_analysis". These folder structure templates facilitate reproducibility, data sharing, and can be utilized on different storage devices to accommodate various data sets generated during experiments, independent of their format.”

2.25 Comment: p.30, line 20: What does it mean that the templates are "workflow-based"?

We kindly refer the reviewer to comment 2.23. We now clarified the previous sentence p. 33, lines 18-20: “These templates are designed to reflect the typical workflow of a research project. This means that the structure is organized in a way that makes it easy to track the different stages of data acquisition, processing and analysis.”

2.26 Comment: p.34, lines 7-12: This sentence is still highly broken as in the initial version of the manuscript. Here is a suggestion for a correct sentence:

We edited the previous sentence following the reviewer’s suggestion p. 37, lines 15-22: This includes data formats (e.g., Neuroscience Information Exchange (NIX; http://g-node.github.io/nix/), [142, 143], Neurodata Without Borders (NWB; https://www.nwb.org), [92, 144], data versioning tools (e.g., DataLad, a US-German collaboration for computational neuroscience project (https://www.datalad.org/) [145], GIN (https://gin.g-node.org), metadata collection tools (e.g., CEDAR (https://metadatacenter.org), NIDM (http://nidm.nidash.org), open metadata markup language (odML), [45, 146], data representation models (Neo [147]), data analysis tools (e.g., Electrophysiology Analysis Toolkit (Elephant; https://python-elephant.org), FieldTrip (https://www.fieldtriptoolbox.org/), [148], simulation interfaces (e.g., PyNN [149]), etc.

2.27 Comment: p.34, lines 7-12: A better link is: https://www.incf.org/sig/incf-working-group-standardized-data

We added the link suggested by the reviewer p. 37 lines 13-14.

2.28 Comment: p.36: Remove : after headings

We now removed the ":" after all headings throughout the manuscript.

2.29 Comment: p.37, lines 2-3: Sentence broken

Thank you for this comment. We now corrected the sentence as follows, p. 38 lines 36-37, p. 39 lines 1-3: "For instance, since most CRC projects run for multiple funding periods and involve extended analyses, data versioning becomes even more crucial. Researchers often modify, refine or add to their datasets during the course of their research. Without proper versioning, it may not be possible to reproduce previous findings, which can negatively impact the credibility of the research outcomes."

2.30 Comment: p.38, lines 8-22: The paragraph is contradicting in that first datalad is advertised as a data sharing platform, then its denied. In fact, to my knowledge, DataLad is a tool for management, not a service.

We thank the reviewer for pointing out the apparent contradiction in the paragraph. We agree with the reviewer that DataLad is not a data sharing platform, but rather a tool for managing distributed datasets. We now restructured the above paragraph p. 39, lines 8-19: "Platforms such as DataLad (https://www.datalad.org/) and GIN (https://gin.g-node.org/) may effectively compensate for a lack of local resources. Data hosting and sharing can also ensure data versioning and encourage reproducible management of scientific data. Both DataLad and GIN are based on git and git-annex to provide a decentralized system for the exchange of large datasets. Datalad is an open-source software package for the management of distributed datasets. It facilitates the acquisition, organization, and management of data stored in remote repositories, but does not offer storage. Moreover, the GIN service can be deployed locally at all participating labs and can be used as an in-house storage server and web user interface for DataLad datasets. Datasets hosted on either platform can be accessed via git-compatible systems. Other examples of resources for supporting collaborative workflow development and integration of data hosting and processing/analysis computing resources include the Open Science Framework (https://osf.io/) and the Open Science Grid (https://opensciencegrid.org/)."

2.31 Comment: p.39, lines 14-19: The sentences were unclear to me. Please clarify.

Thank you for the comment, we rephrased the sentence p. 41 lines 16-30: "Researchers often encounter challenges in determining the best practices for archiving and gaining access to archival systems. Researchers require assistance and support to ensure that their research data are appropriately archived and accessible to other researchers. Firstly, researchers need guidance on best practices for archiving their research data, including how to stay informed about the storage, retention, and disposal of all research data. This is especially crucial as good archival practice requires a scheduled review of data in long-term storage. Therefore, researchers need to remain informed about these practices, whether their data are stored in an institutional or external repository. In addition, researchers need support to ensure that their data handling complies with various regulations and guidelines. This includes existing discipline-specific privacy and ethical standards, copyright or licensing arrangements, and publication and legal requirements. Meeting these regulations and guidelines is crucial to ensure that researchers' data are protected and accessible to other researchers. To address these challenges, research institutions and organizations can provide researchers with the necessary training, resources, and infrastructure for archiving their data. This includes developing guidelines and policies on data management and sharing, providing access to data repositories and storage facilities, and offering training and support on data management and archiving best practices."

Close
