# Supplementary material for: Data management strategy for a collaborative research center
Source: Gigascience. 2023 Jul 4;12:giad049. doi: 10.1093/gigascience/giad049 (PMC10318494; doi:10.1093/gigascience/giad049)
Supplement: giad049_Reviewer_1_Report_Original_Submission — Amanda Charbonneau -- 11/15/2022 Reviewed [file giad049_reviewer_1_report_original_submission.pdf]

Reviewer Report

Title: Data management strategy for a Collaborative Research Centre

Version: Original Submission **Date:** 11/15/2022

Reviewer name: Amanda Charbonneau

Reviewer Comments to Author:

The authors describe a multistage plan that they implemented to improve data standards and management for the Heidelberg Pain Consortium. Having done similar work, I can tell that a great deal of thought and work went into creating this management program, however the paper will need substantial refactoring for that effort to shine through. The pieces of a great paper are already here, they are just difficult to follow.

Major comments:

pg 3, lines 31-33 "Implementing effective data management strategies and ethical rules for the reuse and sharing of high-quality data reduces redundant research, optimizes public research funding, and reduces animal use." This seems like a significant claim supporting the need for RDMs, but has no citation. Is there evidence that RDMs reduce animal use or reduce redundant research? What does optimized public research funding look like? This statement is surrounded by several other assertions with little explanation, e.g.:

"A clear set of regulations and guidelines must be established before sharing human data gathered from clinical or non-clinical populations. Specific rules addressing privacy issues, established processes for data protection, data use and reuse, and the preservation of sensitive data are required. It is essential to make data accessible and understandable to remote (or future) collaborators in order to maximize the potential of existing algorithms and tools and accelerate the creation of new ones."

I would like to see the introduction more throughly discuss the problems with data management and to elaborate on these asserted solutions.

There are multiple other places throughout the text where there are ideas stated as facts without references or elaboration, that I would like to see evidence for, or at least a discussion of. For e.g. pg 13, lines 1-2 and lines 26 - 29; pg 15, lines 13 - 19; pg 16, lines 4-5;

pg 4, lines 2-5 "While the majority of collaborative research consortiums collect a wide variety of multidimensional datasets, the majority of these datasets are typically inadequate for modern research methods and infrastructure." As above, I'm interested how this is quantified, or if there is a reference, and to have a more complete discussion of this idea.

Beginning on line 27 of page 4, the introduction veers into describing many technical details of specific studies. In my first read-through, I actually thought that I had accidentally flipped to a different paper. None of these details seem relevant to the larger concept of creating an effective RDM strategy, and are very disorienting. I would remove them entirely.

Figure 1. In order to understand how the authors have implemented an RDM plan, it seems important for the reader to understand the basic structure of the consortium, however, I do not understand how to interpret figure 1. It appears that the animal projects somehow encapsulate all the others? Are the

relative sizes of the colored portions meaningful? The number of tandem projects is half that of animal, which seems to agree with the peach width being smaller than the green. However there are only 4 human projects for the very large yellow section, and 1 for the black. I would drop this figure entirely. I think Figure 2 does a much better job of describing the consortia, and is much easier to read. Further, pg 8, lines 5 - 12 are a near copy of the legend for Figure 1, but refer to Figure 2 A anyway.

Beginning on pg 6, line 35 to pg 7 line 16; and then again beginning on pg 10, line 34 to pg 18, line 16, this entire text is general background of problems in data management, and specifically around the particular challenges of this consortia. It is altogether a much better introduction than the existing introduction. It covers all of the missing context, as well as discussing the types of studies the Heidelberg Pain Consortium needs to work with without delving into over technical details. I recommend excising this from the results and using it to replace everything after the second paragraph of the introduction. With minor adjustments for grammar, it will be much better there. The move will also make your actual results, which currently don't begin until pg 18, line 18, much easier to find and follow.

In the results (beginning pg 18, line 18), there are several sections where important discussion points are interspersed with the results, making it difficult to tell which are the work of the consortium, and which are simply informational. It would greatly improve readability if these paragraphs were pulled out and integrated into a discussion section, which doesn't currently exist. As I'm finding it difficult to tell which parts are your work, below is my best guess at the paragraphs that should be moved, or dropped:

pg 19, lines 27 - 32
pg 20, lines 1 - 12
pg 21 line 1 - pg 22 line 18
pg 22 lines 19 - 29 (excluding the title, which should be moved to line 30)
pg 22 line 34 - pg 23 line 5
pg 23 lines 18 - 28
pg 26 line 10 - pg 27 line 6
pg 27 line 31 - pg 28 line 1 (excluding the title, which should be moved to line 2)
pg 30 line 33 - pg 32 line 21

For the "Recommended data standards for different data types", "Animal neuroimaging data standardization", and "Data processing, analysis, and visualization" sections, the results and discussion pieces are not as easily pulled out as paragraphs, so I have not listed each individual sentence here. Instead, I would suggest looking at these sections with an eye towards picking out which are results of something your consortium worked on, and which are expounding on ideas or comparing it to other work.

Clearly moving all of those to the discussion will require some editing to make it make sense, but I think that if you make the discussion follow the same minor heading structure you have in the results it will be a relatively painless change.

The first part of the current results: pg 5, line 23 - pg 6 line 34 would be very good if moved to be the first few paragraphs of the discussion, but are too vague to be part of the results.

pg 6, line 37 - pg 7 line 5. "We specifically describe our ongoing RDM efforts, which are divided into two sections: 1) RDM Planning Phase: Identifying common RDM procedures across consortium projects; Evaluating common data management challenges; Special RDM challenges in specific projects; 2) RDM Implementation Phase: resource allocation decisions and implementation of key resources and 3) Data

Dissemination (data management continues even after the end of the funding period)." A minor typo is that it should say three sections, not two. Rather more importantly, both here and in the conclusion, it lists three phases, but phase three does not appear in the actual text. It is not described along with the others in the conclusion, or mentioned at all in the results.
Minor comments:
SBF is never defined, and the authors seem to use SFB1158, SFB and SFB 1158 as synonyms, but I'm not sure if that is the intent.
I would move all of the funding information on pg 4 to the funding section, it's distracting here
There are several minor grammar issues. I am not copying them all here, as I'm sure they'll be fixed in the rearrangement, but:
pg 3 line 26 should be 'cooperative'
pg 6, line 7; its unclear what "progressively practical RDM approaches" means
pg 7, line 1 should be 'three'
pg 7, line 21 'can' should be 'could'
pg 11, lines 23-26, this sentence doesn't seem to finish "In cases where a laboratory produces a large dataset from a single experiment, and the collected metadata are too complex and stored in multiple files having different formats that are only read by the acquisition software or by customized codes written for internal use."
pg 12, lines 17 - 12, this sentence/paragraph seems to be missing words/doesn't finish "Although many advances have been made regarding organization, annotation, and description of research datasets, for example using the Brain Imaging Data Structure (BIDS, https://bids.neuroimaging.io/) for neuroimaging data standards [10] EEG-BIDS for neurophysiology data (e.g., electroencephalography), [11], or MEG-BIDS for magnetoencephalography data [12], whereas other data modalities (e.g., EMA) do not have existing standards yet.
pg 15, lines 4 - 7, this sentence seems to me missing words: "However, while choosing an electronic laboratory notebook for a large-scale neuroscience consortium spanning diverse experimental protocols, the availability of clear documentation and application-centric features become an overarching issue."

Methods

Are the methods appropriate to the aims of the study, are they well described, and are necessary controls included? Choose an item.

Conclusions

Are the conclusions adequately supported by the data shown? Choose an item.

Reporting Standards

Does the manuscript adhere to the journal's guidelines on [minimum standards of reporting?] Choose an item.

Choose an item.

Statistics

Are you able to assess all statistics in the manuscript, including the appropriateness of statistical tests used? Choose an item.

Quality of Written English

Please indicate the quality of language in the manuscript: Choose an item.

Declaration of Competing Interests

Please complete a declaration of competing interests, considering the following questions:

- Have you in the past five years received reimbursements, fees, funding, or salary from an organisation that may in any way gain or lose financially from the publication of this manuscript, either now or in the future?
- Do you hold any stocks or shares in an organisation that may in any way gain or lose financially from the publication of this manuscript, either now or in the future?
- Do you hold or are you currently applying for any patents relating to the content of the manuscript?
- Have you received reimbursements, fees, funding, or salary from an organization that holds or has applied for patents relating to the content of the manuscript?
- Do you have any other financial competing interests?
- Do you have any non-financial competing interests in relation to this paper?

If you can answer no to all of the above, write 'I declare that I have no competing interests' below. If your reply is yes to any, please give details below.

I declare that I have no competing interests

I agree to the open peer review policy of the journal. I understand that my name will be included on my report to the authors and, if the manuscript is accepted for publication, my named report including any attachments I upload will be posted on the website along with the authors' responses. I agree for my report to be made available under an Open Access Creative Commons CC-BY license (http://creativecommons.org/licenses/by/4.0/). I understand that any comments which I do not wish to be included in my named report can be included as confidential comments to the editors, which will not be published.

Choose an item.

To further support our reviewers, we have joined with Publons, where you can gain additional credit to further highlight your hard work (see: https://publons.com/journal/530/gigascience). On publication of this paper, your review will be automatically added to Publons, you can then choose whether or not to claim your Publons credit. I understand this statement.

Yes Choose an item.
