# Supplementary material for: Data management strategy for a collaborative research center
Source: Gigascience. 2023 Jul 4;12:giad049. doi: 10.1093/gigascience/giad049 (PMC10318494; doi:10.1093/gigascience/giad049)
Supplement: giad049_Reviewer_2_Report_Original_Submission — Michael Denker -- 11/18/2022 Reviewed [file giad049_reviewer_2_report_original_submission.pdf]

Reviewer Report

Title: Data management strategy for a Collaborative Research Centre

Version: Original Submission **Date:** 11/18/2022

Reviewer name: Michael Denker

Reviewer Comments to Author:

The authors of the manuscript "Data management strategy for a Collaborative Research Centre" take the reader on a timely tour de force through the various aspects surrounding the establishment of RDM measures in a large, collaborative research project spanning multiple highly heterogeneous subprojects and partners. I found the topic of the paper highly interesting and engaging, as it is based on the experiences and procedures encountered in a real-world scenario. The challenges and solutions are presented in a clear, well-written and structured way in two overarching chapters, respectively, that make the article enjoyable to read. Also, I found the breadth of challenges well captured.
While I am enthusiastic to see the manuscript published, I have several points and suggestions I would like to put up for consideration.
As a general point, I found the article quite lengthy due to a lot of detail that was put into the description of the individual challenges. At the same time, I found many challenges described in the planning phase to be rather high-level statements, and I was often hoping to have these more embedded into the actual problems encountered in the SFB to give illustrative examples. I would like suggest to the authors to reconsider if they find specific examples of some of the more abstract challenges that were actually encountered (similar, e.g., to the last part in "planning" section about specific RDM challenges). The reasoning here is that I find the strong point of this article that it comes from actual experience, and I found this not ideally reflected in some of the more abstract descriptions. An example illustrating this suggestion is given in the detailed points below (p10, l31-32). To compensate, perhaps some of the details in the manuscript could be shorted.
Also, as a general point I found that many of the existing tools/services/... were not referenced (by either publication, DOI, RRDI, https link or at least some pointer to the resource).
- p5, Figure 1: I did not find this illustration very helpful, I think a more standard 2D representation with the individual groups and links between groups forming tandems would be better understandable.
- p6, l13: I think it would be extremely useful for other consortia to know more about the day-to-day tasks of the data manager, in particular in terms of the job profile -- what background is more useful: neuroscience, computer science, software engineer, project planning/organization? As the job description of a "data manager" is evolving, it would be great to hear what tasks made this SFB successful in cross-group RDM.
- p6, l23: What level of granularity was put into the guideline in terms of "data formatting"? As outlined in later sections, the data is highly heterogeneous.
- p8, l2: How was the compliance to the survey? Given the complexity of RDM, did researchers need prior education wrt the meaning of RDM related questions of the survey, and were measures put in place to improve the quality of answers?

- p8, Figure 2C: I found panel 2C not immediately clear in terms of the organization and identifying the row/column structure. A slight improvement visually and/or 1-2 sentences of description of the panel in the caption would help.
- p10, l31-32: This is one concrete example of the general remark above: "manageability issues" are very abstract, an example of what types of problems precisely triggered this statement would be very illuminating and supportive of the statement.
- p11, l2-: The same as the previous comment holds for the challenges associated with multi-modal data, as its unclear on which level the integration step happens. Moreover, I would welcome a more elaborate description of the level of integration of data in a tandem project: how far could researchers go in integration? Did researchers go as far as analyzing data from their tandem partner and vice versa? To which degree could datasets be actually integrated/compared in practice?
- p14, l15-: Did these reproducibility issues occur in practice?
- p15, l1-: One could also mention potential legal/data privacy concerns related to selecting an electronic lab notebook.
- p17, l24: Please reformulate or define "neural assemblies", as this may be ambiguous.
- p17, l15-: I really like this last subsection as it is very concrete and down-to-earth. However, for this paragraph in the section, I found it unclear where exactly the special challenge lies, i.e., what aspects are truly unique to this situation. Are similar problems not equally present, e.g., when looking at the neuropixel probes (p17, l1-) where different spike sorters will produce different results, and potentially similar computationally demanding workflows are required?
- p20, l1-: What distinguishes the three templates? Are DMPs considered living documents that accompany the project duration, and if so, how are they followed up?
- p20, l21-: I found the listing of storage locations somewhat confusing. I would suggest to list first, the available options, underlying technology (e.g., nextcloud, ssh,...) and their properties (e.g., sharing possibilities, availability on HPC, backup, versioning, access,..) and, second, how these factors influenced the choice for labs in the consortium. Which features where actually decisive?
- p21, l7: NFDI-Neuro is currently a non-funded consortium.
- p21, l11: GIN is not linked at its first occurrence.
- p21, l15: How do the folder structure templates relate to standards such as BIDS?
- p21, l24: Similar as above, it would be of interest to better understand the technological foundation and usage scenario of HeiBox.
- p22, l7: I am not certain datalad is properly represented by this description. Does datalad itself offer storage? While I am not expert, I had seen datalad more as an abstraction layer to handle versioned datasets and workflow execution, largely focused on git-based versioning.
- p22: How were storage locations suggested to consortium members? What role did HPC play in this process?
- p23, l2-4: I think https://eln-finder.ulb.tu-darmstadt.de/home is a more recent and complementary resource to the Harvard spreadsheet, that should be also mentioned here.
- p23, l6-: How was the adoption of a common ELN received in labs? How difficult was the integration into lab workflows (especially for complex experiments) in terms of automation, such that ELNs were not perceived as additional burden?
- p24, l2-4: I did not understand this sentence. Which structure and function in which populations?

- p24, l15: I found the purpose of the volunteer platform somewhat unclear. Which problem concretely does it solve, i.e., which type of redundant data acquisition does it help to avoid?
- p24, l26-27: It was not clear to me how BIDS reduces manual metadata input.
- p25, l4: Typo: licenses->license
- p25, l28-32: Typo: Sentence seems broken in the listing of tools.
- p26, l10-: This paragraph left me very confused. If ELNs are adopted (which have a primary function of enabling users to fill in metadata records via a configurable GUI) why then are metadata entered first in a custom GUI that then produces a JSON that is then fed into the ELN? Would it not be more straight forward to enter metadata in ELN-defined schemas, then export these to JSON (in case they are required outside the ELN)? Also, why is this procedure ephys-specific?
- p26, l10-: Which types of metadata are we talking about -- generic or experiment specific?
- p27, l15: I would argue that while TIFF is a complex/potent file format, it's not really propitiatory. Also, the number of image file formats is not particular large. Therefore, I would argue, the situation is somewhat different from neurophysiology.
- p29, l15-: In this paragraph, it was unclear to me if the data management efforts actually led to an increased user base of the HPC machines -- or if essentially those groups used the HPC resources who know how to use them? In other words, how much training was necessary to promote use of HPC machines?

Methods

Are the methods appropriate to the aims of the study, are they well described, and are necessary controls included? Choose an item.

Conclusions

Are the conclusions adequately supported by the data shown? Choose an item.

Reporting Standards

Does the manuscript adhere to the journal's guidelines on minimum standards of reporting? Choose an item.

Choose an item.

Statistics

Are you able to assess all statistics in the manuscript, including the appropriateness of statistical tests used? Choose an item.

Quality of Written English

Please indicate the quality of language in the manuscript: Choose an item.

Declaration of Competing Interests

Please complete a declaration of competing interests, considering the following questions:

- Have you in the past five years received reimbursements, fees, funding, or salary from an organisation that may in any way gain or lose financially from the publication of this manuscript, either now or in the future?
- Do you hold any stocks or shares in an organisation that may in any way gain or lose financially from the publication of this manuscript, either now or in the future?
- Do you hold or are you currently applying for any patents relating to the content of the manuscript?
- Have you received reimbursements, fees, funding, or salary from an organization that holds or has applied for patents relating to the content of the manuscript?
- Do you have any other financial competing interests?
- Do you have any non-financial competing interests in relation to this paper?

If you can answer no to all of the above, write 'I declare that I have no competing interests' below. If your reply is yes to any, please give details below.

I declare that I have no competing interests.

I agree to the open peer review policy of the journal. I understand that my name will be included on my report to the authors and, if the manuscript is accepted for publication, my named report including any attachments I upload will be posted on the website along with the authors' responses. I agree for my report to be made available under an Open Access Creative Commons CC-BY license (http://creativecommons.org/licenses/by/4.0/). I understand that any comments which I do not wish to be included in my named report can be included as confidential comments to the editors, which will not be published.

Choose an item.

To further support our reviewers, we have joined with Publons, where you can gain additional credit to further highlight your hard work (see: https://publons.com/journal/530/gigascience). On publication of this paper, your review will be automatically added to Publons, you can then choose whether or not to claim your Publons credit. I understand this statement.

Yes Choose an item.
