# Supplementary material for: Data management strategy for a collaborative research center
Source: Gigascience. 2023 Jul 4;12:giad049. doi: 10.1093/gigascience/giad049 (PMC10318494; doi:10.1093/gigascience/giad049)
Supplement: giad049_Reviewer_2_Report_Revision_1 — Michael Denker -- 3/9/2023 Reviewed [file giad049_reviewer_2_report_revision_1.pdf]

Reviewer Report

Title: Data management strategy for a Collaborative Research Centre

Version: Revision 1 **Date:** 3/9/2023

Reviewer name: Michael Denker

Reviewer Comments to Author:

The authors have performed a detailed reworking of the manuscript, answering to most of my suggestions and concerns. I still find that, perhaps due to the length of the text, there are a number of issues left to fix.
Primarily, I urge the authors to very carefully and diligently go through the text again as there seem to be a number of inconsistencies, typos, missing links to tools... left to fix, many of which I probably did not spot. For example regarding inconsistencies, sometimes tools are mentioned briefly in one place, and only later when they appear again in the text the tools are introduced in more detail and with a link. Also, I had the feeling that there would be some space for streamlining the text a bit, maybe cutting on some of the more obvious and less CRC-specific aspects of data management. I would advise to have another fellow reader go through the text to best spot potential redundancies.
p.3, line 34: -> information on data
p.4, line 13: intra-
p.4, line 16: Remove etc.
p.5, lines 16-21: These sentences read strange and disconnected, perhaps primarily since it doesn't become clear how "necessary" is justified. I suggest rephrasing.
p.5, line 30: ...such as the Brain...
p.6, line 24: "more" than what?
p.6. line 36: It is unclear, what "data decisions" refer to. The paragraph as such could be sharpened and clarified more.
p.7, line 21: Is a link to a dictionary useful here? Are there better definitions for scientific metadata?
p.8, lines 5-13: It's unclear how these last sentences of the paragraph still relate to the topic of the section, "Metadata".
p.9, line 15ff: I suggest to refer to the resource https://eln-finder.ulb.tu-darmstadt.de
p.12, lines 2: Why would non-unified information be eliminated across conditions - wouldn't it be better to keep it?
p.12, lines 3 and 5: It's unclear what is meant by algorithm here, please clarify.
p.12, lines 26-28: Why can important parameters be safely discarded in a derived dataset? Or do you want to say that they may get accidentally lost?
p.13, line 27: an RDM strategy
p.19, line 17: Why are data from acquisition not directly transferred, but only after a few days? Is this important?
p.19, line 23: the next
p.21, lines 21-32: This paragraph doesn't seem to fit so well in the data processing section. Maybe a

better fit in the section before (storage solutions)?
p.22, lines 25-16: Fix link formatting
p.23, line 8: I am confused about the numerous protocols. If two of them are "anatomical" and "functional", what are all the others? Aren't those a more high-level classification for protocols?
p.23, lines 10-13: The sentence "By organizing..." is not clear -- why would you ever have to input metadata somewhere in order to to access it?
p.24, line 1: Sentence broken -- its unclear what the catalyst link refers to. A company?
p.24, lines 18-21: For the reader, the most interesting question may be: Why design a GUI first rather than use elabftw to design metadata templates for the experiments? I can imaging multiple reasons, but what was the reason here that this was not a good approach?
p.28, lines 12-13: Sentence seems broken
p.29, line 34:) missing
p.30, line 10: As pointed out in the first round of reviews, NFDI-Neuro sadly is not funded.
p.30, lines 14-: I was surprised to see this in the discussion. I would think that the folder structure should be explained. In particular, it's not really apparent without and explanation how this folder structure can be made compatible with the BIDS folder structure, given its division in part 03 and 04 as explained on lines 18-19. Also, it's unclear to me how this folder structure will practically work for data worked on by multiple people (i.e., same data folder, but multiple analysis projects).
p.30, line 20: What does it mean that the templates are "workflow-based"?
p.34, lines 7-12: This sentence is still highly broken as in the initial version of the manuscript. Here is a suggestion for a correct sentence:
"This includes data formats (e.g., Neuroscience Information Exchange (NIX; http://g-node.github.io/nix/) [X, 139], Neurodata Without Borders (NWB; https://www.nwb.org) [93, 140]), data versioning tools (e.g., DataLad (https://www.datalad.org/) [141], GIN (https://gin.g-node.org)), metadata collection tools (e.g., CEDAR (https://metadatacenter.org), NIDM (http://nidm.nidash.org), open metadata markup language (odML) [43, Y]), data representation models (Neo [143]), data analysis tools (e.g., Electrophysiology Analysis Toolkit (Elephant; https://python-elephant.org), FieldTrip (https://www.fieldtriptoolbox.org/) [Z]), simulation interfaces (e.g., PyNN [142]), etc."
with additional reference for NIX (#139 is the corresponding INCF standards document, which is fine to cite, but does not credit the software as such)
[X] Stoewer A, Kellner CJ, Benda J, Wachtler T and Grewe J (2014). File format and library for neuroscience data and metadata. Front. Neuroinform. Conference Abstract: Neuroinformatics 2014. doi: 10.3389/conf.fninf.2014.18.00027
A potential additional reference to odML fitting the papers context could be
[Y] Sprenger, J., Zehl, L., Pick, J., Sonntag, M., Grewe, J., Wachtler, T., GrÃ¼n, S., Denker, M., 2019. odMLtables: A User-Friendly Approach for Managing Metadata of Neurophysiological Experiments. Front. Neuroinform. 13, 62. https://doi.org/10.3389/fninf.2019.00062
Fieldtrip has a citable paper:
[Z] Robert Oostenveld, Pascal Fries, Eric Maris, and Jan-Mathijs Schoffelen. FieldTrip: Open Source Software for Advanced Analysis of MEG, EEG, and Invasive Electrophysiological Data. Computational Intelligence and Neuroscience, 2011; 2011:156869.
p.34, lines 7-12: A better link is: https://www.incf.org/sig/incf-working-group-standardized-data

p.36: Remove : after headings
p.37, lines 2-3: Sentence broken
p.38, lines 8-22: The paragraph is contradicting in that first datalad is advertised as a data sharing platform, then its denied. In fact, to my knowledge, DataLad is a tool for management, not a service.
p.39, lines 14-19: The sentences were unclear to me. Please clarify.

Methods

Are the methods appropriate to the aims of the study, are they well described, and are necessary controls included? Choose an item.

Conclusions

Are the conclusions adequately supported by the data shown? Choose an item.

Reporting Standards

Does the manuscript adhere to the journal's guidelines on minimum standards of reporting? Choose an item.

Choose an item.

Statistics

Are you able to assess all statistics in the manuscript, including the appropriateness of statistical tests used? Choose an item.

Quality of Written English

Please indicate the quality of language in the manuscript: Choose an item.

Declaration of Competing Interests

Please complete a declaration of competing interests, considering the following questions:

- Have you in the past five years received reimbursements, fees, funding, or salary from an organisation that may in any way gain or lose financially from the publication of this manuscript, either now or in the future?
- Do you hold any stocks or shares in an organisation that may in any way gain or lose financially from the publication of this manuscript, either now or in the future?
- Do you hold or are you currently applying for any patents relating to the content of the manuscript?
- Have you received reimbursements, fees, funding, or salary from an organization that holds or has applied for patents relating to the content of the manuscript?
- Do you have any other financial competing interests?

- Do you have any non-financial competing interests in relation to this paper?

If you can answer no to all of the above, write 'I declare that I have no competing interests' below. If your reply is yes to any, please give details below.

I declare that I have no competing interests

I agree to the open peer review policy of the journal. I understand that my name will be included on my report to the authors and, if the manuscript is accepted for publication, my named report including any attachments I upload will be posted on the website along with the authors' responses. I agree for my report to be made available under an Open Access Creative Commons CC-BY license (http://creativecommons.org/licenses/by/4.0/). I understand that any comments which I do not wish to be included in my named report can be included as confidential comments to the editors, which will not be published.

Choose an item.

To further support our reviewers, we have joined with Publons, where you can gain additional credit to further highlight your hard work (see: https://publons.com/journal/530/gigascience). On publication of this paper, your review will be automatically added to Publons, you can then choose whether or not to claim your Publons credit. I understand this statement.

Yes Choose an item.
